# Incidence and risk factors of acute kidney injury after abdominal surgery: a systematic review and meta-analysis

**DOI:** 10.1080/07853890.2025.2547324

**Published:** 2025-08-17

**Authors:** Jian Liu, Shi-hui Lin, Yi-si Zhao, Ren-jie Luo, Zheng-tao Zhang, Liu-yang Wang, Ke Xie, Jing Fan, Mu Zhang, Yu-sen Chai, Hong Tang, Fang Xu

**Affiliations:** a Department of Critical Care Medicine, The First Affiliated Hospital of Chongqing Medical University, Chongqing, P.R. China; b Department of Critical Care Medicine, Youyang Hospital, A Branch of the First Affiliated Hospital of Chongqing Medical University, Chongqing, P.R. China; c Pulmonary Engineering Group, Department of Anaesthesiology and Intensive Care Medicine, University Hospital Carl Gustav Carus Dresden at Technische Universität Dresden, Dresden, Germany

**Keywords:** Acute kidney injury, abdominal surgery, incidence, risk factor, systematic review

## Abstract

**Objective:**

To determine the incidence of acute kidney injury (AKI) following abdominal surgery, assess its outcome associations, and identify factors associated with postoperative AKI development.

**Methods:**

We performed a systematic search of PubMed, Embase, and Cochrane Database of Systematic Reviews, from January 2004, to December 2024. We included studies reporting AKI based on consensus criteria (RIFLE, AKIN, or KDIGO) in adult abdominal surgery patients.

**Results:**

A total of 162 studies (675361 patients) were included. The pooled AKI incidence was 16% (95% CI: 14-17%), with significant variation by surgical procedure. Meta-analysis showed AKI was significantly associated with increased short-term mortality (risk ratio [RR], 6.46; 95% CI: 4.63–9.00) and long-term mortality (RR, 6.36; 95% CI: 3.32–12.16). Mortality risk demonstrated stage-dependent increase, with RR of 2.74 (95%CI: 1.77–4.24), 8.01 (95%CI: 3.18–20.18), and 15.73 (95%CI: 5.52–44.81) for AKI stages 1, 2, and 3, respectively. AKI was associated with prolonged hospital stay (weighted mean difference 4.72 days; 95%CI: 3.43–6.02), also showeing stage-dependent increase of 5.03, 11.16, and 14.46 days for stages 1, 2, and 3, respectively. Twenty-five risk factors were associated with AKI. Meta-analysis of randomized controlled trials revealed that individualized blood pressure target management significantly reduced AKI incidence (RR, 0.67; 95% CI: 0.52–0.88).

**Conclusions:**

AKI remains a common and important complication after abdominal surgery, with severity showing a graded association with mortality and hospital stay. Individualized blood pressure management demonstrates promise in AKI prevention.

**Registration:**

PROSPERO CRD42022304083.

## KEY MESSAGES

1. The incidence of postoperative AKI after abdominal surgery is 16% (95% CI: 14–17%), varying by specific abdominal surgical procedure but not significantly different over time or by consensus definition.
2. AKI severity shows a strong graded association with both short-term and long-term mortality, as well as prolonged hospital stays.
3. Twenty-five factors were identified, providing valuable information for clinical risk assessment.
4. Meta-analysis of randomized trials reveals that individualized blood pressure target management significantly reduces AKI incidence, while other single perioperative interventions (crystalloids versus colloids, restrictive versus liberal fluid management, cardiac output-guided therapy, hemodynamic monitoring) show no significant protective effects.

## Background

Acute kidney injury (AKI) occurs in approximately 17% of hospitalized patients worldwide, representing its average global incidence [1]. Majority occur as a sequela of abdominal surgery [2]. The development of postoperative AKI is associated with longer hospital length of stay (LOS), mortality, post-discharge readmission rates, and development of chronic kidney disease (CKD) [3,4]. Patients with AKI have a significantly higher risk of developing new or progressive CKD (hazard ratio 2.67, 95% confidence interval [CI]1.99–3.58; 17.76 vs 7.59 cases per 100 person-years) and end-stage kidney disease (hazard ratio 4.81, 95% CI 3.04–7.62; 0.47 vs 0.08 cases per 100 person-years), relative to non-AKI patients [5]. Thus, AKI should be recognized as a potential indicator of adverse effects associated with post-abdominal surgery. A previous systematic review of 19 studies with 82,514 patients reported that the incidence of AKI after major abdominal surgery was 13.4% [6]. Advances in perioperative management, supportive interventions in the intensive care unit, and surgical techniques have been developed since 2016. However, dozens of studies have reported a high incidence of AKI, and some even occurred in patients undergoing non-major abdominal surgery [7–11].

Different mechanisms are involved in the development of postoperative AKI with a complex and multifactorial etiology. Renal hypoperfusion, high intra-abdominal pressure, sepsis, and/or nephrotoxic exposure are classical mechanisms of postoperative AKI [12–16]. This has led to a lack of a single and effective renoprotective strategy to protect surgical patients from AKI. Recently, a series of studies have explored and identified risk factors associated with an increased risk of postoperative AKI, such as older age [17], higher body mass index (BMI) [18,19], hypertension [19,20], diabetes mellitus [8,21], red blood cell (RBC) suspension transfusion [8,11,20], and the use of synthetic colloid [10,20,22], among others. Notably, conflicting results exist in the literature regarding certain risk factors. For instance, intraoperative RBC transfusion was identified as a significant risk factor in Kim et al.’s study (odds ratio [OR] = 1.58; 95%CI: 1.06–2.35) [20] but failed to reach statistical significance in Mahmooth et al. research (OR = 2.2; 95%CI: 0.6–8.06) [11]. Similarly, diabetes showed no statistical significance in the STARSurg Collaborative study (OR = 1.16; 95%CI: 0.79–1.7) [23] but emerged as a significant risk factor in Ji Hoon Sim’s research (OR = 2.77; 95%CI: 1.16–6.58) [24].

Perioperative hemodynamic changes play a crucial role in the development of postoperative AKI. Salmasi et al. demonstrated that intraoperative mean arterial pressure (MAP) <65 mmHg or >20% decrease from baseline was progressively associated with postoperative AKI risk, with longer exposure times correlating with higher risk [25]. The subsequent analysis of the POISE-2 trial revealed that sustained postoperative systolic blood pressure below 90 mmHg was associated with increased AKI risk, particularly when combined with anemia [26]. However, the optimal perioperative blood pressure management strategy remains controversial. The recent POISE-3 trial compared an avoid-hypotension strategy (targeting intraoperative MAP ≥80 mmHg) with an avoid-hypertension strategy (targeting MAP ≥60 mmHg) and found no significant difference in postoperative AKI incidence [27]. These findings highlight the complexity of perioperative management. Although several validated postoperative AKI risk assessment tools exist (such as the NSQIP calculator, Thakar score, and SPARK scoring system), they lack comprehensive systematic evaluation specifically for the abdominal surgery population.

Therefore, we conducted this systematic review and meta-analysis to determine the proportion of postoperative AKI according to contemporary consensus criteria [28–30] and to further elucidate associated influence factors, providing evidence for the prevention and management of postoperative AKI.

## Methods

The following three consensus definitions of AKI were used: Risk, Injury, Failure, Loss, End-Stage (RIFLE), Acute Kidney Injury Network (AKIN), and Kidney Disease Improving Global Outcomes (KDIGO) [28–30]. Given that the three consensus definitions mentioned above were the result of continuous change, improvement, and integration, if multiple definitions are used for a study, we extract AKI data in the following order of priority: KDIGO > AKIN > RIFLE.

Previous meta-analyses have demonstrated that the incidence of AKI after liver transplant (LT) and open abdominal aortic aneurysm (OAAA) surgery is higher (47 and 24%, respectively) than that after other abdominal procedures (such as colorectal, hepatobiliary, and gastrointestinal procedures) [6]. If analyzed together, the incidence of AKI after LT and OAAA would carry excessive weight; therefore, they were not included in the main analysis. The data are presented in the supplemental file (Supplementary Table S1).

### Systematic literature search

As the consensus definition of AKI was designed and published in or after 2004 [29], we conducted a systematic search of PubMed, Embase, and the Cochrane Database of Systematic Reviews, from January 1, 2004 to December 31, 2024. The following search themes were used: (((Surgical Procedures, Operative[MeSH]) OR (General Surgery[MeSH])) AND ((Acute Kidney Injury[MeSH]) OR (Renal Insufficiency[MeSH]))) OR ((Risk[MeSH]) OR (Incidence[MeSH])). If more than one publication contained overlapping research subjects, we decided which publication would be included based on the completeness of reporting results. This study was conducted according to the recommendations of the PRISMA reporting guideline [31]. This study was registered in the International Prospective Register of Systematic Reviews (PROSPERO; CRD42022304083).

### Inclusion criteria

1. Adult patient undergoing abdominal cavity surgery
2. The RIFLE, AKIN, or KDIGO diagnosis and classification criteria were used to evaluate postoperative AKI.
3. Data for OR and the corresponding CI are available for the risk factor of AKI after abdominal surgery.
4. No language restriction

### Exclusion criteria

1. Urological procedure
2. Caesarean section
3. Data on AKI is not available for the abdominal surgery group
4. AKI diagnosis was based on diagnostic coding or the receipt of renal replacement therapy
5. Type of article: Case study, review, editorial, non-human study, letter, and conference paper

### Factors associated with postoperative AKI

Based on existing literature [32] and clinical experience, we selected factors associated with postoperative AKI encompassing eight major categories: demographic characteristics, preoperative status assessment, comorbidities, perioperative medication exposure, surgery-related factors, intraoperative management factors, laboratory parameters, and pathological conditions.

### Outcome measures

The primary outcomes of this study include the incidence of AKI in patients following abdominal surgery and the OR and corresponding CI for the influence factors of post-operative AKI. The secondary outcome was the number of patients with AKI according to stage, hospital LOS, and mortality. Studies reporting hospital or < 30-day mortality were defined as short-term mortality. Long-term mortality was defined as mortality > 30 days. If both univariate and multivariate analyses for OR were conducted, the latter was the first choice because the results were adjusted for confounding factors.

### Study selection and data extraction

Each study was independently evaluated by two reviewers (J.L. and S.H.L.), first for title and abstract screening and second for full-text careful reading. Data from the included primary studies were independently extracted by two reviewers (J.L. and S.H.L.) using pre-formulated forms. Disagreements were resolved by a third author (M. Z.). Two reviewers (H.T. and F.X.) reviewed the manuscript.

### Quality assessment

We used the Risk of Bias in Non-randomized Studies of Exposures (ROBINS-E) tool to assess the risk of bias in observational studies across seven domains: (Domain 1) bias due to confounding, (Domain 2) bias in measurement of exposure, (Domain 3) bias in selection of participants, (Domain 4) bias due to post-exposure interventions, (Domain 5) bias due to missing data, (Domain 6) bias in measurement of outcomes, and (Domain 7) bias in selection of reported results. Each study was rated as having low, moderate, high or very high risk of bias for each domain and overall [33]. The risk of bias in the randomized controlled trials (RCT) was assessed using the Cochrane risk-of-bias tool. All quality assessments were conducted independently by two reviewers (J.L. and S.H.L.).

## Analysis

Statistical analyses were performed using Stata/SE (version 17.0; StataCorp, LLC). The random-effects model for meta-analysis was utilized to calculate the overall pooled proportion of AKI among all included studies, the risk ratio (RR) of mortality in patients with AKI, the weighted mean difference in hospital LOS, and the ORs of factors associated with postoperative AKI. Subgroup analysis was conducted for studies with different definitions of AKI, surgical settings, and years of publication. To explore the impact of each study on the overall findings, we conducted a leave-one-out meta-analysis by removing each study from the meta-analysis. Potential publication bias was assessed using the Egger’s test. Between-study heterogeneity was assessed using the I^2^ index. If the I^2^ statistic was 50% or higher, heterogeneity was considered significant.

## Results

### Characteristics of the included studies

**Figure 1.**
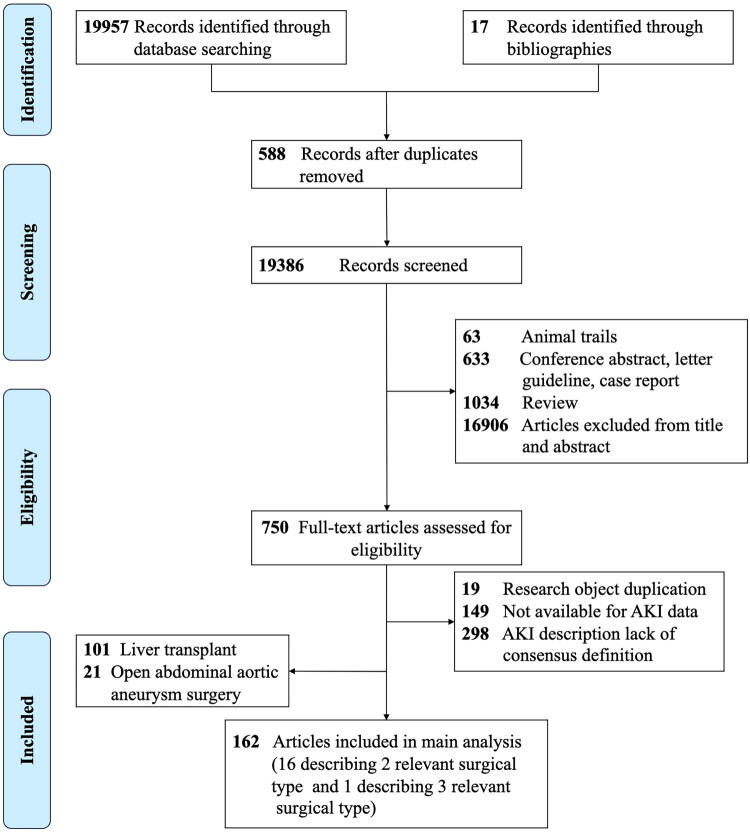
Flow diagram for study selection.

The study selection process is illustrated in Figure 1. In total, 19974 studies were screened. A total of 750 studies were identified for full-text review, which were excluded because of a lack of consensus on the definition of AKI (*n* = 298), unstated incidence of AKI (*n* = 149), and research object duplication (*n* = 19) (Supplementary Table S2). In total, 162 studies satisfied the pre-specified inclusion criteria (Supplementary Table S3) [7,9–11,17–22,24,25,34–183]. Of the 162 included studies, 158 were observational studies [7,9–11,17–20,22, 24,25,34–79,81–86,88–111,113–183], and four were RCT studies[21,80,87,112]. Risk of bias assessment using ROBINS-E revealed that most included studies had low to moderate risk of bias across the seven domains (Supplementary Table S4). The majority of studies demonstrated low risk in participant selection (D3), outcome measurement (D6), and selective reporting (D7). However, moderate risk was commonly observed in confounding bias (D1) and missing data (D5). A small number of studies were classified as having high overall risk of bias, primarily due to inadequate control of confounding factors and high attrition rates. Four of the included RCTs reported the generation of random sequences. Two studies described allocation concealment [21,87]. One study masked the trial to all participants and investigators [21]. Two studies masked the trial to outcome assessors [21,80]. All studies included the same outcome indicators in their reporting as the initially planned research project indicators, and no omissions in the outcome indicators were identified (Supplementary Figure S1). Sixteen studies reported that patients underwent two types of surgery [25,38,42,47,51,64,68,72,73,86,96,139,162,171,172,179]. One study reported that patients underwent three types of surgery, and the number of AKI events in patients undergoing gynecological surgery was 0. These issues were addressed in two separate studies. The included studies reported the following surgical settings: mixed abdominal surgery (72 studies), digestive surgery (46 studies), gynecological surgery (18 studies), hepatobiliary surgery (22 studies), pancreatic surgery (11 studies), cytoreductive surgery plus hyperthermic intraperitoneal chemotherapy with cisplatin (CRS and HIPEC) (eight studies), and bariatric surgery (three studies).

### Incidence of AKI

The meta-analysis of the pooled incidence of AKI was 16% (14–17%, 95% CI), with subgroup analyses presented in Table 1 and the forest plot of individual study incidence rates shown in Supplementary Figure S2. The between-study heterogeneity was considerable (I^2^ = 99.90%). We conducted a leave-one-out meta-analysis, which revealed that the results were robust. Egger’s test (*t* = 4.81, *p* < 0.01) suggested the presence of publication bias; however, the trim-and-fill imputed no missing data. The nonparametric trim-and-fill analysis of 180 studies showed that no studies needed to be fille. Visual inspection of funnel plots with contour lines at 1, 5, and 10% significance levels further confirmed no significant publication bias in this meta-analysis (Supplementary Figure S3).

**Table 1.** Meta-analysis of the proportion of patients developing postoperative AKI.

	No. of studies	No. of patients	Incidence of AKI	95%CI	Heterogeneity	
					*I* ^2^	*τ* ^2^
All patients	180	675361	16%	14–17%	99.90%	0.02
AKI definition						
AKIN	36	62285	13%	10–16%	99.41%	0.01
KDIGO	127	538532	15%	13–18%	99.91%	0.02
RIFLE	17	74544	21%	13–29%	99.84%	0.03
Surgery type						
Mixed abdominal surgery	72	422095	19%	12–22%	99.94%	0.02
Digestive surgery	46	139470	15%	12–18%	99.80%	0.01
Gynecological surgery	18	87066	6%	4–8%	98.78%	0.00
Hepatobiliary surgery	22	15338	12%	9–14%	97.29%	0.00
Pancreatic surgery	11	7067	11%	8–14%	94.07%	0.00
CRS and HIPEC	8	2083	25%	11–38%	98.8%	0.04
Bariatric surgery	3	2242	11%	4–17%	95.87%	0.00
Year of study publication						
2021–2024	78	412038	11%	8–15%	98.22%	0.00
2011–2020	98	247500	17%	14–20%	99.84%	0.02
2004–2010	4	15823	18%	3–33%	99.81%	0.02

Abbreviations: AKI, acute kidney injury; CRS, cytoreductive surgery; HIPEC, hyperthermic intraperitoneal chemotherapy.

As shown in Table 1, the incidence of AKI varied significantly among different types of abdominal surgery (*p* < 0.01). Gynecological procedures demonstrated the lowest AKI incidence at 6% (95% CI: 4–8%), which was notably lower than other surgical categories. Pancreatic surgery (11%, 95% CI: 8–14%), bariatric surgery (11%, 95% CI: 4–17%), and hepatobiliary surgery (12%, 95% CI: 9–14%) showed relatively low AKI incidence rates. Digestive surgery exhibited an AKI incidence of 15% (95% CI: 12–18%), while mixed abdominal procedures showed a rate of 19% (95% CI: 12–22%). Notably, CRS and HIPEC demonstrated the highest AKI incidence at 25% (95% CI: 11–38%), but the wide confidence interval suggests limited precision in this estimate. Importantly, our analysis revealed no statistically significant differences in AKI incidence rates across different AKI definition criteria (*p* = 0.12) or among studies from different publication years (*p* = 0.23).

Furthermore, we compared the 122 studies that we analyzed between LT and OAAA and found a remarkable difference in the incidence of AKI (*p* < 0.01). The incidences of AKI in the LT and OAAA groups were 46 and 33%, respectively. Of the 66 studies that reported the number of patients with AKI according to stage, 72% of patients with AKI were in stage 1 or RIFLE-R, 16% of patients with AKI were in stage 2 or RIFLE-I, and 12% of patients with AKI were in stage 3 or RIFLE-F (Supplementary Table S5) [11,17, 18,20,22,34–36,41,43,46,49,50,54,61,66,69,76,80,83,87–91,93,95–97,100,109,110,112,115,118–120,123, 125,126,128,131,133,134,138,143–147,149,150,154–160,175,181,182].

### Length of hospital stay

**Figure 2.**
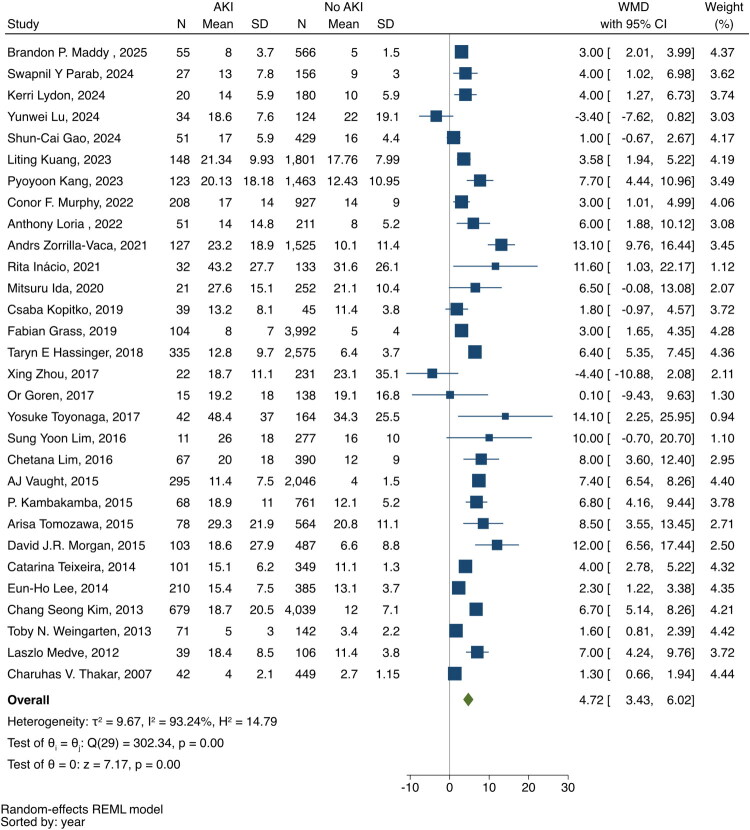
Forest plot of hospital LOS of patients who developed postoperative AKI and those who did not.

A total of 33 studies reported LOS (Supplementary Table S6) [19,35,37,39,41,44,54,65,69,74, 79,82,83,91,92,97, 100,106,107,109,111,115,117,119,124,128,133,134,138,147,149,156,157]. Three studies were not included in the meta-analysis: two reported the mean length of stay without standard deviation [82,107], and another defined patients without AKI as non-AKI and AKI stage 1 [83]. Meta-analysis results suggested that patients with AKI had longer hospital stays than non-AKI patients (weighted mean difference [WMD], 4.72 days; 95%CI: 3.43–6.02) (Figure 2). The between-study heterogeneity was significant (*I*^2^ = 93.24). Additionally, we performed a leave-one-out meta-analysis, which revealed that the results were robust.

## Mortality

**Figure 3.**
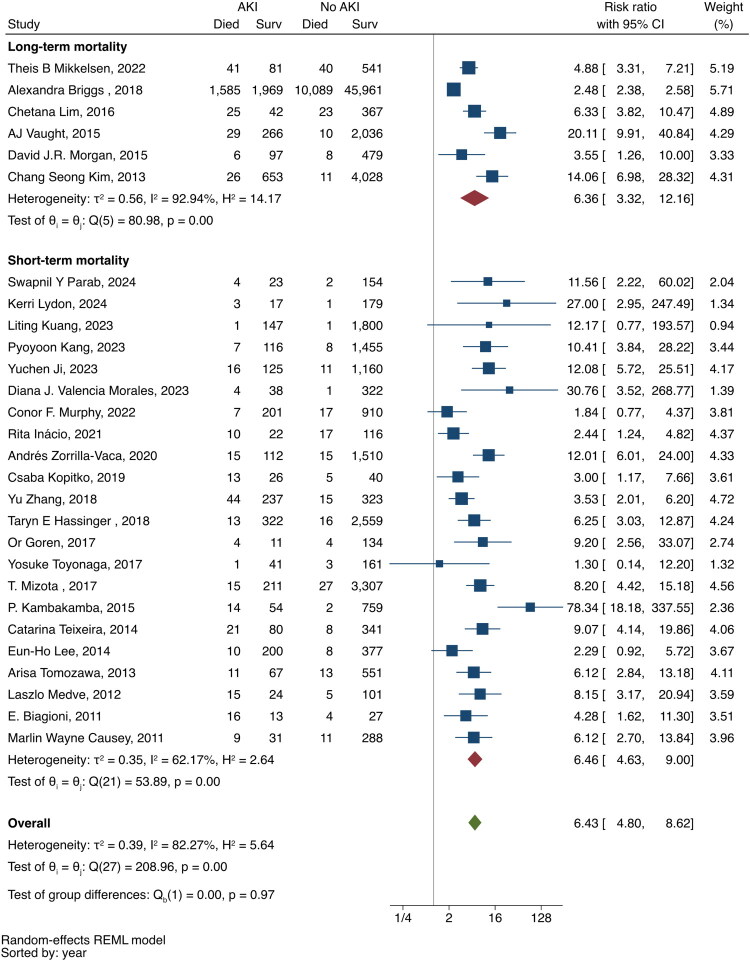
Forest plot of the risk ratio of the association between AKI and mortality.

A total of 32 studies documented the mortality of patients with or without AKI (Supplementary Table S7) [19,35,37,39–41,44,54,65,74,78,82,83,91,92,97,104,106,109–111,115,117,128,133,134,147,149,150,152, 155,156]. Three studies were not included in the meta-analysis: two reported no deaths in non-AKI patients [91,106] and one defined patients without AKI as non-AKI and AKI stage 1 [83]. Meta-analysis of the pooled RR for short-term, long-term, and overall mortality for patients with AKI compared to patients without AKI is 6.46 (95%CI, 4.63–9.00), 6.36 (95%CI, 3.32–12.156), and 6.43 (95%CI, 4.80–98.62) respectively (Figure 3). The I^2^ values for short-term, long-term, and overall mortalities were 62.17, 92.94, and 82.27%, respectively. A leave-one-out meta-analysis showed that these results were robust.

### AKI staging, timing, and clinical outcomes

Among the included studies, 9 studies reported mortality outcomes [35,41,54,83,90,92,97,150,155], 4 studies included length of hospital stay data [35,90, 97,155], and 3 studies provided complications data stratified by AKI stages [35,150,155]. Meta-analysis demonstrated a graded association between AKI stage and clinical outcomes. It should be noted that these analyses were conducted as post-hoc analyses, rather than a pre-planned analyses.

Subgroup analysis by AKI stages showed a stepwise increase in mortality association compared to patients without AKI: Stage 1 (RR, 2.74; 95%CI: 1.77–4.24), Stage 2 (RR, 8.01; 95%CI: 3.18–20.18), and Stage 3 (RR, 15.73; 95%CI: 5.52–44.81) (Figure S4). Similarly, analysis by AKI stages demonstrated progressive increases in hospital LOS with increasing AKI severity: compared to patients without AKI: Stage 1 patients stayed 5.03 (95%CI, 3.61–6.46) days longer, Stage 2 patients 11.16 (95%CI, 9.06–13.26) days longer, and Stage 3 patients 14.46 (95%CI, 6.48–22.45) days longer (p < 0.001) (Figure S5).

Three studies reported complications stratified by AKI stages (Table S8). Despite limited data, they consistently demonstrated higher complication rates in patients with more severe AKI stages, with Stage 3 AKI patients having the highest rates of surgical, pulmonary, and cardiovascular complications. Regarding timing, AKI diagnosis varied significantly among included studies, ranging from immediately after surgery to 30 days postoperatively. Three studies provided detailed temporal analysis: early AKI (within 48 h, 7.9%) was primarily associated with intraoperative factors, while late AKI (48 h–7 days, 3.2%) was related to both preoperative comorbidities and intraoperative factors [182]. Studies distinguishing transient versus persistent AKI found that non-transient AKI (persisting beyond postoperative day 1) was associated with higher complication rates and persistent renal dysfunction [178]. In bariatric surgery, early AKI occurred in 71 patients (within 72 h) versus 30 with late-onset AKI (72 h–30 days), the latter primarily due to volume depletion and complications, increasing readmission rates [100].

## Factors associated with postoperative AKI

Our meta-analysis identified multiple factors associated with postoperative AKI, but these findings should be interpreted with caution as some of these factors were not adjusted for potential confounding variables.

**Figure 4.**
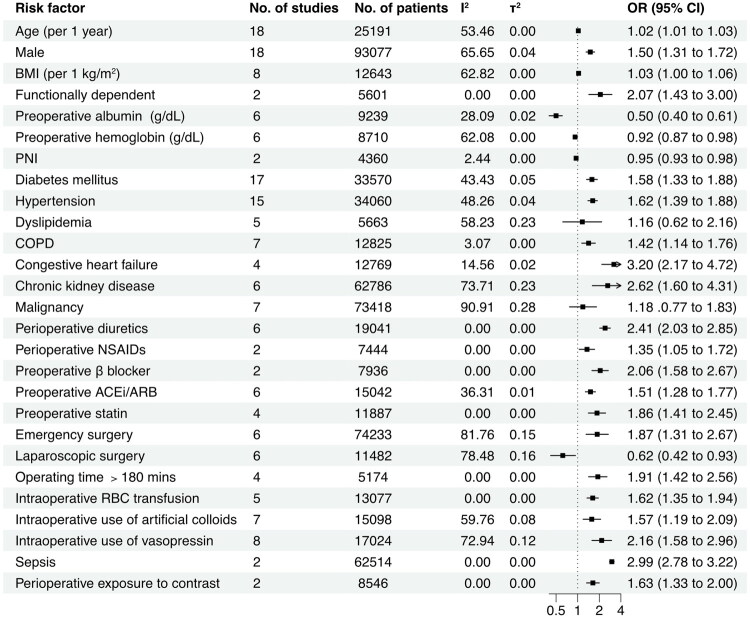
Forest plot of the risk factors for postoperative AKI. Abbreviations: ACEi, angiotensin-converting enzyme inhibitor; ARB, angiotensin II receptor blocker; BMI, body mass index; COPD, chronic obstructive pulmonary disease; NSAID, non-steroidal anti-inflammatory drug; RBC, red blood cell. PNI, prognostic nutritional index, PNI= [10 × serum albumin (g/dL)] + [0.005 × total lymphocyte count (per mm^3^)].

As shown in Figure 4, the pooled analysis of risk factors for AKI after abdominal surgery indicates the following showed positive associations with AKI: age (per 1-year increment) (OR, 1.02; 95% CI: 1.01–1.03; *p* < 0.01), male (vs. female, OR, 1.50; 95% CI: 1.31–1.72; *p* < 0.01), BMI (per 1 kg/m^2^ increment) (OR, 1.03; 95% CI: 1.00–1.06; *p* = 0.02), diabetes mellitus (OR, 1.58; 95% CI: 1.33–1.88; *p* < 0.01), congestive heart failure (OR, 3.20; 95% CI: 2.17–4.72; *p* < 0.01), hypertension (OR, 1.62; 95% CI: 1.39–1.88; *p* < 0.01), chronic obstructive pulmonary disease (COPD) (OR, 1.42; 95% CI: 1.14–1.76; *p* < 0.01), CKD (OR,2.62; 95% CI: 1.60–4.31; *p* < 0.01), sepsis (OR, 2.99; 95% CI: 2.78–3.22; *p* < 0.01), perioperative exposure to contrast agents (OR, 1.63; 95% CI: 1.33–2.00; *p* < 0.01), perioperative use of diuretics (OR, 2.41; 95% CI: 2.03–2.85; *p* < 0.01), perioperative nonsteroidal anti-inflammatory drugs (NSAIDs) (OR, 1.35; 95% CI: 1.05–1.72; *p* = 0.02), preoperative use of *β* blockers (OR, 2.06; 95% CI: 1.58–2.67; *p* < 0.01), preoperative use of angiotensin-converting enzyme inhibitors/angiotensin II receptor blockers (ACEi/ARB) (OR, 1.51; 95% CI: 1.28–1.77; *p* < 0.01), preoperative use of statins (OR, 1.86; 95% CI: 1.41–2.45; *p* < 0.01), emergency surgery (OR, 1.87; 95% CI: 1.31–2.67; *p* < 0.01), intraoperative red blood cell transfusion (OR, 1.62; 95% CI: 1.35–1.94; *p* < 0.01), intraoperative use of artificial colloids (OR, 1.57; 95% CI: 1.19–2.09; *p* < 0.01), intraoperative use of vasopressin (OR,2.16; 95% CI: 1.58–2.96; *p* < 0.01), and surgery duration over 180 min (OR, 1.91; 95% CI: 1.42–2.56; *p* < 0.01). Hemoglobin (per 1 g/dL increase, OR, 0.92; 95% CI: 0.87–0.98; *p* = 0.01), albumin (per 1 g/dL increase, OR, 0.50; 95% CI: 0.40–0.61; *p* < 0.01), prognostic nutritional index (PNI) (OR, 0.95; 95% CI: 0.93–0.98; *p* < 0.01), and laparoscopic surgery (OR, 0.62; 95% CI: 0.42–0.93; *p* = 0.02) showed negative associations with AKI postoperative AKI. Dyslipidemia (OR, 1.16; 95% CI: 0.62–2.16; *p* = 0.65) and malignancy (OR, 1.18; 95% CI: 0.77–1.83; *p* = 0.65) demonstrated no significant association with postoperative AKI. A detailed summary of all the identified influence factors is provided in Supplementary Table S9.

### Effects of perioperative interventions on postoperative AKI

To comprehensively evaluate the effects of perioperative interventions on postoperative AKI, we additionally retrieved RCTs related to perioperative management. It should be noted that these studies included various surgery types (such as abdominal surgery, orthopedic surgery, urological surgery, etc.), which do not fully meet the original inclusion criteria of this study and thus are presented separately as supplementary analyses. A total of 30 RCTs were included, and detailed baseline characteristics and risk of bias are provided in (supplementary file Table S10, Figure S1) [2,27,87,112,184–209].

**Figure 5.**
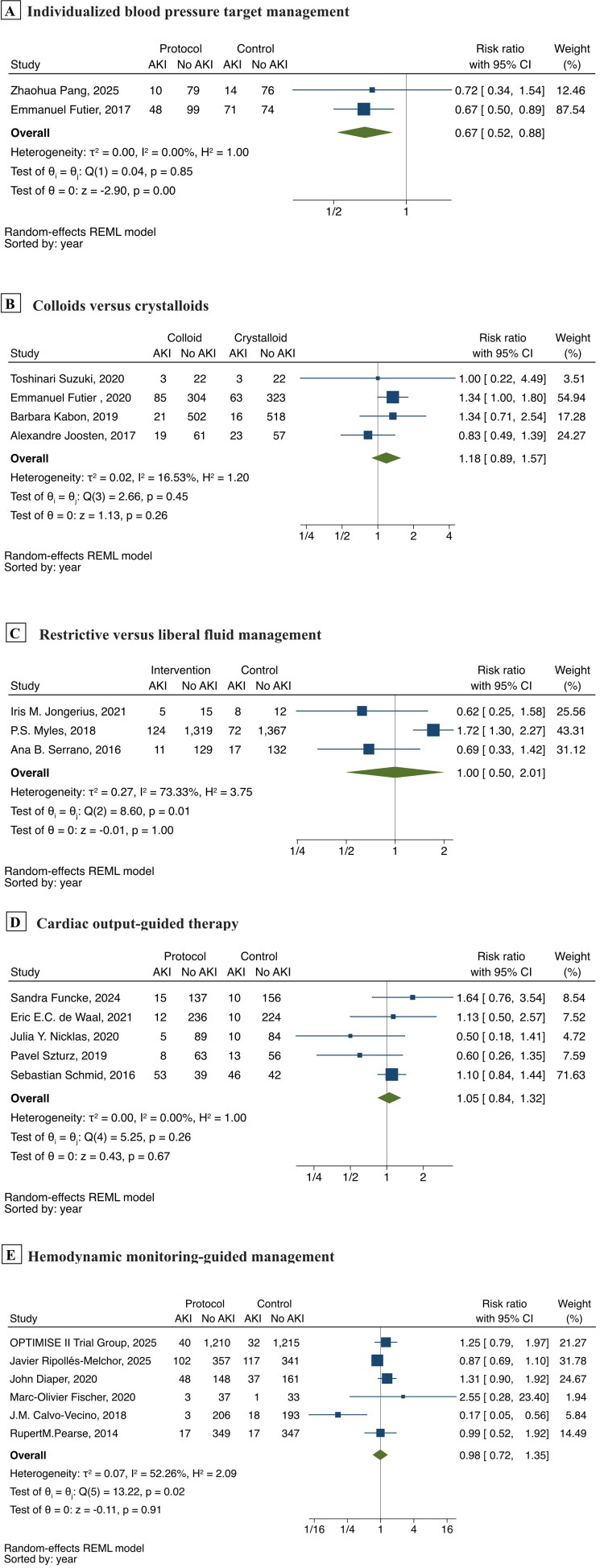
Meta-analysis of perioperative hemodynamic interventions on postoperative acute kidney injury. Forest plots showing risk ratios and 95% CI for different perioperative hemodynamic intervention strategies. (A) Individualized blood pressure target management; (B) Colloids versus crystalloids; (C) Restrictive versus liberal fluid management; (D) Cardiac output-guided therapy; (E) Hemodynamic monitoring-guided management.

Among these, studies related to perioperative hemodynamic management strategies were eligible for meta-analysis (Figure 5). The results showed that individualized blood pressure target management (*n* = 471) significantly reduced postoperative AKI incidence (RR,0.67; 95% CI: 0.52–0.88) [185,207]; the POISE-3 trial (*n* = 7006) compared hypotension-avoidance strategy (MAP ≥ 80mmHg) versus hypertension-avoidance strategy (MAP ≥ 60mmHg) and found no significant difference in AKI incidence (15.1 vs 14.4%) [27]; compared with crystalloids, colloids (*n* = 2042) did not significantly increase the risk of postoperative AKI (RR, 1.18; 95% CI: 0.89–1.57) [2,193,197,198]; restrictive vs. liberal fluid management (*n* = 3211) showed no significant difference in AKI incidence (RR, 1.00; 95% CI: 0.50–2.01) [87,192,208]; cardiac output-guided therapy (*n* = 1308) demonstrated no protective effect against AKI (RR, 1.05; 95% CI: 0.84–1.32) [112,187,189,191,202]; hemodynamic monitoring-guided management (*n* = 5032) also showed no significant benefit (RR, 0.98; 95% CI: 0.72–1.35) [184,196,201,203, 204,206].

Additionally, several other perioperative interventions were evaluated in RCTs, but due to the heterogeneity of interventions, these studies could not be quantitatively combined. Regarding perioperative respiratory management, a large RCT found no significant difference in AKI incidence between low tidal volume and conventional tidal volume ventilation [209]. Similarly, other interventions, including individualized positive end-expiratory pressure, open lung strategy, and varied inspired oxygen concentrations, showed no significant impact on postoperative AKI rates compared to standard care [188,190,195]. In terms of fluid management, a study of 90 patients undergoing emergency laparotomy found that isotonic sodium bicarbonate infusion, compared with balanced salt solution, improved perioperative acid-base balance and significantly reduced postoperative AKI incidence (9 vs 24%) [186]. Another study that preoperative intravenous iron supplementation to treat anaemia showed no significant impact on postoperative AKI rates [194]. Other perioperative interventions, such as preoperative focused cardiac ultrasound for individualized anesthesia and perioperative aspirin administration, also did not shown significant differences in their effects on postoperative AKI [199,205].

## Discussion

This systematic review summarizes the results of 162 studies conducted on 675361 adult patients who underwent abdominal surgery. The incidence of AKI after abdominal surgery is approximately 16%. Twenty-seven variables influencing the development of AKI were analyzed. The study identified twenty-one risk factors associated with an increased risk of AKI and four protective factors associated with a decreased risk of AKI following abdominal surgery. Moreover, two factors showed no significant association.

Through a meta-analysis, we found a strong association between postoperative AKI and short-term (RR, 6.46; 95%CI 4.63–9.00), long-term (RR, 6.36; 95%CI 3.32–12.16), and overall (RR, 6.43; 95%CI 4.63–9.00) mortalities. Although there was significant heterogeneity, consistent evidence suggests an association between AKI and risk of death. Some reports have proposed a relationship between post-abdominal surgery mortality and AKI. Vaca et al. reported that patients with postoperative AKI experienced increased hospital mortality (adjusted OR, 6.29; 95% CI 2.32–17.08) [19]. Beghdadi et al. reported similar results (adjusted OR, 5.09; 95%CI 1.13–22.8) [58]. This suggests that AKI has important predictive significance for the prognosis of patients after abdominal surgery [6]. Further analysis revealed a graded association between AKI stage and clinical outcomes. Among studies reporting detailed staging data, adverse outcomes increased progressively with AKI severity. The relative risk of mortality was nearly threefold higher in Stage 1 AKI patients compared to those without AKI (RR, 2.74; 95%CI: 1.77–4.24), eightfold higher in Stage 2 (RR, 8.01; 95%CI: 3.18–20.18), and nearly sixteen-fold higher in Stage 3 (RR, 15.73; 95%CI: 5.52–44.81). Hospital length of stay demonstrated a similar trend, with Stage 1, 2, and 3 AKI patients staying 5.03, 11.16, and 14.46 days longer respectively than patients without AKI (*p* < 0.01). Complications analysis further supported these findings, with three studies consistently showing increasing complication rates with AKI severity, particularly highest rates of surgical, pulmonary, and cardiovascular complications in Stage 3 AKI patients. This gradient relationship further confirms that AKI serves as an important marker of perioperative harm [6]. Notably, among 66 studies reporting AKI staging, 72% of AKI patients were in early stages. Previous research has shown that even minor postoperative increases in serum creatinine are associated with increased mortality and prolonged hospital stay [117], emphasizing the importance of early identification and intervention.

The temporal patterns of AKI demonstrated important clinical implications. While the diagnostic time window varied considerably among studies (immediate postoperative period to 30 days), detailed temporal analysis from three studies showed that early AKI was primarily associated with intraoperative factors, while late AKI was related to both preoperative comorbidities and intraoperative factors. Notably, non-transient AKI (persisting beyond postoperative day 1) was associated with higher complication rates and persistent renal dysfunction compared to very transient AKI (immediate postoperative period only).

Regarding some special cases of AKI, we reported that the incidence of AKI was lower than that of cardiac surgery–associated [210] and sepsis-associated AKI [211]. The incidences of AKI after LT and OAAA were 46 and 33%, respectively. O’Connor et al. suggested that AKI associated with such procedures should be considered in isolation [6]. The incidence of AKI after LT and OAAA procedures and subgroup analyses by year of publication and certain patient characteristics were not described in a pre-specified manner; these analyses can only be considered exploratory. Owing to the wide variation in baseline patient characteristics between studies, we did not conduct subgroup analyses according to whether the study population had CKD. Some reports confirmed the association between preoperative CKD and AKI following abdominal surgery [19,89]. This was confirmed by subsequent analysis of the risk factors for AKI after abdominal surgery.

We systematically screened the included studies to identify factors associated with postoperative AKI. Non-modifiable factors showing associations with postoperative AKI include advanced age [20–22,24, 35,37,50,52–54,74,75,79,88,90,92,94,111], male sex [10,17–20,22,24,37,41,44,53,74,78,90,100,111,116,121], and comorbidities such as diabetes mellitus [17–22,24,35,52,53,69,79,90,91,94,100,111], hypertension [17,19–22,24,41,52,53,74,79,90,94,100,111], COPD [19–21,41,54,74,90], congestive heart failure [20,35, 74,90], CKD [11,19,39,54,78,111], and sepsis [74,78]. Certain medications also show associations with AKI occurrence, including ACEi/ARB [20,44,94,106,109,121], NSAIDs [20,22,85], β-blockers [20,94], statins [20,44, 94,106,109,121], perioperative exposure to contrast [22,41], and diuretic use [17,20,41,53,94,111]. Preoperative nutritional status show associations with postoperative AKI. Higher preoperative albumin [20,24,44,53,109,117], hemoglobin [20,37,44,53,79,117], and PNI [24,53] levels show negative associations with AKI occurrence, whereas functional dependency [17,74] show positive associations.

Surgery-related factors are also documented in the literature. Emergency surgery poses higher AKI risk compared to elective surgery [20,35,37,78,90,94]. Minimally invasive laparoscopic surgery is associated with lower AKI risk compared to open abdominal surgery [19,37,53,88,116,121]. However, in patients with sepsis, the lower AKI risk associated with laparoscopic surgery should not be the sole determinant of the surgical approach selection. These factors should serve as early warning signs to enable prompt intervention. Our study also identified associations between AKI and intraoperative blood transfusions [11,20,24,41,53] and surgery duration (>180 min) [11,18,69,92].

Proper hemodynamic management is essential for optimal renal perfusion [119]. While our observational studies found significant associations between synthetic colloids use and increased postoperative AKI risk [17,20,22,24,37,53,111], and identified vasopressors as potential independent risk factors [11,20, 22,37,41,75,82,111], subsequent randomized controlled trials have provided higher-level evidence. Meta-analyses showed that individualized blood pressure target management significantly reduced postoperative AKI incidence (RR, 0.67; 95% CI: 0.52–0.88), though the POISE-3 trial found that a fixed hypotension-avoidance strategy (MAP ≥ 80 versus ≥60mmHg) did not show similar benefits. Compared with crystalloids, colloids did not significantly increase the risk of postoperative AKI (RR, 1.18; 95% CI: 0.89–1.57).

Fluid management strategies remain controversial. Observational studies have been limited by heterogeneity in fluid management variables, with studies examining different parameters such as total fluid input and fluid balance [19,21,24,53,85], making systematic comparison and meta-analysis challenging. Mahmooth et al. found no significant difference between nonrestrictive, restrictive, and ultra restrictive fluid protocols [11]. Meta-analysis further confirmed this finding, showing no significant difference in AKI incidence across fluid volume management strategies (RR, 1.00; 95% CI 0.50–2.01). Additionally, neither cardiac output-guided therapy (RR, 1.05; 95% CI: 0.84–1.32) nor advanced hemodynamic monitoring-guided perioperative management (RR, 0.98; 95% CI: 0.72–1.35) demonstrated protective effects against AKI.

Studies of other perioperative interventions have provided important insights. Regarding respiratory management, low tidal volume ventilation (6 mL/kg) compared with conventional tidal volume (10 mL/kg) did not significantly reduce postoperative incidence of AKI. Interventions such as individualized PEEP, open lung strategy, and different inspired oxygen concentrations showed no significant differences in postoperative incidence of AKI. However, in emergency laparotomy patients, isotonic sodium bicarbonate infusion compared with balanced salt solution significantly reduced postoperative AKI incidence. While preoperative rehabilitation exercise programs improved the Comprehensive Complications Index and quality of life in elderly patients, they showed no significant effect on postoperative AKI incidence.

These findings suggest that although observational studies have identified multiple potential risk factors, many perioperative interventions have not shown expected benefits in randomized controlled trials. This discrepancy may reflect the complex pathophysiology of postoperative AKI and the limitations of single interventions in prevention. Therefore, proper risk stratification and individualized management are paramount for optimizing the care of patients at risk for serious postoperative complication [17]. Future research should focus on multimodal intervention strategies while considering patient-specific characteristics and risk factors.

Based on these identified risk factors and our comprehensive analysis, we propose the following clinical implications to enhance the practical value of our findings. Drawing from the risk and protective factors identified in this study, clinicians can implement stratified management strategies: Perioperative physicians can conduct preoperative risk assessments and implement preventive measures for high-risk patients (such as elderly patients, those with chronic kidney disease, hypertension, or diabetes); anesthesiologists should focus on restoring circulating volume, maintaining circulating pressure, and ensuring optimal oxygen delivery; surgeons may consider minimally invasive techniques for high-risk patients to reduce inflammatory response. For identified high-risk patients, postoperative care should include increased frequency of urine output monitoring and renal function assessment to enable early detection and intervention of potential AKI.

Our study results provide foundational data for developing AKI risk scoring systems. Future research can integrate predictive risk factors to construct risk scoring systems; validate the accuracy and clinical utility of scoring tools through large-sample prospective study; and develop digital decision support tools to help clinicians quickly assess patients’ AKI risk and formulate corresponding preventive measures. Such risk stratification tools will help optimize medical resource allocation, enabling closer monitoring and early intervention for high-risk patients.

### Strengths and limitations

This study has several strengths. To our knowledge, this is the first systematic review and meta-analysis to evaluate the risk factors of AKI after abdominal surgery. This study is the most extensive and includes the largest systematic review and meta-analysis to assess the incidence of AKI. Subgroup analyses were conducted to explore sources of heterogeneity. The correlation with clinical practice is the ultimate strength of this study, and the results could lead to the early identification of patients at high risk of AKI and controllable risk factors associated with postoperative AKI by clinical practitioners.

This study has three limitations. First, although our research subjects were explicitly restricted to patients undergoing abdominal surgery, with a clear consensus definition of postoperative AKI, and most included studies had a low to moderate risk of bias, significant heterogeneity was observed in the incidence of AKI, adverse outcomes associated with AKI, and risk factors for AKI. This high degree of heterogeneity warrants further exploration as it may affect the generalizability of our findings. The heterogeneity primarily stems from multiple differentiating factors among the included studies. Abdominal surgery encompasses various procedures of different complexity levels, ranging from minimally invasive laparoscopic cholecystectomy to complex pancreaticoduodenectomy. Each procedure type varies in terms of surgical trauma, operative duration, and physiological impact. Our subgroup analysis revealed significant differences in AKI incidence rates across different surgical types (*p* < 0.01), potentially reflecting variations in hemodynamic changes, inflammatory responses, and organ perfusion status specific to each surgical setting. Moreover, although all studies employed standardized AKI definition criteria (RIFLE, AKIN, or KDIGO), practical application may have varied, including differences in serum creatinine measurement timing, accuracy of urine output monitoring, and methods for determining baseline renal function. The publication timeline span of included studies also reflects the evolution of perioperative management strategies, encompassing changes in fluid management approaches, advances in anesthetic techniques, and improvements in perioperative monitoring. Therefore, our reported 16% incidence rate of postoperative AKI should be interpreted as an overall estimate based on available evidence, rather than a universal single value applicable to all abdominal surgery patients. Clinicians should consider their specific practice environment and individual patient characteristics when assessing postoperative AKI risk. Second, although a comprehensive search of libraries was conducted, potential studies were excluded. Third, the proportion of studies reporting detailed AKI staging data was relatively limited in our research, which restricted our comprehensive assessment of the impact of AKI at different severity levels. We recommend that future prospective studies adopt standardized AKI staging reporting formats and document detailed time windows of AKI occurrence.

## Conclusions

AKI remains a common and important complication following abdominal surgery, with an overall incidence of 16%. This incidence varies significantly by surgical procedure but remains stable across different time periods and AKI diagnostic criteria. This study establishes a clear stage-dependent relationship between AKI severity and clinical outcomes, affecting both mortality risk and length of hospital stay. While individualized blood pressure management shows promise in reducing AKI risk, other perioperative interventions have not demonstrated significant protective effects. These findings emphasize the need for focused attention on blood pressure management within a broader, multimodal approach to AKI prevention, suggesting that future research should explore how to optimize and combine various preventive strategies.

## Supplementary Material

Supplemental Material

PRISMA 2020 checklist.docx

## Data Availability

The data that support the findings of this systematic review and meta-analysis are available from the corresponding author upon reasonable request. The data consist of the extracted information from published studies included in our analysis. All primary sources are cited within the manuscript and listed in the references.
