# Supplementary material for: Incidence and risk factors of acute kidney injury after abdominal surgery: a systematic review and meta-analysis
Source: Ann Med. 2025 Aug 17;57(1):2547324. doi: 10.1080/07853890.2025.2547324 (PMC12360056; doi:10.1080/07853890.2025.2547324)
Supplement: Supplemental Material [file IANN_A_2547324_SM9117.zip › Suppl_data/Figure Legends.docx]

Figure S1. Risk of bias assessments for randomized controlled studies

Figure S2. Meta-analysis of the incidence of AKI after abdominal surgery

Figure S3. Funnel plot with contour lines for publication bias assessment of observational studies examining risk factors for postoperative acute kidney injury

Figure S4. Forest plot of mortality risk by AKI stage in abdominal surgery patients

Figure S5. Forest plot of length of hospital stay by AKI stage in abdominal surgery patients
