# Supplementary material for: Incidence and risk factors of acute kidney injury after abdominal surgery: a systematic review and meta-analysis
Source: Ann Med. 2025 Aug 17;57(1):2547324. doi: 10.1080/07853890.2025.2547324 (PMC12360056; doi:10.1080/07853890.2025.2547324)
Supplement: Supplemental Material [file IANN_A_2547324_SM9117.zip › Suppl_data/Supplemental.docx]

**Supplemental**

Table S1. Baseline characteristics of studies that were excluded from our primary analysis

Table S2. Excluded studies from full-text review with reason(s) for exclusion

Table S3. Main characteristics of included studies (for citations see main manuscript)

Table S4. Risk of bias assessment using ROBINS-E tool for observational studies (For citations see main manuscript)

Table S5. Number of patients by stage of post-operative AKI (For citations see main manuscript)

Table S6. Hospital length of stay (days) of patients who developed post-operative AKI and those who did not (For citations see main manuscript)

Table S7. Mortality of patients who developed post-operative AKI and those who did not (For citations see main manuscript)

Table S8. Complications by AKI stage in abdominal surgery patients

Table S9. Factors associated with AKI after abdominal surgery (For citations see main manuscript)

Table S10 Characteristics and outcomes of Randomized Controlled Trials Evaluating perioperative hemodynamic management strategies

Table S1. Baseline characteristics of studies that were excluded from our primary analysis

| **Author** | **Year** | **Design** | **Operation** | **Age(year) Mean (SD) or median (IQR)** | **Male (%)** | **AKI definition** | **No. of patients** | **No. with AKI** | **Incidence of AKI** |
| --- | --- | --- | --- | --- | --- | --- | --- | --- | --- |
| Dali Zhang | 2021 | Retrospective cohort study | Orthotopic liver transplantation | 49.7（9.8） | 163（79.9） | KDIGO | 204 | 113 | 0.55 |
| Jaesik Park | 2021 | Retrospective cohort study | Living-donor liver transplantation | 53（48-59） | 400（69.3） | KDIGO | 577 | 143 | 0.25 |
| Kyoung-Sun Kim | 2021 | Retrospective cohort study | Liver transplantation | 53.1 (8.5) | 1789 (74.7) | KDIGO | 2395 | 1611 | 0.67 |
| Yihan Zhang | 2021 | Retrospective cohort study | Liver transplantation | 50.7 (10.6) | 682 (87.4) | KDIGO | 780 | 430 | 0.55 |
| Yingqi Chen | 2021 | Retrospective cohort study | Deceased donor liver transplantation | 49.3 (9.8) | 1234 (82) | KDIGO | 1505 | 671 | 0.45 |
| Banghe Bao | 2021 | Retrospective cohort study | Orthotopic liver transplantation | 48.6 (9.8) | 91 (91.9) | KDIGO | 99 | 29 | 0.29 |
| Jian Zhou | 2021 | Retrospective cohort study | Orthotopic liver transplantation | 54.1 (9.6) | 205 (84) | KDIGO | 244 | 163 | 0.67 |
| Simei Zhang | 2020 | Retrospective cohort study | Orthotopic liver transplantation | 47.3 (9.6) | 96 (72.7) | KDIGO | 132 | 66 | 0.50 |
| Francesca Tinti | 2020 | Retrospective cohort study | Liver transplantation | 57 (50-62) | 117 (80.7) | KDIGO | 145 | 83 | 0.57 |
| Tsung-Hsiao Shih | 2020 | Retrospective cohort study | Living donor liver transplantation | 53 (8.6) | NR | KDIGO | 60 | 25 | 0.42 |
| Jiang‑Chen Peng | 2020 | Retrospective cohort study | Liver transplantation | 50.3 (10.4) | 225 (80.4) | KDIGO | 280 | 89 | 0.32 |
| Ji Young Min | 2020 | Retrospective cohort study | Living donor liver transplantation | 51.5 (9.2) | 301 (71.2) | AKIN | 423 | 44 | 0.13 |
| Mengzhuo Guo | 2020 | Retrospective case-control study | Liver transplantation | 50.7 (11.2) | 97 (79.9) | KDIGO | 122 | 52 | 0.43 |
| Haiyang Xie | 2020 | Retrospective cohort study | Deceased donor liver transplantation | 48.7 (9.9) | 139 (88) | AKIN | 158 | 45 | 0.28 |
| Haijin Lv | 2020 | Retrospective cohort study | Orthotopic liver transplantation | 47.4 (10.3) | 156 (89.7) | KDIGO | 174 | 55 | 0.32 |
| Thorsten Feldkamp | 2020 | Retrospective cohort study | Liver transplantation | 48.7 (12.3) | 94 (63.1) | AKIN | 149 | 98 | 0.66 |
| François Martin Carrier | 2020 | Retrospective cohort study | Liver transplantation | 52 (11) | 358 (67.3) | KDIGO | 524 | 366 | 0.70 |
| Lingcan Tan | 2019 | Retrospective cohort study | Liver transplantation | 46.2 (9.7) | 184 (81.1) | KDIGO | 227 | 106 | 0.47 |
| Mingli Zhu | 2019 | Retrospective cohort study | Orthotopic liver transplantation | 50.4 (10.3) | 238 (79.1) | KDIGO | 301 | 94 | 0.31 |
| Je Hyuk Yu | 2019 | Retrospective cohort study | Liver transplantation | 53.8 (8.8) | 662 (74.8) | KDIGO | 885 | 342 | 0.39 |
| Won Ho Kim | 2019 | Retrospective cohort study | Living donor liver transplantation | 53 (48-58) | 559 (76.2) | KDIGO | 734 | 265 | 0.36 |
| Ao Jiao | 2019 | Retrospective cohort study | DCD liver transplantation | 50.4 (10) | 100 (75.8) | KDIGO | 132 | 37 | 0.28 |
| M. Giannella | 2019 | Prospective cohort study | Liver transplantation | 52.8 (10.7) | 394 (71.2) | RIFLE | 553 | 143 | 0.26 |
| Joanna Baron-Stefaniak | 2019 | Retrospective cohort study | Orthotopic liver transplantation | 57 (12) | 36 (76.6) | KDIGO | 47 | 36 | 0.77 |
| Yue Wang | 2017 | Retrospective cohort study | Orthotopic liver transplantation | 46.1 (10.1) | 111 (81) | KDIGO | 137 | 71 | 0.52 |
| Jeannette D. Widmer | 2018 | Retrospective cohort study | Liver transplantation | 55 (48-61) | NR | KDIGO | 378 | 56 | 0.15 |
| In-Gu Jun | 2018 | Retrospective cohort study | Living donor liver transplantation | 53 (8) | 1401 (75) | RIFLE | 1865 | 1270 | 0.68 |
| Marit Kalisvaart | 2018 | Retrospective cohort study | DCD liver transplantation | 58 (51-64) | 256 (70) | KDIGO | 368 | 239 | 0.65 |
| Raimundo Martins | 2018 | Cross-sectional study | Liver Transplantation | 53 (19-72) | 22 (55) | KDIGO | 40 | 34 | 0.85 |
| Gebhard Wagener | 2011 | Prospective cohort study | Orthotopic liver transplantation | 54.3 (11.6) | 60 (65.2) | RIFLE | 92 | 37 | 0.40 |
| Zhi-qiang Zhou | 2017 | Retrospective cohort study | Orthotopic liver transplantation | 48.1 (9.7) | 79 (76.7) | KDIGO | 103 | 42 | 0.41 |
| Seokha Yoo | 2016 | Retrospective cohort study | Liver transplantation | 47.8 (38.4) | 218 (71.7) | RIFLE | 304 | 132 | 0.43 |
| Palak J. Trivedi | 2017 | Retrospective cohort study | DCD liver transplantation | 49 (35-60) | 102 (71.3) | RIFLE | 143 | 76 | 0.43 |
| Emilie Trinh | 2016 | Retrospective cohort study | Liver transplantation | 57.2 (9.9) | 327 (66.6) | KDIGO | 491 | 278 | 0.57 |
| Kai Sun | 2016 | Retrospective cohort study | Liver transplantation | 56 (50-62) | 699 (67.4) | AKIN | 1037 | 569 | 0.55 |
| Mettu Srinivas Reddy | 2017 | RCT | Living donor liver transplantation | 48.5 (10.1) | 30 (73.1) | RIFLE | 41 | 18 | 0.44 |
| Ina Jochmans | 2017 | Prospective cohort study | Liver transplantation | 60 (50-67) | 48 (60) | RIFLE | 80 | 21 | 0.26 |
| Mohamed A. Kandil | 2017 | RCT | Living donor liver transplantation | 45.7 (7.8) | NR | AKIN | 50 | 23 | 0.46 |
| Xiaohong Chen | 2017 | Retrospective cohort study | Liver transplantation | 50.4 (9.6) | 505 (89.2) | AKIN | 566 | 109 | 0.19 |
| Min Suk Chae | 2017 | Retrospective cohort study | Living donor liver transplantation | 53 (48-59) | 230 (68.8) | AKIN | 334 | 76 | 0.23 |
| Weinberg L | 2016 | RCT | Liver transplantation | 50.3 (11.6) | 42 (70) | RIFLE | 60 | 21 | 0.35 |
| Ramona Nicolau-Raducu | 2016 | Retrospective cohort study | Liver transplantation | 55.9 (8) | 468 (66.1) | KDIGO | 708 | 278 | 0.39 |
| T. Mizota | 2016 | Retrospective cohort study | Living donor liver transplantation | 53.1 (11.3) | 166 (51.9) | KDIGO | 320 | 199 | 0.62 |
| In-Gu Jun | 2016 | Retrospective cohort study | Liver transplantation | 52.3 (8.3) | 1217 (75.3) | KDIGO | 1617 | 999 | 0.62 |
| H.A. Erdost | 2016 | Retrospective cohort study | Living donor liver transplantation | 46.7 (10.8) | 309 (70.2) | KDIGO | 440 | 64 | 0.15 |
| Jae Moon Choi | 2016 | Retrospective cohort study | Liver transplantation | 52 (8) | 264 (79.5) | AKIN | 332 | 73 | 0.22 |
| Viju Kumar Bharathan | 2016 | RCT | Living donor liver transplantation | 43.2 (12.1) | 88 (88.9) | AKIN | 99 | 28 | 0.28 |
| Bo-Hyun Sang | 2015 | Retrospective cohort study | Living donor liver transplantation | 51.8 (8) | 772 (77.4) | AKIN | 998 | 593 | 0.59 |
| Mi Hye Park | 2015 | Retrospective cohort study | Living donor liver transplantation | 53.4 (7.1) | 530 (79.9) | RIFLE | 538 | 147 | 0.27 |
| Ahmed Mukhtar | 2015 | Retrospective case-controlled study | Living donor liver transplantation | 50.2 (19.8) | 265 (87.5) | AKIN | 303 | 115 | 0.38 |
| I. A. Hilmi | 2015 | Retrospective cohort study | Orthotopic liver transplantation | 56.7 (9.5) | 286 (67.4) | KDIGO | 424 | 221 | 0.52 |
| Adller G. C. Barreto | 2015 | Retrospective cohort study | Liver transplantation | 56 (48-62) | 60 (50) | AKIN | 134 | 64 | 0.49 |
| S. Papadopoulos | 2014 | Retrospective cohort study | Orthotopic liver transplantation | 51.8 (10.4) | 53 (74.6) | AKIN | 71 | 37 | 0.52 |
| P. Marcelino | 2014 | Prospective cohort study | Liver Transplantation | 49.3 (11.5) | 38 (62.3) | AKIN | 61 | 19 | 0.31 |
| Won Ho Kim | 2014 | RCT | Living donor liver transplantation | 52 (9.6) | 55 (70.5) | RIFLE | 78 | 43 | 0.55 |
| A. Karapanagiotou | 2014 | Retrospective cohort study | Liver transplantation | 51.8 (10.4) | 53 (74.6) | AKIN | 71 | 37 | 0.52 |
| Masashi Utsumi | 2013 | Retrospective cohort study | Living donor liver transplantation | 49.3 (2) | 115 (57.5) | RIFLE | 200 | 121 | 0.61 |
| Jeffrey C Sirota | 2013 | Retrospective cohort study | Liver transplantation | 56.2 (6.8) | 27 (67.5) | RIFLE | 40 | 7 | 0.18 |
| Roberto Camargo Narciso | 2013 | Retrospective cohort study | Liver transplantation | 53 (46-60) | 207 (66) | AKIN | 315 | 255 | 0.81 |
| Zhen-Yong Shao | 2013 | Retrospective cohort study | Liver transplantation | 44 (20-69) | 478 (83.6) | RIFLE | 572 | 85 | 0.15 |
| J. A. Leithead | 2011 | Retrospective cohort study | DCD liver transplantation | 55.9 (8.8) | 104 (59.1) | RIFLE | 176 | 75 | 0.43 |
| I. Umbro | 2011 | Observational study | Liver transplantation | 55.1 (10.2) | 34 (74) | RIFLE | 46 | 26 | 0.57 |
| F. Tinti | 2011 | Retrospective cohort study | Liver transplantation | 55.5 (9.9) | 33 (75) | RIFLE | 44 | 24 | 0.55 |
| E. Biagioni | 2011 | Retrospective cohort study | Orthotopic liver transplantation | 56 (9) | NR | RIFLE | 84 | 24 | 0.29 |
| M. Zhu | 2010 | Retrospective cohort study | Liver transplantation | 48.1 (10) | 154 (79.8) | AKIN | 193 | 116 | 0.60 |
| Andrew J. Portal | 2010 | observation study | Liver transplantation | 50 (38-57) | 56 (58.9) | AKIN | 95 | 30 | 0.32 |
| Jung Pyo Lee | 2010 | Retrospective cohort study | Liver transplantation | 48.3 (9.2) | 312 (72.4) | RIFLE | 431 | 118 | 0.27 |
| A. Kundakci | 2010 | Retrospective cohort study | Liver transplantation | 43.9 (13.4) | 85 (75.9) | RIFLE | 112 | 64 | 0.57 |
| Jose I Iglesias | 2010 | Retrospective cohort study | Orthotopic liver transplantation | 49.2 (10.9) | 392 (57) | AKIN | 688 | 243 | 0.35 |
| Ibtesam A. Hilmi | 2010 | RCT | Orthotopic liver transplantation | 59.5 (9) | 69 (69) | RIFLE | 100 | 34 | 0.34 |
| Ferreira AC | 2010 | Retrospective cohort study | Orthotopic liver transplantation | 44 (12.6) | 453 (64) | RIFLE | 708 | 235 | 0.33 |
| JB Cabezuelo | 2006 | Retrospective cohort study | Liver transplantation | 46 (13) | 142 (77.2) | RIFLE | 184 | 57 | 0.31 |
| Joanna A. Leithead | 2015 | Retrospective cohort study | Split liver transplantation | 52.7 (11) | 197 (65.5) | KDIGO | 301 | 168 | 0.56 |
| Y. Sirivatanauksorn | 2014 | Retrospective cohort study | Orthotopic liver transplantation | 52.5 (22-71) | 56 (69) | RIFLE | 81 | 58 | 0.72 |
| Thiago Gomes Romano | 2014 | Retrospective cohort study | Liver transplantation | 52 (41-59) | 61 (66.6) | AKIN | 92 | 52 | 0.57 |
| Jeong T-D | 2012 | Retrospective cohort study | Liver transplantation | 52 (74) | 15 (78.9) | RIFLE | 19 | 11 | 0.58 |
| Mitra K. Nadim | 2012 | Retrospective cohort study | Liver transplantation | 49.7 (36.9) | 180 (63.6) | RIFLE | 283 | 118 | 0.42 |
| Zhu Mingli | 2009 | Observational study | Orthotopic liver transplantation | 48.1 (10.0) | 154 (79.8) | AKIN | 193 | 116 | 0.60 |
| Dong Zhouzhou | 2018 | Observational study | DCD liver transplantation | 48.2 (9.5) | 84 (63.6) | KDIGO | 132 | 77 | 0.58 |
| Yueh-Tse Wua | 2024 | Prospective observational study | Liver transplantation | 54.9 (10.3) | 53 (75.7) | KDIGO | 70 | 26 | 0.37 |
| Maxime Soucy-Proulx | 2024 | Retrospective study | Liver transplantation | 54 (14) | 342 (73) | KDIGO | 467 | 211 | 0.45 |
| Camila Lima | 2024 | Retrospective study | Liver transplantation | 58 (12) | 64 (64) | KDIGO | 100 | 36 | 0.36 |
| Hye-Mee Kwon | 2024 | randomized controlled trial | Liver transplantation | 56 (51–60) | 150 (73.2) | KDIGO | 205 | 84 | 0.42 |
| Ji-Yoon Jung | 2024 | Retrospective study | Liver transplantation | 52 (49-63) | 641 (74.4) | KDIGO | 861 | 364 | 0.42 |
| Hye-Won Jeong | 2024 | Retrospective study | Liver transplantation | 55 (50–60) | 2193 (73.8) | KDIGO | 1721 | 996 | 0.58 |
| Benedikt Hilger | 2024 | Retrospective cohort study | Liver transplantation | 59 (48–71) | 129 (58.1) | KDIGO | 178 | 153 | 0.86 |
| Benjamin N. Herrmann | 2024 | Retrospective cohort study | Liver transplantation | 59 (49-65) | 138 (59.7) | KDIGO | 231 | 102 | 0.45 |
| Jacek B. Cywinski | 2024 | Retrospective study | Liver transplantation | 56 (11) | 1054 (67) | KDIGO | 1576 | 1160 | 0.74 |
| Matthanja Bieze | 2024 | Retrospective cohort study | Liver transplantation | 58.6 (49.9‒64.5) | 754 (65.4) | KDIGO | 1153 | 544 | 0.47 |
| Matteo Rinaldi | 2023 | Retrospective study | Liver transplantation | 55 (46–62) | 157 (62.8) | KDIGO | 250 | 175 | 0.7 |
| Åsa Norén | 2023 | Retrospective study | Liver transplantation | 63 (54-66) | NA | KDIGO | 30 | 23 | 0.77 |
| Ji‑Yoon Jung | 2023 | Retrospective study | Liver transplantation | 50 (49-60) | 1120 (71.6) | KDIGO | 1565 | 664 | 0.42 |
| Rebecca Caragata | 2023 | Retrospective cohort study | Liver transplantation | 58.0 (50-62) | 874 (67.6) | KDIGO | 1292 | 519 | 0.4 |
| Ignacio Sáez de la Fuente | 2022 | Retrospective study | Liver transplantation | 55.6 (51-63) | 116 (77.3) | KDIGO | 150 | 88 | 0.59 |
| Ana Paula Camargos de Figueirêdo Neves | 2022 | Retrospective study | Liver transplantation | 54 (43-65) | 30 (61.2) | KDIGO | 49 | 41 | 0.84 |
| Moataz Maher Emara | 2022 | Prospective cohort study | Liver transplantation | 53 (43-57) | 56 (70) | KDIGO | 80 | 20 | 0.25 |
| Dewitte, Antoine | 2022 | Retrospective study | Liver transplantation | 58 (52-63) | 335 (76.3) | KDIGO | 439 | 144 | 0.33 |
| Michael S Bleszynski | 2022 | Retrospective study | Liver transplantation | 57 (49–62) | 206 (62.2) | AKIN | 332 | 147 | 0.44 |
| Wang Xin | 2021 | Retrospective study | Liver transplantation | 53 (9) | 91 (83.4) | KDIGO | 109 | 53 | 0.49 |
| Dandan Guo | 2021 | Retrospective study | Liver transplantation | 52 (45–59) | 472 (81.9) | KDIGO | 576 | 207 | 0.36 |
| Lia Cavalcante Cezar | 2021 | Prospective study | Liver transplantation | 54 (12) | 28 (60) | KDIGO | 46 | 24 | 0.52 |
| Tomoaki Hirose | 2020 | Retrospective cohort study | OAAA | 71.6 (7.4) | 254 (87.6) | KDIGO | 290 | 39 | 0.13 |
| Athanasios Saratzis | 2019 | Prospective cohort study | OAAA | 71 (4.6) | 113 (91.1) | KDIGO | 124 | 48 | 0.39 |
| Xavier Chaufour | 2020 | Retrospective cohort study | OAAA | 68 (8) | 292 (92.6) | RIFLE | 315 | 95 | 0.30 |
| Martin H. Bernard | 2019 | Retrospective cohort study | OAAA | Not stated by subgroup | Not stated by subgroup | RIFLE | 678 | 125 | 0.18 |
| Liesa Zabrocki | 2017 | Retrospective cohort study | OAAA | 72 (7) | 79 (86.8) | KDIGO | 91 | 38 | 0.42 |
| Paolo Lentini | 2018 | Prospective cohort study | OAAA | 72.9 (6) | 36 (70.6) | KDIGO | 51 | 23 | 0.45 |
| Philippe Guerci | 2018 | Prospective cohort study | OAAA | 65.9 (9.1) | 47 (94) | KDIGO | 50 | 18 | 0.36 |
| Emmanuelle Duceppe | 2018 | Retrospective cohort study | OAAA | Not stated by subgroup | Not stated by subgroup | AKIN | 216 | 29 | 0.13 |
| Ying Tang | 2017 | Retrospective cohort study | OAAA | 66.5  (59.3-73) | 45 (80.4) | KDIGO | 56 | 24 | 0.43 |
| Andrzej Siemiatkowski | 2017 | Retrospective cohort study | OAAA | 70 (51-87) | 140 (81.9) | KDIGO | 171 | 62 | 0.36 |
| Sytse C. van Beek | 2014 | Retrospective cohort study | OAAA | Not stated by subgroup | Not stated by subgroup | RIFLE | 294 | 224 | 0.76 |
| Noelle Murphy | 2014 | RCT | OAAA | 72.1 (9) | 53 (85.5) | AKIN | 62 | 28 | 0.45 |
| J.-Y. Bang | 2014 | Retrospective cohort study | OAAA | Not stated by subgroup | Not stated by subgroup | AKIN | 254 | 57 | 0.22 |
| M. Tallgren | 2007 | RCT | OAAA | 67.1 (11) | 54 (78.3) | RIFLE | 69 | 15 | 0.22 |
| Yue Jia-ning | 2013 | Retrospective cohort study | OAAA | 63 (12.4) | 37 (72.5) | RIFLE | 51 | 21 | 0.41 |
| Konstantinos M. Pirgakis | 2014 | Prospective cohort study | OAAA | 65.6 (9) | 38 (97.4) | RIFLE | 39 | 13 | 0.33 |
| Aurelien Hostalrich | 2025 | Retrospective study | OAAA | 65 (60-68) | 16 (42.1) | RIFLE | 38 | 11 | 28.9 |
| Natchanikant Tepkit | 2024 | Retrospective study | OAAA | 75.12 (8.5) | 148 (75.5) | KDIGO | 69 | 21 | 0.31 |
| Martin Sigl M | 2024 | Prospective observational study | OAAA | 68 (7.2) | 54 (70) | AKIN | 77 | 6 | 0.08 |
| Petar Zlatanovic | 2023 | Retrospective cohort study | OAAA | 71.3 (6.8) | 125 (86.2) | RIFLE | 145 | 59 | 0.40 |
| Hei Jin Yoon | 2023 | Retrospective study | OAAA | 72.4 (9.2) | 122 (85.3) | KDIGO | 143 | 60 | 0.42 |

Abbreviations: DCD, Donation after Cardiac Death; IQR, interquartile range; NR, not reported; RCT, randomized controlled trial; SD, Standard Deviation; OAAA, open abdominal aortic aneurysm.

Table S2. Excluded studies from full-text review with reason(s) for exclusion

| Author | Year | Title of Study | Reason for exclusion |
| --- | --- | --- | --- |
| Ashish K. Khanna^1^ | 2021 | Postoperative Hypotension and Adverse Clinical Outcomes in Patients Without Intraoperative Hypotension, After Noncardiac Surgery | Data on AKI not available for the abdominal surgery |
| Stephen S Johnston^2^ | 2021 | Association of In-Hospital Surgical Bleeding Events with Prolonged Hospital Length of Stay, Days Spent in Critical Care, Complications, and Mortality: A Retrospective Cohort Study Among Patients Undergoing Neoplasm-Directed Surgeries in English Hospitals | Data on AKI not available for the abdominal surgery and did not use a consensus definition of AKI |
| Yousaf B. Hadi^3^ | 2021 | Outcomes of COVID-19 in Solid Organ Transplant Recipients: A Propensity-matched Analysis of a Large Research Network | Data on AKI not available for the abdominal surgery and did not use a consensus definition of AKI |
| Dominique Engel^4^ | 2021 | Preoperative Concentrated Urine Increases the Incidence of Plasma Creatinine Elevation After Major Surgery | The surgical procedure of the study subjects was urological surgery |
| Jacqueline Del Carpio^5^ | 2021 | Development and validation of a model to predict severe hospital‐acquired acute kidney injury in non‐critically ill patients | Data on AKI not available for the abdominal surgery |
| Ji-Yeon Bang^6^ | 2021 | Impact of Sarcopenia on Acute Kidney Injury after Infrarenal Abdominal Aortic Aneurysm Surgery: A Propensity Matching Analysis | Data on AKI not available for the open surgical treatment |
| Meagan Alvarado^7^ | 2021 | Black Race Is Associated With Higher Rates of Early-Onset End-Stage Renal Disease and Increased Mortality Following Liver Transplantation | No data on postoperative AKI in patients who had undergone liver transplant |
| Ravi Bhatia^8^ | 2021 | Association between fast-track extubation after orthotopic liver transplant, postoperative vasopressor requirement, and acute kidney injury | No data on postoperative AKI in patients who had undergone liver transplant |
| Natasha Abeysekera^9^ | 2021 | Evaluating the need for an integrated geriatric service in older general surgery patients | Data on AKI not available for the abdominal surgery |
| Nuttawut Vongsumran^10^ | 2020 | Standardized glycemic management versus conventional glycemic management and postoperative outcomes in type 2 diabetes patients undergoing elective surgery | Data on AKI not available for the abdominal surgery |
| S. J. van Ommeren-Olijve^11^ | 2020 | Risk factors for non-closure of an intended temporary defunctioning stoma after emergency resection of left-sided obstructive colon cancer | Data on AKI not available for the abdominal surgery |
| K. Ruetzler^12^ | 2020 | Supplemental Intraoperative Oxygen Does Not Promote Acute Kidney Injury or Cardiovascular Complications After Noncardiac Surgery: Subanalysis of an Alternating Intervention Trial | Data on AKI not available for the abdominal surgery |
| S. Park^13^ | 2020 | Intraoperative Arterial Pressure Variability and Postoperative Acute Kidney Injury | Data on AKI not available for the abdominal surgery |
| J. Y. Nicklas^14^ | 2020 | Personalised haemodynamic management targeting baseline cardiac index in high-risk patients undergoing major abdominal surgery: a randomised single-centre clinical trial | Data on AKI not available for the abdominal surgery |
| Morgan Gruner^15^ | - | Role of Blood Management in Optimizing Perioperative Outcomes in Patients With Secondary Anemia Undergoing Hysterectomy or Myomectomy for Abnormal Uterine Bleeding: A Proposed Randomized Control Trial | Data on AKI not available for the abdominal surgery |
| Tae ho Hong^16^ | - | Necessity of Preoperative Empirical Antibiotic Use in Mild to Moderate Acute Inflammatory Gallbladder Disease; a Randomized Controlled Trial. | Data on AKI not available for the abdominal surgery |
| Tamer Alsaied Alnaimy^17^ | - | Laparoscopic Cholecystectomy is no More Risky in Emergent Cases With in Cardiopulmonary Risk: Fundus-Callot Cholecystectomy With Low Pressure Pneumo-peritoneum VS Open Cholecystectomy - Randomized Controlled Trials | Data on AKI not available for the abdominal surgery |
| K. Miyake^18^ | 2020 | Association of pre-operative chronic kidney disease and acute kidney injury with in-hospital outcomes of emergency colorectal surgery: a cohort study | Data on postoperative AKI not available for the abdominal surgery |
| M. R. Mathis^19^ | 2020 | Preoperative Risk and the Association between Hypotension and Postoperative Acute Kidney Injury | Data on postoperative AKI not available for the abdominal surgery |
| K. Maheshwari^20^ | 2020 | Saline versus Lactated Ringer's Solution: The Saline or Lactated Ringer's (SOLAR) Trial | Data on postoperative AKI not available for the abdominal surgery |
| N. Lysak^21^ | 2020 | Cardiovascular death and progression to end-stage renal disease after major surgery in elderly patients | Data on postoperative AKI not available for the abdominal surgery |
| N. E. Ingraham^22^ | 2020 | Pre- and Peri-Operative Factors Associated with Chronic Critical Illness in Liver Transplant Recipients | Did not use a consensus definition of AKI |
| S. Andrianello^23^ | 2020 | Pancreaticojejunostomy With Externalized Stent vs Pancreaticogastrostomy With Externalized Stent for Patients With High-Risk Pancreatic Anastomosis: a Single-Center, Phase 3, Randomized Clinical Trial | Data on postoperative AKI not available for the abdominal surgery |
| S. Yang^24^ | 2019 | Clinical features and outcomes of patients with acute mesenteric ischemia and concomitant colon ischemia: a retrospective cohort study | Data on postoperative AKI not available for the abdominal surgery |
| Y. Tang^25^ | 2019 | Association of Intraoperative Hypotension with Acute Kidney Injury after Noncardiac Surgery in Patients Younger than 60 Years Old | Data on postoperative AKI not available for the abdominal surgery |
| M. Szabó^26^ | 2019 | Role of inferior vena cava collapsibility index in the prediction of hypotension associated with general anesthesia: an observational study | Data on postoperative AKI not available for the abdominal surgery |
| S. Park^27^ | 2019 | Simple Postoperative AKI Risk (SPARK) Classification before Noncardiac Surgery: A Prediction Index Development Study with External Validation | Data on postoperative AKI not available for the abdominal surgery |
| T. K. Oh^28^ | 2019 | Admission to the surgical intensive care unit during intensivist coverage is associated with lower incidence of postoperative acute kidney injury and shorter ventilator time | Data on postoperative AKI not available for the abdominal surgery |
| T. K. Oh^29^ | 2019 | Association of perioperative weight-based fluid balance with 30-day mortality and acute kidney injury among patients in the surgical intensive care unit | Data on postoperative AKI not available for the abdominal surgery |
| T. K. Oh^30^ | 2019 | Association of Preoperative Serum Chloride Levels With Mortality and Morbidity After Noncardiac Surgery: A Retrospective Cohort Study | Data on postoperative AKI not available for the abdominal surgery |
| N. MacDonald^31^ | 2019 | The role of goal-directed therapy in the prevention of acute kidney injury after major gastrointestinal surgery: Substudy of the OPTIMISE trial | Data on postoperative AKI not available for the abdominal surgery |
| V. J. Lei^32^ | 2019 | Risk Stratification for Postoperative Acute Kidney Injury in Major Noncardiac Surgery Using Preoperative and Intraoperative Data | Data on postoperative AKI not available for the abdominal surgery |
| P. Kougias^33^ | 2019 | Effect of Postoperative Permissive Anemia and Cardiovascular Risk Status on Outcomes After Major General and Vascular Surgery Operative Interventions | Data on postoperative AKI not available for the abdominal surgery |
| B. J. Kim^34^ | 2019 | Contemporary analysis of complications associated with biliary stents during neoadjuvant therapy for pancreatic adenocarcinoma | Data on postoperative AKI not available for the abdominal surgery |
| K. C. Hung^35^ | 2019 | Impact of Preoperative Anemia on Postoperative Kidney Function Following Laparoscopic Bariatric Surgery | Data on postoperative AKI not available for the abdominal surgery |
| L. Adhikari^36^ | 2019 | Improved predictive models for acute kidney injury with IDEA: Intraoperative Data Embedded Analytics | Data on postoperative AKI not available for the abdominal surgery |
| I. Zealley^37^ | 2018 | Exposure to contrast media in the perioperative period confers no additional risk of acute kidney injury in surgical patients | Data on postoperative AKI not available for the abdominal surgery |
| L. Weinberg^38^ | 2018 | Associations of fluid amount, type, and balance and acute kidney injury in patients undergoing major surgery | Data on postoperative AKI not available for the abdominal surgery |
| A. Shiba^39^ | 2018 | Association Between Intraoperative Oliguria and Acute Kidney Injury After Major Noncardiac Surgery | Data on postoperative AKI not available for the abdominal surgery |
| S. Sakan^40^ | 2018 | Consequence of Elevated Fibroblast Growth Factor 23 Levels in Acute Kidney Injury, Renal Recovery and Overall Survival in Intensive Care Unit Patients After Major Surgery | Data on postoperative AKI not available for the abdominal surgery |
| S. Park^41^ | 2018 | Awareness, incidence and clinical significance of acute kidney injury after non-general anesthesia: A retrospective cohort study | Data on postoperative AKI not available for the abdominal surgery |
| T. K. Oh^42^ | 2018 | Hyperchloremia and postoperative acute kidney injury: a retrospective analysis of data from the surgical intensive care unit | Data on postoperative AKI not available for the abdominal surgery |
| Nana Xu^43^ | 2018 | Association between preoperative renin-angiotensin system inhibitor use and postoperative acute kidney injury risk in patients with hypertension | Data on postoperative AKI not available for the abdominal surgery |
| Z. Mzoughi^44^ | 2018 | Laparoscopy for perforated duodenal ulcer: A morbidity score based on a cohort study of 384 patients | Data on postoperative AKI not available for the abdominal surgery |
| H. M. Jia^45^ | 2018 | Derivation and validation of plasma endostatin for predicting renal recovery from acute kidney injury: a prospective validation study | Data on postoperative AKI not available for the abdominal surgery |
| B. D. Hinck^46^ | 2018 | Urine kidney injury markers do not increase following gastric bypass: a multi-center cross-sectional study | Data on postoperative AKI not available for the abdominal surgery |
| T. Dagel^47^ | 2018 | Hyperthermic intraperitonal chemotherapy is an independent risk factor for development of acute kidney injury | Data on postoperative AKI not available for the abdominal surgery |
| A. K. Bressan^48^ | 2018 | Efficacy of a Dual-ring Wound Protector for Prevention of Surgical Site Infections After Pancreaticoduodenectomy in Patients With Intrabiliary Stents: a Randomized Clinical Trial | Data on postoperative AKI not available for the abdominal surgery |
| X. J. Zhao^49^ | 2017 | Acute kidney injury is an independent risk factor for myocardial injury after noncardiac surgery in critical patients | Data on postoperative AKI not available for the abdominal surgery |
| Y. L. Sun^50^ | 2017 | Total closure of pancreatic section for end-to-side pancreaticojejunostomy decreases incidence of pancreatic fistula in pancreaticoduodenectomy | Data on postoperative AKI not available for the abdominal surgery |
| S. Spadaro^51^ | 2017 | The effects of storage of red blood cells on the development of postoperative infections after noncardiac surgery | Data on postoperative AKI not available for the abdominal surgery |
| A. Rencuzogullari^52^ | 2017 | Nomogram-derived prediction of postoperative ileus after colectomy: An assessment from nationwide procedure-targeted cohort | Data on postoperative AKI not available for the abdominal surgery |
| J. R. Puckett^53^ | 2017 | Low Versus Standard Urine Output Targets in Patients Undergoing Major Abdominal Surgery: A Randomized Noninferiority Trial | Did not use a consensus definition of AKI |
| H. Ç. Özcan^54^ | 2017 | Emergency peripartum hysterectomy: single center ten-year experience | Data on postoperative AKI not available for the abdominal surgery |
| O. A. Olufajo^55^ | 2017 | Preoperative assessment of surgical risk: creation of a scoring tool to estimate 1-year mortality after emergency abdominal surgery in the elderly patient | Data on postoperative AKI not available for the abdominal surgery |
| P. Ferrada^56^ | 2017 | Loop ileostomy versus total colectomy as surgical treatment for Clostridium difficile-associated disease: An Eastern Association for the Surgery of Trauma multicenter trial | Data on postoperative AKI not available for the abdominal surgery |
| W. Branch-Elliman^57^ | 2017 | Risk of surgical site infection, acute kidney injury, and Clostridium difficile infection following antibiotic prophylaxis with vancomycin plus a beta-lactam versus either drug alone: A national propensity-score-adjusted retrospective cohort study | Data on postoperative AKI not available for the abdominal surgery |
| R. Behman^58^ | 2017 | Laparoscopic Surgery for Adhesive Small Bowel Obstruction Is Associated with a Higher Risk of Bowel Injury: A Population-based Analysis of 8584 Patients | Data on postoperative AKI not available for the abdominal surgery |
| A. M. Zimmerman^59^ | 2016 | Preoperative Myocardial Injury as a Predictor of Mortality in Emergency General Surgery: An Analysis Using the American College of Surgeons NSQIP Database | Data on postoperative AKI not available for the abdominal surgery |
| A. Vijay^60^ | 2016 | Perioperative management of patients treated with angiotensin-converting enzyme inhibitors and angiotensin II receptor blockers: a quality improvement audit | Data on postoperative AKI not available for the abdominal surgery |
| P. Thottakkara^61^ | 2016 | Application of Machine Learning Techniques to High-Dimensional Clinical Data to Forecast Postoperative Complications | Data on postoperative AKI not available for the abdominal surgery |
| C. Pisitsak^62^ | 2016 | Prevalence, Outcomes and Risk factors of Acute Kidney Injury in Surgical Intensive Care Unit: A Multi-Center Thai University-Based Surgical Intensive Care Units Study (THAI-SICU Study) | Research object duplication^63^ |
| A. D. Oprea^64^ | 2016 | Baseline Pulse Pressure, Acute Kidney Injury, and Mortality After Noncardiac Surgery | Data on postoperative AKI not available for the abdominal surgery |
| X. Liu^65^ | 2016 | A Predictive Model for Assessing Surgery-Related Acute Kidney Injury Risk in Hypertensive Patients: A Retrospective Cohort Study | Data on postoperative AKI not available for the abdominal surgery |
| D. Korenkevych^66^ | 2016 | The Pattern of Longitudinal Change in Serum Creatinine and 90-Day Mortality After Major Surgery | Data on postoperative AKI not available for the abdominal surgery |
| M. Huber^67^ | 2016 | Mortality and Cost of Acute and Chronic Kidney Disease after Vascular Surgery | Data on postoperative AKI not available for the open abdominal aortic surgery |
| F. Cauchy^68^ | 2016 | Incidence, risk factors and consequences of bile leakage following laparoscopic major hepatectomy | Data on postoperative AKI not available for the abdominal surgery |
| C. C. Chang^69^ | 2016 | Impact of Peri-Operative Anemia and Blood Transfusions in Patients with Gastric Cancer Receiving Gastrectomy | Data on postoperative AKI not available for the abdominal surgery |
| D. Bushyhead^70^ | 2016 | Pretransplant echocardiographic parameters as markers of posttransplant outcomes in liver transplant recipients | Data on postoperative AKI not available for the liver transplant surgery |
| A. Bardia^71^ | 2016 | Combined Epidural-General Anesthesia vs General Anesthesia Alone for Elective Abdominal Aortic Aneurysm Repair | Data on postoperative AKI not available for the open abdominal aortic surgery |
| H. M. Barakat^72^ | 2016 | Preoperative supervised exercise improves outcomes after elective abdominal aortic aneurysm repair | Data on AKI not available for the open abdominal aortic surgery and did not use a consensus definition of AKI |
| A. Andert^73^ | 2016 | Liver Transplantation and Donor Body Mass Index >30: Use or Refuse? | Did not use a consensus definition of AKI |
| K. B. Wise^74^ | 2015 | Proximal intestinal diversion is associated with increased morbidity in patients undergoing elective colectomy for diverticular disease: an ACS-NSQIP study | Data on AKI not available for the abdominal surgery and did not use a consensus definition of AKI |
| A. Weick^75^ | 2015 | Incidence of cardiovascular and cerebrovascular events associated with sirolimus use after liver transplantation | Data on AKI not available for the liver transplant surgery and did not use a consensus definition of AKI |
| Z. Saze^76^ | 2015 | Risk Models of Operative Morbidities in 16,930 Critically Ill Surgical Patients Based on a Japanese Nationwide Database | Did not use a consensus definition of AKI |
| Z. Moghadamyeghaneh^77^ | 2015 | Effects of ascites on outcomes of colorectal surgery in congestive heart failure patients | Did not use a consensus definition of AKI |
| T. E. Newhook^78^ | 2015 | Impact of Postoperative Venous Thromboembolism on Postoperative Morbidity, Mortality, and Resource Utilization after Hepatectomy | Did not use a consensus definition of AKI |
| J. C. Longenecker^79^ | 2015 | Patterns of Kidney Function Before and After Orthotopic Liver Transplant: Associations With Length of Hospital Stay, Progression to End-Stage Renal Disease, and Mortality | Data on AKI not available for the abdominal surgery and did not use a consensus definition of AKI |
| K. Kashani^80^ | 2015 | Vascular Surgery Kidney Injury Predictive Score: A Historical Cohort Study | Data on AKI not available for the open abdominal aortic surgery |
| W. Huang^81^ | 2015 | Value of Neutrophil Counts in Predicting Surgery-Related Acute Kidney Injury and the Interaction of These Counts With Diabetes in Chronic Kidney Disease Patients With Hypertension: A Cohort Study | Data on postoperative AKI not available for the abdominal surgery |
| C. Hobson^82^ | 2015 | Cost and Mortality Associated With Postoperative Acute Kidney Injury | Data on postoperative AKI not available for the abdominal surgery |
| I. Gocze^83^ | 2015 | Urinary biomarkers TIMP-2 and IGFBP7 early predict acute kidney injury after major surgery | Only 12 patients in includable subgroup with hepatobiliary surgery |
| M. Giannella^84^ | 2015 | High-dose weekly liposomal amphotericin b antifungal prophylaxis in patients undergoing liver transplantation: a prospective phase II trial | Did not use a consensus definition of AKI |
| C. L. Chou^85^ | 2015 | Adverse outcomes after major surgery in patients with pressure ulcer: a nationwide population-based retrospective cohort study | Did not use a consensus definition of AKI |
| H. P. Chen^86^ | 2015 | Incidence and Outcomes of Acute Renal Failure Following Liver Transplantation: A Population-Based Cohort Study | Did not use a consensus definition of AKI |
| M. S. Tsai^87^ | 2014 | Diabetes mellitus and increased postoperative risk of acute renal failure after hepatectomy for hepatocellular carcinoma: a nationwide population-based study | Did not use a consensus definition of AKI |
| G. Spolverato^88^ | 2014 | Failure to rescue as a source of variation in hospital mortality after hepatic surgery | Did not use a consensus definition of AKI |
| M. Shah^89^ | 2014 | Association between angiotensin converting enzyme inhibitor or angiotensin receptor blocker use prior to major elective surgery and the risk of acute dialysis | Data on AKI not available for the abdominal surgery and did not use a consensus definition of AKI |
| R. L. Ruebner^90^ | 2014 | Donation after cardiac death liver transplantation is associated with increased risk of end-stage renal disease | Data on postoperative AKI not available for the liver transplant surgery |
| X. Pan^91^ | 2014 | Perioperative complications in liver transplantation using donation after cardiac death grafts: a propensity-matched study | Data on postoperative AKI not available for the liver transplant surgery |
| J. C. Padussis^92^ | 2014 | Feeding jejunostomy during Whipple is associated with increased morbidity | Did not use a consensus definition of AKI |
| Z. Moghadamyeghaneh^93^ | 2014 | Preoperative dehydration increases risk of postoperative acute renal failure in colon and rectal surgery | Did not use a consensus definition of AKI |
| H. Mahdi^94^ | 2014 | Surgical site infection in women undergoing surgery for gynecologic cancer | Did not use a consensus definition of AKI |
| J. A. Lin^95^ | 2014 | Adverse outcomes after major surgery in patients with systemic lupus erythematosus: a nationwide population-based study | Did not use a consensus definition of AKI |
| N. Lekerika^96^ | 2014 | Predicting fluid responsiveness in patients undergoing orthotopic liver transplantation: effects on intraoperative blood transfusion and postoperative complications | Did not use a consensus definition of AKI |
| H. Kobayashi^97^ | 2014 | Risk model for right hemicolectomy based on 19,070 Japanese patients in the National Clinical Database | Did not use a consensus definition of AKI |
| B. K. Kashy^98^ | 2014 | Effect of hydroxyethyl starch on postoperative kidney function in patients having noncardiac surgery | Data on AKI not available for the abdominal surgery |
| H. Iwata^99^ | 2014 | Negative prognostic impact of renal replacement therapy in adult living-donor liver transplant recipients: preoperative recipient condition and donor factors | Data on AKI not available for the abdominal surgery |
| D. G. Harris^100^ | 2014 | Recurrent kidney injury in critically ill surgical patients is common and associated with worse outcomes | Data on AKI not available for the abdominal surgery |
| W. J. Halabi^101^ | 2014 | Surgery for gallstone ileus: a nationwide comparison of trends and outcomes | Did not use a consensus definition of AKI |
| A. X. Garg^102^ | 2014 | Perioperative aspirin and clonidine and risk of acute kidney injury: a randomized clinical trial | Data on AKI not available for the abdominal surgery |
| A. X. Garg^103^ | 2014 | Aspirin and clonidine in non-cardiac surgery: acute kidney injury substudy protocol of the Perioperative Ischaemic Evaluation (POISE) 2 randomised controlled trial | Data on AKI not available for the abdominal surgery and research object duplication^102^ |
| A. Fernandes^104^ | 2014 | General anesthesia type does not influence serum levels of neutrophil gelatinase-associated lipocalin during the perioperative period in video laparoscopic bariatric surgery | Data on AKI not available for the abdominal surgery and did not use a consensus definition of AKI |
| J. F. Bosset^105^ | 2014 | Fluorouracil-based adjuvant chemotherapy after preoperative chemoradiotherapy in rectal cancer: long-term results of the EORTC 22921 randomised study | Data on AKI not available for the abdominal surgery |
| T. Yang^106^ | 2014 | Risk factors of hospital mortality after re-laparotomy for post-hepatectomy hemorrhage | Did not use a consensus definition of AKI |
| U. Wenger^107^ | 2013 | The relationship between preoperative creatinine clearance and outcomes for patients undergoing liver transplantation: a retrospective observational study | Data on AKI not available for the abdominal surgery and did not use a consensus definition of AKI |
| M. Walsh^108^ | 2013 | The association between perioperative hemoglobin and acute kidney injury in patients having noncardiac surgery | Data on AKI not available for the abdominal surgery |
| P. Sharma^109^ | 2013 | Patient-specific prediction of ESRD after liver transplantation | Data on AKI not available for the liver transplantation surgery |
| I. Kopolovic^110^ | 2013 | Risk factors and outcomes associated with acute kidney injury following ruptured abdominal aortic aneurysm | Data on postoperative AKI not available for the open abdominal aortic surgery |
| R. Kermani^111^ | 2013 | A practical mortality risk score for emergent colectomy | Did not use a consensus definition of AKI |
| J. C. Duchesne^112^ | 2013 | Diluting the benefits of hemostatic resuscitation: a multi-institutional analysis | Did not use a consensus definition of AKI |
| J. M. Blum^113^ | 2013 | Preoperative and intraoperative predictors of postoperative acute respiratory distress syndrome in a general surgical population | Data on AKI not available for the abdominal surgery |
| A. Bihorac^114^ | 2013 | National surgical quality improvement program underestimates the risk associated with mild and moderate postoperative acute kidney injury | Data on AKI not available for the abdominal surgery |
| B. K. Ahn^115^ | 2013 | Single-dose antibiotic prophylaxis is effective enough in colorectal surgery | Data on AKI not available for the abdominal surgery |
| A. M. Yassen^116^ | 2012 | Low dose ketorolac infusion improves postoperative analgesia combined with patient controlled fentanyl analgesia after living donor hepatectomy - Randomized controlled trial | Data on AKI not available for the abdominal surgery |
| S. Kondalsamy-Chennakesavan^117^ | 2012 | Risk factors to predict the incidence of surgical adverse events following open or laparoscopic surgery for apparent early stage endometrial cancer: results from a randomised controlled trial | Data on AKI not available for the abdominal surgery |
| M. Hübner^118^ | 2012 | Preoperative immunonutrition in patients at nutritional risk: results of a double-blinded randomized clinical trial | Data on AKI not available for the abdominal surgery |
| A. Endo^119^ | 2012 | Intraoperative hydroxyethyl starch 70/0.5 is not related to acute kidney injury in surgical patients: retrospective cohort study | Data on AKI not available for the abdominal surgery |
| Marchenko T. V.^120^ | 2019 | Renal replacement therapy after elective surgical procedures | Data on AKI not available for the abdominal surgery |
| Long T. E.^121^ | 2013 | Epidemiology of acute kidney injury in a tertiary care university hospital according to the RIFLE criteria | Data on AKI not available for the abdominal surgery |
| E. Y. Woo^122^ | 2011 | Open abdominal aortic aneurysm repair is feasible and can be done with excellent results in octogenarians | Did not use a consensus definition of AKI |
| A. O. Molnar^123^ | 2011 | Statin use associates with a lower incidence of acute kidney injury after major elective surgery | Data on AKI not available for the abdominal surgery and did not use a consensus definition of AKI |
| G. Jeyabalan^124^ | 2011 | Comparison of modern open infrarenal and pararenal abdominal aortic aneurysm repair on early outcomes and renal dysfunction at one year | Did not use a consensus definition of AKI |
| T. Suzuki^125^ | 2010 | Fluid therapy with hydroxyethyl starch for massive blood loss during surgery | Data on AKI not available for the abdominal surgery |
| C. Spyropoulos^126^ | 2010 | Revisional bariatric surgery: 13-year experience from a tertiary institution | Did not use a consensus definition of AKI |
| F. Speziale^127^ | 2010 | Factors influencing outcome after open surgical repair of juxtarenal abdominal aortic aneurysms | Did not use a consensus definition of AKI |
| J. P. van Kuijk^128^ | 2011 | Preoperative left ventricular dysfunction predisposes to postoperative acute kidney injury and long-term mortality | Data on AKI not available for the open abdominal aortic surgery |
| P. Burra^129^ | 2009 | Factors influencing renal function after liver transplantation. Results from the MOST, an international observational study | Did not use a consensus definition of AKI |
| D. J. Bentrem^130^ | 2009 | Identification of specific quality improvement opportunities for the elderly undergoing gastrointestinal surgery | Did not use a consensus definition of AKI |
| M. Antonello^131^ | 2009 | Open repair for ruptured abdominal aortic aneurysm: is it possible to predict survival? | Did not use a consensus definition of AKI |
| F. J. Abelha^132^ | 2009 | Determinants of postoperative acute kidney injury | Data on AKI not available for the abdominal surgery |
| L. C. Brown^133^ | 2008 | Fit patients with small abdominal aortic aneurysms (AAAs) do not benefit from early intervention | Data on AKI not available for the open abdominal aortic surgery and did not use a consensus definition of AKI |
| M. Pernicky^134^ | 2007 | Perioperative cardiovascular and noncardiovascular risk in patients with colorectal cancer | Did not use a consensus definition of AKI |
| J. Zhou^135^ | 2006 | Conversion to sirolimus immunosuppression in liver transplantation recipients with hepatocellular carcinoma: Report of an initial experience | Did not use a consensus definition of AKI |
| C. Senekowitsch^136^ | 2006 | Replanting the inferior mesentery artery during infrarenal aortic aneurysm repair: influence on postoperative colon ischemia | Data on AKI not available for the open abdominal aortic surgery |
| D. A. de Freitas Carvalho^137^ | 2006 | Rhabdomyolysis after bariatric surgery | Did not use a consensus definition of AKI |
| W. B. Inabnet^138^ | 2005 | Laparoscopic Roux-en-Y gastric bypass in patients with BMI <50: a prospective randomized trial comparing short and long limb lengths | Data on AKI not available for the open abdominal surgery |
| L. Sjöström^139^ | 2005 | Lifestyle, diabetes, and cardiovascular risk factors 10 years after bariatric surgery | Data on AKI not available for the open abdominal surgery |
| F. A. Kudo^140^ | 2005 | Postoperative renal function after elective abdominal aortic aneurysm repair requiring suprarenal aortic cross-clamping | Did not use a consensus definition of AKI |
| P. H. Lo^141^ | 2021 | Adverse Outcomes after Non-Cardiac Surgeries in Patients with Heart Failure: A Propensity-Score Matched Study | Did not use a consensus definition of AKI |
| J. T. Geiger^142^ | 2021 | One-year patient survival correlates with surgeon volume after elective open abdominal aortic surgery | Did not use a consensus definition of AKI |
| S. J. Choo^143^ | 2021 | Outcomes of emergency endovascular versus open repair for abdominal aortic aneurysm rupture | Did not use a consensus definition of AKI |
| L. Chen^144^ | 2021 | New score for prediction of morbidity in patients undergoing open pancreaticoduodenectomy | Data on AKI not available for the open abdominal surgery and did not use a consensus definition of AKI |
| M. Yazawa^145^ | 2020 | Longitudinal Renal Function in Liver Transplant Recipients With Acute-on-Chronic Liver Failure | Did not use a consensus definition of AKI |
| K. T. Weber^146^ | 2020 | Effect of Body Mass Index on Outcomes After Surgery for Perforated Diverticulitis | Data on AKI not available for the open abdominal surgery and did not use a consensus definition of AKI |
| Y. Sanaiha^147^ | 2020 | Acute kidney injury is independently associated with mortality and resource use after emergency general surgery operations | Did not use a consensus definition of AKI |
| A. Z. Paredes^148^ | 2020 | Skilled nursing facility (SNF) utilization and impact of SNF star-quality ratings on outcomes following hepatectomy among Medicare beneficiaries | Did not use a consensus definition of AKI |
| V. Liakopoulos^149^ | 2020 | Renal and cardiovascular outcomes after weight loss from gastric bypass surgery in type 2 diabetes: Cardiorenal risk reductions exceed atherosclerotic benefits | Did not use a consensus definition of AKI |
| H. J. Lee^150^ | 2020 | Different Severity of Clinical Outcomes Between the 2 Subgroups of Stage 1 Acute Kidney Injury After Liver Transplantation | Only the number of patients in AKI stage 1 was reported |
| J. W. Kunstman^151^ | 2020 | Comprehensive Analysis of the Effect of Ketorolac Administration after Pancreaticoduodenectomy | Did not use a consensus definition of AKI |
| J. Jin^152^ | 2020 | Prior bariatric surgery and perioperative cardiovascular outcomes following noncardiac surgery in patients with type 2 diabetes mellitus: Hint from National Inpatient Sample Database | Did not use a consensus definition of AKI |
| Y. Jiang^153^ | 2020 | Sarcoidosis is associated with lower risks of penetrating disease and colectomy in hospitalized patients with inflammatory bowel disease | Did not use a consensus definition of AKI |
| O. D. Drazic^154^ | 2020 | Juxtarenal Abdominal Aortic Aneurysm: Results of Open Surgery in an Academic Center | Did not use a consensus definition of AKI |
| C. Y. Chen^155^ | 2020 | Risk factors of acute renal impairment after cytoreductive surgery and hyperthermic intraperitoneal chemotherapy | Did not use a consensus definition of AKI |
| E. H. Chang^156^ | 2020 | Obesity and surgical complications of pancreaticoduodenectomy: An observation study utilizing ACS NSQIP | Did not use a consensus definition of AKI |
| V. S. Are^157^ | 2020 | Improving Outcomes of Bariatric Surgery in Patients With Cirrhosis in the United States: A Nationwide Assessment | Did not use a consensus definition of AKI |
| F. Alconchel^157^ | 2020 | Impact of Hepatic Artery Thrombosis on the Success of a Liver Transplant Because of Hepatocellular Carcinoma | Did not use a consensus definition of AKI |
| L. Weinberg^158^ | 2019 | Impact of a goal directed fluid therapy algorithm on postoperative morbidity in patients undergoing open right hepatectomy: A single centre retrospective observational study | Did not use a consensus definition of AKI |
| Y. A. Tarbunou^159^ | 2019 | Outcomes associated with hyperglycemia after abdominal aortic aneurysm repair | Did not use a consensus definition of AKI |
| P. Sharma^160^ | 2019 | Impact of Bariatric Surgery on Outcomes of Patients with Sickle Cell Disease: a Nationwide Inpatient Sample Analysis, 2004-2014 | Did not use a consensus definition of AKI |
| B. J. Sandler^161^ | 2019 | Symptomatic human immunodeficiency virus-infected patients have poorer outcomes following emergency general surgery: A study of the nationwide inpatient sample | Did not use a consensus definition of AKI |
| D. M. Pechman^162^ | 2019 | Bariatric surgery in the elderly: outcomes analysis of patients over 70 using the ACS-NSQIP database | Did not use a consensus definition of AKI |
| A. Ninh^163^ | 2019 | Risk Factors and Outcomes for Sepsis after Appendectomy in Adults | Did not use a consensus definition of AKI |
| T. Ma^164^ | 2019 | Value of pretransplant albumin-bilirubin score in predicting outcomes after liver transplantation | Did not use a consensus definition of AKI |
| C. S. Lin^165^ | 2019 | Defining risk of general surgery in patients with chronic obstructive pulmonary diseases | Did not use a consensus definition of AKI |
| J. Levitsky^166^ | 2019 | External Validation of a Pretransplant Biomarker Model (REVERSE) Predictive of Renal Recovery After Liver Transplantation | Did not use a consensus definition of AKI |
| S. Lee^167^ | 2019 | Long-term impact of dialysis-requiring AKI during the perioperative period of liver transplantation on postdischarge outcomes | Did not use a consensus definition of AKI |
| M. Kim^168^ | 2019 | Latent class analysis stratifies mortality risk in patients developing acute kidney injury after high-risk intraabdominal general surgery: a historical cohort study | Did not use a consensus definition of AKI |
| E. L. Keuffel^169^ | 2019 | Hospital costs associated with intraoperative hypotension among non-cardiac surgical patients in the US: a simulation model | Did not use a consensus definition of AKI |
| A. Kazemi^170^ | 2019 | Identifying Factors That Affect Patient Survival After Orthotopic Liver Transplant Using Machine-Learning Techniques | Did not use a consensus definition of AKI |
| J. Hefler^171^ | 2019 | Effects of Chronic Corticosteroid and Immunosuppressant Use in Patients Undergoing Bariatric Surgery | Did not use a consensus definition of AKI |
| S. W. Grahn^172^ | 2019 | System-Wide Improvement for Transitions After Ileostomy Surgery: Can Intensive Monitoring of Protocol Compliance Decrease Readmissions? A Randomized Trial | Did not use a consensus definition of AKI |
| T. Dang^173^ | 2019 | Predictors of acute kidney injury after infrarenal abdominal aortic aneurysm repair in octogenarians | Did not use a consensus definition of AKI |
| E. Braunwarth^174^ | 2019 | Is bile leakage after hepatic resection associated with impaired long-term survival? | Did not use a consensus definition of AKI |
| A. Y. Ali^175^ | 2019 | Effect of Duration of Intensive Care Unit Stay on Outcomes of Adult Living Donor Liver Transplant Recipients | Did not use a consensus definition of AKI |
| K. C. Yoon^176^ | 2018 | Urinary Neutrophil Gelatinase-Associated Lipocalin as a Biomarker for Renal Injury in Liver Transplant Recipients Using Calcineurin Inhibitors | Did not use a consensus definition of AKI |
| M. Sugimoto^177^ | 2018 | Long-term fate of renal function after open surgery for juxtarenal and pararenal aortic aneurysm | Did not use a consensus definition of AKI |
| K. Maheshwari^178^ | 2018 | The association of hypotension during non-cardiac surgery, before and after skin incision, with postoperative acute kidney injury: a retrospective cohort analysis | Did not use a consensus definition of AKI |
| Y. F. Lin^179^ | 2018 | Short-and long-term outcomes after postsurgical acute kidney injury requiring dialysis | Did not use a consensus definition of AKI |
| U. Koppe^180^ | 2018 | Long-term effects of bariatric surgery on acute kidney injury: a propensity-matched cohort in the UK Clinical Practice Research Datalink | Did not use a consensus definition of AKI |
| M. Kim^181^ | 2018 | Risk Stratification for Major Postoperative Complications in Patients Undergoing Intra-abdominal General Surgery Using Latent Class Analysis | Did not use a consensus definition of AKI |
| M. Kim^182^ | 2018 | Two-way Interaction Effects of Perioperative Complications on 30-Day Mortality in General Surgery | Did not use a consensus definition of AKI and research object duplication^181^ |
| W. T. Kassahun^183^ | 2018 | Factors associated with morbidity and in-hospital mortality after surgery beyond the age of 90: Comparison with outcome results of younger patients matched for treatment | Did not use a consensus definition of AKI |
| N. S. Ilic^184^ | 2018 | Evaluation of the renal function using serum Cystatin C following open and endovascular aortic aneurysm repair | Did not use a consensus definition of AKI |
| R. M. Higgins^185^ | 2018 | Preoperative immobility significantly impacts the risk of postoperative complications in bariatric surgery patients | Did not use a consensus definition of AKI |
| R. H. Blackwell^186^ | 2018 | Complications of Recognized and Unrecognized Iatrogenic Ureteral Injury at Time of Hysterectomy: A Population Based Analysis | Did not use a consensus definition of AKI |
| C. A. Behrendt^187^ | 2018 | Incidence, Predictors, and Outcomes of Colonic Ischaemia in Abdominal Aortic Aneurysm Repair | Did not use a consensus definition of AKI |
| D. Antoniak^188^ | 2018 | The Relationship Between Age and Chronic Kidney Disease in Patients Undergoing Pancreatic Resection | Did not use a consensus definition of AKI |
| D. Acheampong^189^ | 2018 | Unplanned postoperative reintubation following general and vascular surgical procedures: Outcomes and risk factors | Did not use a consensus definition of AKI |
| Y. Zongyi^190^ | 2017 | Risk factors of acute kidney injury after orthotopic liver transplantation in China | Did not use a consensus definition of AKI |
| J. J. Siracuse^191^ | 2017 | Contemporary open repair of ruptured abdominal aortic aneurysms | Did not use a consensus definition of AKI |
| E. I. L^192^ | 2017 | Acute kidney injury in ovarian cancer patients undergoing cytoreductive surgery and hyperthermic intra-peritoneal chemotherapy | Did not use a consensus definition of AKI |
| Y. Pan^193^ | 2017 | Incidence and Risk Factors of in-hospital mortality from AKI after non-cardiovascular operation: A nationwide Survey in China | Did not use a consensus definition of AKI |
| A. R. Nandan^194^ | 2017 | The Emergency Surgery Score (ESS) accurately predicts the occurrence of postoperative complications in emergency surgery patients | Data on AKI not available for the open abdominal surgery and did not use a consensus definition of AKI |
| M. D. Maile^195^ | 2017 | Impact of ejection fraction on infectious, renal, and respiratory morbidity for patients undergoing noncardiac surgery | Did not use a consensus definition of AKI |
| L. C. Li^196^ | 2017 | Proteinuria and baseline renal function predict mortality and renal outcomes after sirolimus therapy in liver transplantation recipients | Did not use a consensus definition of AKI |
| L. Li^197^ | 2017 | Ileostomy creation in colorectal cancer surgery: risk of acute kidney injury and chronic kidney disease | Did not use a consensus definition of AKI |
| S. Y. Lee^198^ | 2017 | Perioperative Infections after Open Abdominal Aortic Aneurysm Repair Lead to Increased Risk of Subsequent Complications | Did not use a consensus definition of AKI |
| C. Kunisaki^199^ | 2017 | Modeling preoperative risk factors for potentially lethal morbidities using a nationwide Japanese web-based database of patients undergoing distal gastrectomy for gastric cancer | Did not use a consensus definition of AKI |
| P. Kumar^200^ | 2017 | Outcome of noncardiac surgical patients admitted to a multidisciplinary intensive care unit | Did not use a consensus definition of AKI |
| A. S. Kulaylat^201^ | 2017 | A Surgical Clostridium-Associated Risk of Death Score Predicts Mortality After Colectomy for Clostridium difficile | Did not use a consensus definition of AKI |
| S. G. Kim^202^ | 2017 | Beneficial and harmful effects of nonselective beta blockade on acute kidney injury in liver transplant candidates | Did not use a consensus definition of AKI |
| D. Hokuto^203^ | 2017 | The Administration of Celecoxib as an Analgesic after Liver Resection Is Safe | Did not use a consensus definition of AKI |
| C. W. Hicks^204^ | 2017 | Predicting failure to rescue after abdominal aortic aneurysm repair in elderly patients | Did not use a consensus definition of AKI |
| W. Ding^205^ | 2017 | Open Abdomen Improves Survival in Patients With Peritonitis Secondary to Acute Superior Mesenteric Artery Occlusion | Did not use a consensus definition of AKI |
| W. C. Chapman^206^ | 2017 | Effect of Early Everolimus-Facilitated Reduction of Tacrolimus on Efficacy and Renal Function in De Novo Liver Transplant Recipients: 24-Month Results for the North American Subpopulation | Did not use a consensus definition of AKI |
| R. Babazade^207^ | 2017 | Systemic Lupus Erythematosus Is Associated With Increased Adverse Postoperative Renal Outcomes and Mortality: A Historical Cohort Study Using Administrative Health Data | Did not use a consensus definition of AKI |
| R. F. Alizadeh^208^ | 2017 | Venous thromboembolism in common laparoscopic abdominal surgical operations | Did not use a consensus definition of AKI |
| H. M. Wadei^209^ | 2016 | Early Allograft Dysfunction After Liver Transplantation Is Associated With Short- and Long-Term Kidney Function Impairment | Only the number of patients in AKI stage 3 |
| A. E. Tahaoglu^210^ | 2016 | Emergency peripartum hysterectomy: our experience | Did not use a consensus definition of AKI |
| K. M. Ramonell^211^ | 2016 | Development and Validation of a Risk Calculator for Renal Co mplicatio ns after Colorectal Surgery Using the National Surgical Quality Improvement Program Participant Use Files | Did not use a consensus definition of AKI |
| T. Ozrazgat-Baslanti^212^ | 2016 | Preoperative assessment of the risk for multiple complications after surgery | Data on AKI not available for the abdominal surgery |
| Y. Li^213^ | 2016 | Association of preoperative narcotic use with postoperative complications and prolonged length of hospital stay in patients with Crohn disease | Did not use a consensus definition of AKI |
| G. W. Kang^214^ | 2016 | One-Year Follow-up of the Changes in Renal Function After Liver Transplantation in Patients Without Chronic Kidney Disease | Did not use a consensus definition of AKI |
| Y. Inoue^215^ | 2016 | Does the development of chronic kidney disease and acute kidney injury affect the prognosis after living donor liver transplantation? | Did not use a consensus definition of AKI |
| A. M. Ferrante^216^ | 2016 | Results after elective open repair of pararenal abdominal aortic aneurysms | Did not use a consensus definition of AKI |
| M. Desai^217^ | 2016 | Sex-related trends inmortality after elective abdominal aortic aneurysmsurgery between 2002 and 2013 at National Health Service hospitals in England: Less benefit for women compared with men | Did not use a consensus definition of AKI |
| S. E. Daniels^218^ | 2016 | A Pooled Analysis Evaluating Renal Safety in Placebo- and Active Comparator-Controlled Phase III Trials of Multiple-Dose Injectable HPβCD-Diclofenac in Subjects with Acute Postoperative Pain | Did not use a consensus definition of AKI |
| J. D. Blitz^219^ | 2016 | Preoperative Renal Insufficiency: Underreporting and Association With Readmission and Major Postoperative Morbidity in an Academic Medical Center | Did not use a consensus definition of AKI |
| Z. Aga^220^ | 2016 | Greater intravenous fluid volumes are associated with prolonged recovery after colorectal surgery: a retrospective cohort study | Did not use a consensus definition of AKI |
| H. R. Abdullah^221^ | 2016 | Predictors of Perioperative Acute Kidney Injury in Obese Patients Undergoing Laparoscopic Bariatric Surgery: a Single-Centre Retrospective Cohort Study | Did not use a consensus definition of AKI |
| E. Wakeam^222^ | 2015 | Risk and patterns of secondary complications in surgical inpatients | Did not use a consensus definition of AKI |
| A. Leon-Justel^223^ | 2015 | Point-of-care haemostasis monitoring during liver transplantation reduces transfusion requirements and improves patient outcome | Did not use a consensus definition of AKI |
| M. Kim^224^ | 2015 | Interaction Effects of Acute Kidney Injury, Acute Respiratory Failure, and Sepsis on 30-Day Postoperative Mortality in Patients Undergoing High-Risk Intraabdominal General Surgical Procedures | Did not use a consensus definition of AKI |
| J. Zhang^225^ | 2014 | Acute kidney injury after radical gastrectomy: a single center study | Did not use a consensus definition of AKI |
| M. H. Squires^226^ | 2014 | Effect of preoperative renal insufficiency on postoperative outcomes after pancreatic resection: a single institution experience of 1,061 consecutive patients | Did not use a consensus definition of AKI |
| P. Smoter^227^ | 2014 | Risk factors of acute renal failure after orthotopic liver transplantation: single-center experience | Did not use a consensus definition of AKI |
| F. Klaus^228^ | 2014 | Acute kidney injury after liver transplantation: incidence and mortality | Did not use a consensus definition of AKI |
| C. W. Kimbrough^229^ | 2014 | Factors predictive of readmission after hepatic resection for hepatocellular carcinoma | Did not use a consensus definition of AKI |
| M. Kim^230^ | 2014 | Variations in the risk of acute kidney injury across intraabdominal surgery procedures | Did not use a consensus definition of AKI |
| J. M. Kim^231^ | 2014 | The predictors for continuous renal replacement therapy in liver transplant recipients | Data on AKI not available for the open liver transplant surgery |
| F. Aberg^232^ | 2014 | Neutrophil gelatinase-associated lipocalin associated with irreversibility of pre-liver transplant kidney dysfunction | Did not use a consensus definition of AKI |
| M. D. Jafari^233^ | 2013 | Morbidity of diverting ileostomy for rectal cancer: analysis of the American College of Surgeons National Surgical Quality Improvement Program | Did not use a consensus definition of AKI |
| M. Suzuki^234^ | 2012 | Risk factors for native kidney dysfunction in patients with abdominal multivisceral/small bowel transplantation | Did not use a consensus definition of AKI |
| H. Masoomi^235^ | 2012 | Predictive factors of acute renal failure in colon and rectal surgery | Did not use a consensus definition of AKI |
| Y. H. Lin^236^ | 2012 | The 4-week serum creatinine level predicts long-term renal dysfunction after adult living donor liver transplantation | Did not use a consensus definition of AKI |
| M. E. Lidsky^237^ | 2012 | Advanced age is an independent predictor for increased morbidity and mortality after emergent surgery for diverticulitis | Did not use a consensus definition of AKI |
| I. Kopolovic^238^ | 2012 | Elevated cardiac troponin in the early post-operative period and mortality following ruptured abdominal aortic aneurysm: a retrospective population-based cohort study | Did not use a consensus definition of AKI |
| A. Siniscalchi^239^ | 2012 | Postoperative troponin T elevation as a predictor of early acute kidney injury after orthotopic liver transplantation: a preliminary retrospective study | Did not use a consensus definition of AKI |
| A. Karapanagiotou^240^ | 2012 | Acute kidney injury after orthotopic liver transplantation | Did not use a consensus definition of AKI |
| Y. Inoue^241^ | 2012 | Acute kidney injury following living donor liver transplantation | Did not use a consensus definition of AKI |
| L. Huang^242^ | 2012 | Prealbumin is predictive for postoperative liver insufficiency in patients undergoing liver resection | Did not use a consensus definition of AKI |
| C. J. Hu^243^ | 2012 | Postoperative adverse outcomes in surgical patients with dementia: a retrospective cohort study | Did not use a consensus definition of AKI |
| S. H. Hong^244^ | 2012 | Prediction of newly developed acute renal failure using serum phosphorus concentrations after living-donor liver transplantation | Did not use a consensus definition of AKI |
| I. F. Boin^245^ | 2012 | Can pre-liver transplantation renal insufficiency using a creatinine clearance calculator predict long-term survival? | Data on AKI not available for the abdominal surgery and did not use a consensus definition of AKI |
| B. Zhu^246^ | 2011 | Combined invagination and duct-to-mucosa techniques with modifications: a new method of pancreaticojejunal anastomosis | Did not use a consensus definition of AKI |
| M. S. Zand^247^ | 2011 | High mortality in orthotopic liver transplant recipients who require hemodialysis | Did not use a consensus definition of AKI |
| E. C. Verna^248^ | 2011 | Basiliximab induction and delayed calcineurin inhibitor initiation in liver transplant recipients with renal insufficiency | Data on AKI not available for the liver transplant surgery and did not use a consensus definition of AKI |
| B. Park^249^ | 2011 | Obesity is not an independent risk factor for adverse perioperative and long-term clinical outcomes following open AAA repair or EVAR | Did not use a consensus definition of AKI |
| G. A. Mashour^250^ | 2011 | Perioperative stroke and associated mortality after noncardiac, nonneurologic surgery | Did not use a consensus definition of AKI |
| Q. Ling^251^ | 2011 | Impact of preexisting diabetes mellitus on outcome after liver transplantation in patients with hepatitis B virus-related liver disease | Did not use a consensus definition of AKI |
| A. Memmo^252^ | 2011 | Perioperative fenoldopam for the prevention of acute renal failure in non-cardiac surgery, randomized clinical trial | Did not use a consensus definition of AKI |
| J. A. Lin^253^ | 2011 | Postoperative adverse outcomes in intellectually disabled surgical patients: a nationwide population-based study | Did not use a consensus definition of AKI |
| J. A. Leithead^254^ | 2011 | Chronic kidney disease after liver transplantation for acute liver failure is not associated with perioperative renal dysfunction | Only the number of patients in AKI RIFLE – I or higher was reported |
| J. Chen^255^ | 2011 | Postliver transplant acute renal injury and failure by the RIFLE criteria in patients with normal pretransplant serum creatinine concentrations: a matched study | Only the number of patients in AKI RIFLE –I or RIFLE–F was reported |
| Z. M. Arthurs^256^ | 2011 | A comparison of endovascular revascularization with traditional therapy for the treatment of acute mesenteric ischemia | Did not use a consensus definition of AKI |
| J. Ramachandran^257^ | 2010 | Chronic kidney disease following liver transplantation: a South Australian experience | Did not use a consensus definition of AKI |
| A. K. Mathur^258^ | 2010 | Influence of body mass index on complications and oncologic outcomes following hepatectomy for malignancy | Did not use a consensus definition of AKI |
| M. C. Martin^259^ | 2010 | National outcomes after open repair of abdominal aortic aneurysms with visceral or renal bypass | Did not use a consensus definition of AKI |
| N. A. Khan^260^ | 2010 | Risk of intraoperative hypotension with loop diuretics: a randomized controlled trial | Did not use a consensus definition of AKI |
| L. G. Glance^261^ | 2010 | Perioperative outcomes among patients with the modified metabolic syndrome who are undergoing noncardiac surgery | Did not use a consensus definition of AKI |
| R. S. Davies^262^ | 2010 | Outcome in patients requiring renal replacement therapy after open surgical repair for ruptured abdominal aortic aneurysm | Did not use a consensus definition of AKI |
| B. Subramaniam^263^ | 2009 | Continuous perioperative insulin infusion decreases major cardiovascular events in patients undergoing vascular surgery: a prospective, randomized trial | Did not use a consensus definition of AKI |
| G. Silecchia^264^ | 2009 | Two-stage laparoscopic biliopancreatic diversion with duodenal switch as treatment of high-risk super-obese patients: analysis of complications | Did not use a consensus definition of AKI |
| A. J. Senagore^265^ | 2009 | A national comparison of laparoscopic vs. open colectomy using the National Surgical Quality Improvement Project data | Did not use a consensus definition of AKI |
| C. Paugam-Burtz^266^ | 2009 | Postreperfusion syndrome during liver transplantation for cirrhosis: outcome and predictors | Did not use a consensus definition of AKI |
| N. N. Massarweh^267^ | 2009 | Impact of advancing age on abdominal surgical outcomes | Did not use a consensus definition of AKI |
| S. Kheterpal^268^ | 2009 | Development and validation of an acute kidney injury risk index for patients undergoing general surgery: results from a national data set | Did not use a consensus definition of AKI |
| N. D. Karanjia^269^ | 2009 | Survival and recurrence after neo-adjuvant chemotherapy and liver resection for colorectal metastases: a ten year study | Did not use a consensus definition of AKI |
| W. A. Hackworth^270^ | 2009 | Effect of hyponatraemia on outcomes following orthotopic liver transplantation | Did not use a consensus definition of AKI |
| J. C. Duchesne^271^ | 2009 | Impact of obesity in damage control laparotomy patients | Did not use a consensus definition of AKI |
| E. Charbonney^272^ | 2009 | Prognosis of acute kidney injury requiring renal replacement therapy in solid organ transplanted patients | Did not use a consensus definition of AKI |
| S. H. Nasr^273^ | 2008 | Oxalate nephropathy complicating Roux-en-Y Gastric Bypass: an underrecognized cause of irreversible renal failure | Did not use a consensus definition of AKI |
| R. C. Afonso^274^ | 2008 | Impact of renal failure on liver transplantation survival | Data on AKI not available for the liver transplant surgery |
| O. Sizzi^275^ | 2007 | Italian multicenter study on complications of laparoscopic myomectomy | Did not use a consensus definition of AKI |
| F. A. Robledo^276^ | 2007 | Open versus closed management of the abdomen in the surgical treatment of severe secondary peritonitis: a randomized clinical trial | Did not use a consensus definition of AKI |
| A. O'Riordan^277^ | 2007 | Acute renal disease, as defined by the RIFLE criteria, post-liver transplantation | Only the number of patients in AKI RIFLE –I or RIFLE–F was reported |
| S. Kheterpal^278^ | 2007 | Predictors of postoperative acute renal failure after noncardiac surgery in patients with previously normal renal function | Did not use a consensus definition of AKI |
| F. A. Herrera^279^ | 2007 | The prevalence of obesity and postoperative complications in a Veterans Affairs Medical Center general surgery population | Data on AKI not available for the abdominal surgery |
| F. Aberg^280^ | 2007 | Renal dysfunction in liver transplant patients: comparing patients transplanted for liver tumor or acute or chronic disease | Did not use a consensus definition of AKI |
| Y. Wei^281^ | 2006 | Factors Related to Post-Liver Transplantation Acute Renal Failure | Did not use a consensus definition of AKI |
| M. C. Londoño^282^ | 2006 | Hyponatremia impairs early posttransplantation outcome in patients with cirrhosis undergoing liver transplantation | Did not use a consensus definition of AKI |
| S. M. Lobo^283^ | 2006 | Prospective, randomized trial comparing fluids and dobutamine optimization of oxygen delivery in high-risk surgical patients | Did not use a consensus definition of AKI |
| G. Junge^284^ | 2006 | Acute renal failure after liver transplantation: incidence, etiology, therapy, and outcome | Did not use a consensus definition of AKI |
| P. N. Johnson^285^ | 2006 | Analysis of morbidity in liver transplant recipients following human albumin supplementation: a retrospective pilot study | Data on AKI not available for the liver transplant surgery |
| J. Guitard^286^ | 2006 | Acute renal failure following liver transplantation with induction therapy | Only the number of patients in AKI RIFLE –I or RIFLE–F was reported |
| S. Faenza^287^ | 2006 | Acute renal failure requiring renal replacement therapy after orthotopic liver transplantation | Did not use a consensus definition of AKI |
| C. Ellenberger^288^ | 2006 | Incidence, risk factors and prognosis of changes in serum creatinine early after aortic abdominal surgery | Did not use a consensus definition of AKI |
| J. M. Alamo^289^ | 2006 | Morbidity and mortality in liver retransplantation | Did not use a consensus definition of AKI |
| M. A. Acosta-Merida^290^ | 2006 | Identification of risk factors for perioperative mortality in acute mesenteric ischemia | Did not use a consensus definition of AKI |
| C. C. Lin^291^ | 2005 | The renal-sparing efficacy of basiliximab in adult living donor liver transplantation | Did not use a consensus definition of AKI |
| C. M. Wyatt^292^ | 2004 | The burden of acute renal failure in nonrenal solid organ transplantation | Did not use a consensus definition of AKI |
| C. Vemuri^293^ | 2004 | Effect of increasing patient age on complication rates following intact abdominal aortic aneurysm repair in the United States | Did not use a consensus definition of AKI |
| E. Q. Sanchez^294^ | 2004 | Preoperative and perioperative predictors of the need for renal replacement therapy after orthotopic liver transplantation | Did not use a consensus definition of AKI |
| A. S. Paramesh^295^ | 2004 | Post-liver transplant acute renal failure: factors predicting development of end-stage renal disease | Did not use a consensus definition of AKI |
| M. Lebrón Gallardo^296^ | 2004 | Risk factors for renal dysfunction in the postoperative course of liver transplant | Did not use a consensus definition of AKI |
| S. G. Kim^297^ | 2004 | Incidence and risk factors of renal dysfunction after liver transplantation in Korea | Did not use a consensus definition of AKI |
| R. N. Khurana^298^ | 2004 | Postoperative rhabdomyolysis following laparoscopic gastric bypass in the morbidly obese | Did not use a consensus definition of AKI |
| F. R. Chuang^299^ | 2004 | Acute renal failure after cadaveric related liver transplantation | Did not use a consensus definition of AKI |
| P. Balachandran^300^ | 2004 | Haemorrhagic complications of pancreaticoduodenectomy | Did not use a consensus definition of AKI |
| Shen Z. Y.^301^ | 2006 | Risk factors for renal failure during the early period after standard orthotopic liver transplantation without veno venous bypass | Did not use a consensus definition of AKI |
| Zhu F. X.^302^ | 2005 | Risk factors of renal failure in the early post-liver transplantation period | Did not use a consensus definition of AKI |
| Yuan C. H.^303^ | 2011 | The influential factors and clinical significance of acute renal failure complicated to orthotopic liver transplantation | Did not use a consensus definition of AKI |
| Sabaté S.^304^ | 2011 | Risk factors for postoperative acute kidney injury in a cohort of 2378 patients from 59 hospitals | Did not use a consensus definition of AKI |
| Nemes B.^305^ | 2015 | Bacterial infection after orthotopic liver transplantation | Did not use a consensus definition of AKI |
| Wang Y.^306^ | 2006 | Study on risk factors for early postoperative infection after orthotopic liver transplantation in adults | Did not use a consensus definition of AKI |
| Seller-Pérez G.^307^ | 2004 | Postoperative complications of liver transplantation: relationship with mortality | Did not use a consensus definition of AKI |
| Koscielny A.^308^ | 2016 | Ruptured Abdominal Aortic Aneurysm - Results and Prognostic Factors at a Certified Centre of Vascular Surgery | Did not use a consensus definition of AKI |
| E. Futier^309^ | 2020 | Effect of Hydroxyethyl Starch vs Saline for Volume Replacement Therapy on Death or Postoperative Complications Among High-Risk Patients Undergoing Major Abdominal Surgery: The FLASH Randomized Clinical Trial | Data on AKI not available for the abdominal surgery |
| P. S. Myles ^310^ | 2019 | Importance of intraoperative oliguria during major abdominal surgery: findings of the Restrictive versus Liberal Fluid Therapy in Major Abdominal Surgery trial | Data on AKI not available for the abdominal surgery and research object duplication^311^ |
| I. Dedinská^312^ | 2018 | Acute kidney injury after liver resection in elderly patients | Did not use a consensus definition of AKI |
| P. S. Myles^311^ | 2018 | Restrictive versus Liberal Fluid Therapy for Major Abdominal Surgery | Data on AKI not available for the abdominal surgery |
| A. P. C. S. Boteon^313^ | 2018 | The impact of transarterial chemoembolization induced complications on outcomes after liver transplantation: A propensity-matched study | Did not use a consensus definition of AKI |
| J. Ripolles-Melchor^314^ | 2019 | Association Between Use of Enhanced Recovery After Surgery Protocol and Postoperative Complications in Colorectal Surgery: The Postoperative Outcomes Within Enhanced Recovery After Surgery Protocol (POWER) Study | Research object duplication^315^ |
| A. Turan^316^ | 2020 | Mild Acute Kidney Injury after Noncardiac Surgery Is Associated with Long-term Renal Dysfunction: A Retrospective Cohort Study | Research object duplication^315^ |
| D. J. Roberts^317^ | 2021 | Angiotensin-Converting Enzyme Inhibitor/ Receptor Blocker, Diuretic, or Nonsteroidal Antiinﬂammatory Drug Use After Major Surgery and Acute Kidney Injury: A Case-Control Study | Data on AKI not available for the abdominal surgery |
| I. Gocze^318^ | 2018 | Biomarker-guided Intervention to Prevent Acute Kidney Injury After Major Surgery | Data on AKI not available for the abdominal surgery |
| K. Trongtrakul^319^ | 2021 | External Validation of the Acute Kidney Injury Risk Prediction Score for Critically Ill Surgical Patients Who Underwent Major Non-Cardiothoracic Surgery | Data on AKI not available for the abdominal surgery and research object duplication^63^ |
| M. Y. Argalious^320^ | 2013 | The association of preoperative statin use and acute kidney injury after noncardiac surgery | Research object duplication^321^ |
| J. W. Shim^322^ | 2020 | Impact of intraoperative zero-balance fluid therapy on the occurrence of acute kidney injury in patients who had undergone colorectal cancer resection within an enhanced recovery after surgery protocol: a propensity score matching analysis | Research object duplication^323^ |
| J. W. Shim^324^ | 2020 | Role of intraoperative oliguria in risk stratification for postoperative acute kidney injury in patients undergoing colorectal surgery with an enhanced recovery protocol: A propensity score matching analysis | Research object duplication^323^ |
| K. Slankamenac^325^ | 2009 | Development and validation of a prediction score for postoperative acute renal failure following liver resection | Research object duplication^326^ |
| T. Mizota^327^ | 2019 | Transient acute kidney injury after major abdominal surgery increases chronic kidney disease risk and 1-year mortality | Research object duplication^328^ |
| T. K. Oh^329^ | 2018 | Chronic hyperglycemia with elevated glycated hemoglobin level and its association with postoperative acute kidney injury after a major laparoscopic abdominal surgery in diabetes patients | Research object duplication^330^ |
| T. E. Hassinger^331^ | 2018 | Acute Kidney Injury in the Age of Enhanced Recovery Protocols | Research object duplication^332^ |
| J. Gameiro^333^ | 2018 | Neutrophil, lymphocyte and platelet ratio as a predictor of postoperative acute kidney injury in major abdominal surgery | Research object duplication^334^ |
| STARSurg Collaborative^335^ | 2020 | Perioperative intravenous contrast administration and the incidence of acute kidney injury after major gastrointestinal surgery: prospective, multicentre cohort study | Research object duplication^336^ |
| M. Y. Argalious^337^ | 2017 | Association of body mass index and postoperative acute kidney injury in patients undergoing laparoscopic surgery | Research object duplication^321^ |
| T. N. Weingarten^338^ | 2011 | Nonalcoholic steatohepatitis (NASH) does not increase complications after laparoscopic bariatric surgery | Research object duplication^339^ |
| Joana Gameiro^340^ | 2020 | Transient and Persistent AKI and Outcomes in Patients Undergoing Major Abdominal Surgery | Research object duplication^334^ |
| M. Xie^341^ | 2020 | Acute kidney injury diagnosed by elevated serum creatinine increases mortality in ICU patients following non-cardiac surgery | Research object duplication^342^ |
| R. A. de Moura Pedro^343^ | 2025 | Postoperative oliguria after intermediate and high-risk surgeries in critical care, A cohort analysis | Data on AKI not available for the abdominal surgery |
| B. C. Zhao^344^ | 2024 | Postoperative haemoglobin and anaemia-associated ischaemic events after major noncardiac surgery: A sex-stratified cohort study | Data on AKI not available for the abdominal surgery |
| T. Yagyu^345^ | 2024 | Impact of the Diverting Stoma on Renal Function | Data on AKI not available for the abdominal surgery |
| H. B. Wubet^346^ | 2024 | The incidence and factors associated with anemia in elective surgical patients admitted to a surgical intensive care unit: a retrospective cohort study | Data on AKI not available for the abdominal surgery |
| J. P. Walco^347^ | 2024 | Association between Preoperative Blood Pressures and Postoperative Adverse Events | Data on AKI not available for the abdominal surgery |
| S. Paredes^348^ | 2024 | Metformin Use in Type 2 Diabetics and Delirium After Noncardiac Surgery: A Retrospective Cohort Analysis | Data on AKI not available for the abdominal surgery |
| M. Meersch^349^ | 2024 | Acute kidney disease beyond day 7 after major surgery: a secondary analysis of the EPIS-AKI trial | Data on AKI not available for the abdominal surgery |
| M. Kobata^350^ | 2024 | Associations between intraoperative nociceptive response index and early postoperative acute kidney injury in patients undergoing non-cardiac surgery under general anesthesia: a single-center retrospective cohort study | Data on AKI not available for the abdominal surgery |
| J. Y. Jung^351^ | 2024 | Association between intraoperative tidal volume and postoperative acute kidney injury in non-cardiac surgical patients using a propensity score-weighted analysis | Data on AKI not available for the abdominal surgery |
| J. Wu^352^ | 2023 | Optimization of central venous pressure during the perioperative period is associated with improved prognosis of high-risk operation patients | Data on AKI not available for the abdominal surgery |
| J. Wang^353^ | 2023 | Association of Preoperative Neutrophil-to-Lymphocyte Ratio with Postoperative Acute Kidney Injury and Mortality Following Major Noncardiac Surgeries | Data on AKI not available for the abdominal surgery |
| Q. Sun^354^ | 2023 | Risk factors of perioperative acute kidney injury in elderly patients: a single-center retrospective study | Data on AKI not available for the abdominal surgery |
| G. J. Solares^355^ | 2023 | Real-world outcomes of the hypotension prediction index in the management of intraoperative hypotension during non-cardiac surgery: a retrospective clinical study | Data on AKI not available for the abdominal surgery |
| C. G. Ravetti^356^ | 2023 | Impact of bedside ultrasound to reduce the incidence of acute renal injury in high-risk surgical patients: a randomized clinical trial | Data on AKI not available for the abdominal surgery |
| C. H. Park^357^ | 2023 | Current status of nutritional provision and effects of nutritional support on the clinical outcomes of acute kidney injury requiring continuous renal replacement therapy in the surgical intensive care unit | Data on AKI not available for the abdominal surgery |
| A. N. Moneme^358^ | 2023 | Primary Care Physician Follow-Up and 30-Day Readmission After Emergency General Surgery Admissions | Data on AKI not available for the abdominal surgery |
| W. K. Huang^359^ | 2023 | Association between cumulative duration of deep anesthesia and postoperative acute kidney injury after noncardiac surgeries: a retrospective observational study | Data on AKI not available for the abdominal surgery |
| Y. Zhou^360^ | 2022 | Threshold heterogeneity of perioperative hemoglobin drop for acute kidney injury after noncardiac surgery: a propensity score weighting analysis | Data on AKI not available for the abdominal surgery |
| Y. Tang^361^ | 2022 | Preoperative Neutrophil-Lymphocyte Ratio for predicting surgery-related acute kidney injury in non-cardiac surgery patients under general anaesthesia: A retrospective cohort study | Data on AKI not available for the abdominal surgery |
| H. Strand^362^ | 2022 | Effectiveness of the advisory display SmartPilot® view in the assessment of anesthetic depth in low risk gynecological surgery patients: a randomized controlled trial | Data on AKI not available for the abdominal surgery |
| E. Schneck^363^ | 2022 | Delta-like canonical Notch ligand 1 is predictive for sepsis and acute kidney injury in surgical intensive care patients | Data on AKI not available for the abdominal surgery |
| M. J. Sarna^364^ | 2022 | Metabolic syndrome as a predictor of perioperative outcomes in primary bariatric surgery, a MBSAQIP survey | Data on AKI not available for the abdominal surgery |
| M. Park^365^ | 2022 | Renal injury from sevoflurane in noncardiac surgery: a retrospective cohort study | Data on AKI not available for the abdominal surgery |
| D. R. McIlroy^366^ | 2022 | Oxygen administration during surgery and postoperative organ injury: observational cohort study | Data on AKI not available for the abdominal surgery |
| N. Li^367^ | 2022 | Association between urine microscopy and severe acute kidney injury in critically ill patients following non-cardiac surgery: a prospective cohort study | Data on AKI not available for the abdominal surgery |
| L. A. Goeddel^368^ | 2022 | Association Between Left Ventricular Relative Wall Thickness and Acute Kidney Injury After Noncardiac Surgery | Data on AKI not available for the abdominal surgery |
| D. X. Chi^369^ | 2022 | Effects of postoperative urine protein levels on predicting acute kidney injury in critically ill patients undergoing non-cardiac surgery | Data on AKI not available for the abdominal surgery |
| D. Ariyarathna^370^ | 2022 | Intraoperative vasopressor use and early postoperative acute kidney injury in elderly patients undergoing elective noncardiac surgery | Data on AKI not available for the abdominal surgery |
| P. M. Wanner^371^ | 2021 | Targeting Higher Intraoperative Blood Pressures Does Not Reduce Adverse Cardiovascular Events Following Noncardiac Surgery | Data on AKI not available for the abdominal surgery |
| M. Nishimoto^372^ | 2021 | External Validation of a Prediction Model for Acute Kidney Injury Following Noncardiac Surgery | Data on AKI not available for the abdominal surgery |
| K. McArthur^373^ | 2021 | Trauma and nontrauma damage-control laparotomy: The difference is delirium (data from the Eastern Association for the Surgery of Trauma SLEEP-TIME multicenter trial) | Data on AKI not available for the abdominal surgery |
| P. Y. Hardy^374^ | 2021 | Impact of Preoperative Anemia on Outcomes of Enhanced Recovery Program After Colorectal Surgery: A Monocentric Retrospective Study | Data on AKI not available for the abdominal surgery |
| B. L. Han^375^ | 2020 | Effect of preoperative serum alanine aminotransferase and asparagine aminotransferase ratio on prognosis of patients with gastric cancer | Data on AKI not available for the abdominal surgery |
| D. S. C. Magalhães^376^ | 2019 | Analyzing the Impact of Bariatric Surgery in Kidney Function: a 2-Year Observational Study | Data on AKI not available for the abdominal surgery |
| G. Passo^377^ | 2017 | A Perioperative Clinical Pathway Can Dramatically Reduce Failure-to-rescue Rates After Cytoreductive Surgery for Peritoneal Carcinomatosis: A Retrospective Study of 666 Consecutive Cytoreductions | Data on AKI not available for the abdominal surgery |
| H. Masoomi^378^ | 2012 | Predictive factors of in-hospital mortality in colon and rectal surgery | Data on AKI not available for the abdominal surgery |
| H. Masoomi^379^ | 2012 | Predictive factors of early bowel obstruction in colon and rectal surgery: data from the Nationwide Inpatient Sample, 2006-2008 | Data on AKI not available for the abdominal surgery |
| J. Zhou Done^380^ | 2025 | Major Perioperative Bleeding in Patients on Dialysis Undergoing Nonelective Abdominal Surgeries | Did not use a consensus definition of AKI |
| N. J. Kelly^381^ | 2025 | Total Gastrectomy for Gastric Malignancy: Trends Over 15 Years in Major Morbidity, Mortality, and Patient Selection From The National Surgical Quality Improvement Program | Did not use a consensus definition of AKI |
| A. Elkbuli^382^ | 2025 | National Analysis of Clinical Outcomes Associated With Cirrhotic Blunt Trauma Patients Undergoing Emergency Laparotomy Versus Non-operative Management: A Propensity Case-Matched Analysis | Did not use a consensus definition of AKI |
| L. Weiser^383^ | 2024 | Unplanned hospital readmission after cholecystectomy in adults with cerebral palsy | Did not use a consensus definition of AKI |
| S. Roth^384^ | 2024 | Cardiovascular-Kidney-Metabolic Syndrome: Association with Adverse Events After Major Noncardiac Surgery | Did not use a consensus definition of AKI |
| K. Miyamoto^385^ | 2024 | Age-related changes in intraoperative mean values of nociceptive response in patients undergoing non-cardiac surgery under general anesthesia: A retrospective cohort study | Did not use a consensus definition of AKI |
| W. R. Lorenz^386^ | 2024 | Factors Associated With Respiratory Failure After Open Ventral Hernia Repair: An Evaluation of the NSQIP Database | Did not use a consensus definition of AKI |
| R. Liu^387^ | 2024 | Incidence and risk factors of postoperative delirium following hepatic resection: a retrospective national inpatient sample database study | Did not use a consensus definition of AKI |
| F. S. Jehan^388^ | 2024 | Does the Surgical Approach Affect the Incidence of Post-Hepatectomy Liver Failure in Cirrhotic Patients? An Analysis of the NSQIP Database | Did not use a consensus definition of AKI |
| A. Elkbuli^389^ | 2024 | Evaluating Mortality Outcomes, Transfusion Characteristics, and Risk Factors Associated With Cirrhotic Trauma Patients Undergoing Emergency Laparotomy Versus Non-Operative Management: A National Analysis | Did not use a consensus definition of AKI |
| E. Y. Brovman^390^ | 2024 | Relationship Between Newly Established Perioperative DNR Status and Perioperative Outcomes in the Elderly Population: A NSQIP Database Analysis | Did not use a consensus definition of AKI |
| E. A. Biesel^391^ | 2024 | Surgical complications requiring late surgical revisions after pancreatoduodenectomy increase postoperative morbidity and mortality | Did not use a consensus definition of AKI |
| R. Assaf^392^ | 2024 | Racial Disparities in Outcomes of Bariatric Surgery: An Analysis of 190,319 Patients | Did not use a consensus definition of AKI |
| J. J. Turcotte^393^ | 2024 | Open and Closed Approaches to Skin Closure After Nonelective Open Colorectal Operations | Did not use a consensus definition of AKI |
| P. Tanos^394^ | 2023 | SHARP risk score: A predictor of poor outcomes in adults admitted for emergency general surgery: A prospective cohort study | Did not use a consensus definition of AKI |
| S. Soltanizadeh^395^ | 2023 | Even minor alteration of plasma creatinine after open abdominal surgery is associated with 30-day mortality: A single-centre cohort study | Did not use a consensus definition of AKI |
| C. Saitta^396^ | 2023 | Development of a novel score (RENSAFE) to determine probability of acute kidney injury and renal functional decline post surgery: A multicenter analysis | Did not use a consensus definition of AKI |
| M. Rutegård^397^ | 2023 | Defunctioning loop ileostomy in anterior resection for rectal cancer and subsequent renal failure: nationwide population-based study | Did not use a consensus definition of AKI |
| M. Omar^398^ | 2023 | Risk factors of acute renal failure in patients with protective ileostomy after rectal cancer surgery | Did not use a consensus definition of AKI |
| J. J. Nicholson^399^ | 2023 | Perioperative outcomes in intermediate and high-risk patients after major surgery following introduction of a dedicated perioperative medicine team: A single centre cohort study | Did not use a consensus definition of AKI |
| L. R. Maurer^400^ | 2023 | Validation of the Al-based Predictive OpTimal Trees in Emergency Surgery Risk (POTTER) Calculator in Patients 65 Years and Older | Did not use a consensus definition of AKI |
| T. M. Connelly^401^ | 2023 | Outcomes of clostridioides difficile infection on inflammatory bowel disease patients undergoing colonic resection: A propensity score weighted NSQIP analysis | Did not use a consensus definition of AKI |
| F. Walther^402^ | 2022 | Relationships between multiple patient safety outcomes and healthcare and hospital-related risk factors in colorectal resection cases: cross-sectional evidence from a nationwide sample of 232 German hospitals | Did not use a consensus definition of AKI |
| D. Tessman^403^ | 2022 | Surgical Outcomes of Distal Pancreatectomy in Elderly Patients | Did not use a consensus definition of AKI |
| S. Sheriff^404^ | 2022 | Predictors of mortality within the first year post-hepatectomy for hepatocellular carcinoma | Did not use a consensus definition of AKI |
| C. E. Sharon^405^ | 2022 | Fourteen years of pancreatic surgery for malignancy among ACS-NSQIP centers: Trends in major morbidity and mortality | Did not use a consensus definition of AKI |
| R. Mihailov^406^ | 2022 | Nomogram for Prediction of Postoperative Morbidity in Patients with Colon Cancer Requiring Emergency Therapy | Did not use a consensus definition of AKI |
| W. T. Kassahun^407^ | 2022 | Assessing differences in surgical outcomes following emergency abdominal exploration for complications of elective surgery and high-risk primary emergencies | Did not use a consensus definition of AKI |
| K. Karamchandani^408^ | 2022 | Non-Cardiac Surgery | Did not use a consensus definition of AKI |
| L. V. Huckaby^409^ | 2022 | Accuracy of Risk Estimation for Surgeons Versus Risk Calculators in Emergency General Surgery | Did not use a consensus definition of AKI |
| S. Hamade^410^ | 2022 | Evaluation of the American College of Surgeons National Surgical Quality Improvement Program Risk Calculator to predict outcomes after hysterectomies | Did not use a consensus definition of AKI |
| C. Ellenberger^411^ | 2022 | Myocardial injury after major noncardiac surgery: A secondary analysis of a randomized controlled trial | Did not use a consensus definition of AKI |
| S. Curran^412^ | 2022 | The impact of hypoalbuminemia on postoperative outcomes after outpatient surgery: a national analysis of the NSQIP database | Did not use a consensus definition of AKI |
| M. S. Benk^413^ | 2022 | Evaluation of complications after laparoscopic and open appendectomy by the American College of Surgeons National Surgical Quality Improvement Program surgical risk calculator | Did not use a consensus definition of AKI |
| P. P. Argueta^414^ | 2022 | Thirty-Day Readmission After Bariatric Surgery: Causes, Effects on Outcomes, and Predictors | Did not use a consensus definition of AKI |
| R. Seishima^415^ | 2021 | Safety and feasibility of laparoscopic surgery for elderly rectal cancer patients in Japan: a nationwide study | Did not use a consensus definition of AKI |
| F. Peng^416^ | 2021 | Comparative study of short-term efficacy,effectiveness and safety at different stages of the laparoscopic pancreaticoduodenectomy learning curve | Did not use a consensus definition of AKI |
| J. B. Oliver^417^ | 2021 | The Impact of Chronic Liver Disease on Postoperative Outcomes and Resource Utilization | Did not use a consensus definition of AKI |
| S. Niemeläinen^418^ | 2021 | The Clinical Frailty Scale is a useful tool for predicting postoperative complications following elective colon cancer surgery at the age of 80 years and above: A prospective, multicentre observational study | Did not use a consensus definition of AKI |
| A. S. Mierzwa^419^ | 2021 | Characterizing Timing of Postoperative Complications Following Elective Roux-en-Y gastric Bypass and Sleeve Gastrectomy | Did not use a consensus definition of AKI |
| N. E. Kim^420^ | 2021 | Risk Factors for Readmission after Ileostomy Creation: an NSQIP Database Study | Did not use a consensus definition of AKI |
| M. El Edelbi^421^ | 2021 | Comparing Emergent and Elective Colectomy Outcomes in Elderly Patients: A NSQIP Study | Did not use a consensus definition of AKI |
| J. H. Wolf^422^ | 2020 | Preoperative Nutritional Status Predicts Major Morbidity After Primary Rectal Cancer Resection | Did not use a consensus definition of AKI |
| E. L. Vos^423^ | 2020 | Performance of the American College of Surgeons NSQIP Surgical Risk Calculator for Total Gastrectomy | Did not use a consensus definition of AKI |
| K. Sahara^424^ | 2020 | Evaluation of the ACS NSQIP Surgical Risk Calculator in Elderly Patients Undergoing Hepatectomy for Hepatocellular Carcinoma | Did not use a consensus definition of AKI |
| K. Kongpakwattana^425^ | 2020 | Clinical and economic burden of postsurgical complications of high-risk surgeries: a cohort study in Thailand | Did not use a consensus definition of AKI |
| H. M. A. Kaafarani^426^ | 2020 | Prospective validation of the Emergency Surgery Score in emergency general surgery: An Eastern Association for the Surgery of Trauma multicenter study | Did not use a consensus definition of AKI |
| S. M. Alzahrani^427^ | 2020 | Validation of the ACS NSQIP Surgical Risk Calculator for Patients with Early Gastric Cancer Treated with Laparoscopic Gastrectomy | Did not use a consensus definition of AKI |
| F. R. Montes^428^ | 2019 | Association between emergency department length of stay and adverse perioperative outcomes in emergency surgery: a cohort study in two Colombian University hospitals | Did not use a consensus definition of AKI |
| E. M. Gleeson^429^ | 2019 | Patient-specific predictors of failure to rescue after pancreaticoduodenectomy | Did not use a consensus definition of AKI |
| A. D. Geller^430^ | 2019 | Relative Incremental Cost of Postoperative Complications of Esophagectomy | Did not use a consensus definition of AKI |
| E. Chacon^431^ | 2019 | Effect of critical care complications on perioperative mortality and hospital length of stay after hepatectomy: A multicenter analysis of 21,443 patients | Did not use a consensus definition of AKI |
| P. Sharma^432^ | 2018 | Impact of Bariatric Surgery on Outcomes of Patients with Inflammatory Bowel Disease: a Nationwide Inpatient Sample Analysis, 2004-2014 | Did not use a consensus definition of AKI |
| F. Schlottmann^433^ | 2018 | Surgery for benign esophageal disorders in the US: risk factors for complications and trends of morbidity | Did not use a consensus definition of AKI |
| R. Ramanathan^434^ | 2018 | Predictors of Short-Term Readmission After Pancreaticoduodenectomy | Did not use a consensus definition of AKI |
| T. R. McCarty^435^ | 2018 | Impact of bariatric surgery on outcomes of patients with nonalcoholic fatty liver disease: a nationwide inpatient sample analysis, 2004-2012 | Did not use a consensus definition of AKI |
| R. Fletcher^436^ | 2018 | Predictors of Increased Length of Hospital Stay Following Laparoscopic Sleeve Gastrectomy from the National Surgical Quality Improvement Program | Did not use a consensus definition of AKI |
| M. El Amrani^437^ | 2018 | Failure-to-rescue in Patients Undergoing Pancreatectomy: Is Hospital Volume a Standard for Quality Improvement Programs? Nationwide Analysis of 12,333 Patients | Did not use a consensus definition of AKI |
| J. B. Ammori^438^ | 2018 | Predictors of 30-day readmissions after gastrectomy for malignancy | Did not use a consensus definition of AKI |
| M. D. Whealon^439^ | 2017 | Volume and outcomes relationship in laparoscopic diaphragmatic hernia repair | Did not use a consensus definition of AKI |
| T. Watanabe^440^ | 2017 | Prediction model for complications after low anterior resection based on data from 33,411 Japanese patients included in the National Clinical Database | Did not use a consensus definition of AKI |
| J. B. Wang^441^ | 2017 | gastric cancer after laparoscopy-assisted total gastrectomy: results from an 8-year experience at a large-scale single center | Did not use a consensus definition of AKI |
| H. Kikuchi^442^ | 2017 | Development and external validation of preoperative risk models for operative morbidities after total gastrectomy using a Japanese web-based nationwide registry | Did not use a consensus definition of AKI |
| R. Gupta^443^ | 2017 | Impact of intraoperative blood loss on the short-term outcomes of laparoscopic liver resection | Did not use a consensus definition of AKI |
| H. Yokoo^444^ | 2016 | Models predicting the risks of six life-threatening morbidities and bile leakage in 14,970 hepatectomy patients registered in the National Clinical Database of Japan | Did not use a consensus definition of AKI |
| C. Rivard^445^ | 2016 | Evaluation of the performance of the ACS NSQIP surgical risk calculator in gynecologic oncology patients undergoing laparotomy | Did not use a consensus definition of AKI |
| T. E. Newhook^446^ | 2016 | Morbidity and mortality of hepatectomy for benign liver tumors | Did not use a consensus definition of AKI |
| A. C. Murray^447^ | 2016 | 30-day mortality after elective colorectal surgery can reasonably be predicted | Did not use a consensus definition of AKI |
| H. Ichikawa^448^ | 2016 | Surgical and long-term outcomes following oesophagectomy in oesophageal cancer patients with comorbidity | Did not use a consensus definition of AKI |
| L. Doyon^449^ | 2016 | Resident participation in laparoscopic Roux-en-Y gastric bypass: a comparison of outcomes from the ACS-NSQIP database | Did not use a consensus definition of AKI |
| A. R. Chang^450^ | 2016 | Bariatric surgery is associated with improvement in kidney outcomes | Did not use a consensus definition of AKI |
| G. Alsfasser^451^ | 2016 | Volume-outcome relationship in pancreatic surgery | Did not use a consensus definition of AKI |
| J. B. Szender^452^ | 2015 | Evaluation of the National Surgical Quality Improvement Program Universal Surgical Risk Calculator for a gynecologic oncology service | Did not use a consensus definition of AKI |
| J. Kenig^453^ | 2015 | The prognostic role of comorbidities in older patients qualified for emergency abdominal surgery | Did not use a consensus definition of AKI |
| B. Joseph^454^ | 2015 | Factors associated with failure-to-rescue in patients undergoing trauma laparotomy | Did not use a consensus definition of AKI |
| C. Huang^455^ | 2015 | Postoperative 30-day mortality may underestimate the risk of esophagectomy | Did not use a consensus definition of AKI |
| M. L. Friedell^456^ | 2015 | β-Blockade and Operative Mortality in Noncardiac Surgery: Harmful or Helpful? | Did not use a consensus definition of AKI |
| A. Sako^457^ | 2014 | Prevalence and in-hospital mortality of gastrostomy and jejunostomy in Japan: a retrospective study with a national administrative database | Did not use a consensus definition of AKI |
| H. Mahdi^458^ | 2014 | Predictors of surgical site infection in women undergoing hysterectomy for benign gynecologic disease: a multicenter analysis using the national surgical quality improvement program data | Did not use a consensus definition of AKI |
| M. Huebner^459^ | 2014 | Timing of complications and length of stay after rectal cancer surgery | Did not use a consensus definition of AKI |
| R. Ricciardi^460^ | 2013 | Which adverse events are associated with mortality and prolonged length of stay following colorectal surgery? | Did not use a consensus definition of AKI |
| A. Nimeri^461^ | 2013 | Are results of bariatric surgery different in the Middle East? Early experience of an international bariatric surgery program and an ACS NSQIP outcomes comparison | Did not use a consensus definition of AKI |
| K. Nandipati^462^ | 2013 | Factors predicting the increased risk for return to the operating room in bariatric patients: a NSQIP database study | Did not use a consensus definition of AKI |
| C. Y. Kang^463^ | 2013 | Risk factors for anastomotic leakage after anterior resection for rectal cancer | Did not use a consensus definition of AKI |
| K. Hardiman^464^ | 2013 | Laparoscopic colectomy reduces morbidity and mortality in obese patients | Did not use a consensus definition of AKI |
| S. G. de la Fuente^465^ | 2013 | Functional status determines postoperative outcomes in elderly patients undergoing hepatic resections | Did not use a consensus definition of AKI |
| A. D. Shaw^466^ | 2012 | Major complications, mortality, and resource utilization after open abdominal surgery: 0.9% saline compared to Plasma-Lyte | Did not use a consensus definition of AKI |
| E. Finlayson^467^ | 2012 | Outcomes after rectal cancer surgery in elderly nursing home residents | Did not use a consensus definition of AKI |
| M. M. Cone^468^ | 2012 | Effect of surgical approach on 30-day mortality and morbidity after elective colectomy: a NSQIP study | Did not use a consensus definition of AKI |
| S. W. Leichtle^469^ | 2011 | Does preoperative anemia adversely affect colon and rectal surgery outcomes? | Did not use a consensus definition of AKI |
| J. E. Carroll^470^ | 2010 | In-hospital mortality after resection of biliary tract cancer in the United States | Did not use a consensus definition of AKI |
| R. P. Merkow^471^ | 2009 | Effect of body mass index on short-term outcomes after colectomy for cancer | Did not use a consensus definition of AKI |
| T. Kusano^472^ | 2009 | Predictors and prognostic significance of operative complications in patients with hepatocellular carcinoma who underwent hepatic resection | Did not use a consensus definition of AKI |
| T. Almahmeed^473^ | 2007 | Morbidity of anastomotic leaks in patients undergoing Roux-en-Y gastric bypass | Did not use a consensus definition of AKI |
| R. A. Schroeder^474^ | 2006 | Predictive indices of morbidity and mortality after liver resection | Did not use a consensus definition of AKI |
| P. A. McCullough^475^ | 2006 | Cardiorespiratory fitness and short-term complications after bariatric surgery | Did not use a consensus definition of AKI |

REFERENCE

1 Khanna, A. K. *et al.* Postoperative Hypotension and Adverse Clinical Outcomes in Patients Without Intraoperative Hypotension, After Noncardiac Surgery. *Anesth Analg* **132**, 1410-1420, doi:10.1213/ane.0000000000005374 (2021).

2 Johnston, S. S. *et al.* Association of In-Hospital Surgical Bleeding Events with Prolonged Hospital Length of Stay, Days Spent in Critical Care, Complications, and Mortality: A Retrospective Cohort Study Among Patients Undergoing Neoplasm-Directed Surgeries in English Hospitals. *Clinicoecon Outcomes Res* **13**, 19-29, doi:10.2147/ceor.S287970 (2021).

3 Hadi, Y. B., Naqvi, S. F. Z., Kupec, J. T., Sofka, S. & Sarwari, A. Outcomes of COVID-19 in Solid Organ Transplant Recipients: A Propensity-matched Analysis of a Large Research Network. *Transplantation* **105**, 1365-1371, doi:10.1097/tp.0000000000003670 (2021).

4 Engel, D., Löffel, L. M., Wuethrich, P. Y. & Hahn, R. G. Preoperative Concentrated Urine Increases the Incidence of Plasma Creatinine Elevation After Major Surgery. *Frontiers in Medicine* **8**, doi:10.3389/fmed.2021.699969 (2021).

5 Carpio, J. D. *et al.* Development and validation of a model to predict severe hospital‐acquired acute kidney injury in non‐critically ill patients. *Journal of Clinical Medicine* **10**, doi:10.3390/jcm10173959 (2021).

6 Bang, J. Y. *et al.* Impact of Sarcopenia on Acute Kidney Injury after Infrarenal Abdominal Aortic Aneurysm Surgery: A Propensity Matching Analysis. *Nutrients* **13**, doi:10.3390/nu13072212 (2021).

7 Alvarado, M., Schaubel, D. E., Reddy, K. R. & Bittermann, T. Black Race Is Associated With Higher Rates of Early-Onset End-Stage Renal Disease and Increased Mortality Following Liver Transplantation. *Liver Transpl* **27**, 1154-1164, doi:10.1002/lt.26054 (2021).

8 Bhatia, R., Fabes, J., Krzanicki, D., Rahman, S. & Spiro, M. Association between fast-track extubation after orthotopic liver transplant, postoperative vasopressor requirement, and acute kidney injury. *Experimental and Clinical Transplantation* **19**, 339-344, doi:10.6002/ect.2020.0422 (2021).

9 Abeysekera, N. *et al.* Evaluating the need for an integrated geriatric service in older general surgery patients. *ANZ J Surg* **91**, 341-347, doi:10.1111/ans.16612 (2021).

10 Vongsumran, N., Buranapin, S. & Manosroi, W. Standardized glycemic management versus conventional glycemic management and postoperative outcomes in type 2 diabetes patients undergoing elective surgery. *Diabetes, Metabolic Syndrome and Obesity: Targets and Therapy* **13**, 2593-2601, doi:10.2147/DMSO.S262444 (2020).

11 van Ommeren-Olijve, S. J., Burbach, J. P. M. & Furnée, E. J. B. Risk factors for non-closure of an intended temporary defunctioning stoma after emergency resection of left-sided obstructive colon cancer. *Int J Colorectal Dis* **35**, 1087-1093, doi:10.1007/s00384-020-03559-1 (2020).

12 Ruetzler, K. *et al.* Supplemental Intraoperative Oxygen Does Not Promote Acute Kidney Injury or Cardiovascular Complications After Noncardiac Surgery: Subanalysis of an Alternating Intervention Trial. *Anesth Analg* **130**, 933-940, doi:10.1213/ane.0000000000004359 (2020).

13 Park, S. *et al.* Intraoperative Arterial Pressure Variability and Postoperative Acute Kidney Injury. *Clin J Am Soc Nephrol* **15**, 35-46, doi:10.2215/cjn.06620619 (2020).

14 Nicklas, J. Y. *et al.* Personalised haemodynamic management targeting baseline cardiac index in high-risk patients undergoing major abdominal surgery: a randomised single-centre clinical trial. *British Journal of Anaesthesia* **125**, 122-132, doi:10.1016/j.bja.2020.04.094 (2020).

15 Nct. Role of Blood Management in Perioperative Outcomes. [*https://clinicaltrials.gov/show/NCT04475497*](https://clinicaltrials.gov/show/NCT04475497) (2020).

16 Nct. Necessity of Preoperative Empirical Antibiotic Use in Acute Cholecystitis. [*https://clinicaltrials.gov/show/NCT04661371*](https://clinicaltrials.gov/show/NCT04661371) (2020).

17 Nct. Laparoscopic Cholecystectomy is no More Risky in Emergent Cases With in Cardiopulmonary Risk: fundus-Callot Cholecystectomy With Low Pressure Pneumo-peritoneum VS Open Cholecystectomy. [*https://clinicaltrials.gov/show/NCT04373447*](https://clinicaltrials.gov/show/NCT04373447) (2020).

18 Miyake, K. *et al.* Association of pre-operative chronic kidney disease and acute kidney injury with in-hospital outcomes of emergency colorectal surgery: a cohort study. *World J Emerg Surg* **15**, 22, doi:10.1186/s13017-020-00303-6 (2020).

19 Mathis, M. R. *et al.* Preoperative Risk and the Association between Hypotension and Postoperative Acute Kidney Injury. *Anesthesiology* **132**, 461-475, doi:10.1097/aln.0000000000003063 (2020).

20 Maheshwari, K. *et al.* Saline versus Lactated Ringer's Solution: The Saline or Lactated Ringer's (SOLAR) Trial. *Anesthesiology* **132**, 614-624, doi:10.1097/aln.0000000000003130 (2020).

21 Lysak, N. *et al.* Cardiovascular death and progression to end-stage renal disease after major surgery in elderly patients. *BJS Open* **4**, 145-156, doi:10.1002/bjs5.50232 (2020).

22 Ingraham, N. E., Tignanelli, C. J., Menk, J. & Chipman, J. G. Pre- and Peri-Operative Factors Associated with Chronic Critical Illness in Liver Transplant Recipients. *Surg Infect (Larchmt)* **21**, 246-254, doi:10.1089/sur.2019.192 (2020).

23 Andrianello, S. *et al.* Pancreaticojejunostomy With Externalized Stent vs Pancreaticogastrostomy With Externalized Stent for Patients With High-Risk Pancreatic Anastomosis: a Single-Center, Phase 3, Randomized Clinical Trial. *JAMA surgery* **155**, 313‐321, doi:10.1001/jamasurg.2019.6035 (2020).

24 Yang, S. *et al.* Clinical features and outcomes of patients with acute mesenteric ischemia and concomitant colon ischemia: a retrospective cohort study. *Journal of Surgical Research* **233**, 231-239, doi:10.1016/j.jss.2018.08.010 (2019).

25 Tang, Y. *et al.* Association of Intraoperative Hypotension with Acute Kidney Injury after Noncardiac Surgery in Patients Younger than 60 Years Old. *Kidney Blood Press Res* **44**, 211-221, doi:10.1159/000498990 (2019).

26 Szabó, M., Bozó, A., Darvas, K., Horváth, A. & Iványi, Z. D. Role of inferior vena cava collapsibility index in the prediction of hypotension associated with general anesthesia: an observational study. *BMC Anesthesiol* **19**, 139, doi:10.1186/s12871-019-0809-4 (2019).

27 Park, S. *et al.* Simple Postoperative AKI Risk (SPARK) Classification before Noncardiac Surgery: A Prediction Index Development Study with External Validation. *J Am Soc Nephrol* **30**, 170-181, doi:10.1681/asn.2018070757 (2019).

28 Oh, T. K., Song, I. A. & Jeon, Y. T. Admission to the surgical intensive care unit during intensivist coverage is associated with lower incidence of postoperative acute kidney injury and shorter ventilator time. *J Anesth* **33**, 647-655, doi:10.1007/s00540-019-02684-8 (2019).

29 Oh, T. K., Song, I. A., Do, S. H., Jheon, S. & Lim, C. Association of perioperative weight-based fluid balance with 30-day mortality and acute kidney injury among patients in the surgical intensive care unit. *J Anesth* **33**, 354-363, doi:10.1007/s00540-019-02630-8 (2019).

30 Oh, T. K. *et al.* Association of Preoperative Serum Chloride Levels With Mortality and Morbidity After Noncardiac Surgery: A Retrospective Cohort Study. *Anesth Analg* **129**, 1494-1501, doi:10.1213/ane.0000000000003958 (2019).

31 MacDonald, N. *et al.* The role of goal-directed therapy in the prevention of acute kidney injury after major gastrointestinal surgery: Substudy of the OPTIMISE trial. *Eur J Anaesthesiol* **36**, 924-932, doi:10.1097/eja.0000000000001104 (2019).

32 Lei, V. J. *et al.* Risk Stratification for Postoperative Acute Kidney Injury in Major Noncardiac Surgery Using Preoperative and Intraoperative Data. *JAMA Netw Open* **2**, e1916921, doi:10.1001/jamanetworkopen.2019.16921 (2019).

33 Kougias, P., Sharath, S., Mi, Z., Biswas, K. & Mills, J. L. Effect of Postoperative Permissive Anemia and Cardiovascular Risk Status on Outcomes After Major General and Vascular Surgery Operative Interventions. *Ann Surg* **270**, 602-611, doi:10.1097/sla.0000000000003525 (2019).

34 Kim, B. J. *et al.* Contemporary analysis of complications associated with biliary stents during neoadjuvant therapy for pancreatic adenocarcinoma. *HPB* **21**, 662-668, doi:10.1016/j.hpb.2018.10.009 (2019).

35 Hung, K. C. *et al.* Impact of Preoperative Anemia on Postoperative Kidney Function Following Laparoscopic Bariatric Surgery. *Obes Surg* **29**, 2527-2534, doi:10.1007/s11695-019-03869-6 (2019).

36 Adhikari, L. *et al.* Improved predictive models for acute kidney injury with IDEA: Intraoperative Data Embedded Analytics. *PLoS One* **14**, e0214904, doi:10.1371/journal.pone.0214904 (2019).

37 Zealley, I., Wang, H., Donnan, P. T. & Bell, S. Exposure to contrast media in the perioperative period confers no additional risk of acute kidney injury in surgical patients. *Nephrol Dial Transplant* **33**, 1751-1756, doi:10.1093/ndt/gfx325 (2018).

38 Weinberg, L. *et al.* Associations of fluid amount, type, and balance and acute kidney injury in patients undergoing major surgery. *Anaesth Intensive Care* **46**, 79-87, doi:10.1177/0310057x1804600112 (2018).

39 Shiba, A., Uchino, S., Fujii, T., Takinami, M. & Uezono, S. Association Between Intraoperative Oliguria and Acute Kidney Injury After Major Noncardiac Surgery. *Anesth Analg* **127**, 1229-1235, doi:10.1213/ane.0000000000003576 (2018).

40 Sakan, S., Premuzic, V., Bandic Pavlovic, D. & Basic-Jukic, N. Consequence of Elevated Fibroblast Growth Factor 23 Levels in Acute Kidney Injury, Renal Recovery and Overall Survival in Intensive Care Unit Patients After Major Surgery. *Ther Apher Dial* **22**, 544-551, doi:10.1111/1744-9987.12703 (2018).

41 Park, S. *et al.* Awareness, incidence and clinical significance of acute kidney injury after non-general anesthesia: A retrospective cohort study. *Medicine (Baltimore)* **97**, e12014, doi:10.1097/md.0000000000012014 (2018).

42 Oh, T. K. *et al.* Hyperchloremia and postoperative acute kidney injury: a retrospective analysis of data from the surgical intensive care unit. *Crit Care* **22**, 277, doi:10.1186/s13054-018-2216-5 (2018).

43 Xu, N. *et al.* Association between preoperative renin-angiotensin system inhibitor use and postoperative acute kidney injury risk in patients with hypertension. *Clinical Nephrology* **89**, 403-412, doi:10.5414/CN109319 (2018).

44 Mzoughi, Z. *et al.* Laparoscopy for perforated duodenal ulcer: A morbidity score based on a cohort study of 384 patients. *Tunis Med* **96**, 167-170 (2018).

45 Jia, H. M. *et al.* Derivation and validation of plasma endostatin for predicting renal recovery from acute kidney injury: a prospective validation study. *Crit Care* **22**, 305, doi:10.1186/s13054-018-2232-5 (2018).

46 Hinck, B. D. *et al.* Urine kidney injury markers do not increase following gastric bypass: a multi-center cross-sectional study. *Can J Urol* **25**, 9199-9204 (2018).

47 Dagel, T. *et al.* Hyperthermic intraperitonal chemotherapy is an independent risk factor for development of acute kidney injury. *J buon* **23**, 1528-1533 (2018).

48 Bressan, A. K. *et al.* Efficacy of a Dual-ring Wound Protector for Prevention of Surgical Site Infections After Pancreaticoduodenectomy in Patients With Intrabiliary Stents: a Randomized Clinical Trial. *Annals of surgery* **268**, 35‐40, doi:10.1097/SLA.0000000000002614 (2018).

49 Zhao, X. J., Zhu, F. X., Li, S., Zhang, H. B. & An, Y. Z. Acute kidney injury is an independent risk factor for myocardial injury after noncardiac surgery in critical patients. *J Crit Care* **39**, 225-231, doi:10.1016/j.jcrc.2017.01.011 (2017).

50 Sun, Y. L. *et al.* Total closure of pancreatic section for end-to-side pancreaticojejunostomy decreases incidence of pancreatic fistula in pancreaticoduodenectomy. *Hepatobiliary & pancreatic diseases international* **16**, 310‐314, doi:10.1016/s1499-3872(17)60010-9 (2017).

51 Spadaro, S. *et al.* The effects of storage of red blood cells on the development of postoperative infections after noncardiac surgery. *Transfusion* **57**, 2727-2737, doi:10.1111/trf.14249 (2017).

52 Rencuzogullari, A., Benlice, C., Costedio, M., Remzi, F. H. & Gorgun, E. Nomogram-derived prediction of postoperative ileus after colectomy: An assessment from nationwide procedure-targeted cohort. *American Surgeon* **83**, 564-572 (2017).

53 Puckett, J. R. *et al.* Low Versus Standard Urine Output Targets in Patients Undergoing Major Abdominal Surgery: A Randomized Noninferiority Trial. *Ann Surg* **265**, 874-881, doi:10.1097/sla.0000000000002044 (2017).

54 Özcan, H. Ç., Uğur, M. G., Balat, Ö., Bayramoğlu Tepe, N. & Sucu, S. Emergency peripartum hysterectomy: single center ten-year experience. *Journal of Maternal-Fetal and Neonatal Medicine* **30**, 2778-2783, doi:10.1080/14767058.2016.1263293 (2017).

55 Olufajo, O. A. *et al.* Preoperative assessment of surgical risk: creation of a scoring tool to estimate 1-year mortality after emergency abdominal surgery in the elderly patient. *Am J Surg* **213**, 771-777.e771, doi:10.1016/j.amjsurg.2016.08.007 (2017).

56 Ferrada, P. *et al.* Loop ileostomy versus total colectomy as surgical treatment for Clostridium difficile-associated disease: An Eastern Association for the Surgery of Trauma multicenter trial. *J Trauma Acute Care Surg* **83**, 36-40, doi:10.1097/ta.0000000000001498 (2017).

57 Branch-Elliman, W. *et al.* Risk of surgical site infection, acute kidney injury, and Clostridium difficile infection following antibiotic prophylaxis with vancomycin plus a beta-lactam versus either drug alone: A national propensity-score-adjusted retrospective cohort study. *PLoS Medicine* **14**, doi:10.1371/journal.pmed.1002340 (2017).

58 Behman, R. *et al.* Laparoscopic Surgery for Adhesive Small Bowel Obstruction Is Associated with a Higher Risk of Bowel Injury: A Population-based Analysis of 8584 Patients. *Annals of Surgery* **266**, 489-498, doi:10.1097/SLA.0000000000002369 (2017).

59 Zimmerman, A. M. *et al.* Preoperative Myocardial Injury as a Predictor of Mortality in Emergency General Surgery: An Analysis Using the American College of Surgeons NSQIP Database. *J Am Coll Surg* **223**, 381-386, doi:10.1016/j.jamcollsurg.2016.04.043 (2016).

60 Vijay, A., Grover, A., Coulson, T. G. & Myles, P. S. Perioperative management of patients treated with angiotensin-converting enzyme inhibitors and angiotensin II receptor blockers: a quality improvement audit. *Anaesth Intensive Care* **44**, 346-352, doi:10.1177/0310057x1604400305 (2016).

61 Thottakkara, P. *et al.* Application of Machine Learning Techniques to High-Dimensional Clinical Data to Forecast Postoperative Complications. *PLoS One* **11**, e0155705, doi:10.1371/journal.pone.0155705 (2016).

62 Pisitsak, C. *et al.* Prevalence, Outcomes and Risk factors of Acute Kidney Injury in Surgical Intensive Care Unit: A Multi-Center Thai University-Based Surgical Intensive Care Units Study (THAI-SICU Study). *J Med Assoc Thai* **99 Suppl 6**, S193-s200 (2016).

63 Trongtrakul, K. *et al.* Acute kidney injury risk prediction score for critically-ill surgical patients. *BMC Anesthesiol* **20**, 140, doi:10.1186/s12871-020-01046-2 (2020).

64 Oprea, A. D. *et al.* Baseline Pulse Pressure, Acute Kidney Injury, and Mortality After Noncardiac Surgery. *Anesth Analg* **123**, 1480-1489, doi:10.1213/ane.0000000000001557 (2016).

65 Liu, X. *et al.* A Predictive Model for Assessing Surgery-Related Acute Kidney Injury Risk in Hypertensive Patients: A Retrospective Cohort Study. *PLoS One* **11**, e0165280, doi:10.1371/journal.pone.0165280 (2016).

66 Korenkevych, D. *et al.* The Pattern of Longitudinal Change in Serum Creatinine and 90-Day Mortality After Major Surgery. *Ann Surg* **263**, 1219-1227, doi:10.1097/sla.0000000000001362 (2016).

67 Huber, M. *et al.* Mortality and Cost of Acute and Chronic Kidney Disease after Vascular Surgery. *Ann Vasc Surg* **30**, 72-81.e71-72, doi:10.1016/j.avsg.2015.04.092 (2016).

68 Cauchy, F. *et al.* Incidence, risk factors and consequences of bile leakage following laparoscopic major hepatectomy. *Surgical Endoscopy* **30**, 3709-3719, doi:10.1007/s00464-015-4666-z (2016).

69 Chang, C. C. *et al.* Impact of Peri-Operative Anemia and Blood Transfusions in Patients with Gastric Cancer Receiving Gastrectomy. *Asian Pac J Cancer Prev* **17**, 1427-1431, doi:10.7314/apjcp.2016.17.3.1427 (2016).

70 Bushyhead, D., Kirkpatrick, J. N. & Goldberg, D. Pretransplant echocardiographic parameters as markers of posttransplant outcomes in liver transplant recipients. *Liver Transpl* **22**, 316-323, doi:10.1002/lt.24375 (2016).

71 Bardia, A. *et al.* Combined Epidural-General Anesthesia vs General Anesthesia Alone for Elective Abdominal Aortic Aneurysm Repair. *JAMA Surg* **151**, 1116-1123, doi:10.1001/jamasurg.2016.2733 (2016).

72 Barakat, H. M., Shahin, Y., Khan, J. A., McCollum, P. T. & Chetter, I. C. Preoperative supervised exercise improves outcomes after elective abdominal aortic aneurysm repair. *Annals of Surgery* **264**, 47-53, doi:10.1097/SLA.0000000000001609 (2016).

73 Andert, A. *et al.* Liver Transplantation and Donor Body Mass Index >30: Use or Refuse? *Ann Transplant* **21**, 185-193, doi:10.12659/aot.896101 (2016).

74 Wise, K. B. *et al.* Proximal intestinal diversion is associated with increased morbidity in patients undergoing elective colectomy for diverticular disease: an ACS-NSQIP study. *J Gastrointest Surg* **19**, 535-542, doi:10.1007/s11605-014-2700-4 (2015).

75 Weick, A. *et al.* Incidence of cardiovascular and cerebrovascular events associated with sirolimus use after liver transplantation. *Transplantation Proceedings* **47**, 460-464, doi:10.1016/j.transproceed.2014.11.036 (2015).

76 Saze, Z. *et al.* Risk Models of Operative Morbidities in 16,930 Critically Ill Surgical Patients Based on a Japanese Nationwide Database. *Medicine (Baltimore)* **94**, e1224, doi:10.1097/md.0000000000001224 (2015).

77 Moghadamyeghaneh, Z., Carmichael, J. C., Mills, S. D., Pigazzi, A. & Stamos, M. J. Effects of ascites on outcomes of colorectal surgery in congestive heart failure patients. *Am J Surg* **209**, 1020-1027, doi:10.1016/j.amjsurg.2014.08.021 (2015).

78 Newhook, T. E. *et al.* Impact of Postoperative Venous Thromboembolism on Postoperative Morbidity, Mortality, and Resource Utilization after Hepatectomy. *Am Surg* **81**, 1216-1223 (2015).

79 Longenecker, J. C., Estrella, M. M., Segev, D. L. & Atta, M. G. Patterns of Kidney Function Before and After Orthotopic Liver Transplant: Associations With Length of Hospital Stay, Progression to End-Stage Renal Disease, and Mortality. *Transplantation* **99**, 2556-2564, doi:10.1097/tp.0000000000000767 (2015).

80 Kashani, K. *et al.* Vascular Surgery Kidney Injury Predictive Score: A Historical Cohort Study. *J Cardiothorac Vasc Anesth* **29**, 1588-1595, doi:10.1053/j.jvca.2015.04.013 (2015).

81 Huang, W. *et al.* Value of Neutrophil Counts in Predicting Surgery-Related Acute Kidney Injury and the Interaction of These Counts With Diabetes in Chronic Kidney Disease Patients With Hypertension: A Cohort Study. *Medicine (Baltimore)* **94**, e1780, doi:10.1097/md.0000000000001780 (2015).

82 Hobson, C. *et al.* Cost and Mortality Associated With Postoperative Acute Kidney Injury. *Ann Surg* **261**, 1207-1214, doi:10.1097/sla.0000000000000732 (2015).

83 Gocze, I. *et al.* Urinary biomarkers TIMP-2 and IGFBP7 early predict acute kidney injury after major surgery. *PLoS One* **10**, e0120863, doi:10.1371/journal.pone.0120863 (2015).

84 Giannella, M. *et al.* High-dose weekly liposomal amphotericin b antifungal prophylaxis in patients undergoing liver transplantation: a prospective phase II trial. *Transplantation* **99**, 848-854, doi:10.1097/tp.0000000000000393 (2015).

85 Chou, C. L. *et al.* Adverse outcomes after major surgery in patients with pressure ulcer: a nationwide population-based retrospective cohort study. *PLoS One* **10**, e0127731, doi:10.1371/journal.pone.0127731 (2015).

86 Chen, H. P., Tsai, Y. F., Lin, J. R., Liu, F. C. & Yu, H. P. Incidence and Outcomes of Acute Renal Failure Following Liver Transplantation: A Population-Based Cohort Study. *Medicine (Baltimore)* **94**, e2320, doi:10.1097/md.0000000000002320 (2015).

87 Tsai, M. S., Lin, C. L., Chang, S. N., Lee, P. H. & Kao, C. H. Diabetes mellitus and increased postoperative risk of acute renal failure after hepatectomy for hepatocellular carcinoma: a nationwide population-based study. *Ann Surg Oncol* **21**, 3810-3816, doi:10.1245/s10434-014-3777-4 (2014).

88 Spolverato, G., Ejaz, A., Hyder, O., Kim, Y. & Pawlik, T. M. Failure to rescue as a source of variation in hospital mortality after hepatic surgery. *Br J Surg* **101**, 836-846, doi:10.1002/bjs.9492 (2014).

89 Shah, M. *et al.* Association between angiotensin converting enzyme inhibitor or angiotensin receptor blocker use prior to major elective surgery and the risk of acute dialysis. *BMC Nephrol* **15**, 53, doi:10.1186/1471-2369-15-53 (2014).

90 Ruebner, R. L., Reese, P. P. & Abt, P. L. Donation after cardiac death liver transplantation is associated with increased risk of end-stage renal disease. *Transpl Int* **27**, 1263-1271, doi:10.1111/tri.12409 (2014).

91 Pan, X. *et al.* Perioperative complications in liver transplantation using donation after cardiac death grafts: a propensity-matched study. *Liver Transpl* **20**, 823-830, doi:10.1002/lt.23888 (2014).

92 Padussis, J. C. *et al.* Feeding jejunostomy during Whipple is associated with increased morbidity. *J Surg Res* **187**, 361-366, doi:10.1016/j.jss.2012.10.010 (2014).

93 Moghadamyeghaneh, Z. *et al.* Preoperative dehydration increases risk of postoperative acute renal failure in colon and rectal surgery. *J Gastrointest Surg* **18**, 2178-2185, doi:10.1007/s11605-014-2661-7 (2014).

94 Mahdi, H. *et al.* Surgical site infection in women undergoing surgery for gynecologic cancer. *Int J Gynecol Cancer* **24**, 779-786, doi:10.1097/igc.0000000000000126 (2014).

95 Lin, J. A. *et al.* Adverse outcomes after major surgery in patients with systemic lupus erythematosus: a nationwide population-based study. *Ann Rheum Dis* **73**, 1646-1651, doi:10.1136/annrheumdis-2012-202758 (2014).

96 Lekerika, N. *et al.* Predicting fluid responsiveness in patients undergoing orthotopic liver transplantation: effects on intraoperative blood transfusion and postoperative complications. *Transplant Proc* **46**, 3087-3091, doi:10.1016/j.transproceed.2014.10.005 (2014).

97 Kobayashi, H. *et al.* Risk model for right hemicolectomy based on 19,070 Japanese patients in the National Clinical Database. *J Gastroenterol* **49**, 1047-1055, doi:10.1007/s00535-013-0860-8 (2014).

98 Kashy, B. K. *et al.* Effect of hydroxyethyl starch on postoperative kidney function in patients having noncardiac surgery. *Anesthesiology* **121**, 730-739, doi:10.1097/aln.0000000000000375 (2014).

99 Iwata, H. *et al.* Negative prognostic impact of renal replacement therapy in adult living-donor liver transplant recipients: preoperative recipient condition and donor factors. *Transplant Proc* **46**, 716-720, doi:10.1016/j.transproceed.2013.11.113 (2014).

100 Harris, D. G. *et al.* Recurrent kidney injury in critically ill surgical patients is common and associated with worse outcomes. *J Trauma Acute Care Surg* **76**, 1397-1401, doi:10.1097/ta.0000000000000241 (2014).

101 Halabi, W. J. *et al.* Surgery for gallstone ileus: a nationwide comparison of trends and outcomes. *Ann Surg* **259**, 329-335, doi:10.1097/SLA.0b013e31827eefed (2014).

102 Garg, A. X. *et al.* Perioperative aspirin and clonidine and risk of acute kidney injury: a randomized clinical trial. *Jama* **312**, 2254-2264, doi:10.1001/jama.2014.15284 (2014).

103 Garg, A. X. *et al.* Aspirin and clonidine in non-cardiac surgery: acute kidney injury substudy protocol of the Perioperative Ischaemic Evaluation (POISE) 2 randomised controlled trial. *BMJ Open* **4**, e004886, doi:10.1136/bmjopen-2014-004886 (2014).

104 Fernandes, A. *et al.* General anesthesia type does not influence serum levels of neutrophil gelatinase-associated lipocalin during the perioperative period in video laparoscopic bariatric surgery. *Clinics (Sao Paulo)* **69**, 655-659, doi:10.6061/clinics/2014(10)01 (2014).

105 Bosset, J. F. *et al.* Fluorouracil-based adjuvant chemotherapy after preoperative chemoradiotherapy in rectal cancer: long-term results of the EORTC 22921 randomised study. *The lancet. Oncology* **15**, 184‐190, doi:10.1016/S1470-2045(13)70599-0 (2014).

106 Yang, T. *et al.* Risk factors of hospital mortality after re-laparotomy for post-hepatectomy hemorrhage. *World J Surg* **37**, 2394-2401, doi:10.1007/s00268-013-2147-x (2013).

107 Wenger, U. *et al.* The relationship between preoperative creatinine clearance and outcomes for patients undergoing liver transplantation: a retrospective observational study. *BMC Nephrol* **14**, 37, doi:10.1186/1471-2369-14-37 (2013).

108 Walsh, M. *et al.* The association between perioperative hemoglobin and acute kidney injury in patients having noncardiac surgery. *Anesth Analg* **117**, 924-931, doi:10.1213/ANE.0b013e3182a1ec84 (2013).

109 Sharma, P., Goodrich, N. P., Schaubel, D. E., Guidinger, M. K. & Merion, R. M. Patient-specific prediction of ESRD after liver transplantation. *J Am Soc Nephrol* **24**, 2045-2052, doi:10.1681/asn.2013040436 (2013).

110 Kopolovic, I. *et al.* Risk factors and outcomes associated with acute kidney injury following ruptured abdominal aortic aneurysm. *BMC Nephrology* **14**, doi:10.1186/1471-2369-14-99 (2013).

111 Kermani, R. *et al.* A practical mortality risk score for emergent colectomy. *Dis Colon Rectum* **56**, 467-474, doi:10.1097/DCR.0b013e31827d0f93 (2013).

112 Duchesne, J. C. *et al.* Diluting the benefits of hemostatic resuscitation: a multi-institutional analysis. *J Trauma Acute Care Surg* **75**, 76-82, doi:10.1097/TA.0b013e3182987df3 (2013).

113 Blum, J. M. *et al.* Preoperative and intraoperative predictors of postoperative acute respiratory distress syndrome in a general surgical population. *Anesthesiology* **118**, 19-29, doi:10.1097/ALN.0b013e3182794975 (2013).

114 Bihorac, A. *et al.* National surgical quality improvement program underestimates the risk associated with mild and moderate postoperative acute kidney injury. *Crit Care Med* **41**, 2570-2583, doi:10.1097/CCM.0b013e31829860fc (2013).

115 Ahn, B. K. & Lee, K. H. Single-dose antibiotic prophylaxis is effective enough in colorectal surgery. *ANZ journal of surgery* **83**, 641‐645, doi:10.1111/j.1445-2197.2012.06244.x (2013).

116 Yassen, A. M. & Sayed, G. E. Low dose ketorolac infusion improves postoperative analgesia combined with patient controlled fentanyl analgesia after living donor hepatectomy - Randomized controlled trial. *Egyptian journal of anaesthesia* **28**, 199‐204, doi:10.1016/j.egja.2012.02.010 (2012).

117 Kondalsamy-Chennakesavan, S. *et al.* Risk factors to predict the incidence of surgical adverse events following open or laparoscopic surgery for apparent early stage endometrial cancer: results from a randomised controlled trial. *European journal of cancer (Oxford, England : 1990)* **48**, 2155‐2162, doi:10.1016/j.ejca.2012.03.013 (2012).

118 Hübner, M. *et al.* Preoperative immunonutrition in patients at nutritional risk: results of a double-blinded randomized clinical trial. *European journal of clinical nutrition* **66**, 850‐855, doi:10.1038/ejcn.2012.53 (2012).

119 Endo, A. *et al.* Intraoperative hydroxyethyl starch 70/0.5 is not related to acute kidney injury in surgical patients: retrospective cohort study. *Anesth Analg* **115**, 1309-1314, doi:10.1213/ANE.0b013e31826ba8d7 (2012).

120 Marchenko, T. V., Nikoda, V. V., Lishova, E. A., Goncharova, A. V. & Mayachkin, R. B. [Renal replacement therapy after elective surgical procedures]. *Khirurgiia (Mosk)*, 62-68, doi:10.17116/hirurgia201910162 (2019).

121 Long, T. E., Sigurdsson, M. I., Indridason, O. S., Sigvaldason, K. & Sigurdsson, G. H. [Epidemiology of acute kidney injury in a tertiary care university hospital according to the RIFLE criteria]. *Laeknabladid* **99**, 499-503, doi:10.17992/lbl.2013.11.516 (2013).

122 Woo, E. Y. *et al.* Open abdominal aortic aneurysm repair is feasible and can be done with excellent results in octogenarians. *J Vasc Surg* **53**, 278-284, doi:10.1016/j.jvs.2010.08.061 (2011).

123 Molnar, A. O. *et al.* Statin use associates with a lower incidence of acute kidney injury after major elective surgery. *J Am Soc Nephrol* **22**, 939-946, doi:10.1681/asn.2010050442 (2011).

124 Jeyabalan, G., Park, T., Rhee, R. Y., Makaroun, M. S. & Cho, J. S. Comparison of modern open infrarenal and pararenal abdominal aortic aneurysm repair on early outcomes and renal dysfunction at one year. *J Vasc Surg* **54**, 654-659, doi:10.1016/j.jvs.2011.03.007 (2011).

125 Suzuki, T., Miyao, H., Terui, K., Koyama, K. & Shiibashi, M. Fluid therapy with hydroxyethyl starch for massive blood loss during surgery. *J Anesth* **24**, 418-425, doi:10.1007/s00540-010-0914-5 (2010).

126 Spyropoulos, C., Kehagias, I., Panagiotopoulos, S., Mead, N. & Kalfarentzos, F. Revisional bariatric surgery: 13-year experience from a tertiary institution. *Arch Surg* **145**, 173-177, doi:10.1001/archsurg.2009.260 (2010).

127 Speziale, F., Ruggiero, M., Sbarigia, E., Marino, M. & Menna, D. Factors influencing outcome after open surgical repair of juxtarenal abdominal aortic aneurysms. *Vascular* **18**, 141-146, doi:10.2310/6670.2010.00035 (2010).

128 van Kuijk, J. P. *et al.* Preoperative left ventricular dysfunction predisposes to postoperative acute kidney injury and long-term mortality. *J Nephrol* **24**, 764-770, doi:10.5301/jn.2011.6384 (2011).

129 Burra, P. *et al.* Factors influencing renal function after liver transplantation. Results from the MOST, an international observational study. *Dig Liver Dis* **41**, 350-356, doi:10.1016/j.dld.2008.09.018 (2009).

130 Bentrem, D. J., Cohen, M. E., Hynes, D. M., Ko, C. Y. & Bilimoria, K. Y. Identification of specific quality improvement opportunities for the elderly undergoing gastrointestinal surgery. *Arch Surg* **144**, 1013-1020, doi:10.1001/archsurg.2009.114 (2009).

131 Antonello, M. *et al.* Open repair for ruptured abdominal aortic aneurysm: is it possible to predict survival? *Ann Vasc Surg* **23**, 159-166, doi:10.1016/j.avsg.2008.05.011 (2009).

132 Abelha, F. J., Botelho, M., Fernandes, V. & Barros, H. Determinants of postoperative acute kidney injury. *Crit Care* **13**, R79, doi:10.1186/cc7894 (2009).

133 Brown, L. C., Thompson, S. G., Greenhalgh, R. M. & Powell, J. T. Fit patients with small abdominal aortic aneurysms (AAAs) do not benefit from early intervention. *Journal of vascular surgery* **48**, 1375‐1381, doi:10.1016/j.jvs.2008.07.014 (2008).

134 Pernicky, M., Murin, J. & Labas, P. Perioperative cardiovascular and noncardiovascular risk in patients with colorectal cancer. *Bratisl Lek Listy* **108**, 246-250 (2007).

135 Zhou, J. *et al.* Conversion to sirolimus immunosuppression in liver transplantation recipients with hepatocellular carcinoma: Report of an initial experience. *World J Gastroenterol* **12**, 3114-3118, doi:10.3748/wjg.v12.i19.3114 (2006).

136 Senekowitsch, C. *et al.* Replanting the inferior mesentery artery during infrarenal aortic aneurysm repair: influence on postoperative colon ischemia. *Journal of vascular surgery* **43**, 689‐694, doi:10.1016/j.jvs.2005.12.016 (2006).

137 de Freitas Carvalho, D. A. *et al.* Rhabdomyolysis after bariatric surgery. *Obes Surg* **16**, 740-744, doi:10.1381/096089206777346655 (2006).

138 Inabnet, W. B., Quinn, T., Gagner, M., Urban, M. & Pomp, A. Laparoscopic Roux-en-Y gastric bypass in patients with BMI <50: a prospective randomized trial comparing short and long limb lengths. *Obesity surgery* **15**, 51‐57, doi:10.1381/0960892052993468 (2005).

139 Sjöström, L. *et al.* Lifestyle, diabetes, and cardiovascular risk factors 10 years after bariatric surgery. *New England journal of medicine* **351**, 2683‐2693, doi:10.1056/NEJMoa035622 (2004).

140 Kudo, F. A. *et al.* Postoperative renal function after elective abdominal aortic aneurysm repair requiring suprarenal aortic cross-clamping. *Surg Today* **34**, 1010-1013, doi:10.1007/s00595-004-2871-9 (2004).

141 Lo, P. H. *et al.* Adverse Outcomes after Non-Cardiac Surgeries in Patients with Heart Failure: A Propensity-Score Matched Study. *J Clin Med* **10**, doi:10.3390/jcm10071501 (2021).

142 Geiger, J. T. *et al.* One-year patient survival correlates with surgeon volume after elective open abdominal aortic surgery. *J Vasc Surg* **73**, 108-116.e101, doi:10.1016/j.jvs.2020.04.509 (2021).

143 Choo, S. J., Jeon, Y. B., Oh, S. S. & Shinn, S. H. Outcomes of emergency endovascular versus open repair for abdominal aortic aneurysm rupture. *Ann Surg Treat Res* **100**, 291-297, doi:10.4174/astr.2021.100.5.291 (2021).

144 Chen, L., Peng, L., Wang, C., Li, S. C. & Zhang, M. New score for prediction of morbidity in patients undergoing open pancreaticoduodenectomy. *Journal of International Medical Research* **49**, doi:10.1177/03000605211001984 (2021).

145 Yazawa, M. *et al.* Longitudinal Renal Function in Liver Transplant Recipients With Acute-on-Chronic Liver Failure. *Clin Transl Gastroenterol* **11**, e00185, doi:10.14309/ctg.0000000000000185 (2020).

146 Weber, K. T. *et al.* Effect of Body Mass Index on Outcomes After Surgery for Perforated Diverticulitis. *J Surg Res* **247**, 220-226, doi:10.1016/j.jss.2019.10.020 (2020).

147 Sanaiha, Y. *et al.* Acute kidney injury is independently associated with mortality and resource use after emergency general surgery operations. *Surgery* **167**, 328-334, doi:10.1016/j.surg.2019.07.035 (2020).

148 Paredes, A. Z. *et al.* Skilled nursing facility (SNF) utilization and impact of SNF star-quality ratings on outcomes following hepatectomy among Medicare beneficiaries. *HPB* **22**, 109-115, doi:10.1016/j.hpb.2019.05.012 (2020).

149 Liakopoulos, V. *et al.* Renal and cardiovascular outcomes after weight loss from gastric bypass surgery in type 2 diabetes: Cardiorenal risk reductions exceed atherosclerotic benefits. *Diabetes Care* **43**, 1276-1284, doi:10.2337/dc19-1703 (2020).

150 Lee, H. J., Kim, W. H., Jung, C. W., Suh, K. S. & Lee, K. H. Different Severity of Clinical Outcomes Between the 2 Subgroups of Stage 1 Acute Kidney Injury After Liver Transplantation. *Transplantation* **104**, 2327-2333, doi:10.1097/tp.0000000000003135 (2020).

151 Kunstman, J. W., Brandt, W. S., Azar, S. A., Jean, R. A. & Salem, R. R. Comprehensive Analysis of the Effect of Ketorolac Administration after Pancreaticoduodenectomy. *J Am Coll Surg* **230**, 935-942.e932, doi:10.1016/j.jamcollsurg.2020.02.024 (2020).

152 Jin, J. *et al.* Prior bariatric surgery and perioperative cardiovascular outcomes following noncardiac surgery in patients with type 2 diabetes mellitus: Hint from National Inpatient Sample Database. *Cardiovascular Diabetology* **19**, doi:10.1186/s12933-020-01084-7 (2020).

153 Jiang, Y., Rim, D. S., Rodgers, B. & Ahlawat, S. Sarcoidosis is associated with lower risks of penetrating disease and colectomy in hospitalized patients with inflammatory bowel disease. *JGH Open* **4**, 1199-1206, doi:10.1002/jgh3.12423 (2020).

154 Drazic, O. D. *et al.* Juxtarenal Abdominal Aortic Aneurysm: Results of Open Surgery in an Academic Center. *Ann Vasc Surg* **66**, 28-34, doi:10.1016/j.avsg.2019.10.036 (2020).

155 Chen, C. Y. *et al.* Risk factors of acute renal impairment after cytoreductive surgery and hyperthermic intraperitoneal chemotherapy. *International Journal of Hyperthermia* **37**, 1279-1286, doi:10.1080/02656736.2020.1846793 (2020).

156 Chang, E. H. *et al.* Obesity and surgical complications of pancreaticoduodenectomy: An observation study utilizing ACS NSQIP. *Am J Surg* **220**, 135-139, doi:10.1016/j.amjsurg.2019.10.030 (2020).

157 Are, V. S. *et al.* Improving Outcomes of Bariatric Surgery in Patients With Cirrhosis in the United States: A Nationwide Assessment. *Am J Gastroenterol* **115**, 1849-1856, doi:10.14309/ajg.0000000000000911 (2020).

158 Weinberg, L. *et al.* Impact of a goal directed fluid therapy algorithm on postoperative morbidity in patients undergoing open right hepatectomy: A single centre retrospective observational study. *BMC Anesthesiology* **19**, doi:10.1186/s12871-019-0803-x (2019).

159 Tarbunou, Y. A., Smith, J. B., Kruse, R. L. & Vogel, T. R. Outcomes associated with hyperglycemia after abdominal aortic aneurysm repair. *J Vasc Surg* **69**, 763-773.e763, doi:10.1016/j.jvs.2018.05.240 (2019).

160 Sharma, P., McCarty, T. R., Yadav, S., Ngu, J. N. & Njei, B. Impact of Bariatric Surgery on Outcomes of Patients with Sickle Cell Disease: a Nationwide Inpatient Sample Analysis, 2004-2014. *Obes Surg* **29**, 1789-1796, doi:10.1007/s11695-019-03780-0 (2019).

161 Sandler, B. J., Davis, K. A. & Schuster, K. M. Symptomatic human immunodeficiency virus-infected patients have poorer outcomes following emergency general surgery: A study of the nationwide inpatient sample. *J Trauma Acute Care Surg* **86**, 479-488, doi:10.1097/ta.0000000000002161 (2019).

162 Pechman, D. M. *et al.* Bariatric surgery in the elderly: outcomes analysis of patients over 70 using the ACS-NSQIP database. *Surg Obes Relat Dis* **15**, 1923-1932, doi:10.1016/j.soard.2019.08.011 (2019).

163 Ninh, A., Wood, K., Bui, A. H. & Leitman, I. M. Risk Factors and Outcomes for Sepsis after Appendectomy in Adults. *Surg Infect (Larchmt)* **20**, 601-606, doi:10.1089/sur.2019.003 (2019).

164 Ma, T. *et al.* Value of pretransplant albumin-bilirubin score in predicting outcomes after liver transplantation. *World Journal of Gastroenterology* **25**, 1879-1889, doi:10.3748/wjg.v25.i15.1879 (2019).

165 Lin, C. S. *et al.* Defining risk of general surgery in patients with chronic obstructive pulmonary diseases. *Qjm* **112**, 107-113, doi:10.1093/qjmed/hcy240 (2019).

166 Levitsky, J. *et al.* External Validation of a Pretransplant Biomarker Model (REVERSE) Predictive of Renal Recovery After Liver Transplantation. *Hepatology* **70**, 1349-1359, doi:10.1002/hep.30667 (2019).

167 Lee, S. *et al.* Long-term impact of dialysis-requiring AKI during the perioperative period of liver transplantation on postdischarge outcomes. *Clin Transplant* **33**, e13649, doi:10.1111/ctr.13649 (2019).

168 Kim, M., Wall, M. M., Kiran, R. P. & Li, G. Latent class analysis stratifies mortality risk in patients developing acute kidney injury after high-risk intraabdominal general surgery: a historical cohort study. *Can J Anaesth* **66**, 36-47, doi:10.1007/s12630-018-1221-0 (2019).

169 Keuffel, E. L., Rizzo, J., Stevens, M., Gunnarsson, C. & Maheshwari, K. Hospital costs associated with intraoperative hypotension among non-cardiac surgical patients in the US: a simulation model. *J Med Econ* **22**, 645-651, doi:10.1080/13696998.2019.1591147 (2019).

170 Kazemi, A., Kazemi, K., Sami, A. & Sharifian, R. Identifying Factors That Affect Patient Survival After Orthotopic Liver Transplant Using Machine-Learning Techniques. *Exp Clin Transplant* **17**, 775-783, doi:10.6002/ect.2018.0170 (2019).

171 Hefler, J. *et al.* Effects of Chronic Corticosteroid and Immunosuppressant Use in Patients Undergoing Bariatric Surgery. *Obesity Surgery* **29**, 3309-3315, doi:10.1007/s11695-019-03995-1 (2019).

172 Grahn, S. W. *et al.* System-Wide Improvement for Transitions After Ileostomy Surgery: Can Intensive Monitoring of Protocol Compliance Decrease Readmissions? A Randomized Trial. *Dis Colon Rectum* **62**, 363-370, doi:10.1097/dcr.0000000000001286 (2019).

173 Dang, T., Dakour-Aridi, H., Rizwan, M., Nejim, B. & Malas, M. B. Predictors of acute kidney injury after infrarenal abdominal aortic aneurysm repair in octogenarians. *J Vasc Surg* **69**, 752-762.e751, doi:10.1016/j.jvs.2018.05.227 (2019).

174 Braunwarth, E. *et al.* Is bile leakage after hepatic resection associated with impaired long-term survival? *Eur J Surg Oncol* **45**, 1077-1083, doi:10.1016/j.ejso.2019.02.021 (2019).

175 Ali, A. Y. *et al.* Effect of Duration of Intensive Care Unit Stay on Outcomes of Adult Living Donor Liver Transplant Recipients. *Transplant Proc* **51**, 2425-2429, doi:10.1016/j.transproceed.2019.03.045 (2019).

176 Yoon, K. C. *et al.* Urinary Neutrophil Gelatinase-Associated Lipocalin as a Biomarker for Renal Injury in Liver Transplant Recipients Using Calcineurin Inhibitors. *Transplant Proc* **50**, 3667-3672, doi:10.1016/j.transproceed.2018.09.009 (2018).

177 Sugimoto, M. *et al.* Long-term fate of renal function after open surgery for juxtarenal and pararenal aortic aneurysm. *J Vasc Surg* **67**, 1042-1050, doi:10.1016/j.jvs.2017.07.121 (2018).

178 Maheshwari, K. *et al.* The association of hypotension during non-cardiac surgery, before and after skin incision, with postoperative acute kidney injury: a retrospective cohort analysis. *Anaesthesia* **73**, 1223-1228, doi:10.1111/anae.14416 (2018).

179 Lin, Y. F., Huang, T. M., Lin, S. L., Wu, V. C. & Wu, K. D. Short-and long-term outcomes after postsurgical acute kidney injury requiring dialysis. *Clinical Epidemiology* **10**, 1583-1598, doi:10.2147/CLEP.S169302 (2018).

180 Koppe, U. *et al.* Long-term effects of bariatric surgery on acute kidney injury: a propensity-matched cohort in the UK Clinical Practice Research Datalink. *BMJ Open* **8**, e020371, doi:10.1136/bmjopen-2017-020371 (2018).

181 Kim, M., Wall, M. M. & Li, G. Risk Stratification for Major Postoperative Complications in Patients Undergoing Intra-abdominal General Surgery Using Latent Class Analysis. *Anesth Analg* **126**, 848-857, doi:10.1213/ane.0000000000002345 (2018).

182 Kim, M. & Li, G. Two-way Interaction Effects of Perioperative Complications on 30-Day Mortality in General Surgery. *World J Surg* **42**, 2-11, doi:10.1007/s00268-017-4156-7 (2018).

183 Kassahun, W. T., Staab, H., Gockel, I. & Mehdorn, M. Factors associated with morbidity and in-hospital mortality after surgery beyond the age of 90: Comparison with outcome results of younger patients matched for treatment. *American Journal of Surgery* **216**, 1063-1069, doi:10.1016/j.amjsurg.2017.11.032 (2018).

184 Ilic, N. S. *et al.* Evaluation of the renal function using serum Cystatin C following open and endovascular aortic aneurysm repair. *Vascular* **26**, 132-141, doi:10.1177/1708538117717348 (2018).

185 Higgins, R. M., Helm, M., Gould, J. C. & Kindel, T. L. Preoperative immobility significantly impacts the risk of postoperative complications in bariatric surgery patients. *Surg Obes Relat Dis* **14**, 842-848, doi:10.1016/j.soard.2018.02.025 (2018).

186 Blackwell, R. H. *et al.* Complications of Recognized and Unrecognized Iatrogenic Ureteral Injury at Time of Hysterectomy: A Population Based Analysis. *J Urol* **199**, 1540-1545, doi:10.1016/j.juro.2017.12.067 (2018).

187 Behrendt, C. A. *et al.* Incidence, Predictors, and Outcomes of Colonic Ischaemia in Abdominal Aortic Aneurysm Repair. *Eur J Vasc Endovasc Surg* **56**, 507-513, doi:10.1016/j.ejvs.2018.06.010 (2018).

188 Antoniak, D. *et al.* The Relationship Between Age and Chronic Kidney Disease in Patients Undergoing Pancreatic Resection. *J Gastrointest Surg* **22**, 1376-1384, doi:10.1007/s11605-018-3743-8 (2018).

189 Acheampong, D. *et al.* Unplanned postoperative reintubation following general and vascular surgical procedures: Outcomes and risk factors. *Ann Med Surg (Lond)* **33**, 40-43, doi:10.1016/j.amsu.2018.08.013 (2018).

190 Zongyi, Y., Baifeng, L., Funian, Z., Hao, L. & Xin, W. Risk factors of acute kidney injury after orthotopic liver transplantation in China. *Sci Rep* **7**, 41555, doi:10.1038/srep41555 (2017).

191 Siracuse, J. J. *et al.* Contemporary open repair of ruptured abdominal aortic aneurysms. *J Vasc Surg* **65**, 1023-1028, doi:10.1016/j.jvs.2016.08.115 (2017).

192 Sin, E. I. L., Chia, C. S., Tan, G. H. C., Soo, K. C. & Teo, M. C. C. Acute kidney injury in ovarian cancer patients undergoing cytoreductive surgery and hyperthermic intra-peritoneal chemotherapy. *International Journal of Hyperthermia* **33**, 690-695, doi:10.1080/02656736.2017.1293304 (2017).

193 Pan, Y., Wang, W., Wang, J., Yang, L. & Ding, F. Incidence and Risk Factors of in-hospital mortality from AKI after non-cardiovascular operation: A nationwide Survey in China. *Sci Rep* **7**, 13953, doi:10.1038/s41598-017-13763-9 (2017).

194 Nandan, A. R. *et al.* The Emergency Surgery Score (ESS) accurately predicts the occurrence of postoperative complications in emergency surgery patients. *J Trauma Acute Care Surg* **83**, 84-89, doi:10.1097/ta.0000000000001500 (2017).

195 Maile, M. D., Armstrong, W. F., Jewell, E. S. & Engoren, M. C. Impact of ejection fraction on infectious, renal, and respiratory morbidity for patients undergoing noncardiac surgery. *J Clin Anesth* **36**, 1-9, doi:10.1016/j.jclinane.2016.08.038 (2017).

196 Li, L. C. *et al.* Proteinuria and baseline renal function predict mortality and renal outcomes after sirolimus therapy in liver transplantation recipients. *BMC Gastroenterol* **17**, 58, doi:10.1186/s12876-017-0611-z (2017).

197 Li, L. *et al.* Ileostomy creation in colorectal cancer surgery: risk of acute kidney injury and chronic kidney disease. *J Surg Res* **210**, 204-212, doi:10.1016/j.jss.2016.11.039 (2017).

198 Lee, S. Y. *et al.* Perioperative Infections after Open Abdominal Aortic Aneurysm Repair Lead to Increased Risk of Subsequent Complications. *Ann Vasc Surg* **44**, 203-210, doi:10.1016/j.avsg.2017.04.022 (2017).

199 Kunisaki, C. *et al.* Modeling preoperative risk factors for potentially lethal morbidities using a nationwide Japanese web-based database of patients undergoing distal gastrectomy for gastric cancer. *Gastric Cancer* **20**, 496-507, doi:10.1007/s10120-016-0634-0 (2017).

200 Kumar, P., Renuka, M. K., Kalaiselvan, M. S. & Arunkumar, A. S. Outcome of noncardiac surgical patients admitted to a multidisciplinary intensive care unit. *Indian Journal of Critical Care Medicine* **21**, 17-22, doi:10.4103/0972-5229.198321 (2017).

201 Kulaylat, A. S., Kassam, Z., Hollenbeak, C. S. & Stewart, D. B., Sr. A Surgical Clostridium-Associated Risk of Death Score Predicts Mortality After Colectomy for Clostridium difficile. *Dis Colon Rectum* **60**, 1285-1290, doi:10.1097/dcr.0000000000000920 (2017).

202 Kim, S. G., Larson, J. J., Lee, J. S., Therneau, T. M. & Kim, W. R. Beneficial and harmful effects of nonselective beta blockade on acute kidney injury in liver transplant candidates. *Liver Transpl* **23**, 733-740, doi:10.1002/lt.24744 (2017).

203 Hokuto, D. *et al.* The Administration of Celecoxib as an Analgesic after Liver Resection Is Safe. *Dig Surg* **34**, 108-113, doi:10.1159/000449042 (2017).

204 Hicks, C. W., O'Kelly, A., Obeid, T., Locham, S. & Malas, M. B. Predicting failure to rescue after abdominal aortic aneurysm repair in elderly patients. *J Surg Res* **217**, 265-270, doi:10.1016/j.jss.2017.06.025 (2017).

205 Ding, W. *et al.* Open Abdomen Improves Survival in Patients With Peritonitis Secondary to Acute Superior Mesenteric Artery Occlusion. *J Clin Gastroenterol* **51**, e77-e82, doi:10.1097/mcg.0000000000000799 (2017).

206 Chapman, W. C. *et al.* Effect of Early Everolimus-Facilitated Reduction of Tacrolimus on Efficacy and Renal Function in De Novo Liver Transplant Recipients: 24-Month Results for the North American Subpopulation. *Transplantation* **101**, 341-349, doi:10.1097/tp.0000000000001524 (2017).

207 Babazade, R., Yilmaz, H. O., Leung, S. M., Zimmerman, N. M. & Turan, A. Systemic Lupus Erythematosus Is Associated With Increased Adverse Postoperative Renal Outcomes and Mortality: A Historical Cohort Study Using Administrative Health Data. *Anesth Analg* **124**, 1118-1126, doi:10.1213/ane.0000000000001911 (2017).

208 Alizadeh, R. F., Sujatha-Bhaskar, S., Li, S., Stamos, M. J. & Nguyen, N. T. Venous thromboembolism in common laparoscopic abdominal surgical operations. *American Journal of Surgery* **214**, 1127-1132, doi:10.1016/j.amjsurg.2017.08.032 (2017).

209 Wadei, H. M. *et al.* Early Allograft Dysfunction After Liver Transplantation Is Associated With Short- and Long-Term Kidney Function Impairment. *Am J Transplant* **16**, 850-859, doi:10.1111/ajt.13527 (2016).

210 Tahaoglu, A. E. *et al.* Emergency peripartum hysterectomy: our experience. *Irish Journal of Medical Science* **185**, 833-838, doi:10.1007/s11845-015-1376-4 (2016).

211 Ramonell, K. M. *et al.* Development and Validation of a Risk Calculator for Renal Complications after Colorectal Surgery Using the National Surgical Quality Improvement Program Participant Use Files. *Am Surg* **82**, 1244-1249 (2016).

212 Ozrazgat-Baslanti, T. *et al.* Preoperative assessment of the risk for multiple complications after surgery. *Surgery* **160**, 463-472, doi:10.1016/j.surg.2016.04.013 (2016).

213 Li, Y., Stocchi, L., Cherla, D., Liu, X. & Remzi, F. H. Association of preoperative narcotic use with postoperative complications and prolonged length of hospital stay in patients with Crohn disease. *JAMA Surgery* **151**, 726-734, doi:10.1001/jamasurg.2015.5558 (2016).

214 Kang, G. W. *et al.* One-Year Follow-up of the Changes in Renal Function After Liver Transplantation in Patients Without Chronic Kidney Disease. *Transplant Proc* **48**, 1190-1193, doi:10.1016/j.transproceed.2016.02.013 (2016).

215 Inoue, Y. *et al.* Does the development of chronic kidney disease and acute kidney injury affect the prognosis after living donor liver transplantation? *Clin Transplant* **30**, 518-527, doi:10.1111/ctr.12715 (2016).

216 Ferrante, A. M., Moscato, U., Colacchio, E. C. & Snider, F. Results after elective open repair of pararenal abdominal aortic aneurysms. *J Vasc Surg* **63**, 1443-1450, doi:10.1016/j.jvs.2015.12.034 (2016).

217 Desai, M., Choke, E., Sayers, R. D., Nath, M. & Bown, M. J. Sex-related trends inmortality after elective abdominal aortic aneurysmsurgery between 2002 and 2013 at National Health Service hospitals in England: Less benefit for women compared with men. *European Heart Journal* **37**, 3452-3460, doi:10.1093/eurheartj/ehw335 (2016).

218 Daniels, S. E. *et al.* A Pooled Analysis Evaluating Renal Safety in Placebo- and Active Comparator-Controlled Phase III Trials of Multiple-Dose Injectable HPβCD-Diclofenac in Subjects with Acute Postoperative Pain. *Pain Med* **17**, 2378-2388, doi:10.1093/pm/pnw146 (2016).

219 Blitz, J. D. *et al.* Preoperative Renal Insufficiency: Underreporting and Association With Readmission and Major Postoperative Morbidity in an Academic Medical Center. *Anesth Analg* **123**, 1500-1515, doi:10.1213/ane.0000000000001573 (2016).

220 Aga, Z., Machina, M. & McCluskey, S. A. Greater intravenous fluid volumes are associated with prolonged recovery after colorectal surgery: a retrospective cohort study. *Br J Anaesth* **116**, 804-810, doi:10.1093/bja/aew125 (2016).

221 Abdullah, H. R. *et al.* Predictors of Perioperative Acute Kidney Injury in Obese Patients Undergoing Laparoscopic Bariatric Surgery: a Single-Centre Retrospective Cohort Study. *Obes Surg* **26**, 1493-1499, doi:10.1007/s11695-015-1938-6 (2016).

222 Wakeam, E., Hyder, J. A., Jiang, W., Lipsitz, S. A. & Finlayson, S. Risk and patterns of secondary complications in surgical inpatients. *JAMA Surg* **150**, 65-73, doi:10.1001/jamasurg.2014.1795 (2015).

223 Leon-Justel, A. *et al.* Point-of-care haemostasis monitoring during liver transplantation reduces transfusion requirements and improves patient outcome. *Clin Chim Acta* **446**, 277-283, doi:10.1016/j.cca.2015.04.022 (2015).

224 Kim, M., Brady, J. E. & Li, G. Interaction Effects of Acute Kidney Injury, Acute Respiratory Failure, and Sepsis on 30-Day Postoperative Mortality in Patients Undergoing High-Risk Intraabdominal General Surgical Procedures. *Anesth Analg* **121**, 1536-1546, doi:10.1213/ane.0000000000000915 (2015).

225 Zhang, J. *et al.* Acute kidney injury after radical gastrectomy: a single center study. *Int Urol Nephrol* **46**, 973-977, doi:10.1007/s11255-013-0618-5 (2014).

226 Squires, M. H., 3rd *et al.* Effect of preoperative renal insufficiency on postoperative outcomes after pancreatic resection: a single institution experience of 1,061 consecutive patients. *J Am Coll Surg* **218**, 92-101, doi:10.1016/j.jamcollsurg.2013.09.012 (2014).

227 Smoter, P. *et al.* Risk factors of acute renal failure after orthotopic liver transplantation: single-center experience. *Transplant Proc* **46**, 2786-2789, doi:10.1016/j.transproceed.2014.09.044 (2014).

228 Klaus, F. *et al.* Acute kidney injury after liver transplantation: incidence and mortality. *Transplant Proc* **46**, 1819-1821, doi:10.1016/j.transproceed.2014.05.053 (2014).

229 Kimbrough, C. W. *et al.* Factors predictive of readmission after hepatic resection for hepatocellular carcinoma. *Surgery* **156**, 1039-1046, doi:10.1016/j.surg.2014.06.057 (2014).

230 Kim, M., Brady, J. E. & Li, G. Variations in the risk of acute kidney injury across intraabdominal surgery procedures. *Anesth Analg* **119**, 1121-1132, doi:10.1213/ane.0000000000000425 (2014).

231 Kim, J. M. *et al.* The predictors for continuous renal replacement therapy in liver transplant recipients. *Transplant Proc* **46**, 184-191, doi:10.1016/j.transproceed.2013.07.075 (2014).

232 Aberg, F. *et al.* Neutrophil gelatinase-associated lipocalin associated with irreversibility of pre-liver transplant kidney dysfunction. *Clin Transplant* **28**, 869-876, doi:10.1111/ctr.12394 (2014).

233 Jafari, M. D. *et al.* Morbidity of diverting ileostomy for rectal cancer: analysis of the American College of Surgeons National Surgical Quality Improvement Program. *Am Surg* **79**, 1034-1039 (2013).

234 Suzuki, M. *et al.* Risk factors for native kidney dysfunction in patients with abdominal multivisceral/small bowel transplantation. *Clin Transplant* **26**, E351-358, doi:10.1111/j.1399-0012.2012.01672.x (2012).

235 Masoomi, H. *et al.* Predictive factors of acute renal failure in colon and rectal surgery. *Am Surg* **78**, 1019-1023, doi:10.1177/000313481207801001 (2012).

236 Lin, Y. H. *et al.* The 4-week serum creatinine level predicts long-term renal dysfunction after adult living donor liver transplantation. *Transplant Proc* **44**, 772-775, doi:10.1016/j.transproceed.2012.03.034 (2012).

237 Lidsky, M. E., Thacker, J. K., Lagoo-Deenadayalan, S. A. & Scarborough, J. E. Advanced age is an independent predictor for increased morbidity and mortality after emergent surgery for diverticulitis. *Surgery* **152**, 465-472, doi:10.1016/j.surg.2012.06.038 (2012).

238 Kopolovic, I. *et al.* Elevated cardiac troponin in the early post-operative period and mortality following ruptured abdominal aortic aneurysm: a retrospective population-based cohort study. *Critical Care* **16**, doi:10.1186/cc11461 (2012).

239 Siniscalchi, A. *et al.* Postoperative troponin T elevation as a predictor of early acute kidney injury after orthotopic liver transplantation: a preliminary retrospective study. *Transplant Proc* **44**, 1999-2001, doi:10.1016/j.transproceed.2012.06.039 (2012).

240 Karapanagiotou, A. *et al.* Acute kidney injury after orthotopic liver transplantation. *Transplant Proc* **44**, 2727-2729, doi:10.1016/j.transproceed.2012.09.096 (2012).

241 Inoue, Y. *et al.* Acute kidney injury following living donor liver transplantation. *Clin Transplant* **26**, E530-535, doi:10.1111/ctr.12027 (2012).

242 Huang, L. *et al.* Prealbumin is predictive for postoperative liver insufficiency in patients undergoing liver resection. *World Journal of Gastroenterology* **18**, 7021-7025, doi:10.3748/wjg.v18.i47.7021 (2012).

243 Hu, C. J., Liao, C. C., Chang, C. C., Wu, C. H. & Chen, T. L. Postoperative adverse outcomes in surgical patients with dementia: a retrospective cohort study. *World J Surg* **36**, 2051-2058, doi:10.1007/s00268-012-1609-x (2012).

244 Hong, S. H., Park, C. O. & Park, C. S. Prediction of newly developed acute renal failure using serum phosphorus concentrations after living-donor liver transplantation. *J Int Med Res* **40**, 2199-2212, doi:10.1177/030006051204000618 (2012).

245 Boin, I. F. *et al.* Can pre-liver transplantation renal insufficiency using a creatinine clearance calculator predict long-term survival? *Transplant Proc* **44**, 2452-2454, doi:10.1016/j.transproceed.2012.07.028 (2012).

246 Zhu, B., Geng, L., Ma, Y. G., Zhang, Y. J. & Wu, M. C. Combined invagination and duct-to-mucosa techniques with modifications: a new method of pancreaticojejunal anastomosis. *Hepatobiliary Pancreat Dis Int* **10**, 422-427, doi:10.1016/s1499-3872(11)60072-6 (2011).

247 Zand, M. S. *et al.* High mortality in orthotopic liver transplant recipients who require hemodialysis. *Clin Transplant* **25**, 213-221, doi:10.1111/j.1399-0012.2010.01238.x (2011).

248 Verna, E. C. *et al.* Basiliximab induction and delayed calcineurin inhibitor initiation in liver transplant recipients with renal insufficiency. *Transplantation* **91**, 1254-1260, doi:10.1097/TP.0b013e318218f0f5 (2011).

249 Park, B. *et al.* Obesity is not an independent risk factor for adverse perioperative and long-term clinical outcomes following open AAA repair or EVAR. *Vasc Endovascular Surg* **45**, 607-613, doi:10.1177/1538574411415427 (2011).

250 Mashour, G. A., Shanks, A. M. & Kheterpal, S. Perioperative stroke and associated mortality after noncardiac, nonneurologic surgery. *Anesthesiology* **114**, 1289-1296, doi:10.1097/ALN.0b013e318216e7f4 (2011).

251 Ling, Q. *et al.* Impact of preexisting diabetes mellitus on outcome after liver transplantation in patients with hepatitis B virus-related liver disease. *Digestive Diseases and Sciences* **56**, 889-893, doi:10.1007/s10620-010-1358-3 (2011).

252 Memmo, A. *et al.* Perioperative fenoldopam for the prevention of acute renal failure in non-cardiac surgery, randomized clinical trial. *Signa Vitae* **6**, 14-19, doi:10.22514/SV61.052011.2 (2011).

253 Lin, J. A., Liao, C. C., Chang, C. C., Chang, H. & Chen, T. L. Postoperative adverse outcomes in intellectually disabled surgical patients: a nationwide population-based study. *PLoS One* **6**, e26977, doi:10.1371/journal.pone.0026977 (2011).

254 Leithead, J. A. *et al.* Chronic kidney disease after liver transplantation for acute liver failure is not associated with perioperative renal dysfunction. *Am J Transplant* **11**, 1905-1915, doi:10.1111/j.1600-6143.2011.03649.x (2011).

255 Chen, J. *et al.* Postliver transplant acute renal injury and failure by the RIFLE criteria in patients with normal pretransplant serum creatinine concentrations: a matched study. *Transplantation* **91**, 348-353, doi:10.1097/TP.0b013e31820437da (2011).

256 Arthurs, Z. M. *et al.* A comparison of endovascular revascularization with traditional therapy for the treatment of acute mesenteric ischemia. *J Vasc Surg* **53**, 698-704; discussion 704-695, doi:10.1016/j.jvs.2010.09.049 (2011).

257 Ramachandran, J. *et al.* Chronic kidney disease following liver transplantation: a South Australian experience. *Transplant Proc* **42**, 3644-3646, doi:10.1016/j.transproceed.2010.06.022 (2010).

258 Mathur, A. K. *et al.* Influence of body mass index on complications and oncologic outcomes following hepatectomy for malignancy. *J Gastrointest Surg* **14**, 849-857, doi:10.1007/s11605-010-1163-5 (2010).

259 Martin, M. C. *et al.* National outcomes after open repair of abdominal aortic aneurysms with visceral or renal bypass. *Ann Vasc Surg* **24**, 106-112, doi:10.1016/j.avsg.2009.09.008 (2010).

260 Khan, N. A. *et al.* Risk of intraoperative hypotension with loop diuretics: a randomized controlled trial. *American journal of medicine* **123**, 1059.e1051‐1058, doi:10.1016/j.amjmed.2010.07.019 (2010).

261 Glance, L. G. *et al.* Perioperative outcomes among patients with the modified metabolic syndrome who are undergoing noncardiac surgery. *Anesthesiology* **113**, 859-872, doi:10.1097/ALN.0b013e3181eff32e (2010).

262 Davies, R. S., Dawlatly, S., Clarkson, J. R., Bradbury, A. W. & Adam, D. J. Outcome in patients requiring renal replacement therapy after open surgical repair for ruptured abdominal aortic aneurysm. *Vasc Endovascular Surg* **44**, 170-173, doi:10.1177/1538574410361972 (2010).

263 Subramaniam, B. *et al.* Continuous perioperative insulin infusion decreases major cardiovascular events in patients undergoing vascular surgery: a prospective, randomized trial. *Anesthesiology* **110**, 970-977, doi:10.1097/ALN.0b013e3181a1005b (2009).

264 Silecchia, G. *et al.* Two-stage laparoscopic biliopancreatic diversion with duodenal switch as treatment of high-risk super-obese patients: analysis of complications. *Surg Endosc* **23**, 1032-1037, doi:10.1007/s00464-008-0113-8 (2009).

265 Senagore, A. J., Stulberg, J. J., Byrnes, J. & Delaney, C. P. A national comparison of laparoscopic vs. open colectomy using the National Surgical Quality Improvement Project data. *Dis Colon Rectum* **52**, 183-186, doi:10.1007/DCR.0b013e31819ad4a4 (2009).

266 Paugam-Burtz, C. *et al.* Postreperfusion syndrome during liver transplantation for cirrhosis: outcome and predictors. *Liver Transpl* **15**, 522-529, doi:10.1002/lt.21730 (2009).

267 Massarweh, N. N., Legner, V. J., Symons, R. G., McCormick, W. C. & Flum, D. R. Impact of advancing age on abdominal surgical outcomes. *Archives of Surgery* **144**, 1108-1114, doi:10.1001/archsurg.2009.204 (2009).

268 Kheterpal, S. *et al.* Development and validation of an acute kidney injury risk index for patients undergoing general surgery: results from a national data set. *Anesthesiology* **110**, 505-515, doi:10.1097/ALN.0b013e3181979440 (2009).

269 Karanjia, N. D., Lordan, J. T., Fawcett, W. J., Quiney, N. & Worthington, T. R. Survival and recurrence after neo-adjuvant chemotherapy and liver resection for colorectal metastases - A ten year study. *European Journal of Surgical Oncology* **35**, 838-843, doi:10.1016/j.ejso.2008.09.017 (2009).

270 Hackworth, W. A. *et al.* Effect of hyponatraemia on outcomes following orthotopic liver transplantation. *Liver Int* **29**, 1071-1077, doi:10.1111/j.1478-3231.2009.01982.x (2009).

271 Duchesne, J. C. *et al.* Impact of obesity in damage control laparotomy patients. *J Trauma* **67**, 108-112; discussion 112-104, doi:10.1097/TA.0b013e3181a92ce0 (2009).

272 Charbonney, E. *et al.* Prognosis of acute kidney injury requiring renal replacement therapy in solid organ transplanted patients. *Transpl Int* **22**, 1058-1063, doi:10.1111/j.1432-2277.2009.00914.x (2009).

273 Nasr, S. H. *et al.* Oxalate nephropathy complicating Roux-en-Y Gastric Bypass: an underrecognized cause of irreversible renal failure. *Clin J Am Soc Nephrol* **3**, 1676-1683, doi:10.2215/cjn.02940608 (2008).

274 Afonso, R. C. *et al.* Impact of renal failure on liver transplantation survival. *Transplant Proc* **40**, 808-810, doi:10.1016/j.transproceed.2008.02.062 (2008).

275 Sizzi, O. *et al.* Italian multicenter study on complications of laparoscopic myomectomy. *J Minim Invasive Gynecol* **14**, 453-462, doi:10.1016/j.jmig.2007.01.013 (2007).

276 Robledo, F. A. *et al.* Open versus closed management of the abdomen in the surgical treatment of severe secondary peritonitis: a randomized clinical trial. *Surg Infect (Larchmt)* **8**, 63-72, doi:10.1089/sur.2006.8.016 (2007).

277 O'Riordan, A. *et al.* Acute renal disease, as defined by the RIFLE criteria, post-liver transplantation. *Am J Transplant* **7**, 168-176, doi:10.1111/j.1600-6143.2006.01602.x (2007).

278 Kheterpal, S. *et al.* Predictors of postoperative acute renal failure after noncardiac surgery in patients with previously normal renal function. *Anesthesiology* **107**, 892-902, doi:10.1097/01.anes.0000290588.29668.38 (2007).

279 Herrera, F. A. *et al.* The prevalence of obesity and postoperative complications in a Veterans Affairs Medical Center general surgery population. *American Surgeon* **73**, 1009-1012 (2007).

280 Aberg, F., Koivusalo, A. M., Höckerstedt, K. & Isoniemi, H. Renal dysfunction in liver transplant patients: comparing patients transplanted for liver tumor or acute or chronic disease. *Transpl Int* **20**, 591-599, doi:10.1111/j.1432-2277.2007.00482.x (2007).

281 Wei, Y. *et al.* Factors Related to Post-Liver Transplantation Acute Renal Failure. *Transplantation Proceedings* **38**, 2982-2984, doi:10.1016/j.transproceed.2006.08.156 (2006).

282 Londoño, M. C. *et al.* Hyponatremia impairs early posttransplantation outcome in patients with cirrhosis undergoing liver transplantation. *Gastroenterology* **130**, 1135-1143, doi:10.1053/j.gastro.2006.02.017 (2006).

283 Lobo, S. M. *et al.* Prospective, randomized trial comparing fluids and dobutamine optimization of oxygen delivery in high-risk surgical patients [ISRCTN42445141]. *Critical Care* **10**, doi:10.1186/cc4913 (2006).

284 Junge, G. *et al.* Acute renal failure after liver transplantation: incidence, etiology, therapy, and outcome. *Transplant Proc* **38**, 723-724, doi:10.1016/j.transproceed.2006.01.074 (2006).

285 Johnson, P. N. *et al.* Analysis of morbidity in liver transplant recipients following human albumin supplementation: a retrospective pilot study. *Prog Transplant* **16**, 197-205 (2006).

286 Guitard, J. *et al.* Acute renal failure following liver transplantation with induction therapy. *Clin Nephrol* **65**, 103-112, doi:10.5414/cnp65103 (2006).

287 Faenza, S. *et al.* Acute renal failure requiring renal replacement therapy after orthotopic liver transplantation. *Transplant Proc* **38**, 1141-1142, doi:10.1016/j.transproceed.2006.02.151 (2006).

288 Ellenberger, C. *et al.* Incidence, risk factors and prognosis of changes in serum creatinine early after aortic abdominal surgery. *Intensive Care Med* **32**, 1808-1816, doi:10.1007/s00134-006-0308-1 (2006).

289 Alamo, J. M. *et al.* Morbidity and mortality in liver retransplantation. *Transplant Proc* **38**, 2475-2477, doi:10.1016/j.transproceed.2006.08.056 (2006).

290 Acosta-Merida, M. A., Marchena-Gomez, J., Hemmersbach-Miller, M., Roque-Castellano, C. & Hernandez-Romero, J. M. Identification of risk factors for perioperative mortality in acute mesenteric ischemia. *World J Surg* **30**, 1579-1585, doi:10.1007/s00268-005-0560-5 (2006).

291 Lin, C. C. *et al.* The renal-sparing efficacy of basiliximab in adult living donor liver transplantation. *Liver Transpl* **11**, 1258-1264, doi:10.1002/lt.20520 (2005).

292 Wyatt, C. M. & Arons, R. R. The burden of acute renal failure in nonrenal solid organ transplantation. *Transplantation* **78**, 1351-1355, doi:10.1097/01.tp.0000140848.05002.b8 (2004).

293 Vemuri, C. *et al.* Effect of increasing patient age on complication rates following intact abdominal aortic aneurysm repair in the United States. *J Surg Res* **118**, 26-31, doi:10.1016/j.jss.2004.02.007 (2004).

294 Sanchez, E. Q. *et al.* Preoperative and perioperative predictors of the need for renal replacement therapy after orthotopic liver transplantation. *Transplantation* **78**, 1048-1054, doi:10.1097/01.tp.0000137176.95730.5b (2004).

295 Paramesh, A. S. *et al.* Post-liver transplant acute renal failure: factors predicting development of end-stage renal disease. *Clin Transplant* **18**, 94-99, doi:10.1046/j.1399-0012.2003.00132.x (2004).

296 Lebrón Gallardo, M. *et al.* Risk factors for renal dysfunction in the postoperative course of liver transplant. *Liver Transpl* **10**, 1379-1385, doi:10.1002/lt.20215 (2004).

297 Kim, S. G. *et al.* Incidence and risk factors of renal dysfunction after liver transplantation in Korea. *Transplant Proc* **36**, 2318-2320, doi:10.1016/j.transproceed.2004.06.042 (2004).

298 Khurana, R. N. *et al.* Postoperative rhabdomyolysis following laparoscopic gastric bypass in the morbidly obese. *Arch Surg* **139**, 73-76, doi:10.1001/archsurg.139.1.73 (2004).

299 Chuang, F. R. *et al.* Acute renal failure after cadaveric related liver transplantation. *Transplant Proc* **36**, 2328-2330, doi:10.1016/j.transproceed.2004.07.002 (2004).

300 Balachandran, P. *et al.* Haemorrhagic complications of pancreaticoduodenectomy. *ANZ J Surg* **74**, 945-950, doi:10.1111/j.1445-1433.2004.03212.x (2004).

301 Shen, Z. Y., Zheng, W. P. & Liu, Y. H. [Risk factors for renal failure during the early period after standard orthotopic liver transplantation without veno venous bypass]. *Zhongguo Wei Zhong Bing Ji Jiu Yi Xue* **18**, 397-399 (2006).

302 Zhu, F. X. *et al.* [Risk factors of renal failure in the early post-liver transplantation period]. *Zhonghua Gan Zang Bing Za Zhi* **13**, 168-170 (2005).

303 Yuan, C. H. *et al.* [The influential factors and clinical significance of acute renal failure complicated to orthotopic liver transplantation]. *Zhonghua Wai Ke Za Zhi* **49**, 1003-1006 (2011).

304 Sabaté, S., Gomar, C., Canet, J., Sierra, P. & Castillo, J. [Risk factors for postoperative acute kidney injury in a cohort of 2378 patients from 59 hospitals]. *Rev Esp Anestesiol Reanim* **58**, 548-555, doi:10.1016/s0034-9356(11)70139-6 (2011).

305 Nemes, B. *et al.* [Bacterial infection after orthotopic liver transplantation]. *Orv Hetil* **156**, 1366-1382, doi:10.1556/650.2015.30204 (2015).

306 Wang, Y., Liu, Y. H., Zheng, W. P., Ming, Y. & Shen, Z. Y. [Study on risk factors for early postoperative infection after orthotopic liver transplantation in adults]. *Zhongguo Wei Zhong Bing Ji Jiu Yi Xue* **18**, 406-408 (2006).

307 Seller-Pérez, G. *et al.* [Postoperative complications of liver transplantation: relationship with mortality]. *Med Clin (Barc)* **123**, 321-327, doi:10.1016/s0025-7753(04)74505-4 (2004).

308 Koscielny, A., Kühnel, M., Verrel, F. & Kalff, J. C. [Ruptured Abdominal Aortic Aneurysm - Results and Prognostic Factors at a Certified Centre of Vascular Surgery]. *Zentralbl Chir* **141**, 510-517, doi:10.1055/s-0042-105519 (2016).

309 Futier, E. *et al.* Effect of Hydroxyethyl Starch vs Saline for Volume Replacement Therapy on Death or Postoperative Complications Among High-Risk Patients Undergoing Major Abdominal Surgery: The FLASH Randomized Clinical Trial. *Jama* **323**, 225-236, doi:10.1001/jama.2019.20833 (2020).

310 Myles, P. S., McIlroy, D. R., Bellomo, R. & Wallace, S. Importance of intraoperative oliguria during major abdominal surgery: findings of the Restrictive versus Liberal Fluid Therapy in Major Abdominal Surgery trial. *Br J Anaesth* **122**, 726-733, doi:10.1016/j.bja.2019.01.010 (2019).

311 Myles, P. S. *et al.* Restrictive versus Liberal Fluid Therapy for Major Abdominal Surgery. *N Engl J Med* **378**, 2263-2274, doi:10.1056/NEJMoa1801601 (2018).

312 Dedinská, I., Mikolajčík, P., Skálová, P., Mokáň, M. & Laca, Ľ. Acute kidney injury after liver resection in elderly patients. *BMC Nephrol* **20**, 272, doi:10.1186/s12882-019-1449-0 (2019).

313 Boteon, A. P. C. S. *et al.* The impact of transarterial chemoembolization induced complications on outcomes after liver transplantation: A propensity-matched study. *Clinical Transplantation* **32**, doi:10.1111/ctr.13255 (2018).

314 Ripolles-Melchor, J. *et al.* Association Between Use of Enhanced Recovery After Surgery Protocol and Postoperative Complications in Colorectal Surgery: The Postoperative Outcomes Within Enhanced Recovery After Surgery Protocol (POWER) Study. *JAMA Surg* **154**, 725-736, doi:10.1001/jamasurg.2019.0995 (2019).

315 Zorrilla-Vaca, A. *et al.* Risk factors for acute kidney injury in an enhanced recovery pathway for colorectal surgery. *Surg Today* **51**, 537-544, doi:10.1007/s00595-020-02107-2 (2021).

316 Turan, A. *et al.* Mild Acute Kidney Injury after Noncardiac Surgery Is Associated with Long-term Renal Dysfunction: A Retrospective Cohort Study. *Anesthesiology* **132**, 1053-1061, doi:10.1097/ALN.0000000000003109 (2020).

317 Roberts, D. J. *et al.* Angiotensin-Converting Enzyme Inhibitor/Receptor Blocker, Diuretic, or Nonsteroidal Anti-inflammatory Drug Use After Major Surgery and Acute Kidney Injury: A Case-Control Study. *J Surg Res* **263**, 34-43, doi:10.1016/j.jss.2021.01.019 (2021).

318 Gocze, I. *et al.* Biomarker-guided Intervention to Prevent Acute Kidney Injury After Major Surgery: The Prospective Randomized BigpAK Study. *Ann Surg* **267**, 1013-1020, doi:10.1097/SLA.0000000000002485 (2018).

319 Trongtrakul, K. *et al.* External Validation of the Acute Kidney Injury Risk Prediction Score for Critically Ill Surgical Patients Who Underwent Major Non-Cardiothoracic Surgery. *Healthcare (Basel)* **9**, doi:10.3390/healthcare9020209 (2021).

320 Argalious, M. Y., Dalton, J. E., Sreenivasalu, T., O'Hara, J. & Sessler, D. I. The association of preoperative statin use and acute kidney injury after noncardiac surgery. *Anesth Analg* **117**, 916-923, doi:10.1213/ANE.0b013e31828175ab (2013).

321 Argalious, M. Y., Mao, G., Davison, R. K., Chow, C. & Bhavani, S. Association of Intraoperative Tidal Volumes and Acute Kidney Injury After Noncardiac Surgery. *Anesth Analg* **130**, 925-932, doi:10.1213/ane.0000000000004254 (2020).

322 Shim, J. W. *et al.* Impact of intraoperative zero-balance fluid therapy on the occurrence of acute kidney injury in patients who had undergone colorectal cancer resection within an enhanced recovery after surgery protocol: a propensity score matching analysis. *Int J Colorectal Dis* **35**, 1537-1548, doi:10.1007/s00384-020-03616-9 (2020).

323 Shim, J. W. *et al.* Male Patients may be More Vulnerable to Acute Kidney Injury After Colorectal Surgery in an Enhanced Recovery Program: A Propensity Score Matching Analysis. *World J Surg* **45**, 1642-1651, doi:10.1007/s00268-021-06041-3 (2021).

324 Shim, J. W. *et al.* Role of intraoperative oliguria in risk stratification for postoperative acute kidney injury in patients undergoing colorectal surgery with an enhanced recovery protocol: A propensity score matching analysis. *PLoS One* **15**, e0231447, doi:10.1371/journal.pone.0231447 (2020).

325 Slankamenac, K. *et al.* Development and validation of a prediction score for postoperative acute renal failure following liver resection. *Ann Surg* **250**, 720-728, doi:10.1097/SLA.0b013e3181bdd840 (2009).

326 Slankamenac, K., Beck-Schimmer, B., Breitenstein, S., Puhan, M. A. & Clavien, P. A. Novel prediction score including pre- and intraoperative parameters best predicts acute kidney injury after liver surgery. *World J Surg* **37**, 2618-2628, doi:10.1007/s00268-013-2159-6 (2013).

327 Mizota, T. *et al.* Transient acute kidney injury after major abdominal surgery increases chronic kidney disease risk and 1-year mortality. *J Crit Care* **50**, 17-22, doi:10.1016/j.jcrc.2018.11.008 (2019).

328 Mizota, T. *et al.* Intraoperative oliguria predicts acute kidney injury after major abdominal surgery. *Br J Anaesth* **119**, 1127-1134, doi:10.1093/bja/aex255 (2017).

329 Oh, T. K., Han, S., Oh, A. Y., Kim, S. & Ryu, J. H. Chronic hyperglycemia with elevated glycated hemoglobin level and its association with postoperative acute kidney injury after a major laparoscopic abdominal surgery in diabetes patients. *J Anesth* **32**, 740-747, doi:10.1007/s00540-018-2551-3 (2018).

330 Oh, T. K. *et al.* Retrospective analysis of the association between intraoperative magnesium sulfate infusion and postoperative acute kidney injury after major laparoscopic abdominal surgery. *Sci Rep* **9**, 2833, doi:10.1038/s41598-019-39106-4 (2019).

331 Hassinger, T. E. *et al.* Acute Kidney Injury in the Age of Enhanced Recovery Protocols. *Dis Colon Rectum* **61**, 946-954, doi:10.1097/dcr.0000000000001059 (2018).

332 Hassinger, T. E. *et al.* Ureteral stents increase risk of postoperative acute kidney injury following colorectal surgery. *Surg Endosc* **32**, 3342-3348, doi:10.1007/s00464-018-6054-y (2018).

333 Gameiro, J. *et al.* Neutrophil, lymphocyte and platelet ratio as a predictor of postoperative acute kidney injury in major abdominal surgery. *BMC Nephrol* **19**, 320, doi:10.1186/s12882-018-1073-4 (2018).

334 Teixeira, C. *et al.* Acute kidney injury after major abdominal surgery: a retrospective cohort analysis. *Crit Care Res Pract* **2014**, 132175, doi:10.1155/2014/132175 (2014).

335 Collaborative, S. Perioperative intravenous contrast administration and the incidence of acute kidney injury after major gastrointestinal surgery: prospective, multicentre cohort study. *Br J Surg* **107**, 1023-1032, doi:10.1002/bjs.11453 (2020).

336 Collaborative, S. Association between peri-operative angiotensin-converting enzyme inhibitors and angiotensin-2 receptor blockers and acute kidney injury in major elective non-cardiac surgery: a multicentre, prospective cohort study. *Anaesthesia* **73**, 1214-1222, doi:10.1111/anae.14349 (2018).

337 Argalious, M. Y., Makarova, N., Leone, A., Cywinski, J. & Farag, E. Association of body mass index and postoperative acute kidney injury in patients undergoing laparoscopic surgery. *Ochsner Journal* **17**, 224-232, doi:10.1043/1524-5012-17.3.224 (2017).

338 Weingarten, T. N. *et al.* Nonalcoholic steatohepatitis (NASH) does not increase complications after laparoscopic bariatric surgery. *Obes Surg* **21**, 1714-1720, doi:10.1007/s11695-011-0521-z (2011).

339 Weingarten, T. N. *et al.* Acute kidney injury following bariatric surgery. *Obes Surg* **23**, 64-70, doi:10.1007/s11695-012-0766-1 (2013).

340 Gameiro, J. *et al.* Transient and Persistent AKI and Outcomes in Patients Undergoing Major Abdominal Surgery. *Nephron* **144**, 236-244, doi:10.1159/000506397 (2020).

341 Xie, M., Li, N., Qiao, H., Guo, J. F. & Li, S. L. [Acute kidney injury diagnosed by elevated serum creatinine increases mortality in ICU patients following non-cardiac surgery]. *Zhonghua Yi Xue Za Zhi* **100**, 3285-3290, doi:10.3760/cma.j.cn112137-20200318-00824 (2020).

342 Li, N. *et al.* Preoperative hypoalbuminemia was associated with acute kidney injury in high-risk patients following non-cardiac surgery: a retrospective cohort study. *BMC Anesthesiol* **19**, 171, doi:10.1186/s12871-019-0842-3 (2019).

343 de Moura Pedro, R. A. *et al.* Postoperative oliguria after intermediate and high-risk surgeries in critical care, A cohort analysis. *J Crit Care* **85**, 154976, doi:10.1016/j.jcrc.2024.154976 (2025).

344 Zhao, B. C. *et al.* Postoperative haemoglobin and anaemia-associated ischaemic events after major noncardiac surgery: A sex-stratified cohort study. *J Clin Anesth* **95**, 111439, doi:10.1016/j.jclinane.2024.111439 (2024).

345 Yagyu, T. *et al.* Impact of the Diverting Stoma on Renal Function. *Dis Colon Rectum* **67**, 1576-1583, doi:10.1097/dcr.0000000000003517 (2024).

346 Wubet, H. B. *et al.* The incidence and factors associated with anemia in elective surgical patients admitted to a surgical intensive care unit: a retrospective cohort study. *Eur J Med Res* **29**, 290, doi:10.1186/s40001-024-01887-4 (2024).

347 Walco, J. P. *et al.* Association between Preoperative Blood Pressures and Postoperative Adverse Events. *Anesthesiology* **141**, 272-285, doi:10.1097/aln.0000000000004991 (2024).

348 Paredes, S. *et al.* Metformin Use in Type 2 Diabetics and Delirium After Noncardiac Surgery: A Retrospective Cohort Analysis. *Anesth Analg* **138**, 1304-1312, doi:10.1213/ane.0000000000006863 (2024).

349 Meersch, M. *et al.* Acute kidney disease beyond day 7 after major surgery: a secondary analysis of the EPIS-AKI trial. *Intensive Care Med* **50**, 247-257, doi:10.1007/s00134-023-07314-2 (2024).

350 Kobata, M. *et al.* Associations between intraoperative nociceptive response index and early postoperative acute kidney injury in patients undergoing non-cardiac surgery under general anesthesia: a single-center retrospective cohort study. *J Clin Monit Comput* **38**, 1297-1304, doi:10.1007/s10877-024-01184-9 (2024).

351 Jung, J. Y. *et al.* Association between intraoperative tidal volume and postoperative acute kidney injury in non-cardiac surgical patients using a propensity score-weighted analysis. *Sci Rep* **14**, 20079, doi:10.1038/s41598-024-71134-7 (2024).

352 Wu, J., Li, J., Chen, H., Shang, X. & Yu, R. Optimization of central venous pressure during the perioperative period is associated with improved prognosis of high-risk operation patients. *J Intensive Med* **3**, 165-170, doi:10.1016/j.jointm.2022.06.003 (2023).

353 Wang, J., Bi, Y., Ma, J., He, Y. & Liu, B. Association of Preoperative Neutrophil-to-Lymphocyte Ratio with Postoperative Acute Kidney Injury and Mortality Following Major Noncardiac Surgeries. *World J Surg* **47**, 948-961, doi:10.1007/s00268-022-06878-2 (2023).

354 Sun, Q. *et al.* Risk factors of perioperative acute kidney injury in elderly patients: a single-center retrospective study. *Int Urol Nephrol* **55**, 459-467, doi:10.1007/s11255-022-03345-8 (2023).

355 Solares, G. J. *et al.* Real-world outcomes of the hypotension prediction index in the management of intraoperative hypotension during non-cardiac surgery: a retrospective clinical study. *J Clin Monit Comput* **37**, 211-220, doi:10.1007/s10877-022-00881-7 (2023).

356 Ravetti, C. G. *et al.* Impact of bedside ultrasound to reduce the incidence of acute renal injury in high-risk surgical patients: a randomized clinical trial. *J Ultrasound* **26**, 449-457, doi:10.1007/s40477-022-00730-y (2023).

357 Park, C. H. & Lee, J. W. Current status of nutritional provision and effects of nutritional support on the clinical outcomes of acute kidney injury requiring continuous renal replacement therapy in the surgical intensive care unit. *Asia Pac J Clin Nutr* **32**, 321-329, doi:10.6133/apjcn.202309_32(3).0003 (2023).

358 Moneme, A. N., Wirtalla, C. J., Roberts, S. E., Keele, L. J. & Kelz, R. R. Primary Care Physician Follow-Up and 30-Day Readmission After Emergency General Surgery Admissions. *JAMA Surg* **158**, 1293-1301, doi:10.1001/jamasurg.2023.4534 (2023).

359 Huang, W. K. *et al.* Association between cumulative duration of deep anesthesia and postoperative acute kidney injury after noncardiac surgeries: a retrospective observational study. *Ren Fail* **45**, 2287130, doi:10.1080/0886022x.2023.2287130 (2023).

360 Zhou, Y. & Liu, S. Threshold heterogeneity of perioperative hemoglobin drop for acute kidney injury after noncardiac surgery: a propensity score weighting analysis. *BMC Nephrol* **23**, 206, doi:10.1186/s12882-022-02834-3 (2022).

361 Tang, Y. *et al.* Preoperative Neutrophil-Lymphocyte Ratio for predicting surgery-related acute kidney injury in non-cardiac surgery patients under general anaesthesia: A retrospective cohort study. *PLoS One* **17**, e0270066, doi:10.1371/journal.pone.0270066 (2022).

362 Strand, H., Elshaug, A. C., Bernersen, Ø. & Ballangrud, R. Effectiveness of the advisory display SmartPilot® view in the assessment of anesthetic depth in low risk gynecological surgery patients: a randomized controlled trial. *BMC Anesthesiol* **22**, 57, doi:10.1186/s12871-022-01593-w (2022).

363 Schneck, E. *et al.* Delta-like canonical Notch ligand 1 is predictive for sepsis and acute kidney injury in surgical intensive care patients. *Sci Rep* **12**, 13355, doi:10.1038/s41598-022-17778-9 (2022).

364 Sarna, M. J., Giorgi, M. & Luhrs, A. R. Metabolic syndrome as a predictor of perioperative outcomes in primary bariatric surgery, a MBSAQIP survey. *Surg Endosc* **36**, 6122-6128, doi:10.1007/s00464-021-08954-5 (2022).

365 Park, M., Jung, K., Cho, H. S. & Min, J. J. Renal injury from sevoflurane in noncardiac surgery: a retrospective cohort study. *Br J Anaesth* **129**, 182-190, doi:10.1016/j.bja.2022.04.026 (2022).

366 McIlroy, D. R. *et al.* Oxygen administration during surgery and postoperative organ injury: observational cohort study. *Bmj* **379**, e070941, doi:10.1136/bmj-2022-070941 (2022).

367 Li, N. *et al.* Association between urine microscopy and severe acute kidney injury in critically ill patients following non-cardiac surgery: a prospective cohort study. *Ann Palliat Med* **11**, 2327-2337, doi:10.21037/apm-21-3085 (2022).

368 Goeddel, L. A. *et al.* Association Between Left Ventricular Relative Wall Thickness and Acute Kidney Injury After Noncardiac Surgery. *Anesth Analg* **135**, 605-616, doi:10.1213/ane.0000000000006055 (2022).

369 Chi, D. X. *et al.* [Effects of postoperative urine protein levels on predicting acute kidney injury in critically ill patients undergoing non-cardiac surgery]. *Zhonghua Yi Xue Za Zhi* **102**, 336-343, doi:10.3760/cma.j.cn112137-20210719-01607 (2022).

370 Ariyarathna, D. *et al.* Intraoperative vasopressor use and early postoperative acute kidney injury in elderly patients undergoing elective noncardiac surgery. *Ren Fail* **44**, 648-659, doi:10.1080/0886022x.2022.2061997 (2022).

371 Wanner, P. M. *et al.* Targeting Higher Intraoperative Blood Pressures Does Not Reduce Adverse Cardiovascular Events Following Noncardiac Surgery. *J Am Coll Cardiol* **78**, 1753-1764, doi:10.1016/j.jacc.2021.08.048 (2021).

372 Nishimoto, M. *et al.* External Validation of a Prediction Model for Acute Kidney Injury Following Noncardiac Surgery. *JAMA Netw Open* **4**, e2127362, doi:10.1001/jamanetworkopen.2021.27362 (2021).

373 McArthur, K. *et al.* Trauma and nontrauma damage-control laparotomy: The difference is delirium (data from the Eastern Association for the Surgery of Trauma SLEEP-TIME multicenter trial). *J Trauma Acute Care Surg* **91**, 100-107, doi:10.1097/ta.0000000000003210 (2021).

374 Hardy, P. Y. *et al.* Impact of Preoperative Anemia on Outcomes of Enhanced Recovery Program After Colorectal Surgery: A Monocentric Retrospective Study. *World J Surg* **45**, 2326-2336, doi:10.1007/s00268-021-06161-w (2021).

375 Han, B. L., Wang, Y. M. & Xue, Y. W. [Effect of preoperative serum alanine aminotransferase and asparagine aminotransferase ratio on prognosis of patients with gastric cancer]. *Zhonghua Wei Chang Wai Ke Za Zhi* **23**, 65-70, doi:10.3760/cma.j.issn.1671-0274.2020.01.011 (2020).

376 Magalhães, D. S. C. *et al.* Analyzing the Impact of Bariatric Surgery in Kidney Function: a 2-Year Observational Study. *Obes Surg* **29**, 197-206, doi:10.1007/s11695-018-3508-1 (2019).

377 Passot, G. *et al.* A Perioperative Clinical Pathway Can Dramatically Reduce Failure-to-rescue Rates After Cytoreductive Surgery for Peritoneal Carcinomatosis: A Retrospective Study of 666 Consecutive Cytoreductions. *Ann Surg* **265**, 806-813, doi:10.1097/sla.0000000000001723 (2017).

378 Masoomi, H. *et al.* Predictive factors of in-hospital mortality in colon and rectal surgery. *J Am Coll Surg* **215**, 255-261, doi:10.1016/j.jamcollsurg.2012.04.019 (2012).

379 Masoomi, H. *et al.* Predictive factors of early bowel obstruction in colon and rectal surgery: data from the Nationwide Inpatient Sample, 2006-2008. *J Am Coll Surg* **214**, 831-837, doi:10.1016/j.jamcollsurg.2012.01.044 (2012).

380 Zhou Done, J., Ostertag-Hill, C. A., Ziegler, O. & Vithiananthan, S. Major Perioperative Bleeding in Patients on Dialysis Undergoing Nonelective Abdominal Surgeries. *J Surg Res* **305**, 356-366, doi:10.1016/j.jss.2024.11.029 (2025).

381 Kelly, N. J. *et al.* Total Gastrectomy for Gastric Malignancy: Trends Over 15 Years in Major Morbidity, Mortality, and Patient Selection From The National Surgical Quality Improvement Program. *J Surg Oncol* **131**, 624-629, doi:10.1002/jso.27990 (2025).

382 Elkbuli, A. *et al.* National Analysis of Clinical Outcomes Associated With Cirrhotic Blunt Trauma Patients Undergoing Emergency Laparotomy Versus Non-operative Management: A Propensity Case-Matched Analysis. *Am Surg* **91**, 336-344, doi:10.1177/00031348241256078 (2025).

383 Weiser, L. & Lin, M. Y. C. Unplanned hospital readmission after cholecystectomy in adults with cerebral palsy. *Surg Endosc* **38**, 6597-6604, doi:10.1007/s00464-024-11224-9 (2024).

384 Roth, S. *et al.* Cardiovascular-Kidney-Metabolic Syndrome: Association with Adverse Events After Major Noncardiac Surgery. *Anesth Analg* **139**, 679-681, doi:10.1213/ane.0000000000006975 (2024).

385 Miyamoto, K. *et al.* Age-related changes in intraoperative mean values of nociceptive response in patients undergoing non-cardiac surgery under general anesthesia: A retrospective cohort study. *J Clin Monit Comput* **38**, 581-589, doi:10.1007/s10877-023-01125-y (2024).

386 Lorenz, W. R. *et al.* Factors Associated With Respiratory Failure After Open Ventral Hernia Repair: An Evaluation of the NSQIP Database. *Am Surg* **90**, 1916-1918, doi:10.1177/00031348241241731 (2024).

387 Liu, R. *et al.* Incidence and risk factors of postoperative delirium following hepatic resection: a retrospective national inpatient sample database study. *BMC Surg* **24**, 151, doi:10.1186/s12893-024-02436-w (2024).

388 Jehan, F. S. *et al.* Does the Surgical Approach Affect the Incidence of Post-Hepatectomy Liver Failure in Cirrhotic Patients? An Analysis of the NSQIP Database. *Am Surg* **90**, 2901-2906, doi:10.1177/00031348241246175 (2024).

389 Elkbuli, A. *et al.* Evaluating Mortality Outcomes, Transfusion Characteristics, and Risk Factors Associated With Cirrhotic Trauma Patients Undergoing Emergency Laparotomy Versus Non-Operative Management: A National Analysis. *Am Surg* **90**, 1347-1356, doi:10.1177/00031348241230087 (2024).

390 Brovman, E. Y. *et al.* Relationship Between Newly Established Perioperative DNR Status and Perioperative Outcomes in the Elderly Population: A NSQIP Database Analysis. *J Palliat Care* **39**, 97-104, doi:10.1177/0825859720944746 (2024).

391 Biesel, E. A. *et al.* Surgical complications requiring late surgical revisions after pancreatoduodenectomy increase postoperative morbidity and mortality. *Scand J Surg* **113**, 88-97, doi:10.1177/14574969231206132 (2024).

392 Assaf, R. *et al.* Racial Disparities in Outcomes of Bariatric Surgery: An Analysis of 190,319 Patients. *Jsls* **28**, doi:10.4293/jsls.2024.00042 (2024).

393 Turcotte, J. J., Allen, R. S., Klune, J. R. & Feather, C. B. Open and Closed Approaches to Skin Closure After Nonelective Open Colorectal Operations. *Am Surg* **89**, 2520-2528, doi:10.1177/00031348221101578 (2023).

394 Tanos, P. *et al.* SHARP risk score: A predictor of poor outcomes in adults admitted for emergency general surgery: A prospective cohort study. *Asian J Surg* **46**, 2668-2674, doi:10.1016/j.asjsur.2022.10.049 (2023).

395 Soltanizadeh, S., Jensen, K. K., Nordklint, A. K., Jørgensen, H. L. & Jørgensen, L. N. Even minor alteration of plasma creatinine after open abdominal surgery is associated with 30-day mortality: A single-centre cohort study. *J Visc Surg* **160**, 19-26, doi:10.1016/j.jviscsurg.2021.10.008 (2023).

396 Saitta, C. *et al.* Development of a novel score (RENSAFE) to determine probability of acute kidney injury and renal functional decline post surgery: A multicenter analysis. *Urol Oncol* **41**, 487.e415-487.e423, doi:10.1016/j.urolonc.2023.09.015 (2023).

397 Rutegård, M. *et al.* Defunctioning loop ileostomy in anterior resection for rectal cancer and subsequent renal failure: nationwide population-based study. *BJS Open* **7**, doi:10.1093/bjsopen/zrad010 (2023).

398 Omar, M. *et al.* Risk factors of acute renal failure in patients with protective ileostomy after rectal cancer surgery. *BMC Surg* **23**, 107, doi:10.1186/s12893-023-02016-4 (2023).

399 Nicholson, J. J. *et al.* Perioperative outcomes in intermediate and high-risk patients after major surgery following introduction of a dedicated perioperative medicine team: A single centre cohort study. *Anaesth Intensive Care* **51**, 120-129, doi:10.1177/0310057x221119814 (2023).

400 Maurer, L. R. *et al.* Validation of the Al-based Predictive OpTimal Trees in Emergency Surgery Risk (POTTER) Calculator in Patients 65 Years and Older. *Ann Surg* **277**, e8-e15, doi:10.1097/sla.0000000000004714 (2023).

401 Connelly, T. M. *et al.* Outcomes of clostridioides difficile infection on inflammatory bowel disease patients undergoing colonic resection: A propensity score weighted NSQIP analysis. *Am J Surg* **225**, 553-557, doi:10.1016/j.amjsurg.2022.10.061 (2023).

402 Walther, F. *et al.* Relationships between multiple patient safety outcomes and healthcare and hospital-related risk factors in colorectal resection cases: cross-sectional evidence from a nationwide sample of 232 German hospitals. *BMJ Open* **12**, e058481, doi:10.1136/bmjopen-2021-058481 (2022).

403 Tessman, D., Chou, J., Shebrain, S. & Munene, G. Surgical Outcomes of Distal Pancreatectomy in Elderly Patients. *Am Surg* **88**, 115-119, doi:10.1177/0003134820982574 (2022).

404 Sheriff, S. *et al.* Predictors of mortality within the first year post-hepatectomy for hepatocellular carcinoma. *J Egypt Natl Canc Inst* **34**, 14, doi:10.1186/s43046-022-00113-8 (2022).

405 Sharon, C. E. *et al.* Fourteen years of pancreatic surgery for malignancy among ACS-NSQIP centers: Trends in major morbidity and mortality. *Surgery* **172**, 708-714, doi:10.1016/j.surg.2022.03.030 (2022).

406 Mihailov, R. *et al.* Nomogram for Prediction of Postoperative Morbidity in Patients with Colon Cancer Requiring Emergency Therapy. *Med Sci Monit* **28**, e936303, doi:10.12659/msm.936303 (2022).

407 Kassahun, W. T., Babel, J. & Mehdorn, M. Assessing differences in surgical outcomes following emergency abdominal exploration for complications of elective surgery and high-risk primary emergencies. *Sci Rep* **12**, 1349, doi:10.1038/s41598-022-05326-4 (2022).

408 Karamchandani, K. *et al.* Failure to Rescue After Severe Acute Kidney Injury in Patients Undergoing Non-Cardiac Surgery. *J Surg Res* **279**, 148-163, doi:10.1016/j.jss.2022.05.018 (2022).

409 Huckaby, L. V. *et al.* Accuracy of Risk Estimation for Surgeons Versus Risk Calculators in Emergency General Surgery. *J Surg Res* **278**, 57-63, doi:10.1016/j.jss.2022.04.042 (2022).

410 Hamade, S. *et al.* Evaluation of the American College of Surgeons National Surgical Quality Improvement Program Risk Calculator to predict outcomes after hysterectomies. *Int J Gynaecol Obstet* **158**, 714-721, doi:10.1002/ijgo.14075 (2022).

411 Ellenberger, C. *et al.* Myocardial injury after major noncardiac surgery: A secondary analysis of a randomized controlled trial. *Surgery* **171**, 1626-1634, doi:10.1016/j.surg.2021.10.029 (2022).

412 Curran, S., Apruzzese, P., Kendall, M. C. & De Oliveira, G. The impact of hypoalbuminemia on postoperative outcomes after outpatient surgery: a national analysis of the NSQIP database. *Can J Anaesth* **69**, 1099-1106, doi:10.1007/s12630-022-02280-7 (2022).

413 Benk, M. S., Olcucuoğlu, E. & Kaya İ, O. Evaluation of complications after laparoscopic and open appendectomy by the American College of Surgeons National Surgical Quality Improvement Program surgical risk calculator. *Ulus Travma Acil Cerrahi Derg* **28**, 418-427, doi:10.14744/tjtes.2020.45808 (2022).

414 Argueta, P. P. *et al.* Thirty-Day Readmission After Bariatric Surgery: Causes, Effects on Outcomes, and Predictors. *Dig Dis Sci* **67**, 834-843, doi:10.1007/s10620-021-06934-2 (2022).

415 Seishima, R. *et al.* Safety and feasibility of laparoscopic surgery for elderly rectal cancer patients in Japan: a nationwide study. *BJS Open* **5**, doi:10.1093/bjsopen/zrab007 (2021).

416 Peng, F., Wang, M., Xie, Y., Xu, M. & Qin, R. Y. [Comparative study of short-term efficacy,effectiveness and safety at different stages of the laparoscopic pancreaticoduodenectomy learning curve]. *Zhonghua Wai Ke Za Zhi* **59**, 618-623, doi:10.3760/cma.j.cn112139-20210330-00147 (2021).

417 Oliver, J. B., Merchant, A. M. & Koneru, B. The Impact of Chronic Liver Disease on Postoperative Outcomes and Resource Utilization. *J Invest Surg* **34**, 617-626, doi:10.1080/08941939.2019.1676846 (2021).

418 Niemeläinen, S. *et al.* The Clinical Frailty Scale is a useful tool for predicting postoperative complications following elective colon cancer surgery at the age of 80 years and above: A prospective, multicentre observational study. *Colorectal Dis* **23**, 1824-1836, doi:10.1111/codi.15689 (2021).

419 Mierzwa, A. S. *et al.* Characterizing Timing of Postoperative Complications Following Elective Roux-en-Y gastric Bypass and Sleeve Gastrectomy. *Obes Surg* **31**, 4492-4501, doi:10.1007/s11695-021-05638-w (2021).

420 Kim, N. E. & Hall, J. F. Risk Factors for Readmission after Ileostomy Creation: an NSQIP Database Study. *J Gastrointest Surg* **25**, 1010-1018, doi:10.1007/s11605-020-04549-y (2021).

421 El Edelbi, M. *et al.* Comparing Emergent and Elective Colectomy Outcomes in Elderly Patients: A NSQIP Study. *Int J Surg Oncol* **2021**, 9990434, doi:10.1155/2021/9990434 (2021).

422 Wolf, J. H. *et al.* Preoperative Nutritional Status Predicts Major Morbidity After Primary Rectal Cancer Resection. *J Surg Res* **255**, 325-331, doi:10.1016/j.jss.2020.05.081 (2020).

423 Vos, E. L. *et al.* Performance of the American College of Surgeons NSQIP Surgical Risk Calculator for Total Gastrectomy. *J Am Coll Surg* **231**, 650-656, doi:10.1016/j.jamcollsurg.2020.09.023 (2020).

424 Sahara, K. *et al.* Evaluation of the ACS NSQIP Surgical Risk Calculator in Elderly Patients Undergoing Hepatectomy for Hepatocellular Carcinoma. *J Gastrointest Surg* **24**, 551-559, doi:10.1007/s11605-019-04174-4 (2020).

425 Kongpakwattana, K., Dilokthornsakul, P., Dhippayom, T. & Chaiyakunapruk, N. Clinical and economic burden of postsurgical complications of high-risk surgeries: a cohort study in Thailand. *J Med Econ* **23**, 1046-1052, doi:10.1080/13696998.2020.1787420 (2020).

426 Kaafarani, H. M. A. *et al.* Prospective validation of the Emergency Surgery Score in emergency general surgery: An Eastern Association for the Surgery of Trauma multicenter study. *J Trauma Acute Care Surg* **89**, 118-124, doi:10.1097/ta.0000000000002658 (2020).

427 Alzahrani, S. M., Ko, C. S. & Yoo, M. W. Validation of the ACS NSQIP Surgical Risk Calculator for Patients with Early Gastric Cancer Treated with Laparoscopic Gastrectomy. *J Gastric Cancer* **20**, 267-276, doi:10.5230/jgc.2020.20.e27 (2020).

428 Montes, F. R. *et al.* Association between emergency department length of stay and adverse perioperative outcomes in emergency surgery: a cohort study in two Colombian University hospitals. *BMC Emerg Med* **19**, 27, doi:10.1186/s12873-019-0241-6 (2019).

429 Gleeson, E. M. *et al.* Patient-specific predictors of failure to rescue after pancreaticoduodenectomy. *HPB (Oxford)* **21**, 283-290, doi:10.1016/j.hpb.2018.07.022 (2019).

430 Geller, A. D. *et al.* Relative Incremental Cost of Postoperative Complications of Esophagectomy. *Semin Thorac Cardiovasc Surg* **31**, 290-299, doi:10.1053/j.semtcvs.2018.10.010 (2019).

431 Chacon, E. *et al.* Effect of critical care complications on perioperative mortality and hospital length of stay after hepatectomy: A multicenter analysis of 21,443 patients. *Am J Surg* **218**, 151-156, doi:10.1016/j.amjsurg.2018.11.016 (2019).

432 Sharma, P., McCarty, T. R. & Njei, B. Impact of Bariatric Surgery on Outcomes of Patients with Inflammatory Bowel Disease: a Nationwide Inpatient Sample Analysis, 2004-2014. *Obes Surg* **28**, 1015-1024, doi:10.1007/s11695-017-2959-0 (2018).

433 Schlottmann, F., Strassle, P. D. & Patti, M. G. Surgery for benign esophageal disorders in the US: risk factors for complications and trends of morbidity. *Surg Endosc* **32**, 3675-3682, doi:10.1007/s00464-018-6102-7 (2018).

434 Ramanathan, R., Mason, T., Wolfe, L. G. & Kaplan, B. J. Predictors of Short-Term Readmission After Pancreaticoduodenectomy. *J Gastrointest Surg* **22**, 998-1006, doi:10.1007/s11605-018-3700-6 (2018).

435 McCarty, T. R., Echouffo-Tcheugui, J. B., Lange, A., Haque, L. & Njei, B. Impact of bariatric surgery on outcomes of patients with nonalcoholic fatty liver disease: a nationwide inpatient sample analysis, 2004-2012. *Surg Obes Relat Dis* **14**, 74-80, doi:10.1016/j.soard.2017.09.511 (2018).

436 Fletcher, R., Deal, R., Kubasiak, J., Torquati, A. & Omotosho, P. Predictors of Increased Length of Hospital Stay Following Laparoscopic Sleeve Gastrectomy from the National Surgical Quality Improvement Program. *J Gastrointest Surg* **22**, 274-278, doi:10.1007/s11605-017-3642-4 (2018).

437 El Amrani, M. *et al.* Failure-to-rescue in Patients Undergoing Pancreatectomy: Is Hospital Volume a Standard for Quality Improvement Programs? Nationwide Analysis of 12,333 Patients. *Ann Surg* **268**, 799-807, doi:10.1097/sla.0000000000002945 (2018).

438 Ammori, J. B., Navale, S., Schiltz, N. & Koroukian, S. M. Predictors of 30-day readmissions after gastrectomy for malignancy. *J Surg Res* **224**, 176-184, doi:10.1016/j.jss.2017.12.004 (2018).

439 Whealon, M. D., Blondet, J. J., Gahagan, J. V., Phelan, M. J. & Nguyen, N. T. Volume and outcomes relationship in laparoscopic diaphragmatic hernia repair. *Surg Endosc* **31**, 4224-4230, doi:10.1007/s00464-017-5482-4 (2017).

440 Watanabe, T. *et al.* Prediction model for complications after low anterior resection based on data from 33,411 Japanese patients included in the National Clinical Database. *Surgery* **161**, 1597-1608, doi:10.1016/j.surg.2016.12.011 (2017).

441 Wang, J. B. *et al.* Effect of comorbidities on postoperative complications in patients with gastric cancer after laparoscopy-assisted total gastrectomy: results from an 8-year experience at a large-scale single center. *Surg Endosc* **31**, 2651-2660, doi:10.1007/s00464-016-5279-x (2017).

442 Kikuchi, H. *et al.* Development and external validation of preoperative risk models for operative morbidities after total gastrectomy using a Japanese web-based nationwide registry. *Gastric Cancer* **20**, 987-997, doi:10.1007/s10120-017-0706-9 (2017).

443 Gupta, R. *et al.* Impact of intraoperative blood loss on the short-term outcomes of laparoscopic liver resection. *Surg Endosc* **31**, 4451-4457, doi:10.1007/s00464-017-5496-y (2017).

444 Yokoo, H. *et al.* Models predicting the risks of six life-threatening morbidities and bile leakage in 14,970 hepatectomy patients registered in the National Clinical Database of Japan. *Medicine (Baltimore)* **95**, e5466, doi:10.1097/md.0000000000005466 (2016).

445 Rivard, C. *et al.* Evaluation of the performance of the ACS NSQIP surgical risk calculator in gynecologic oncology patients undergoing laparotomy. *Gynecol Oncol* **141**, 281-286, doi:10.1016/j.ygyno.2016.02.015 (2016).

446 Newhook, T. E. *et al.* Morbidity and mortality of hepatectomy for benign liver tumors. *Am J Surg* **211**, 102-108, doi:10.1016/j.amjsurg.2015.06.010 (2016).

447 Murray, A. C., Mauro, C., Rein, J. & Kiran, R. P. 30-day mortality after elective colorectal surgery can reasonably be predicted. *Tech Coloproctol* **20**, 567-576, doi:10.1007/s10151-016-1503-x (2016).

448 Ichikawa, H. *et al.* Surgical and long-term outcomes following oesophagectomy in oesophageal cancer patients with comorbidity. *Int J Surg* **36**, 212-218, doi:10.1016/j.ijsu.2016.10.041 (2016).

449 Doyon, L., Moreno-Koehler, A., Ricciardi, R. & Nepomnayshy, D. Resident participation in laparoscopic Roux-en-Y gastric bypass: a comparison of outcomes from the ACS-NSQIP database. *Surg Endosc* **30**, 3216-3224, doi:10.1007/s00464-015-4627-6 (2016).

450 Chang, A. R. *et al.* Bariatric surgery is associated with improvement in kidney outcomes. *Kidney Int* **90**, 164-171, doi:10.1016/j.kint.2016.02.039 (2016).

451 Alsfasser, G. *et al.* Volume-outcome relationship in pancreatic surgery. *Br J Surg* **103**, 136-143, doi:10.1002/bjs.9958 (2016).

452 Szender, J. B. *et al.* Evaluation of the National Surgical Quality Improvement Program Universal Surgical Risk Calculator for a gynecologic oncology service. *Int J Gynecol Cancer* **25**, 512-520, doi:10.1097/igc.0000000000000378 (2015).

453 Kenig, J., Richter, P., Olszewska, U. & Żychiewicz, B. The prognostic role of comorbidities in older patients qualified for emergency abdominal surgery. *Pol Przegl Chir* **86**, 569-575, doi:10.1515/pjs-2015-0003 (2015).

454 Joseph, B. *et al.* Factors associated with failure-to-rescue in patients undergoing trauma laparotomy. *Surgery* **158**, 393-398, doi:10.1016/j.surg.2015.03.047 (2015).

455 Huang, C. *et al.* [Postoperative 30-day mortality may underestimate the risk of esophagectomy]. *Zhonghua Wei Chang Wai Ke Za Zhi* **18**, 897-900 (2015).

456 Friedell, M. L., Van Way, C. W., 3rd, Freyberg, R. W. & Almenoff, P. L. β-Blockade and Operative Mortality in Noncardiac Surgery: Harmful or Helpful? *JAMA Surg* **150**, 658-663, doi:10.1001/jamasurg.2015.86 (2015).

457 Sako, A. *et al.* Prevalence and in-hospital mortality of gastrostomy and jejunostomy in Japan: a retrospective study with a national administrative database. *Gastrointest Endosc* **80**, 88-96, doi:10.1016/j.gie.2013.12.006 (2014).

458 Mahdi, H., Goodrich, S., Lockhart, D., DeBernardo, R. & Moslemi-Kebria, M. Predictors of surgical site infection in women undergoing hysterectomy for benign gynecologic disease: a multicenter analysis using the national surgical quality improvement program data. *J Minim Invasive Gynecol* **21**, 901-909, doi:10.1016/j.jmig.2014.04.003 (2014).

459 Huebner, M., Hübner, M., Cima, R. R. & Larson, D. W. Timing of complications and length of stay after rectal cancer surgery. *J Am Coll Surg* **218**, 914-919, doi:10.1016/j.jamcollsurg.2013.12.042 (2014).

460 Ricciardi, R. *et al.* Which adverse events are associated with mortality and prolonged length of stay following colorectal surgery? *J Gastrointest Surg* **17**, 1485-1493, doi:10.1007/s11605-013-2224-3 (2013).

461 Nimeri, A. *et al.* Are results of bariatric surgery different in the Middle East? Early experience of an international bariatric surgery program and an ACS NSQIP outcomes comparison. *J Am Coll Surg* **216**, 1082-1088, doi:10.1016/j.jamcollsurg.2013.01.063 (2013).

462 Nandipati, K. *et al.* Factors predicting the increased risk for return to the operating room in bariatric patients: a NSQIP database study. *Surg Endosc* **27**, 1172-1177, doi:10.1007/s00464-012-2571-2 (2013).

463 Kang, C. Y. *et al.* Risk factors for anastomotic leakage after anterior resection for rectal cancer. *JAMA Surg* **148**, 65-71, doi:10.1001/2013.jamasurg.2 (2013).

464 Hardiman, K., Chang, E. T., Diggs, B. S. & Lu, K. C. Laparoscopic colectomy reduces morbidity and mortality in obese patients. *Surg Endosc* **27**, 2907-2910, doi:10.1007/s00464-013-2853-3 (2013).

465 de la Fuente, S. G., Bennett, K. M. & Scarborough, J. E. Functional status determines postoperative outcomes in elderly patients undergoing hepatic resections. *J Surg Oncol* **107**, 865-870, doi:10.1002/jso.23335 (2013).

466 Shaw, A. D. *et al.* Major complications, mortality, and resource utilization after open abdominal surgery: 0.9% saline compared to Plasma-Lyte. *Ann Surg* **255**, 821-829, doi:10.1097/SLA.0b013e31825074f5 (2012).

467 Finlayson, E., Zhao, S. & Varma, M. G. Outcomes after rectal cancer surgery in elderly nursing home residents. *Dis Colon Rectum* **55**, 1229-1235, doi:10.1097/DCR.0b013e318267bfe3 (2012).

468 Cone, M. M. *et al.* Effect of surgical approach on 30-day mortality and morbidity after elective colectomy: a NSQIP study. *J Gastrointest Surg* **16**, 1212-1217, doi:10.1007/s11605-012-1860-3 (2012).

469 Leichtle, S. W., Mouawad, N. J., Lampman, R., Singal, B. & Cleary, R. K. Does preoperative anemia adversely affect colon and rectal surgery outcomes? *J Am Coll Surg* **212**, 187-194, doi:10.1016/j.jamcollsurg.2010.09.013 (2011).

470 Carroll, J. E., Jr. *et al.* In-hospital mortality after resection of biliary tract cancer in the United States. *HPB (Oxford)* **12**, 62-67, doi:10.1111/j.1477-2574.2009.00129.x (2010).

471 Merkow, R. P., Bilimoria, K. Y., McCarter, M. D. & Bentrem, D. J. Effect of body mass index on short-term outcomes after colectomy for cancer. *J Am Coll Surg* **208**, 53-61, doi:10.1016/j.jamcollsurg.2008.08.032 (2009).

472 Kusano, T. *et al.* Predictors and prognostic significance of operative complications in patients with hepatocellular carcinoma who underwent hepatic resection. *Eur J Surg Oncol* **35**, 1179-1185, doi:10.1016/j.ejso.2009.04.008 (2009).

473 Almahmeed, T. *et al.* Morbidity of anastomotic leaks in patients undergoing Roux-en-Y gastric bypass. *Arch Surg* **142**, 954-957, doi:10.1001/archsurg.142.10.954 (2007).

474 Schroeder, R. A. *et al.* Predictive indices of morbidity and mortality after liver resection. *Ann Surg* **243**, 373-379, doi:10.1097/01.sla.0000201483.95911.08 (2006).

475 McCullough, P. A. *et al.* Cardiorespiratory fitness and short-term complications after bariatric surgery. *Chest* **130**, 517-525, doi:10.1378/chest.130.2.517 (2006).

Table S3. Main characteristics of included studies (for citations see main manuscript)

| Author | Year | Design | Operation | Inclusion Criteria | Exclusion Criteria | Age (year)  Mean (SD) or median (IQR) | Male (%) | AKI definition | No. of patients | No. with AKI | Incidence of AKI |
| --- | --- | --- | --- | --- | --- | --- | --- | --- | --- | --- | --- |
| Andrs Zorrilla‑Vaca | 2021 | Prospective cohort study | Colorectal surgery | Age ≥ 18 years, within an ERP | Nil | 67 (12) | 997 (60.3) | KDIGO | 1652 | 127 | 0.08 |
| Bing-Cheng Zhao | 2021 | Retrospective cohort study | General abdominal surgery | Age ≥ 18 years, with a serum creatinine and NT- proBNP measurement within 30 pre-operative days, at least one serum creatinine measurement within 7 days after surgery | Cardiac, vascular, urological, neurological, transplant or  obstetric surgery, surgery lasting <1 h, eGFR < 15 ml/min/1.73 m^2^, history of RRT or kidney transplantation, pre-operative AKI | Not stated by subgroup | Not stated by subgroup | KDIGO | 2534 | 228 | 0.09 |
|  |  |  | Gynecologic surgery |  |  | Not stated by subgroup | - |  | 101 | 4 | 0.04 |
| Jianjun Xu | 2021 | Retrospective cohort study | Hepatectomy | HCC, none of the participants were on treatment for HCC, and on preoperative steroids, aspirin, chemotherapy or other treatments | No HBV infection history, ruptured HCC, non-Barcelona clinic liver cancer 0 or A staged HCC, lack critical clinical data | 54.3 (15-84) | 424 (88.5) | KDIGO | 479 | 51 | 0.11 |
| Ji Hoon Sim | 2021 | Retrospective cohort study | Hepatectomy | Age ≥ 18 years, HCC, open hepatectomy | Age < 18 or ≥ 80 years, severe cardiopulmonary, CKD, received RRT, intervention in the urinary system during surgery, emergency surgery, incomplete data, missing PNI or serum creatinine values | 62.6 (11.8) | 512 (57.9) | KDIGO | 817 | 59 | 0.07 |
| Ji Hoon Sim | 2021 | Retrospective cohort study | Colorectal surgery | Age ≥ 18 years, colorectal cancer | Severe cardiopulmonary, CKD, received RRT, intervention pertaining to urethra, ureter,  or kidneys during during surgery, emergency surgery, incomplete data or laboratory values | 59.8 (11.2) | 2176 (61.4) | KDIGO | 3543 | 361 | 0.10 |
| Jung-Woo Shim | 2021 | Retrospective cohort study | Laparoscopic colorectal resection | Elective operation for colorectal cancer, consent to the ERAS protocol | Any critical illness compromising surgery, general anesthesia, emergency case, infection | 64 (12) | 236 (52.1) | KDIGO | 453 | 77 | 0.17 |
| Annika Kurreck | 2022 | Retrospective cohort study | CRS and HIPEC | Underwent CRS in combination with HIPEC including cisplatin (CDDP) | Did not receive  intraperitoneal CDDP | 57 (19-83) | 105 (44.1) | AKIN | 238 | 62 | 0.26 |
| Minjae Kim | 2021 | Retrospective cohort study | Abdominal surgery | Intra-abdominal surgery | Missing or incomplete AIMS, outpatient procedures, preoperative acute renal failure or dialysis, missing pre- or postoperative serum creatinine measurement | 56.2 (17.4) | 1112 (41.3) | KDIGO | 2691 | 234 | 0.09 |
| Bo Rim Kim | 2021 | Retrospective cohort study | Open major abdominal surgery | Age ≥ 18 years, open surgery, analyzed the data from the  first surgery, | Without pre- or postoperative serum creatinine data, history of ESRD, RRT, kidney transplantation, nephrectomy | 60.5 (13.7) | 2179 (60.3) | KDIGO | 3616 | 238 | 0.07 |
| Rita Inácio | 2021 | Retrospective cohort study | Elective major Abdominal surgery | Age ≥ 18years, elective major abdominal surgery | CKD received RRT, RRT performed 1 week before surgery, serum creatinine measurement < 2 times during hospital stay, emergency surgery, without intraoperative diuresis monitoring | 69.2 (14) | 96 (58.2) | KDIGO | 165 | 32 | 0.19 |
| Yan Zhou | 2020 | Retrospective cohort study | Digestive surgery | Elective non-cardiac non-kidney surgery, age ≥ 18 years, | Obstetric surgeries, local infiltration anesthesia, missing perioperative data, with more than one operation within a year (including reopening of surgical cases) | Not stated by subgroup | Not stated by subgroup | KDIGO | 20936 | 842 | 0.04 |
| Konlawij Trongtrakul | 2020 | Retrospective cohort study | Abdomen & colorectal surgery | Age ≥ 18 years, major non-cardiothoracic operations, admission to ICU | Admitted to the ICU for less than 24 h, admitted to the ICU due to medical rather  than surgical reasons | Not stated by subgroup | Not stated by subgroup | KDIGO | 2078 | 232 | 0.11 |
| Masatoshi Nishimoto | 2020 | Retrospective cohort study | Abdominal surgery | Non-cardiac surgery, general anesthesia | Obstetric or urological surgery, missing data, multiple surgeries during observation period, pre-operative dialysis | Not stated by subgroup | Not stated by subgroup | KDIGO | 998 | 102 | 0.10 |
| James G.D. Wiener | 2020 | Retrospective cohort study | Colorectal surgery | Colorectal surgery, undergoing ERAS | Trauma, emergent surgery, preoperative acute renal failure or the currently requiring or on dialysis, with no preoperative serum creatinine measurement within 30 days of operation | 57.1 (47.6-67.5) | 496 (47) | KDIGO | 1052 | 112 | 0.11 |
| Mitsuru Ida | 2020 | Retrospective cohort study | Pancreas surgery | Aged ≥ 20 years, underwent pancreatoduodenectomy and distal pancreatectomy with a diagnosis of malignant tumor | Aged < 20 years, end-stage renal disease, patients in whom surgery cannot be completed, underwent procedures other than pancreatoduodenectomy and distal pancreatectomy，patients with incomplete data | 72（66-76） | 177(64.8) | KDIGO | 273 | 21 | 0.08 |
| Zayan Mahmooth | 2020 | Retrospective cohort study | Pancreatoduodenectomy | Age ≥ 18 years, presenting  for elective pancreatoduodenectomy, with complete IOIVF and pre-operative weights | Prior pancreatic surgery or bypass, prolonged preoperative surgical care requiring admission, conditions which may alter baseline characteristics | 63.2 (11.8) | NA | KDIGO | 383 | 78 | 0.20 |
| Gaetan-Romain Joliat | 2020 | Retrospective cohort study | Liver surgery | Liver surgery, undergoing ERAS | Nil | 64 (54-69) | 110 (53) | KDIGO | 285 | 79 | 0.28 |
| Xiaowei Guo | 2020 | Randomized Controlled Trial | Gastrointestinal surgery | ASA grade 1-3, age > 65 years, BMI < 28 kg/cm^2^, gastrointestinal tumor resection, general anesthesia | Severe hepatorenal insufficiency, history of hyperthyroidism and pheochromocytoma, severe cardiocerebrovascular disease, hypertension grade III, hemodynamic instability, COPD，pulmonary hypertension, recent use of tricyclic antidepressants or monoamine oxidase inhibitors or use of NSAIDs in the past month, emergency operation | 71 (5.3) | 112 (69.1) | KDIGO | 162 | 21 | 0.13 |
| Nassiba Beghdadi | 2020 | Observational study | Emergent open colectomy | Ischemic colitis patients | Nil | 67.4 (13.7) | 32 (30) | KDIGO | 94 | 51 | 0.54 |
| Maged Y. Argalious | 2020 | Retrospective cohort study | Digestive surgery | ASA status I–IV, aged > 18 years, noncardiac surgery, duration of surgery ≥2 hours, endotracheal intubation and mechanical ventilation during surgery, minimum of 1 night of postoperative hospital stay | Local, regional anesthesia or under monitored anesthesia care, chronic kidney disease more severe than stage 4 or on dialysis or with estimated glomerular filtration rate <30 mL/min preoperatively | Not stated by subgroup | Not stated by subgroup | AKIN | 12753 | 1130 | 0.09 |
| Miho Murashima | 2019 | Retrospective cohort study | Abdominal surgery | Age ≥ 18years, non-cardiac surgery, general anesthesia | Urological surgery, obstetric surgery, without creatinine values within 1 month pre-operatively or 1 week post-operatively, undergone dialysis pre-operatively | Not stated by subgroup | Not stated by subgroup | KDIGO | 889 | 89 | 0.10 |
| Hannan Chaudery | 2019 | Observational study | Abdominal surgery | Adult, elective in-patient surgery | Day-case, radiological, emergency procedures | Not stated by subgroup | Not stated by subgroup | KDIGO | 6501 | 174 | 0.03 |
| Qin Wu | 2019 | Retrospective cohort study | Abdominal surgery | Age ≥80 years, discharged from the ICU during the observation period | non-postsurgical ICU admission, ICU stay <24 h, insufficient data for analysis, occurrence of AKI prior to surgery, established of CKD before ICU admission. | Not stated by subgroup | Not stated by subgroup | KDIGO | 141 | 15 | 0.11 |
| Charlotte Slagelse | 2019 | Retrospective cohort study | Colorectal cancer surgery | Colorectal cancer surgery | Chronic RRT within 30 days before surgery, explorative-only proce- dure, no follow-up data were available | 69.5 (11.2) | 5291 (53) | KDIGO | 9932 | 2000 | 0.20 |
| Tak Kyu oh | 2019 | Retrospective cohort study | Major laparoscopic abdominal surgery | Major laparoscopic abdominal surgery | Emergency surgery, single-port laparoscopy, discharged by postoperative day 3, renal replacement therapy during the preoperative period, nephrectomy or nephroureterectomy, simple appendectomy or cholecystectomy, incomplete medical records, intraoperative open conversion | 60.7 (13) | 2427 (63.4) | KDIGO | 3828 | 186 | 0.05 |
| Shengnan Li | 2019 | Retrospective cohort study | Abdominal surgery | ICU patients, major surgery (cranium, chest, abdomen, or pelvis) | Cardiac surgery, emergent surgery, transplantation, renal replacement therapy or end-stage renal disease before surgery | Not stated by subgroup | Not stated by subgroup | KDIGO | 1990 | 1195 | 0.60 |
| Nan Li | 2019 | Retrospective cohort study | Abdominal surgery | Age ≥ 18 years, non-cardiac surgery, admitted to the surgical intensive care unit, at a high risk of postoperative AKI | Chronic kidney disease stage 5 or requiring long-term dialysis, surgery involving kidney, AKI events before surgery; incomplete clinical data | Not stated by subgroup | Not stated by subgroup | KDIGO | 330 | 101 | 0.31 |
|  |  |  | Gynecologic surgery |  |  | Not stated by subgroup | - |  | 25 | 8 | 0.32 |
| Csaba Kopitko | 2019 | Prospective cohort study | Major abdominal surgery | A minimum of 48-h ICU postoperative therapy | Age < 18years, end stage kidney disease, suprarenal cross-clamping, undergoing kidney, urinary bladder operations | 68 (13.9) | 49 (58.3) | AKIN | 84 | 39 | 0.46 |
| Crystal P. Koerner | 2019 | Retrospective cohort study | Abdominal surgery | Undergoing colorectal surgery managed with ERAS and non-ERAS protocols | Emergent operation, without preoperative creatinine, on dialysis, pregnant | 59 (13.6) | 160 (51.8) | KDIGO | 309 | 44 | 0.14 |
| Youn Kyung Kee | 2019 | Retrospective cohort study | Colorectal surgery with intestinal resection | Underwent colorectal surgery with intestinal resection (for any indication) | Age < 18 or ≥ 80 years, follow-up < 3-month，post-renal AKI, missing data, preoperative chronic kidney disease or end-stage renal disease | 57.9 (13.5) | 415 (60.8) | RIFLE | 683 | 177 | 0.26 |
| Müzeyyen Iyigun | 2019 | Prospective cohort study | General surgery | ASA grade 1-2, age > 40years, noncardiac surgery | cardiovascular, urological and transplantation surgeries, local anesthesia, monitored anesthesia care, day case surgical procedures, previous renal surgery, pre-existing renal disease requiring renal replacement therapy, preoperative serum creatinine level higher than 1.4 mg/dl | Not stated by subgroup | Not stated by subgroup | KDIGO | 327 | 16 | 0.05 |
|  |  |  | Gynecologic surgery |  |  | Not stated by subgroup | - |  | 52 | 8 | 0.15 |
| Fabian Grass | 2019 | Retrospective cohort study | Colorectal surgery | Elective colorectal surgical, with ERAS | Nil | 55 (17) | 2125 (51.9) | RIFLE | 4096 | 104 | 0.03 |
| Konlawij Trongtrakul | 2019 | Prospective cohort study | Gastrointestinal surgery | Age≥18years, admitted to surgical ICU | ESKD, on maintenance hemodialysis prior to admission | Not stated by subgroup | Not stated by subgroup | KDIGO | 117 | 47 | 0.40 |
| Ivana Dedinská | 2019 | Retrospective cohort study | Liver resection | Liver resection | Kidney disease, diabetes mellitus, poor glomerular filtration | 58.7 (11.7) | 101 (51.8) | KDIGO | 195 | 26 | 0.13 |
| Anthony Bonavia | 2019 | Retrospective cohort study | Colorectal surgery | Age >18 years, having a scheduled visit to the anesthesia preoperative clinic before surgery, surgical time > 4hours | Undergoing multiple operative procedures within the given study period | NA | 543 (53.2) | KDIGO | 1020 | 567 | 0.56 |
| L. M. Vernooij | 2018 | Retrospective cohort study | General surgery | Age ≥50 years, intermediate or high risk non-cardiac surgery, general or spinal anaesthesia, postoperative hospital stays of ≥24 h | Reoperations within 30 days or within the same hospital admission, without intraoperative BP measurements, anaesthesia duration was <20 min | Not stated by subgroup | Not stated by subgroup | AKIN | 2176 | 214 | 0.10 |
|  |  |  | Gynecologic surgery |  |  | Not stated by subgroup | - |  | 621 | 17 | 0.03 |
| S. T. Collaborative | 2018 | Prospective cohort study | Major gastrointestinal surgery | Age ≥18 years, undergoing elective or emergency gastrointestinal resection, liver resection, or reversal of ileostomy or colostomy，using any operative approach | Other surgeries, developed AKI before undergoing surgery, with kidney transplant, undergone renal replacement therapy in the 90 days preceding surgery, it was not possible to establish whether they developed AKI in the first 7 days after operation | Not stated by aggregate | 2909 (55.3) | KDIGO | 4544 | 646 | 0.14 |
| Leili Pourafkari | 2018 | Retrospective cohort study | General surgery | Non-cardiovascular surgery | Cardiac and vascular surgeries, CKD stage V, lacked postoperative creatinine measurement | Not stated by subgroup | Not stated by subgroup | AKIN | 2928 | 515 | 0.18 |
|  |  |  | Gynecologic surgery |  |  | Not stated by subgroup | - |  | 97 | 1 | 0.01 |
| Taryn E Hassinger | 2018 | Retrospective cohort study | Colorectal surgery | Placement of temporary prophylactic ureteral stents prior to surgery, removed at the conclusion of the procedure prior to the patient leaving the operating room | Preoperative renal failure requiring hemodialysis, postoperative ureteral stent placement | 59 (16.7) | 1394 (47.9) | KDIGO | 2910 | 335 | 0.12 |
| S.Y. Ham | 2018 | Retrospective cohort study | Living-donor hepatectomy | Living-donor hepatectomy | Perioperative discontinuation of PCA | 34 (10.6) | 187 (59.1) | AKIN | 316 | 24 | 0.08 |
| Jonathan Garnier | 2018 | Retrospective cohort study | Major liver resection | Did not require the Pringle maneuver during liver transection | Nil | 66 (30-88) | 55 (50) | KDIGO | 111 | 24 | 0.22 |
| Juan P Cata | 2018 | Retrospective cohort study | CRS and HIPEC | Age≥18years | Incomplete perioperative data, preoperative RRT | 51 (12.7) | 213 (44.8) | AKIN | 475 | 101 | 0.21 |
| Joseph H. Marcotte | 2018 | Observational study | Colorectal surgery | Colorectal surgery | Emergency cases，admissions through the Emergency department | 61.8 (13.9) | 130 (49.2) | KDIGO | 264 | 18 | 0.07 |
| Yu Zhang | 2018 | Observational study | Abdominal surgery | Undergoing surgery, surgery performed in any operating room (except for outpatient surgery), with an operation time greater than 1 h | Aged < 18 years，RRT before surgery，with an ICU stay of < 24 h，renal transplantation < 3 months, insufficient clinical data | Not stated by subgroup | Not stated by subgroup | KDIGO | 619 | 281 | 0.45 |
| Alexandra Briggs | 2018 | Observational study | Emergency general surgery | Undergoing appendectomy, cholecystectomy, lysis of peritoneal adhesions, laparotomy, partial colectomy, operative management of peptic ulcer disease and small bowel resection within 48 h of ICU admission | ESRD prior to hospital admission, CKD stage 5 | 57.1 (18.2) | 35056 (59) | RIFLE | 59604 | 3554 | 0.06 |
| Vicente-Hernández B | 2017 | Retrospective cohort study | Apendicectomy | Age≥18years, underwent open or laparoscopic appendectom | Did not comply with fluid control at 4, 6 and 12 hours after surgery | 45.4 (46.2) | 34 (41.5) | KDIGO | 82 | 21 | 0.26 |
| Xing Zhou | 2017 | Observational study | Hepatectomy | Hepatitis B or C virus-related hepatocellular carcinoma, did not receive treatment for HCC before the diagnosis | Ruptured hepatocellular carcinoma，Barcelona Clinic Liver Cancer (BCLC) B-, C-, or D-staged HCC, other treatments, transarterial chemoembolization, percutaneous ethanol injection or transplantation (as opposed to surgery) | 53 (15-83) | 224 (88.5) | KDIGO | 253 | 22 | 0.09 |
| Xiujuan Wu | 2017 | Randomized Controlled Trial | Gastrointestinal surgery | Chronic hypertension, age 65–80 years, ASA grade 1-3, predicted surgery time >60 min, no surgery for preexisting renal disease, current left ventricular ejection fraction >50%, no sign of cardiac dysfunction | Used NSAIDs during the past month; heart failure within 2 months; myocardial infarction during the past month (conﬁrmed by blood-speciﬁc enzymes); current severe pulmonary function insufﬁciency; current intermediate to severe pulmonary hypertension; CKD or renal dysfunction | 73.3 (6.1) | 424 (65.6) | KDIGO | 646 | 71 | 0.11 |
| Y. Toyonaga | 2017 | Retrospective cohort study | Abdominal surgery | Underwent emergency abdominal or cerebral surgery | Pre-operative hemodialysis, age < 18 years, unknown clinical history, lack of blood test data, SIRS score and/or SAS not available, weight unknown | 56 (20) | 265 (60) | AKIN | 439 | 82 | 0.19 |
| Vafi Salmasi | 2017 | Retrospective cohort study | Colorectal resection | Preoperative and postoperative serum creatinine measurement available, blood pressure recorded within 6 months before surgery. | Urinary obstruction, renal transplant, nephrectomy procdures, history dialysis, missing creatinine, missing calculated eGFR, baseline eGFR<60ml/kg m^2^, surgical time < 60 minutes, no intraoperative BP reading, BP reading <6 per hour, the gap between two consecutive BP reading >10 minutes, no baseline BP within 6 months | Not stated by subgroup | Not stated by subgroup | AKIN | 4879 | 414 | 0.08 |
|  |  |  | Hysterectomy |  |  | Not stated by subgroup | - |  | 4251 | 196 | 0.05 |
| T. Mizota | 2017 | Retrospective cohort study | Major abdominal surgery | Age ≥18 years, general anaesthesia | Cardiac or urological procedures, ESRD, receipt of haemodialysis, received diuretics during surgery | 66 (56-73) | 2182 (61.3) | KDIGO | 3560 | 226 | 0.06 |
| Tyler J. Loftus | 2017 | Retrospective cohort study | Open abdominal surgery | Age ≥18 years, exploratory laparotomy, and temporary abdominal closure | Survival < 96 hours, cirrhosis, dialysis at initial laparotomy, pre-existing intestinal fistula, necrotizing pancreatitis | 56 (43-69) | 133 (53) | KDIGO | 251 | 70 | 0.28 |
| M. E. O'Connor | 2017 | Retrospective cohort study | Gastrointestinal surgery | Elective major non-cardiac surgery | Age <18 years, procedures performed under the specialty renal transplant surgery, any surgical procedure involving nephrectomy or partial nephrectomy, baseline eGFR<15ml/min 1.73m^2^ (stage 5 CKD) undergoing non-renal surgery, patient who did not have a serum creatinine measurement after surgery | Not stated by subgroup | Not stated by subgroup | KDIGO | 348 | 25 | 0.07 |
|  |  |  | Hepatobiliary |  |  | Not stated by subgroup | Not stated by subgroup |  | 276 | 20 | 0.07 |
|  |  |  | Gynaecology |  |  | Not stated by subgroup | - |  | 23 | 0 | 0.00 |
| Yosuke Toyonaga | 2017 | Retrospective cohort study | Abdominal surgery | Admission to ICU after elective abdominal surgery | Preoperative haemodialysis, preoperative eGFR < 60 mL/min/1.73m^2^, postoperative renal replacement therapy, perioperative use of NaHCO_3_ of ≥ 200 mL, serum creatinine concentration not available, age <18years, measurements using the Stewart approach not available, body weight not available | 64.5 (12.1) | 144 (69.9) | RIFLE | 206 | 42 | 0.20 |
| Fabian Grass | 2017 | Retrospective cohort study | Colorectal surgery | Colorectal surgery within an ERP | Nil | 63 (16.3) | 276 (53.8) | RIFLE | 513 | 42 | 0.08 |
| Or Goren | 2017 | Retrospective cohort study | Pancreatic surgery | Primary, open, non-emergent pancreatic surgery, age ≥ 18years, | An intervention in urethra, ureters or kidneys, laparoscopic surgeries, surgeries with a cross clamp proximal to the renal arteries, exposed to contrast dye in the preceding 7 days before the surgery or in the following 2 days after the surgery, already on RRT, lack of information on perioperative creatinine, intraoperative fluid administration, intraoperative urine output, length of surgery or weight | 63.8 (11.8) | 72 (47.1) | AKIN | 153 | 15 | 0.10 |
| A Abrahamsson | 2017 | Retrospective cohort study | Open pancreatic surgery | Underwent open pancreatic surgery from January 2014 to December 2014 were not treated according to the ERP concept, underwent pancreatic surgery from January 2015 to December 2015 were treated according to the ERP concept | Underwent surgery in January or February 2015 | 68.5 (10.5) | 62 (50.4) | KDIGO | 123 | 9 | 0.07 |
| Hung-Chieh Wu | 2016 | Retrospective cohort study | Abdominal surgery | Nil | Receiving radiocontrast study within the first 7 postoperative days, suffered from AKI >7 days after surgery, subjects on maintenance dialysis or dialysis prior to surgery, lengths of hospitalization < 48 h, lack of baseline demographic and biochemical data, age < 20years | Not stated by subgroup | Not stated by subgroup | KDIGO | 168 | 56 | 0.33 |
|  |  |  | Gynecologic surgery |  |  | Not stated by subgroup | - |  | 38 | 9 | 0.24 |
| Ana B Serrano | 2016 | Randomized Controlled Trial | Major elective open abdominal surgery | Adult, ASA grade 2-4 | pregnancy, laparoscopic or minor or urgent surgery, CKD (baseline serum creatinine >3.0 mg/dl), having any disease could result in volume overload, preoperative intravenous hydration had not been deﬁned | 63.3 (15.3) | 179 (54.6) | AKIN | 328 | 28 | 0.09 |
| Morgan E. Grams | 2016 | Retrospective cohort study | General (gastrointestinal/ abdominal) surgery | First instance of major surgery | Preoperative eGFR < 60 mL/min/1.73 m^2^，surgery > 30 days after admission，kidney surgeries, transplantation surgeries, gynecologic surgeries，developed ESRD between the original cohort inclusion date and major surgery date | 63 (12) | 41998  (94.2) | KDIGO | 44597 | 5887 | 0.13 |
| Stefano Romagnoli | 2016 | Prospective cohort study | Major abdominal surgery | Less than 6 metabolic equivalent, Revised Cardiac Risk Index for Pre-Operative Risk (RCRI) ≥3, developed AKI within 48 hours after surgery | Nil | 70.8 (11.1) | 151 (58.5) | KDIGO | 258 | 31 | 0.12 |
| Samuel Quan | 2016 | Retrospective cohort study | Abdominal surgery | Major noncardiac surgery, age ≥18years, SCr measured within 30 days prior to surgery, 7 days after surgery, at least two UO measurements within 24 h during the first 7 days after surgery | ESRD prior to surgery, dialysis  Or a catheter inserted for dialysis on or prior to the day of surgery | Not stated by subgroup | Not stated by subgroup | KDIGO | 3036 | 200 | 0.07 |
| Thorir E Long | 2016 | Observational study | Abdominal surgery | Abdominal surgery | Age < 18years, requiring RRT for ESRD before surgery | 62 (17) | 1779 (45.60) | KDIGO | 3902 | 264 | 0.07 |
| Sung Yoon Lim | 2016 | Retrospective cohort study | Rectal surgery | Age≥18 years, elective rectal resection, rectal cancer | Baseline eGFR < 60 mL/min/1.73 m^2^, ESRD on maintenance RRT, AKI developed in the week before surgery | 62 (12) | 185 (64.2) | AKIN | 288 | 11 | 0.04 |
| Sebastian Schmid | 2016 | Randomized Controlled Trial | Major abdominal surgery | Age≥18years, ASA grade 1-3, undergoing major non-cardiac surgery planned to last at least 3 hours with an expected subsequent intensive care treatment of more than 3 days | Need for dialysis and contraindication for an arterial line in the femoral artery | 66 (11.5) | 138 (76.7) | KDIGO | 180 | 99 | 0.55 |
| Chetana Lim | 2016 | Retrospective cohort study | Hepatectomy | Hepatocellular carcinoma, Child class A liver function, a sufﬁcient volume of the future remnant liver | Without available baseline scr levels | 60.5 (13.4) | 379 (83) | KDIGO | 457 | 67 | 0.15 |
| S.-K. Kim | 2016 | Retrospective cohort study | Hepatectomy | Right hepatectomy for living donor liver transplantation | Nil | 27.5 (8.1) | 532 (36.4) | AKIN | 1641 | 68 | 0.04 |
| Ji-Yeon Bang | 2016 | Retrospective cohort study | Colorectal surgery | Colorectal surgery, adult | Repeated operations, combined operations on parts other than large or small bowel, with anesthetics other than sevoflurane or propofol, pre- or postoperative sCr values were not available | 60 (51-68) | 2667 (67.7) | AKIN | 4320 | 414 | 0.10 |
| A. Arjona-Sanchez | 2016 | Retrospective cohort study | CRS and HIPEC | CRS and HIPEC | Nil | 56.7 (10.5) | 9 (6.4) | RIFLE | 141 | 61 | 0.43 |
| Hung-Chieh Wu | 2015 | Retrospective cohort study | Abdominal surgery | Age≥18 years, major surgery | Cardiac surgery, admitted due to medical problem, RRT during admission, lengths of hospitalization < 48 h, chronic dialysis, RRT before surgery, without data on urine output or lack of baseline renal function, suffered from AKI 7 days after surgery, receiving radiocontrast study within the first 7 postoperative days | Not stated by subgroup | Not stated by subgroup | KDIGO | 125 | 39 | 0.31 |
|  |  |  | Gynecologic surgery |  |  | Not stated by subgroup | - |  | 18 | 4 | 0.22 |
| David J.R. Morgan | 2015 | Observational study | Bariatric surgery | Age≥18 years, admitted to an ICU | Nil | 47.9 (11.3) | 294 (60) | AKIN | 590 | 103 | 0.17 |
| Camilo Correa-Gallego | 2015 | Retrospective cohort study | Liver resection | Liver resection under low central venous pressure | Nil | 61 (51-70) | 1081 (51) | RIFLE | 2116 | 336 | 0.16 |
| AJ Vaught | 2015 | Retrospective cohort study | Major gynaecologic surgery | Age ≥ 18years, admitted to the hospital > 24 hours, | CKD prior to admission | 54 (15) | - | RIFLE | 2341 | 295 | 0.13 |
| Louise Y. Sun | 2015 | Retrospective cohort study | General Abdominal surgery | Elective noncardiac surgery, length of stay >1-day, intraoperative invasive BP monitoring | Preoperative MAP less than 65 mmhg, dialysis dependence, urologic surgery, surgical duration < 30 min | Not stated by subgroup | Not stated by subgroup | AKIN | 1348 | 130 | 0.10 |
|  |  |  | Gynaecologic surgery |  |  | Not stated by subgroup | - |  | 865 | 27 | 0.03 |
| P. Kambakamba | 2015 | Retrospective cohort study | Major liver resection | Liver resection | Combined procedures, liver cyst deroofing, all ablative procedures, factors known to cause postoperative AKI, intraoperative discontinuation of epidural analgesia | 57.4 (14) | 448 (54) | AKIN | 829 | 68 | 0.08 |
| Ren Hounu | 2015 | Observational study | Esophageal cancer surgery | Esophageal cancer surgery, postoperative pathologically confirmed diagnosis | Nil | 64(15.1) | Nil | KDIGO | 262 | 23 | 0.08 |
| Miho Tagawa | 2015 | Retrospective cohort study | Abdominal surgery | Age≥18years, non-cardiac surgery, general anesthesia | Urological surgery, missing creatinine values, preoperative dialysis | Not stated by subgroup | Not stated by subgroup | AKIN | 1219 | 79 | 0.06 |
| Arisa Tomozawa | 2015 | Retrospective cohort study | Major liver resection | Liver resection surgery | Emergency surgery，preoperative dialysis, died within 3 days postoperatively, lack of indocyanine green data, anesthetic agents other than sevoflurane | 67 (10.4) | 463 (72) | AKIN | 642 | 78 | 0.12 |
| David R. Mcllroy | 2015 | Retrospective cohort study | General abdominal surgery | Age > 40 years, nonemergent surgery | Preoperative RRT, partial or complete nephrectomy, or with no postoperative serum creatinine measurement within 7 days of surgery | Not stated by subgroup | Not stated by subgroup | KDIGO | 115 | 6 | 0.05 |
| Catarina Teixeira | 2014 | Retrospective cohort study | Major abdominal surgery | Age ≥ 18years, scheduled or urgent major nonvascular abdominal surgery, admitted to the Post-Anesthesia Care Unit, | CKD on RRT, RRT in week before surgery, hospital stay < 48 hours, < 2 determinations of serum creatinine, repeat surgery | 62 (26) | 227 (50.4) | KDIGO | 450 | 101 | 0.22 |
| Murat Biteker | 2014 | Prospective cohort study | Gastrointestinal surgery | Noncardiac and nonvascular surgery | Pre-existing renal dysfunction, local anesthesia, monitored anesthesia care, day case surgical procedures, emergent surgical, moribund patient (ASA5 or not expected to live >24 hours) | Not stated by subgroup | Not stated by subgroup | RIFLE | 510 | 36 | 0.07 |
|  |  |  | Gynecologic surgery |  |  | Not stated by subgroup | - |  | 59 | 6 | 0.10 |
| Eun-Ho Lee | 2014 | Retrospective cohort study | Esophageal cancer surgery | Esophageal cancer surgery | Missing preoperative serum creatinine measurements, pre-existing renal failure requiring preoperative dialysis, repeat esophageal surgery, concomitant renal or hepatic surgery | 62 (8.2) | 560 (94.1) | AKIN | 595 | 210 | 0.35 |
| Shu Li | 2014 | Retrospective cohort study | Major abdominal surgery | Age >18 years, APACHE II score >8, endotracheal intubation and mechanical ventilation for abdominal surgery under general anesthesia, cumulative duration of postoperative mechanical ventilation > 24 h | Died or discontinued mechanical ventilation due to other reasons before weaning, pregnant or nursing women, underwent sequential noninvasive mechanical ventilation after weaning as planned, tracheotomy, etiologies of elevated TnT were both ischemic and non-ischemic or could not be confirmed | 63.8 (16.5) | 212 (55.6) | KDIGO | 381 | 120 | 0.31 |
| Samira Bell | 2014 | Interrupted time series analysis | Gastrointestinal surgery | Age > 18 years, underwent surgical procedures within the specified follow-up period | Nil | 61.9 (16.1) | 1416 (43.3) | KDIGO | 3271 | 322 | 0.10 |
|  |  |  | Gynecologic surgery |  |  | 54.1 (13.7) | - |  | 403 | 17 | 0.04 |
| Eunjung Cho | 2014 | Prospective cohort study | Hepatobiliary surgery | Elective hepatobiliary surgery | Age <18 years, eGFR < 60ml/min/1.73m^2^, maintenance RRT, preoperative AKI,  emergency surgery | 57 (12.4) | 84 (63.2) | AKIN | 111 | 2 | 0.02 |
| Chang Seong Kim | 2013 | Retrospective cohort study | Gastric cancer surgery | Gastric cancer surgery | Insufficient data, emergency operation, CKD (preoperative eGFR< 60 mL/ min/1.73 m^2^), ESRD, history of hemodialysis, peritoneal dialysis, kidney transplantation, died within 24 hours of gastric surgery | 63.2 (12.1) | 3171 (67.2) | KDIGO | 4718 | 679 | 0.14 |
| Toby N. Weingarten | 2013 | Case–control study | Bariatric surgery | Surgery is only for obesity | Without baseline and postoperative sCr | 49.2 (12) | 65 (30.5) | AKIN | 1161 | 71 | 0.06 |
| Chia-Ter Chao | 2013 | Prospective cohort study | Abdominal surgery | Age ≥ 65 years, admitted into ICU | Receiving chronic dialysis (>3 months of any form of dialysis), received dialysis before ICU admission, hospital stay < 2 days, only 1 serum creatinine value during admission, repeated ICU admission | Not stated by subgroup | Not stated by subgroup | AKIN | 1972 | 400 | 0.20 |
| Ian M. Paquette | 2013 | Observational study | Colorectal operation with ileostomy creation | Primary admitting diagnosis of renal failure or dehydration, no other conditions leading to renal failure and dehydration | Intravenous fluids as an outpatient | 47 (16.5) | 109 (54.2) | AKIN | 201 | 18 | 0.09 |
| Ksenija Slankamenac | 2013 | Observational study | Liver surgery | Liver surgery | Liver trauma, incomplete intraoperative data | 58 (13.5) | 302 (55) | RIFLE | 549 | 82 | 0.15 |
| Laszlo Medve | 2012 | Observational study | Abdominal surgery | Respect to surgical interventions | Medical patients, CKD patients on dialysis, theoretically the renal transplant patients, polytraumatic patients, several body cavities affecting surgical intervention | Not stated by subgroup | Not stated by subgroup | AKIN | 145 | 39 | 0.27 |
| Steven M. Brunelli | 2012 | Retrospective cohort study | Major open abdominal surgery | major open abdominal, cardiac, thoracic, or vascular procedure | ESRD, dialysis, renal transplantation, AKIN stage 1 prior or at the time of surgery, surgery occurred > 3 days after hospital admission, no baseline creatinine measurement | Not stated by subgroup | Not stated by subgroup | AKIN | 1912 | 512 | 0.27 |
| Vin-Cent Wu | 2011 | Observational study | General surgery | Major surgery | Kidney transplant, repeated admission, ICU stay < 2 days, died during hospital admission | Not stated by subgroup | Not stated by subgroup | RIFLE | 432 | 236 | 0.55 |
| Ashwin Kurian | 2011 | Retrospective cohort study | Open colorectal resection | Age ≥ 80 years | Laparoscopic colon resections | 84.5 (80-94) | 72 (44.4) | AKIN | 162 | 21 | 0.13 |
| Marlin Wayne Causey | 2011 | Retrospective cohort study | Colectomy | Age > 18years, emergent and elective colectomies | Rectal operations | 61.9 (0.94) | 205 (60.5) | RIFLE | 339 | 40 | 0.12 |
| E. Biagioni | 2011 | Observational study | Abdominal surgery | OLT or nonelective abdominal surgery, ICU stay > 2 days, no prior end-stage renal failure | Nil | 73 (14) | NA | RIFLE | 60 | 29 | 0.48 |
| Enoch Arhinful | 2011 | Retrospective cohort study | Abdominal surgery | Octogenarian | Intraoperative deaths | 84 (80-98) | 33 (49.3) | RIFLE | 67 | 24 | 0.36 |
| Steven G. Coca | 2010 | Retrospective cohort study | General surgery | Noncardiac surgery, US Veterans Affairs Medical Centers diabetic veterans, with a pre- and postoperative serum creatinine values | Preoperative AKI, ESRD, metastatic cancer, died during hospital stay | Not stated by subgroup | Not stated by subgroup | AKIN | 11460 | 2166 | 0.19 |
| Thomas Armstrong | 2009 | Retrospective cohort study | Liver resection | Liver resection | Long-term RRT, Non-low central venous  pressure anesthesia | 56 (48) | 892 (58) | AKIN | 1535 | 78 | 0.05 |
| Azra Bihorac | 2009 | Retrospective cohort study | General/gastrointestinl surgery | General/gastrointestinal, vascular surgery, cardiothoracic surgery or neurosurgical operative procedure, survived to be discharged from the hospital | Trauma, burn, orthopedic, ear-nose-throat, urologic, and kidney transplantation surgery, CKD of any stage | Not stated by subgroup | Not stated by subgroup | RIFLE | 2337 | 918 | 0.39 |
| Charuhas V. Thakar | 2007 | Retrospective cohort study | Bariatric surgery | Bariatric surgery | Missing postoperative data | 43 (10) | 81 (16.5) | RIFLE | 491 | 42 | 0.09 |
| Christian T.J. Magyar | 2025 | Retrospective cohort study | Laparoscopic liver resection | Age > 18years, elective L-LR | Missing postoperative data | 61.0 (51.1-69.0) | 206 (57) | KDIGO | 360 | 16 | 4.4 |
| Brandon P. Maddy | 2025 | Retrospective cohort study | Open gynecologic surgery | Age >18 years, open surgery for gynecologic malignancy | No preop creatinine within 6 months, no postop creatinine, benign/borderline pathology, no research authorization, CKD stage ≥4 | 62.8 (12.3) | NA | KDIGO | 621 | 55 | 8.9 |
| Lingzi Yin | 2024 | Prospective cohort study | Major abdominal surgery | Age ≥65 years, elective major abdominal surgery (expected duration ≥2h) | MI ≥30 kg/m², preop GFR <30 ml/min/1.73m², history of nephrectomy, contrast agent within 24h, skin abnormalities, impaired hearing/vision | 71.1 (5.0) | 169 (71.6) | KDIGO | 236 | 44 | 18.6 |
| Rao Sun | 2024 | Retrospective cohort study | Major abdominal surgery | Age ≥65 years, elective major abdominal surgery under general anesthesia | ASA V, concurrent cardiac/renal surgery, ESRD, insufficient lab data for nutritional/AKI evaluation | 69 (67-74) | 1951 (70.3) | KDIGO | 2775 | 144 | 5.2 |
| Xiran Peng | 2024 | Prospective cohort study | Abdominal surgery | Age ≥65 years, noncardiac surgery | CKD (eGFR <60 ml/min/1.73m²), urologic procedures, lost to follow-up | Not stated by subgroup | Not stated by subgroup | KDIGO | 2728 | 193 | 7.1 |
| Swapnil Y. Parab | 2024 | Prospective observational study | Elective oesophagectomy | Adult patients， elective oesophagectomy | Preop chronic renal insufficiency (creatinine >1.5 mg/dl), history of AKI, history of RRT | 54.4 (11.8) | 98 (53.6) | KDIGO | 183 | 27 | 14.7 |
| Emi Ota | 2024 | Retrospective observational study | Colorectal tumor surgery with diverting ileostomy | Primary colorectal tumors (cancer, neuroendocrine tumor, leiomyosarcoma), underwent primary tumor resection and diverting ileostomy, underwent stoma closure | Diseases other than primary tumors, colostomy, incomplete data, R2 resection, no stoma closure | 67 (27-89) | 334 (75.6) | KDIGO | 442 | 99 | 22.4 |
| Isaac D. Schmied | 2024 | Retrospective cohort study | Colorectal surgery | Age ≥18 years, ASA 1-4, colorectal cancer and benign diseases | Preop sepsis, upper abdominal colorectal procedures not typically considered for stents | 61.6 (13.5) | 197 (48) | KDIGO | 410 | 83 | 20.2 |
| David R. McIlroy | 2024 | Secondary analysis of RCT | Major abdominal surgery | Adult patients undergoing elective major abdominal/pelvic surgery, expected duration ≥2h, anticipated hospital stay ≥3 days | Urgent/time-critical surgery, hepatic resection, end-stage kidney failure requiring dialysis | 70 (58-76) | 1180 (48.3) | KDIGO | 2444 | 318 | 13 |
| John D. Mannion | 2024 | Retrospective cohort study | Colorectal surgery | Consecutive colorectal surgery patients | None reported | 64.3 (14.6) | 597 (48.8) | KDIGO | 1224 | 301 | 24.6 |
| Kerri Lydon | 2024 | Retrospective cohort study | Major pancreatic surgery | Adults >18 years, pancreatectomy for malignancy | Planned pancreatic resection excluded due to metastatic/unresectable disease | 68(58-74) | 97(48.5) | KDIGO | 200 | 20 | 10 |
| Yunwei Lu | 2024 | Retrospective cohort study | CRS-HIPEC | CRS-HIPEC for various primary malignancies with peritoneal dissemination | Incomplete perioperative data, severe liver/kidney dysfunction, identified CKD, only diagnostic laparoscopy, dialysis patients | 60 (12) | 63 (39.9) | KDIGO | 158 | 34 | 21.5 |
| Yunwei Lu | 2024 | Retrospective observational study | Major abdominal surgery | Adults ≥18 years, elective major abdominal surgery (duration ≥120 min) | ASA V-VI, no IV dexmedetomidine, missing Scr data, incomplete discharge data, missing dexmedetomidine dosage, pre-existing CKD or eGFR <60 | 60 (12) | 63 (39.9) | KDIGO | 158 | 34 | 21.5 |
| Siqian Li | 2024 | Retrospective observational study | Pancreatic surgery | Adults 18-85 years, pancreatic surgery, general anesthesia | Preoperative dialysis dependence, urological procedures during operation, emergency surgery, previous pancreatic surgery history, incomplete data | 61 (18-85) | 835 (55.5) | KDIGO | 1504 | 79 | 5.3 |
| Martin Krause | 2024 | Retrospective observational study | CRS-HIPEC | Adults, undergoing HIPEC | Scheduled but did not undergo HIPEC, on dialysis prior to surgery | 55 (17) | 179 (43.4) | KDIGO | 412 | 36 | 8.7 |
| Shun-Cai Gao | 2024 | Retrospective cohort study | CRS-HIPEC | Adults ≥18 years undergoing open or mini-invasive CRS-HIPEC | Previous CKD (eGFR <60), on dialysis, concomitant nephrectomy, repeated CRS-HIPEC, missing baseline creatinine, missing postoperative creatinine data, missing intraoperative temperature data | 57 (11) | 177 (36.9) | KDIGO | 480 | 51 | 10.6 |
| Yichun Cheng | 2024 | retrospective cohort study | Abdominal surgery | Adults ≥18 years undergoing surgical procedures | Outpatient surgery (≤1 day stay), nephrectomy surgery, end-stage renal disease, missing baseline or postoperative creatinine | 56(15) | 89835(53.1) | KDIGO | 169330 | 7112 | 4.2 |
|  |  |  | Gynaecologic |  |  | 41(13) |  | KDIGO | 71482 | 1573 | 2.2 |
| Diego Beltrán | 2024 | Retrospective cohort study | CRS-HIPEC | Admitted to ICU after CRS and HIPEC | Did not provide consent to participate | 59.5 (10.5) | 61 (49.6) | KDIGO | 123 | 6 | 4.9 |
| Yao Yu | 2023 | Prospective Cohort Study | Open hepatectomy | Older patients ≥60 years undergoing elective open hepatectomy | Emergency surgery, liver transplantation, BMI >30, renal depth >4cm, eGFR <60, unable to consent | 66 (60–70) | 109 (69) | KDIGO | 157 | 23 | 14.6 |
| Yang-Tian Yan | 2023 | Retrospective cohort study | Digestive system | Adults 18-85 years undergoing major noncardiac surgery | Preoperative creatinine ≥132.5 μmol/L, cardiac surgery, genitourinary surgery, history of renal disease, missing data | Not stated by subgroup | Not stated by subgroup | KDIGO | 4959 | 47 | 9.7 |
| Oskar Swartling | 2023 | Retrospective cohort study | Pancreatoduodenectomy | Adults ≥18 years, pancreatoduodenectomy | Previous left-sided pancreatic resection, no postoperative creatinine or urine volume measurements | 68 (61-74) | 517 (53.3) | KDIGO | 970 | 137 | 14.1 |
| E Rossouw | 2023 | Retrospective cohort study | Abdominal surgery | Adults ≥18 years, major non-cardiac surgery | End-stage kidney disease (eGFR <15), previous RRT, cardiac surgery, caesarean section | Not stated by subgroup | Not stated by subgroup | KDIGO | 442 | 82 | 18.6 |
| Zbigniew Putowski | 2023 | Retrospective cohort study | Pancreaticoduodenectomy | Adults, pancreaticoduodenectomy | End-stage renal disease (CKD G5), intraoperative death, re-operation before AKI onset, incomplete data | 64 (57-69) | 154 (50.8) | KDIGO | 303 | 58 | 19.1 |
| Lukas F Liesenfeld | 2023 | Retrospective observational study | CRS-HIPEC | Patients with peritoneal malignancies receiving CDDP-containing HIPEC | Not specified | 62 (10) | 23 (41.2) | KDIGO | 56 | 37 | 66.1 |
| Liting Kuang | 2023 | Retrospective case-control study | Hepatectomy | Patients undergoing hepatectomy | Preoperative acute renal failure, requiring RRT for CKD, age <18, missing baseline data, nephrotoxic medication | 56 (12) | 1662 (85.3) | KDIGO | 1949 | 148 | 7.6 |
| Hye Jin Kim | 2023 | Retrospective observational study | Major Abdominal Surgery | Patients undergoing major abdominal surgery with RBC transfusion | Incomplete data | 62(51-72) | 962(40) | KDIGO | 2408 | 321 | 13.3 |
| Pyoyoon Kang | 2023 | Retrospective cohort study | General surgery | Elective or emergent operations under general anesthesia at general surgery department | Multiple surgeries during single admission, inadequate anesthetic records, incomplete data | 61(14) | 933(60) | KDIGO | 1586 | 123 | 7.8 |
| Yuchen Ji | 2023 | Retrospective cohort study | Pancreaticoduodenectomy | Patients undergoing classic Whipple procedure with standard lymphadenectomy for PDAC | Age <18 years, pre-existing pancreatic surgery, evidence of metastasis, locally advanced or borderline unresectable tumors, additional organ resection | 63(9) | 829（63.2） | KDIGO | 1312 | 141 | 10.7 |
| Tasuku Fujii | 2023 | Retrospective cohort study | Abdominal surgery | Under general anesthesia using FloTrac/EV1000 system | Emergency surgery, ASA-PS ≥3, hemodialysis for CKD | 69 (55–75) | 269 (60%) | KDIGO | 448 | 59 | 13 |
| Diana J. Valencia Morales | 2023 | Retrospective cohort study | Laparoscopic pancreas surgery | Adult patients undergoing laparoscopic pancreas operations | Diagnostic laparoscopy, missing creatinine values, severe CKD requiring hemodialysis, no informed consent | 66 (58-74) | 201（55） | AKIN | 365 | 42 | 11.4 |
| Jianghua Shen | 2022 | Retrospective cohort study | Major abdominal surgery | Patients aged ≥75 years undergoing scheduled major non-vascular abdominal surgery | ESKD patients receiving RRT or kidney transplant recipients, missing baseline or follow-up creatinine values | 81 (4.4) | 297 (51.8) | KDIGO | 573 | 33 | 5.8 |
| Zbigniew Putowski | 2022 | Prospective cohort study | Abdominal surgery | Patients undergoing abdominal surgery | Organ procurement, reoperations, local anesthesia/monitored anesthesia supervision, immediate procedures, cardiac valve defects | 61 (45-68) | 239 (46) | KDIGO | 494 | 32 | 6.5 |
| Theis B Mikkelsen | 2022 | Retrospective cohort study | Major emergency abdominal surgery | Age ≥18, major emergency abdominal surgery within 72h of admission | Minor abdominal procedures (appendectomy, cholecystectomy), trauma surgery, missing creatinine values |  | 319（45.4） | KDIGO | 703 | 122 | 17.4 |
| Conor F. Murphy | 2022 | Retrospective observational study | Esophageal cancer surgery | Curative-intent surgery for esophageal cancer | Endoscopic therapy only, emergency surgery | 64.2 (9.2) | 855 (75.3) | AKIN | 1135 | 208 | 18.3 |
| Anthony Loria | 2022 | Retrospective cohort stud | Ileostomy formation | Adults with baseline serum creatinine discharged alive after ileostomy formation | Missing baseline creatinine, death during admission | 61 (52-68) | 138 (52.7) | KDIGO | 262 | 51 | 19.4 |
| Eriya Imai | 2022 | Retrospective cohort study | Laparoscopic liver resection | Aged ≥18, elective LLR with ASA 1-3, hepatectomy for primary/secondary malignancies or benign tumors | Cardiac arrhythmias, end-stage renal disease on hemodialysis, concomitant pancreaticoduodenectomy, missing perioperative data | 72 (65–77) | 94 (74.0) | KDIGO | 127 | 34 | 26.8 |
| Paul Andrew Drakeford | 2022 | Retrospective cohort study | Colorectal surgery | Adult patients requiring CRS within ERAS program | Age <18, stage 5 CKD, concomitant liver resection | 67.8 (11.4) | 325 (58.6) | KDIGO | 555 | 74 | 13.3 |
| Catherine Chiu | 2022 | Retrospective observational study | Major abdominal surgery | Adults >18 undergoing non-emergent, non-obstetric, non-ambulatory major abdominal surgery with case duration >120 min requiring general anaesthesia | Liver resections, organ transplantations, patients requiring intraoperative inotropes/vasopressors, pre-induction vasopressors, pre-induction intubation, ASA 5-6, missing critical variables | 58 | 15414 (47.8) | KDIGO | 32250 | 3991 | 12.4 |
| STARSurg Collaborative | 2022 | Retrospective observational study | Major gastrointestinal surgery | Adults, undergoing major gastrointestinal surgery | Incomplete outcome data, incomplete predictor data | 75.2 (16.3) | 6393 (64.6) | KDIGO | 9889 | 1413 | 14.3 |
| Bernd Saugel | 2022 | Retrospective cohort study | General surgery | Adults having noncardiac surgery with general anaesthesia lasting >60 min | CKD stage 5, BMI >100, missing baseline creatinine, need for RRT, kidney transplant history, <10 MAP values, ASA 6, genitourinary surgery | Not stated by subgroup | Not stated by subgroup | KDIGO | 10514 | 816 | 7.8 |
|  |  |  | Gynaecologic |  |  |  |  | KDIGO | 1750 | 58 | 3.3 |
| Sina Krone | 2025 | Retrospective observational study | Abdominal surgery | Adults ≥18, major noncardiac surgery, general anaesthesia, receiving continuous vasopressor infusions, surgery >2h, hospital stay ≥1 night | Transplant surgery, obstetric procedures, trauma, pre-case vasopressor use, transfer within 24h, ECMO | Not stated by subgroup | Not stated by subgroup | KDIGO | 752 | 108 | 14.4 |
| Alexander Zarbock | 2023 | Retrospective observational study | Abdominal surgery | Adults ≥18 undergoing major surgery (≥2h) requiring ICU or HDU admission | Pre-existing AKI, AKI within 3 months, ESRD on dialysis, kidney transplant | Not stated by subgroup | Not stated by subgroup | KDIGO | 3170 | 571 | 18 |
| Tetsuya Shimada | 2023 | Retrospective observational study | Digestive system surgery | Adults who had noncardiac surgery with creatinine recorded preoperatively and postoperatively | CKD, urologic procedures, preoperative creatinine >1.3 mg/dL, anesthesia <60 min, invalid/unavailable postoperative BP data >9 consecutive hours | Not stated by subgroup | Not stated by subgroup | KDIGO | 26104 | 1306 | 5 |
| Judith Schiefer | 2023 | Retrospective cohort study | Abdominal surgery | Adults >18 admitted to anaesthesiological/surgical ICUs after major surgery | Chronic RRT, renal transplantation, missing preoperative bSCr within 14 days, missing UO values, no surgery prior ICU admission | Not stated by subgroup | Not stated by subgroup | KDIGO | 1297 | 385 | 29.7 |
| Li Dong | 2023 | Retrospective cohort study | Major abdominal surgery | Adults，major abdominal surgery under general anesthesia | Missing intraoperative EtCO2 data, second/subsequent surgery, urological surgery, RRT for ESRD， preoperative platelet <100×10³/μL, preoperative total bilirubin ≥2.0 mg/dL | 66 (56–74) | 2551 (61.2) | KDIGO | 4171 | 290 | 7 |
| Yuanqiang Dai | 2023 | Retrospective cohort study | Open pancreatic surgery | Adults, underwent pancreatectomy | Tumor metastasis, age <18, total pancreatectomy, lost to follow-up Not specified Not specified KDIGO (AKI as secondary outcome) | 60 (12) | 861 (58.1) | KDIGO | 1481 | 86 | 5.8 |
| Amjad Bani Hani | 2023 | Cross-sectional retrospective study | Digestive system suregery | Surgical and non-surgical Octogenarians (≥80 years) admitted to SICU | Presented to SICU with AKI, ESRD on dialysis, missing clinical data | Not stated by subgroup | Not stated by subgroup | AKIN | 51 | 13 | 25.5 |
| Jiao-Nan Yang | 2022 | Prospective cohort study | Abdominal surgery | High-risk adults undergoing non-cardiac surgery admitted to SICU | CKD stage 5/long-term dialysis, no preoperative urinalysis, emergency surgery, kidney-related surgery, AKI before SICU admission, no Foley catheter, | Not stated by subgroup | Not stated by subgroup | KDIGO | 391 | 36 | 9.2 |
| Jiaqi Wang | 2022 | Retrospective observational study | abdominal surgery | Adults undergoing major/intermediate noncardiac surgery with preoperative NT-proBNP and LVEF measurements | Minor surgery, cardiac/neurological/vascular/urological/ophthalmology/transplant/obstetric procedures, eGFR<15 mL/min/1.73m², preoperative AKI, RRT/kidney transplant history | Not stated by subgroup | Not stated by subgroup | KDIGO | 1330 | 116 | 8.7 |
|  |  |  | Gynaecological |  |  |  |  |  | 52 | 3 | 5.8 |
| Tetsuya Shimada | 2022 | Retrospective observational study | abdominal surgery | Adults with noncardiac surgery (ASA 1-5), surgery ≥1 hour, pre/postoperative creatinine recorded, preoperative clinic BP within 6 months | CKD (eGFR<60 mL/min/1.73m²), dialysis, urological procedures, anesthesia <60 min, invalid/unavailable data >10 consecutive min | Not stated by subgroup | Not stated by subgroup | KDIGO | 7007 | 499 | 7.1 |
|  |  |  | Gynaecological |  |  |  |  |  | 4271 | 88 | 2.1 |
| Hirotaka Okuda | 2022 | Prospective observational study | Emergency laparotomy | Adults (43-99 years) undergoing emergency laparotomy for acute abdomen due to digestive system diseases | Chronic renal disease requiring hemodialysis, acute abdomen from urological/gynecological disorders | 75 (59-82) | 33 (68.7) | KDIGO | 48 | 10 | 20.8 |
| Linhui Hu | 2022 | Prospective observational study | Abdominal surgery | Critically ill patients admitted to ICU immediately after noncardiovascular emergency surgery | Age <18 years, refusal of consent, preexisting ESRD, presence of AKI before emergency surgery, missing admission data | Not stated by subgroup | Not stated by subgroup | KDIGO | 161 | 79 | 49.1 |
| W. Brenton French | 2022 | Retrospective cohort study | Abdominal surgery | Adults (≥18 years) undergoing elective surgery with postoperative LOS ≥24 hours | Admitted prior to operation, postoperative stay <24h, cardiac surgery, urological surgery, nephrectomy, organ transplants, preoperative ESRD | Not stated by subgroup | Not stated by subgroup | KDIGO | 2042 | 215 | 10.5 |
| Wendy De Guglielmo | 2022 | Case-control study | Abdominal surgery | Patients aged ≥65 years undergoing scheduled non-cardiac, non-ambulatory surgery | Emergency surgery, cardiac surgery, endovascular surgery, nephrectomy, ambulatory surgery (LOS<24h), CKD on hemodialysis, pre-operative AKI, high percentage of missing data ≥65 years | Not stated by subgroup | Not stated by subgroup | KDIGO | 320 | 91 | 28.4 |
| J Ripollés-Melchor | 2022 | Retrospective observational study | Elective colorectal surgery | Adult patients undergoing elective colorectal surgery | No valid preoperative BP, no data on outcomes | 73 (61-80) | 499 (61.6) | KDIGO | 810 | 94 | 11.6 |
| Kazuyuki Mizunoya | 2021 | Retrospective cohort study | Elective hepatectomy | Adult patients undergoing elective hepatectomy | Concomitant colorectal cancer surgery, donor hepatectomy for liver transplantation, patients on dialysis | 67 (60-74) | 544(72.5) | KDIGO | 750 | 135 | 18 |
| Henrique Tadashi Katayama | 2021 | Prospective cohort study | abdominal surgery | Patients aged ≥18 years undergoing non-cardiac surgery requiring post-operative ICU care | Pre-operative AKI, chronic kidney disease, terminal cancer, palliative care, severe liver failure (Child C), pregnant women, hospital stay <12h, ICU readmission ≥18 years | Not stated by subgroup | Not stated by subgroup | KDIGO | 252 | 59 | 23.4 |
|  |  |  | Gynaecological |  |  |  |  |  | 19 | 1 | 5.3 |
| Bertha M. Cordova-Sánchez | 2021 | Retrospective cohort study | Abdominal surgery | Adult patients (>18 years) admitted to ICU within 24h after oncological surgery with at least one organ dysfunction | CKD undergoing RRT, pre-operative AKI | Not stated by subgroup | Not stated by subgroup | KDIGO | 294 | 196 | 66.7 |
| Karthik V Iyer | 2025 | Retrospective observational study | Major gastro-intestinal surgery | Adult patients (>18 years) undergoing major (>2 hours) open abdominal surgery | Chronic kidney disease history, surgery within 6 months of present admission >18 years | 46（16） | 239 (51.8) | KDIGO | 461 | 83 | 18 |
| Seokyung Shin | 2025 | Retrospective observational study | Major hepatectomy | Patients undergoing extended right hepatectomy, extended left hepatectomy, or central hepatectomy | Not specified | 56 (15) | 940 (68) | KDIGO | 1383 | 154 | 11.1 |
| Thomas G. Matkov | 2024 | Retrospective cohort study | Colorectal surgery | Patients undergoing open and robotic-assisted laparoscopic colorectal surgeries | Unilateral stenting, preoperative hemodialysis patients | 60 | 299 (46.2) | KDIGO | 633 | 25 | 3.9 |

Abbreviations: ACEi, angiotensin converting enzyme inhibitor; ARB angiotensin II receptor blocker; AKI, acute kidney injury; APACHE, Acute Physiology and Chronic Health Evaluation; ASA, American Society of Anesthesiologists; BMI, body mass index; BUN, Blood Urea Nitrogen; CKD, chronic kidney disease; COPD, chronic obstructive pulmonary disease; CTP, Child-Turcotte-Pugh; eGFR, estimated glomerular filtration rate; ICU, intensive care unit; MAP, mean arterial blood pressure; MBP, mean blood pressure; MELD, Model For End-Stage Liver Disease; PNI, prognostic nutritional index, PNI=[10 × serum albumin (g/dL)] + [0.005 × total lymphocyte count (per mm 3)]; POD, post-operative day; RBC, red blood cell.

Table S4. **Risk of bias assessment using ROBINS-E tool for observational studies (For citations see main manuscript)**

| Study ID | D1 | D2 | D3 | D4 | D5 | D6 | D7 | Overall |
| --- | --- | --- | --- | --- | --- | --- | --- | --- |
| Bing-Cheng Zhao | Low | Moderate | Low | Low | Low | Low | Low | Moderate |
| Jianjun Xu | Moderate | Moderate | Low | Low | Low | Low | Moderate | Moderate |
| Ji Hoon Sim | Moderate | Moderate | Low | Low | Low | Low | Moderate | Moderate |
| Ji Hoon Sim | Moderate | Moderate | Low | Low | Moderate | Low | Moderate | Moderate |
| Jung-Woo Shim | Low | Moderate | Low | Low | Low | Low | Moderate | Moderate |
| Annika Kurreck | High | Moderate | Low | Moderate | Low | Low | Moderate | High |
| Minjae Kim | Low | Low | Low | Low | Low | Low | Low | Low |
| Bo Rim Kim | Moderate | Moderate | Low | Low | High | Low | Low | High |
| Rita Inácio | Moderate | Moderate | Low | Low | Moderate | Low | Low | Moderate |
| Yan Zhou | Low | Low | Low | Low | Moderate | Low | Low | Low |
| Konlawij Trongtrakul | Moderate | Low | Low | Low | Moderate | Low | Low | Moderate |
| Andrés Zorrilla‑Vaca | Moderate | Low | Low | Low | Moderate | Low | Low | Moderate |
| Masatoshi Nishimoto | Low | Low | Low | Low | Moderate | Low | Low | Low |
| Zayan Mahmooth | Moderate | Moderate | Low | Low | Low | Low | Low | Moderate |
| Gaetan-Romain Joliat | Moderate | Low | Low | Moderate | Moderate | Low | Low | Moderate |
| Nassiba Beghdadi | Low | Low | Low | Moderate | Low | Low | Low | Low |
| Maged Y. Argalious | Low | Moderate | Low | Low | High | Low | Low | Moderate |
| Miho Murashima | Low | Moderate | Low | Low | Moderate | Low | Low | Moderate |
| Hannan Chaudery | Low | Low | Low | Low | Moderate | Low | Low | Low |
| Qin Wu | Moderate | Moderate | Low | Low | Moderate | Low | Low | Moderate |
| Charlotte Slagelse | Low | Low | Low | Low | Moderate | Low | Low | Low |
| Tak Kyu oh | Moderate | High | Low | Low | High | Low | Low | High |
| Shengnan Li | Low | Low | Low | Low | Moderate | Low | Low | Low |
| Nan Li | Moderate | Moderate | Low | Low | Low | Low | Low | Moderate |
| Csaba Kopitko | Moderate | Moderate | Low | Low | Low | Low | Low | Moderate |
| Crystal P. Koerner | Moderate | Moderate | Low | Moderate | Low | Low | Low | Moderate |
| Youn Kyung Kee | Moderate | Moderate | Low | Low | Moderate | Low | Low | Moderate |
| Müzeyyen Iyigun | Moderate | Moderate | Low | Low | Low | Low | Low | Moderate |
| Fabian Grass | Moderate | High | Low | Low | High | Low | Low | High |
| Ivana Dedinská | Moderate | Moderate | Low | Low | Moderate | Low | Low | Moderate |
| Anthony Bonavia | Moderate | Moderate | Low | Moderate | Low | Low | Low | Moderate |
| L. M. Vernooij | Moderate | Moderate | Low | Low | Low | Low | Low | Moderate |
| S. T. Collaborative | Low | Low | Low | Low | Low | Low | Low | Low |
| Leili Pourafkari | Moderate | High | Low | Moderate | Moderate | Moderate | Low | Moderate |
| Yu Zhang | Moderate | Moderate | Low | Low | Low | Low | Low | Moderate |
| Joseph H. Marcotte | Low | Moderate | Low | Moderate | Low | Low | Low | Moderate |
| James G.D.Wiener | Moderate | High | Low | Moderate | Moderate | Moderate | Low | Moderate |
| Mitsuru Ida | Moderate | Moderate | Low | Moderate | Low | Low | Low | Moderate |
| Taryn E Hassinger | Moderate | Low | Low | Moderate | Low | Low | Low | Moderate |
| S.Y. Ham | Moderate | Moderate | Low | Moderate | Low | Low | Low | Moderate |
| Jonathan Garnier | Moderate | High | Low | Moderate | Low | Low | Moderate | High |
| Juan P Cata | Moderate | Moderate | Low | Moderate | Low | Low | Low | Moderate |
| Alexandra Briggs | Low | Moderate | Low | Moderate | Low | Low | Low | Moderate |
| Vicente-Hernández B | High | High | Low | Moderate | High | Moderate | Moderate | High |
| Xing Zhou | Moderate | High | Low | Moderate | Low | Low | Moderate | High |
| Konlawij Trongtrakul | Low | Low | Low | Moderate | Low | Low | Low | Low |
| Yosuke Toyonaga | Moderate | Moderate | Low | Moderate | Moderate | Low | Low | Moderate |
| Vafi Salmasi | Low | Low | Low | Low | Low | Low | Low | Low |
| T. Mizota | Low | Low | Low | Moderate | Low | Low | Low | Low |
| Tyler J. Loftus | Moderate | Moderate | Low | Moderate | Low | Low | Low | Moderate |
| M. E. O'Connor | Low | Low | Low | Low | Low | Low | Low | Low |
| Or Goren | Moderate | Moderate | Low | Moderate | Low | Low | Low | Moderate |
| Yosuke Toyonaga | Moderate | Moderate | Low | Moderate | Moderate | Low | Low | Moderate |
| Fabian Grass | Low | Low | Low | Low | Low | Low | Low | Low |
| A Abrahamsson | High | Moderate | Moderate | High | Moderate | Low | Moderate | High |
| Hung-Chieh Wu | Moderate | Moderate | Low | Moderate | Low | Low | Low | Moderate |
| Stefano Romagnoli | Low | Low | Low | Low | Low | Low | Low | Low |
| Morgan E. Grams | Low | Moderate | Low | Moderate | Low | Low | Low | Low |
| Samuel Quan | Low | High | Low | Moderate | High | Low | Low | High |
| Thorir E Long | Moderate | High | Low | Moderate | High | Low | Low | High |
| Sung Yoon Lim | Moderate | Moderate | Low | Moderate | Low | Low | Low | Moderate |
| Chetana Lim | Low | Low | Low | Moderate | Moderate | Low | Low | Low |
| S.-K. Kim | Low | Low | Low | Moderate | Moderate | Low | Low | Low |
| Ji-Yeon Bang | Moderate | Moderate | Low | Moderate | Moderate | Low | Low | Moderate |
| A. Arjona-Sanchez | Moderate | High | Low | Moderate | High | Low | Low | High |
| Hung-Chieh Wu | Moderate | Moderate | Low | Moderate | Moderate | Low | Low | Moderate |
| David J.R. Morgan | Low | Low | Low | Low | Moderate | Low | Low | Low |
| Miho Tagawa | Low | Moderate | Low | Moderate | Low | Low | Low | Low |
| Arisa Tomozawa | Moderate | Moderate | Low | Moderate | Moderate | Low | Low | Moderate |
| Louise Y. Sun | Low | Low | Low | Moderate | Low | Low | Low | Low |
| Ren Hounu | Moderate | Moderate | Low | Moderate | Moderate | Low | Moderate | Moderate |
| P. Kambakamba | Low | Low | Low | Moderate | Low | Low | Low | Low |
| David R. Mcllroy | Moderate | Moderate | Low | Low | Moderate | Low | Low | Moderate |
| AJ Vaught | Moderate | Moderate | Low | Low | Low | Low | Low | Moderate |
| Eunjung Cho | Low | Low | Low | Low | Low | Low | Low | Low |
| Camilo Correa-Gallego | Low | Low | Low | Low | Low | Low | Low | Low |
| Samira Bell | Low | Low | Low | Low | Moderate | Low | Low | Moderate |
| Shu Li | Moderate | Moderate | Moderate | Moderate | Low | Low | Low | Moderate |
| Catarina Teixeira | Moderate | Low | Low | Low | Low | Low | Low | Low |
| Eun-Ho Lee | Low | Low | Low | Low | Low | Low | Low | Low |
| Murat Biteker | Low | Low | Low | Low | Low | Low | Low | Low |
| Toby N. Weingarten | Moderate | Moderate | Low | Low | Moderate | Low | Low | Moderate |
| Chang Seong Kim | Low | Low | Low | Low | Low | Low | Low | Low |
| Ksenija Slankamenac | Low | Low | Low | Low | Moderate | Low | Low | Low |
| Ian M. Paquette | Moderate | Moderate | Low | Low | Moderate | Low | Low | Moderate |
| Chia-Ter Chao | Low | Low | Low | Low | Low | Low | Low | Low |
| Steven M.Brunelli | Low | Low | Low | Low | Low | Low | Low | Low |
| Laszlo Medve | Moderate | Moderate | Moderate | Moderate | Moderate | Low | Low | Moderate |
| Vin-Cent Wu | Low | Low | Low | Low | Low | Low | Low | Low |
| Ashwin Kurian | High | High | Moderate | Moderate | Moderate | Low | Low | High |
| Marlin Wayne Causey | Moderate | High | Moderate | Moderate | Moderate | Low | Low | Moderate |
| E. Biagioni | High | High | Moderate | High | Moderate | Low | Low | High |
| Enoch Arhinful | Moderate | Moderate | Low | Low | Low | Low | Low | Moderate |
| Steven G. Coca | Low | Low | Low | Low | Low | Low | Low | Low |
| Thomas Armstrong | Moderate | Moderate | Low | Low | Low | Low | Low | Moderate |
| Azra Bihorac | Low | Low | Low | Low | Low | Low | Low | Low |
| Charuhas V.Thakar | Moderate | Moderate | Low | Low | Low | Low | Low | Moderate |
| Christian T.J. Magyar | Moderate | Low | Low | Moderate | Low | Low | Low | Moderate |
| Brandon P. Maddy | Low | Low | Low | Moderate | Low | Low | Low | Low |
| Lingzi Yin | Low | Low | Low | Low | Low | Low | Low | Low |
| Rao Sun | Low | Low | Moderate | Low | Moderate | Low | Low | Moderate |
| Xiran Peng | Low | Low | Low | Low | Low | Low | Low | Low |
| Swapnil Y Parab | Moderate | Low | Low | Low | Low | Low | Low | Moderate |
| Emi Ota | Low | Low | Low | Low | Low | Low | Low | Low |
| Isaac D. Schmied | Low | Low | Moderate | Low | Low | Low | Low | Low |
| David R. McIlroy | Low | Low | Low | Low | Low | Low | Low | Low |
| John D. Mannion | Moderate | Low | Low | Moderate | Moderate | Low | Low | Moderate |
| Kerri Lydon | Low | Low | Low | Low | Low | Low | Low | Low |
| Yunwei Lu | Moderate | Low | Low | Moderate | Low | Low | Low | Moderate |
| Haibei Liu | Low | Low | Low | Low | Low | Low | Low | Low |
| Siqian Li | Low | Low | Low | Low | Low | Low | Low | Low |
| Martin Krause | Low | Low | Low | Low | Low | Low | Low | Low |
| Shun-Cai Gao | Low | Low | Low | Low | Low | Low | Low | Low |
| Yichun Cheng | Low | Low | Low | Low | Low | Low | Low | Low |
| Diego Beltrán | Moderate | Low | Low | Low | Low | Low | Low | Moderate |
| Yao Yu | Low | Low | Low | Low | Low | Low | Low | Low |
| Yang-Tian Ya | Low | Low | Low | Low | Low | Low | Low | Low |
| Oskar Swartling | Low | Low | Low | Low | Low | Low | Low | Low |
| E Rossouw | Moderate | Low | Moderate | Moderate | High | Low | Low | High |
| Zbigniew Putowski | Moderate | Low | Low | Moderate | Low | Low | Low | Moderate |
| Lukas F Liesenfeld | Moderate | Low | Low | Moderate | Low | Low | Low | Moderate |
| Liting Kuang | Low | Low | Low | Low | Low | Low | Low | Low |
| Hye Jin Kim | Low | Low | Low | Low | Low | Low | Low | Low |
| Pyoyoon Kang | Low | Low | Low | Low | Low | Low | Low | Low |
| Yuchen Ji | Low | Low | Low | Low | Low | Low | Low | Low |
| Tasuku Fujii | Moderate | Low | Low | Low | Low | Low | Low | Moderate |
| Diana J. Valencia Morales | Moderate | Low | Low | Low | Low | Low | Low | Moderate |
| Jianghua Shen | Low | Low | Low | Low | Low | Low | Low | Low |
| Zbigniew Putowski | Low | Low | Low | Low | Low | Low | Low | Low |
| Theis B Mikkelsen | Low | Low | Moderate | Low | Moderate | Low | Low | Moderate |
| Conor F. Murphy | Low | Low | Low | Low | Low | Low | Low | Low |
| Anthony Loria | Low | Low | Low | Low | Low | Low | Low | Low |
| Eriya Imai | Low | Low | Low | Low | Low | Low | Low | Low |
| Paul Andrew Drakeford | Low | Low | Low | Low | Low | Low | Low | Low |
| Catherine Chiu | Low | Low | Low | Low | Low | Low | Low | Low |
| STARSurg Collaborative (2022) | Low | Low | Low | Low | Low | Low | Low | Low |
| Bernd Saugel | Low | Low | Low | Low | Low | Low | Low | Low |
| Sina Krone | Low | Low | Low | Low | Low | Low | Low | Low |
| Alexander Zarbock | Low | Low | Low | Low | Low | Low | Low | Low |
| Tetsuya Shimada | Low | Moderate | Low | Moderate | Low | Low | Low | Moderate |
| Judith Schiefer | Low | Low | Low | Low | Low | Low | Low | Low |
| Li Dong | Low | Low | Low | Low | Low | Low | Low | Low |
| Yuanqiang Dai | Low | Low | Low | Low | Moderate | Low | Low | Moderate |
| Amjad Bani Hani | Moderate | Low | Moderate | Low | Moderate | Moderate | Low | Moderate |
| Jiao‑Nan Yang | Low | Low | Low | Low | Low | Low | Low | Low |
| Jiaqi Wang | Low | Low | Moderate | Low | Low | Low | Low | Moderate |
| Tetsuya Shimada | Low | Low | Low | Low | Low | Low | Low | Low |
| Hirotaka Okuda | Moderate | Low | Low | Low | Low | Low | Low | Moderate |
| Linhui Hu | Low | Low | Low | Low | Low | Low | Low | Low |
| W. Brenton French | Low | Low | Low | Low | Low | Low | Low | Low |
| Wendy De Guglielmo | Moderate | Low | Moderate | Low | Moderate | Moderate | Low | Moderate |
| J Ripollés-Melchor | Low | Low | Low | Low | Low | Low | Low | Low |
| Kazuyuki Mizunoya | Low | Low | Low | Low | Low | Low | Low | Low |
| Henrique Tadashi Katayama | Low | Low | Low | Low | Low | Low | Low | Low |
| Bertha M. Cordova-Sánchez | Low | Low | Low | Low | Low | Low | Low | Low |
| Anthony Loria | Low | Low | Low | Low | Low | Low | Low | Low |
| Karthik V Iyer | Low | Low | Low | Low | Low | Low | Low | Low |
| Seokyung Shin | Low | Low | Low | Low | Moderate | Low | Low | Moderate |
| Thomas G. Matkov | Moderate | Low | Moderate | Low | Low | Low | Low | Moderate |

Domains

D1: Risk of bias due to confounding

D2: Risk of bias arising from measurement of the exposure

D3: Risk of bias in selection of participants into the study (or into the analysis)

D4: Risk of bias due to post-exposure interventions

D5: Risk of bias due to missing data

D6: Risk of bias arising from measurement of the outcomes

D7: Risk of bias in selection of the reported results

**Table S5. Number of patients by stage of post-operative AKI (For citations see main manuscript)**

| Study | Year | Operation | AKI definition | No. of patients with AKI | Stage 1 or RIFLE –R | Stage 2 or RIFLE – I | Stage 3 or RIFLE-F |
| --- | --- | --- | --- | --- | --- | --- | --- |
| Annika Kurreck | 2022 | CRS and HIPEC | AKIN | 62 | 32 | 17 | 13 |
| Minjae Kim | 2021 | Abdominal surgery | KDIGO | 234 | 170 | 37 | 27 |
| Bo Rim Kim | 2021 | Open abdominal surgery | KDIGO | 238 | 190 | 24 | 24 |
| Rita Inácio | 2021 | Major abdominal surgery | KDIGO | 32 | 14 | 8 | 10 |
| Zayan Mahmooth | 2020 | Pancreatoduodenectomy | KDIGO | 78 | 64 | 6 | 8 |
| Mitsuru Ida | 2020 | Pancreas surgery | KDIGO | 21 | 18 | 3 | 0 |
| Hannan Chaudery | 2019 | Abdominal surgery | KDIGO | 174 | 83 | 48 | 43 |
| T.K. Oh | 2019 | Laparoscopic abdominal surgery | KDIGO | 186 | 146 | 29 | 11 |
| Crystal P.Koerner | 2019 | Abdominal surgery | KDIGO | 44 | 30 | 11 | 3 |
| Fabian Grass | 2019 | Colorectal surgery | RIFLE | 104 | 82 | 21 | 1 |
| S. T. Collaborative | 2018 | Major gastrointestinal surgery | KDIGO | 646 | 438 | 113 | 95 |
| Jonathan Garnier | 2018 | Hepatectomy | KDIGO | 24 | 15 | 4 | 5 |
| Yu Zhang | 2018 | Abdominal surgery | KDIGO | 281 | 144 | 61 | 76 |
| Xiujuan Wu | 2017 | Gastrointestinal surgery | KDIGO | 71 | 49 | 22 | 0 |
| Tyler J.Loftus | 2017 | Open abdominal surgery | KDIGO | 143 | 35 | 53 | 55 |
| Vicente-Hernández B | 2017 | Appendicectomy | KDIGO | 21 | 10 | 11 | 0 |
| Sebastian Schmid | 2016 | Major abdominal surgery | KDIGO | 99 | 61 | 34 | 4 |
| Ana B Serrano | 2016 | Open abdominal surgery | AKIN | 28 | 23 | 3 | 2 |
| Stefano Romagnoli | 2016 | Abdominal surgery | AKIN | 31 | 31 | 0 | 0 |
| Samuel Quan | 2016 | Abdominal surgery | KDIGO | 200 | 156 | 25 | 19 |
| Thorir E Long | 2016 | Abdominal surgery | KDIGO | 264 | 172 | 49 | 43 |
| Sung Yoon Lim | 2016 | Rectal surgery | AKIN | 11 | 6 | 1 | 4 |
| S.-K.Kim | 2016 | Hepatectomy | AKIN | 68 | 67 | 1 | 0 |
| A. Arjona-Sanchez | 2016 | CRS and HIPEC | RIFLE | 61 | 18 | 26 | 17 |
| Morgan E. Grams | 2016 | General (gastrointestinal/ abdominal) surgery | KDIGO | 5887 | 4192 | 981 | 669 |
| AJ Vaught | 2015 | Gynecological surgery | RIFLE | 295 | 185 | 65 | 45 |
| Camilo Correa-Gallego | 2015 | Liver resection | RIFLE | 336 | 278 | 49 | 9 |
| Hung-Chieh Wu | 2015 | Abdominal surgery | KDIGO | 39 | 13 | 12 | 14 |
|  |  | Gynecological surgery |  | 4 | 0 | 2 | 2 |
| David J.R. Morgan | 2015 | Bariatric surgery | AKIN | 103 | 82 | 9 | 12 |
| Ren Hounu | 2015 | Esophageal surgery | KDIGO | 23 | 13 | 7 | 3 |
| Eun-Ho Lee | 2014 | Esophageal Surgery | AKIN | 210 | 180 | 16 | 14 |
| Eunjung Cho | 2014 | Hepatobiliary surgery | AKIN | 10 | 5 | 2 | 3 |
| Chia-Ter Chao | 2013 | Abdominal surgery | AKIN | 400 | 238 | 37 | 125 |
| Chang Seong Kim | 2013 | Gastrectomy | KDIGO | 679 | 589 | 61 | 29 |
| Toby N.Weingarten | 2013 | Bariatric surgery | AKIN | 71 | 66 | 3 | 2 |
| Laszlo Medve | 2012 | Abdominal surgery | AKIN | 39 | 20 | 10 | 9 |
| Azra Bihorac | 2009 | General/gastrointestinal surgery | RIFLE | 918 | 370 | 300 | 248 |
| Thomas Armstrong | 2009 | Liver resection | AKIN | 78 | 61 | 12 | 5 |
| Christian T.J. Magyar | 2025 | laparoscopic liver resection | KDIGO | 16 | 3 | 6 | 7 |
| Lingzi Yin | 2025 | major abdominal surgery | KDIGO | 44 | 37 | 7 | 0 |
| Rao Sun | 2024 | major abdominal surgery | KDIGO | 144 | 124 | 16 | 4 |
| Swapnil Y Parab | 2024 | elective oesophagectomy surger | KDIGO | 27 | 15 | 7 | 5 |
| David R. McIlroy | 2024 | Major Abdominal Surgery | KDIGO | 318 | 223 | 59 | 36 |
| Kerri Lydon | 2024 | pancreatic surgery | KDIGO | 20 | 16 | 1 | 3 |
| Yunwei Lu | 2024 | CRS-HIPEC | KDIGO | 34 | 21 | 6 | 7 |
| Shun-Cai Gao | 2024 | CRS-HIPEC | KDIGO | 51 | 42 | 8 | 1 |
| Yao Yu | 2023 | liver resection | KDIGO | 23 | 19 | 4 | 0 |
| Oskar Swartling | 2023 | pancreatoduodenectomy | KDIGO | 137 | 73 | 39 | 25 |
| E Rossouw | 2023 | abdominal surgery | KDIGO | 82 | 32 | 26 | 24 |
| Zbigniew Putowski | 2023 | Pancreaticoduodenectomy | KDIGO | 58 | 47 | 9 | 2 |
| Lukas F Liesenfeld | 2023 | CRS-HIPEC | KDIGO | 37 | 15 | 2 | 20 |
| Liting Kuang | 2023 | hepatectomy | KDIGO | 148 | 135 | 9 | 4 |
| Pyoyoon Kang | 2023 | abdominal surgery | KDIGO | 123 | 101 | 17 | 5 |
| Yuchen Ji | 2023 | pancreaticoduodenectomy | KDIGO | 141 | 112 | 21 | 8 |
| Zbigniew Putowski | 2022 | abdominal surgery | KDIGO | 32 | 24 | 5 | 3 |
| Theis B Mikkelsen | 2022 | major emergency abdominal surgery | KDIGO | 122 | 82 | 26 | 14 |
| Conor F. Murphy | 2022 | Esophageal Cancer Surgery | AKIN | 208 | 173 | 28 | 7 |
| Anthony Loria | 2022 | ileostomy formation | KDIGO | 51 | 38 | 8 | 5 |
| Eriya Imai | 2023 | laparoscopic liver resection | KDIGO | 34 | 31 | 3 | 0 |
| Paul Andrew Drakeford | 2022 | colorectal surgery | KDIGO | 74 | 62 | 11 | 1 |
| Catherine Chiu | 2022 | major abdominal surgery | KDIGO | 3991 | 3385 | 456 | 150 |
| Judith Schiefer | 2023 | abdominal surgery | KDIGO | 385 | 192 | 62 | 131 |
| W. Brenton French | 2022 | abdominal surgery | KDIGO | 215 | 163 | 32 | 20 |
| Bertha M. C ordova-Sánchez | 2021 | abdominal surgery | KDIGO | 196 | 88 | 53 | 55 |
| Karthik V Iyer | 2025 | major gastro-intestinal surgery | KDIGO | 83 | 52 | 27 | 4 |
| Seokyung Shin | 2025 | major hepatectomy | KDIGO | 154 | 105 | 35 | 14 |

Abbreviations: CRS, cytoreductive surgery; HIPEC, hyperthermic intraperitoneal chemotherapy.

**Table S6. Hospital length of stay (days) of patients who developed post-operative AKI and those who did not (For citations see main manuscript)**

| **Study** | **Year** | **Operation** | **Length of stay with AKI Mean (SD) or Median (IQR)** | **Length of stay without AKI Mean (SD) or Median (IQR)** |
| --- | --- | --- | --- | --- |
| Andrs Zorrilla‑Vaca | 2021 | Colorectal surgery | 23.2 (18.9) | 10.1 (11.4) |
| Rita Inácio | 2021 | Abdominal surgery | 43.2 (27.7) | 31.6 (26.1) |
| Csaba Kopitko | 2019 | Abdominal surgery | 13 (8-18.5) | 11 (9-14) |
| Fabian Grass | 2019 | Colorectal surgery | 8 (7) | 5 (4) |
| Joseph H. Marcotte | 2018 | Colorectal surgery | 5.11 | 8.4 |
| Taryn E Hassinger | 2018 | Colorectal surgery | 11 (7 - 20) | 6 (4 - 9) |
| Xing Zhou | 2017 | Hepatectomy | 18 (12–26) | 15 (3–50) |
| Tyler J.Loftus | 2017 | Open abdominal surgery | 23 (12–33) ^a^ | 22 (11–33) ^b^ |
| Or Goren | 2017 | Pancreatic surgery | 19.2(18.0) | 19.1(16.8) |
| Yosuke Toyonaga | 2017 | Abdominal surgery | 48.4 (37.0) | 34.3 (25.5) |
| T.Mizota | 2017 | Major abdominal surgery | 26 | 15 |
| Mitsuru Ida | 2020 | Pancreas surgery | 27.6 (15.1) | 21.1 (10.4) |
| Sung Yoon Lim | 2016 | Rectal surgery | 26 (18) | 16 (10) |
| Chetana Lim | 2016 | Hepatectomy | 20 (18) | 12 (9) |
| David J.R. Morgan | 2015 | Bariatric surgery | 18.6 (27.90) | 6.6 (8.80) |
| Arisa Tomozawa | 2015 | Liver resection | 26.5 (16-45) | 19 (14-29) |
| AJ Vaught | 2015 | Gynecological surgery | 10 (7-17) | 4 (3-5) |
| P. Kambakamba | 2015 | Liver resection | 18 (12–26.5) | 11 (9–16) |
| Eun-Ho Lee | 2014 | Esophageal surgery | 14 (11-21) | 12 (11-16) |
| Catarina Teixeira | 2014 | Abdominal surgery | 15.1(6.2) | 11.1(1.3) |
| Chang Seong Kim | 2013 | Gastrectomy | 18.7 (20.5) | 12（(7.1) |
| Toby N. Weingarten | 2013 | Bariatric surgery | 5 (3-7) | 3 (2-5) |
| Laszlo Medve | 2012 | Abdominal surgery | 18(13-24) | 11(9-14) |
| Charuhas V. Thakar | 2007 | Bariatric surgery | 4 (2.1) | 2.7 (1.15) |
| Brandon P. Maddy | 2025 | Gynecologic oncology surgery | 8 (3.7) | 5 (1.5) |
| Swapnil Y Parab | 2024 | Oesophagectomy surger | 13 (7.8) | 9 (3) |
| Kerri Lydon | 2024 | Pancreatic surgery | 14 (5.9) | 10 (5.9) |
| Yunwei Lu | 2024 | CRS-HIPEC | 18.6 (7.6) | 22 (19.1) |
| Shun-Cai Gao | 2024 | CRS-HIPEC | 17 (5.9) | 16 (4.4) |
| Liting Kuang | 2023 | Hepatectomy | 21.34 (9.93) | 17.76 (7.99) |
| Pyoyoon Kang | 2023 | Abdominal surgery | 20.13 (18.18) | 12.43 (10.95) |
| Conor F. Murphy | 2022 | Esophageal cancer surgery | 17 (14) | 14 (9) |
| Anthony Loria | 2022 | Ileostomy formation | 14 (14.8) | 8 (5.2) |

^a^ Patients with postoperative AKI were defined as stage 2 and 3. ^b^ Patients without postoperative was defined as non-AKI and stage 1.

**Table S7. Mortality of patients who developed post-operative AKI and those who did not (For citations see main manuscript)**

| Study | No. of patients | Mortality definition | Deaths with AKI | Survivals with AKI | Deaths without AKI | Survivals without AKI |
| --- | --- | --- | --- | --- | --- | --- |
| AJ Vaught | 2341 | Hospital | 20 | 275 | 2 | 2044 |
|  |  | 90 days | 29 | 266 | 10 | 2036 |
| Catarina Teixeira | 450 | Hospital | 21 | 80 | 8 | 341 |
| P. Kambakamba | 829 | Hospital | 14 | 54 | 2 | 759 |
| Chang Seong Kim | 4718 | Hospital | 24 | 655 | 7 | 4032 |
|  |  | 90 days | 26 | 653 | 11 | 4028 |
| Arisa Tomozawa | 642 | Hospital | 11 | 67 | 13 | 551 |
| Andrés Zorrilla‑Vaca | 1652 | Hospital | 15 | 112 | 15 | 1510 |
| Rita Inácio | 165 | Hospital | 10 | 22 | 17 | 116 |
| Csaba Kopitko | 84 | Hospital | 13 | 26 | 5 | 40 |
| Alexandra Briggs | 59604 | 90 days | 1310 | 2244 | 6894 | 49156 |
|  |  | 1 year | 1585 | 1969 | 10089 | 45961 |
| Tyler J. Loftus | 251 | hospital | 21* | 49 | 14** | 167 |
| Sung Yoon Lim | 288 | hospital | 2 | 9 | 0 | 277 |
| E. Biagioni | 60 | hospital | 16 | 13 | 4 | 27 |
| Marlin Wayne Causey | 339 | hospital | 9 | 31 | 11 | 288 |
| Eun-Ho Lee | 595 | 30 days | 10 | 200 | 8 | 377 |
| Chetana Lim | 457 | 90 days | 25 | 42 | 23 | 367 |
| David J.R. Morgan | 590 | ≥ 1year | 6 | 97 | 8 | 479 |
| Yu Zhang | 619 | ICU | 29 | 252 | 3 | 335 |
|  |  | hospital | 44 | 237 | 15 | 323 |
|  |  | 28 days | 46 | 235 | 12 | 326 |
| Laszlo Medve | 145 | ICU | 12 | 27 | 4 | 102 |
|  |  | hospital | 15 | 24 | 5 | 101 |
| Or Goren | 153 | hospital | 4 | 11 | 4 | 134 |
| Yosuke Toyonaga | 206 | ICU | 0 | 42 | 1 | 163 |
|  |  | hospital | 1 | 41 | 3 | 161 |
| Taryn E Hassinger | 2910 | 30 days | 13 | 322 | 16 | 2559 |
| T. Mizota | 3650 | hospital | 15 | 211 | 27 | 3307 |
| Charuhas V. Thakar | 491 | hospital | 2 | 102 | 0 | 449 |
| Swapnil Y Parab | 183 | hospital | 4 | 23 | 2 | 154 |
| Kerri Lydon | 200 | hospital | 3 | 17 | 1 | 179 |
| Yunwei Lu | 158 | hospital | 5 | 29 | 0 | 124 |
| Liting Kuang | 1949 | hospital | 1 | 147 | 1 | 1800 |
| Pyoyoon Kang | 1586 | hospital | 7 | 116 | 8 | 1455 |
| Yuchen Ji | 1312 | hospital | 16 | 125 | 11 | 1160 |
| Diana J. Valencia Morales | 365 | hospital | 4 | 38 | 1 | 322 |
| Theis B Mikkelsen | 703 | hospital | 34 | 88 | 27 | 554 |
| Theis B Mikkelsen | 703 | 90 days | 41 | 81 | 40 | 541 |
| Conor F. Murphy | 1135 | hospital | 7 | 201 | 17 | 910 |

* Patients with postoperative AKI are defined as stage 2 and 3. ** Patients without postoperative defined as non-AKI and stage 1.

**Table S8. Complications by AKI stage in abdominal surgery patients**

| study | year | Complication | No AKI | All AKI | | AKI  Stage 1 | AKI  Stage 2 | AKI  Stage3 | | P value | |
| --- | --- | --- | --- | --- | --- | --- | --- | --- | --- | --- | --- |
| AJ Vaught | 2015 | Mechanical wound complications, n (%) | 30 (1) | 23 (8) | | 9 (5) | 4 (6) | 10 (22) | | <0.0083 | |
|  |  | Surgical infections, n (%) | 56 (3) | 18 (6) | | 7 (4) | 8 (12) | 6 (13) | | <0.0083 | |
|  |  | Pulmonary complications, n (%) | 50 (2) | 44 (15) | | 20 (11) | 10 (15) | 14 (31) | | <0.0083 | |
|  |  | Cardiovascular complications, n (%) | 30 (1) | 17 (6) | | 6 (3) | 7 (10) | 4 (9) | | <0.0083 | |
|  |  | Venous thromboembolism, n (%) | 15 (0.7) | 14 (5) | | 8 (4) | 2 (3) | 4 (9) | | <0.0083 | |
|  |  | Gastrointestinal complications, n (%) | 62 (3) | 36 (12) | | 23 (12) | 6 (9) | 7 (16) | | <0.0083 | |
| Yuchen Ji | 2022 | Clavien–Dindo classification grade≥III, n (%) | 61 (5.2) | 21 (14.9) | | 11 (9.8) | 7 (33.3) | 33 (37.5) | | 0.004 | |
| Theis B Mikkelsen | 2022 | Surgical complications, n (%) | 107 (18.4) NA | | 19 (23.2) | | 2 (7.7) | 4 (28.6) | 0.26 | |  |

**Table S9. Influence factors of AKI after abdominal surgery (For citations see main manuscript)**

| **Risk factor** | **Author** | **Year** | **OR (95%CI)** |
| --- | --- | --- | --- |
| Age (per 1 year) | Jianjun Xu | 2021 | 0.99 (0.93-1.06) ^b^ |
|  | Ji Hoon Sim | 2021 | 1.01 (0.98-1.04) ^b^ |
|  | Ji Hoon Sim | 2021 | 1.00 (0.99-1.02) ^b^ |
|  | Bo Rim Kim | 2021 | 1.03 (1.02-1.04) ^a^ |
|  | Rita Inácio | 2021 | 1.06 (1.02-1.10) ^b^ |
|  | Xiaowei Guo | 2020 | 1.00 (0.92-1.09) ^a^ |
|  | Tak Kyu oh | 2019 | 1.01 (0.99-1.02) ^b^ |
|  | Taryn E Hassinger | 2018 | 1.03 (1.02-1.04) ^b^ |
|  | S.Y. Ham | 2018 | 0.97 (0.92-1.02) ^a^ |
|  | Alexandra Briggs | 2018 | 1.01 (1.01-1.01) ^a^ |
|  | Xing Zhou | 2017 | 1.05 (1.01-1.10) ^a^ |
|  | Or Goren | 2017 | 1.10 (0.90-1.10) ^b^ |
|  | Stefano Romagnoli | 2016 | 1.09 (1.03-1.15) ^b^ |
|  | Thorir E Long | 2016 | 1.01 (1.00-1.03) ^b^ |
|  | Chetana Lim | 2016 | 1.03 (1.00-1.05) ^b^ |
|  | Ji-Yeon Bang | 2016 | 1.02 (1.01-1.03) ^b^ |
|  | AJ Vaught | 2015 | 1.02 (1.01-1.03) ^b^ |
|  | Eunjung Cho | 2014 | 1.04 (1.01-1.08) ^b^ |
|  | Catarina Teixeira | 2014 | 1.02 (1.01-1.05) ^b^ |
|  | Tyler J. Loftus | 2017 | 1.03 (1.02-1.05) ^b c^ |
|  | A. Arjona-Sanchez | 2016 | 1.01 (0.97-1.05) ^a d^ |
| Male | Ji Hoon Sim | 2021 | 2.06 (0.81-5.26) ^b^ |
|  | Ji Hoon Sim | 2021 | 1.60 (1.19-2.14) ^b^ |
|  | Jung-Woo Shim | 2021 | 3.04 (1.77-5.21) ^b^ |
|  | Bo Rim Kim | 2021 | 1.82 (1.36-2.44) ^a^ |
|  | Andrés Zorrilla‑Vaca | 2021 | 2.33 (1.36-4.01) ^b^ |
|  | Tak Kyu oh | 2019 | 1.47 (1.02-2.11) ^b^ |
|  | Taryn E Hassinger | 2018 | 1.57 (1.21-2.04) ^b^ |
|  | Alexandra Briggs | 2018 | 0.98 (0.91-1.05) ^a^ |
|  | Or Goren | 2017 | 0.80 (0.30-2.20) ^a^ |
|  | Catarina Teixeira | 2014 | 1.50 (0.90-2.40) ^a^ |
|  | Chang Seong Kim | 2013 | 1.75 (1.37-2.23) ^b^ |
|  | Toby N. Weingarten | 2013 | 1.34 (0.69-2.63) ^b^ |
|  | Ian M. Paquette | 2013 | 1.00 (0.40-2.81) ^a^ |
| Female | Annika Kurreck | 2022 | 0.74 (0.34-1.62) ^b^ |
|  | Minjae Kim | 2021 | 0.77 (0.57-1.04) ^b^ |
|  | S. T. Collaborative | 2018 | 0.76 (0.63-0.90) ^b^ |
|  | Thorir E Long | 2016 | 0.68 (0.47-0.98) ^b^ |
|  | Arisa Tomozawa | 2015 | 0.53 (0.28-1.03) ^b^ |
| BMI (kg/m^2^) | Ji Hoon Sim | 2021 | 1.09 (0.98-1.21) ^b^ |
|  | Ji Hoon Sim | 2021 | 1.06 (1.02-1.11) ^b^ |
|  | Tak Kyu oh | 2019 | 0.98 (0.94-1.03) ^b^ |
|  | Taryn E Hassinger | 2018 | 1.02 (1.01-1.04) ^b^ |
|  | Stefano Romagnoli | 2016 | 1.12 (1.02-1.24) ^b^ |
|  | Eun-Ho Lee | 2014 | 1.07 (1.01-1.14) ^b^ |
|  | Ian M. Paquette | 2013 | 0.92 (0.82-1.03) ^b^ |
|  | Charuhas V. Thakar | 2007 | 1.03 (1.00-1.06) ^b^ |
| Chronic kidney disease | Andrés Zorrilla‑Vaca | 2021 | 2.45 (1.42-4.23) ^b^ |
|  | Rita Inácio | 2021 | 2.36 (0.73-7.65) ^b^ |
|  | Zayan Mahmooth | 2020 | 0.87 (0.39-1.96) ^a^ |
|  | Alexandra Briggs | 2018 | 4.18 (3.89-4.49) ^b^ |
|  | Or Goren | 2017 | 5.30 (0.80-37.10) ^b^ |
|  | P. Kambakamba | 2015 | 3.18 (1.64-6.15) ^b^ |
| Diabetes mellitus | Annika Kurreck | 2022 | 1.17 (0.27-5.17) ^b^ |
|  | Andrés Zorrilla‑Vaca | 2021 | 1.01 (0.59-1.73) ^b^ |
|  | Jianjun Xu | 2021 | 0.40 (0.07-2.35) ^b^ |
|  | Ji Hoon Sim | 2021 | 2.77 (1.16-6.58) ^b^ |
|  | Ji Hoon Sim | 2021 | 1.48 (1.10-1.98) ^b^ |
|  | Minjae Kim | 2021 | 1.38 (0.98-1.94) ^b^ |
|  | Bo Rim Kim | 2021 | 1.97 (1.50-2.59) ^a^ |
|  | Xiaowei Guo | 2020 | 1.72 (0.52-5.73) ^a^ |
|  | Tak Kyu oh | 2019 | 0.96 (0.64-1.44) ^a^ |
|  | Fabian Grass | 2019 | 2.48 (1.53-4.02) ^b^ |
|  | Xing Zhou | 2017 | 1.50 (0.41-5.47) ^a^ |
|  | Or Goren | 2017 | 0.80 (0.30-2.80) ^a^ |
|  | Thorir E Long | 2016 | 2.54 (1.40-4.35) ^a^ |
|  | Sung Yoon Lim | 2016 | 3.49 (0.84-14.42) ^b^ |
|  | Ji-Yeon Bang | 2016 | 1.53 (1.17-2.00) ^b^ |
|  | AJ Vaught | 2015 | 1.40 (0.98-1.99) ^b^ |
|  | Toby N. Weingarten | 2013 | 2.77 (1.36-5.65) ^b^ |
|  | A. Arjona-Sanchez | 2016 | 1.20 (0.28-5.06) ^a d^ |
| Hypertension | Annika Kurreck | 2022 | 5.28 (2.37-11.78) ^b^ |
|  | Andrés Zorrilla‑Vaca | 2021 | 1.02 (0.59-1.76) ^b^ |
|  | Jianjun Xu | 2021 | 9.37 (2.26-38.81) ^b^ |
|  | Ji Hoon Sim | 2021 | 1.60 (0.66-3.91) ^a^ |
|  | Ji Hoon Sim | 2021 | 1.46 (1.12-1.91) ^b^ |
|  | Minjae Kim | 2021 | 1.45 (1.02-2.06) ^b^ |
|  | Bo Rim Kim | 2021 | 2.16 (1.66-2.82) ^a^ |
|  | Xiaowei Guo | 2020 | 1.88 (0.62-5.66) ^b^ |
|  | Tak Kyu oh | 2019 | 1.15 (0.79-1.67) ^b^ |
|  | Taryn E Hassinger | 2018 | 1.55 (1.16-2.08) ^b^ |
|  | Xing Zhou | 2017 | 5.85 (1.60-21.42) ^b^ |
|  | Or Goren | 2017 | 1.60 (0.50-4.60) ^a^ |
|  | Thorir E Long | 2016 | 1.75 (1.10-2.74) ^b^ |
|  | Ji-Yeon Bang | 2016 | 1.44 (1.14-1.82) ^b^ |
|  | Arisa Tomozawa | 2015 | 2.10 (1.11-3.97) ^b^ |
|  | Chang Seong Kim | 2013 | 1.27 (1.04-1.54) ^b^ |
|  | Toby N. Weingarten | 2013 | 1.78 (0.78-4.05) ^b^ |
|  | A. Arjona-Sanchez | 2016 | 1.50 (0.69-3.35) ^a d^ |
| COPD | Andrés Zorrilla‑Vaca | 2021 | 1.44 (0.84-2.47) ^b^ |
|  | Bo Rim Kim | 2021 | 1.05 (0.51-2.19) ^a^ |
|  | Rita Inácio | 2021 | 4.26 (1.02-17.45) ^b^ |
|  | Xiaowei Guo | 2020 | 1.54 (0.40-5.92) ^a^ |
|  | Taryn E Hassinger | 2018 | 0.97 (0.59-1.59) ^b^ |
|  | Thorir E Long | 2016 | 1.54 (0.78-2.87) ^b^ |
|  | Chang Seong Kim | 2013 | 1.64 (1.15-2.35) ^b^ |
| Congestive heart failure | Bo Rim Kim | 2021 | 3.76 (1.25-11.24) ^b^ |
|  | Taryn E Hassinger | 2018 | 6.51 (2.60-16.29) ^b^ |
|  | Thorir E Long | 2016 | 3.07 (1.74-5.14) ^a^ |
|  | AJ Vaught | 2015 | 2.25 (1.23-4.11) ^b^ |
| Dyslipidemia | Tak Kyu oh | 2019 | 0.79 (0.38-1.63) ^a^ |
|  | Or Goren | 2017 | 0.80 (0.30-2.80) ^a^ |
|  | Toby N. Weingarten | 2013 | 0.91 (0.43-1.91) ^b^ |
|  | Charuhas V. Thakar | 2007 | 2.74 (1.33-5.67) ^b^ |
| Preoperative albumin (per 1 g/dl) | Ji Hoon Sim | 2021 | 0.33 (0.17-0.62) ^b^ |
|  | Ji Hoon Sim | 2021 | 0.55 (0.44-0.69) ^a^ |
|  | Bo Rim Kim | 2021 | 0.39 (0.29-0.53) ^b^ |
|  | Yosuke Toyonaga | 2017 | 0.80 (0.30-1.60) ^b^ |
|  | Arisa Tomozawa | 2015 | 0.71 (0.37-1.36) ^b^ |
|  | Eun-Ho Lee | 2014 | 0.52 (0.33-0.84) ^b^ |
| Hypoalbuminemia | Andrés Zorrilla‑Vaca | 2021 | 1.68 (1.02-2.79) ^b^ |
|  | Chang Seong Kim | 2013 | 1.40 (1.11-1.77) ^b^ |
| Preoperative hemoglobin (per 1 g/dl) | Ji Hoon Sim | 2021 | 0.96 (0.89-1.04) ^b^ |
|  | Bo Rim Kim | 2021 | 0.85 (0.79-0.91) ^a^ |
|  | Xing Zhou | 2017 | 0.97 (0.94-1.00) ^b^ |
|  | Yosuke Toyonaga | 2017 | 0.70 (0.50-1.00) ^b^ |
|  | Arisa Tomozawa | 2015 | 0.91 (0.76-1.09) ^b^ |
|  | Catarina Teixeira | 2014 | 1.00 (0.80-1.20) ^b^ |
| Perioperative NSAID | Bo Rim Kim | 2021 | 1.38 (0.96-1.98) ^a^ |
|  | Tak Kyu oh | 2019 | 1.21 (0.86-1.71) ^b^ |
|  | A Abrahamsson | 2017 | 5.42 (1.34-21.90) ^a^ |
| Preoperative ACEi/ARB | Bo Rim Kim | 2021 | 1.68 (1.26-2.23) ^a^ |
|  | S. T. Collaborative | 2018 | 1.30 (1.07-1.59) ^b^ |
|  | Ji-Yeon Bang | 2016 | 1.86 (1.42-2.43) ^a^ |
|  | Arisa Tomozawa | 2015 | 1.07 (0.56-2.04) ^b^ |
|  | Eun-Ho Lee | 2014 | 1.35 (1.05-1.75) ^b^ |
|  | Charuhas V. Thakar | 2007 | 2.17 (1.11-4.24) ^b^ |
| Preoperative β blocker | Bo Rim Kim | 2021 | 1.91 (1.21-3.02) ^a^ |
|  | Ji-Yeon Bang | 2016 | 2.13 (1.55-2.92) ^a^ |
| Preoperative statin | Bo Rim Kim | 2021 | 2.28 (1.38-3.76) ^a^ |
|  | Ji-Yeon Bang | 2016 | 1.70 (1.22-2.36) ^a^ |
|  | Tak Kyu oh | 2019 | 1.21 (0.86-1.71) ^b^ |
|  | A Abrahamsson | 2017 | 5.42 (1.34-21.90) ^a^ |
| Functionally dependent | Taryn E Hassinger | 2018 | 1.65 (0.96-2.85) ^b^ |
|  | Taryn E Hassinger | 2018 | 2.93 (1.42-6.06) ^b^ |
|  | Minjae Kim | 2021 | 2.05 (1.24-3.38) ^b^ |
| Perioperative exposure to contrast | Tak Kyu oh | 2019 | 1.83 (1.03-3.27) ^b^ |
|  | Chang Seong Kim | 2013 | 1.60 (1.29-2.00) ^b^ |
| PNI | Ji Hoon Sim | 2021 | 0.92 (0.85-0.99) ^b^ |
|  | Ji Hoon Sim | 2021 | 0.96 (0.93-0.99) ^b^ |
| Malignancy | Zayan Mahmooth | 2020 | 0.81 (0.38-1.74) ^b^ |
|  | Tak Kyu oh | 2019 | 0.43 (0.30-0.63) ^b^ |
|  | Taryn E Hassinger | 2018 | 1.17 (0.72-1.90) ^b^ |
|  | Thorir E Long | 2016 | 1.27 (0.87-1.82) ^a^ |
|  | Alexandra Briggs | 2018 | 1.83 (1.70-1.97) ^b^ |
|  | AJ Vaught | 2015 | 1.80 (1.17-2.76) ^b^ |
|  | Catarina Teixeira | 2014 | 1.40 (0.80-2.40) ^b^ |
| Sepsis | Taryn E Hassinger | 2018 | 2.19 (1.13-4.27) ^a^ |
|  | Alexandra Briggs | 2018 | 5.28 (4.90-5.69) ^a^ |
|  | Tyler J. Loftus | 2017 | 2.53 (1.36-4.69) ^a c^ |
| Perioperative diuretics use | Ji Hoon Sim | 2021 | 1.57 (0.35-5.19) ^b^ |
|  | Minjae Kim | 2021 | 2.08 (1.10-3.90) ^b^ |
|  | Bo Rim Kim | 2021 | 3.27 (1.43-7.51) ^a^ |
|  | Or Goren | 2017 | 20.50 (1.70-243.00) ^a^ |
|  | Ji-Yeon Bang | 2016 | 2.44 (1.15-5.17) ^b^ |
|  | Chang Seong Kim | 2013 | 2.39 (1.98-2.88) ^b^ |
| Intraoperative vasopressor infusion | Bo Rim Kim | 2021 | 2.36 (1.52-3.68) ^a^ |
|  | Bo Rim Kim | 2021 | 3.99 (2.42-6.58) ^a^ |
|  | Zayan Mahmooth | 2020 | 3.08 (0.86-11.12) ^b^ |
|  | Tak Kyu oh | 2019 | 4.19 (2.15-8.16) ^b^ |
|  | S.Y. Ham | 2018 | 0.57 (0.11-2.82) ^a^ |
|  | T. Mizota | 2017 | 1.67 (1.15-2.44) ^b^ |
|  | Or Goren | 2017 | 1.30 (0.40-4.10) ^a^ |
|  | Catarina Teixeira | 2014 | 1.10 (0.50-2.00) ^b^ |
|  | Chang Seong Kim | 2013 | 1.87 (1.28-2.72) ^b^ |
| Operating time > 180 mins | Annika Kurreck | 2022 | 2.13 (0.99-4.58) ^b^ |
|  | Zayan Mahmooth | 2020 | 1.57 (0.84-2.96) ^b^ |
|  | Fabian Grass | 2019 | 1.91 (1.28-2.88) ^b^ |
|  | Chetana Lim | 2016 | 2.40 (1.00-5.90) ^b^ |
| Total fluids (ml/kg) | Ji Hoon Sim | 2021 | 1.00 (0.99-1.02) ^b^ |
|  | Ji Hoon Sim | 2021 | 1.00 (0.98-1.02) ^b^ |
| Intraoperative fluids ≥ 1650ml (ref. < 1650ml) | Andrés Zorrilla‑Vaca | 2021 | 1.06 (0.66-1.71) ^b^ |
| Restrictive fluid (ref. ultra-restrictive) | Zayan Mahmooth | 2020 | 0.74 (0.37-1.49) ^b^ |
| non-restrictive (ref. ultra-restrictive) | Zayan Mahmooth | 2020 | 0.51 (0.21-1.21) ^b^ |
| Intraoperative liquid volume (liter) | A Abrahamsson | 2017 | 1.50 (0.95-2.36) ^a^ |
| Intraoperative colloid volume (per 100ml) | Eun-Ho Lee | 2014 | 1.11 (1.06-1.18) ^b^ |
| Fluid balance (liter) POD 0 | A Abrahamsson | 2017 | 1.03 (0.53-2.00) ^a^ |
| Crystalloid (ml/kg) | Ji Hoon Sim | 2021 | 1.00 (1.00-1.01) ^a^ |
| Crystalloids (liter) | A. Arjona-Sanchez | 2016 | 1.00 (1.00-1.00) ^a d^ |
| Intraoperative crystalloid (liter) | Bo Rim Kim | 2021 | 1.20 (1.06-1.37) ^b^ |
| Colloids (liter) | A. Arjona-Sanchez | 2016 | 1.00 (0.99-1.01) ^a d^ |
| Colloid (ml/kg) | Ji Hoon Sim | 2021 | 0.99 (0.96-1.01) ^a^ |
| Intraoperative synthetic colloid use | Ji Hoon Sim | 2021 | 1.99 (1.05-3.80) ^b^ |
|  | Ji Hoon Sim | 2021 | 0.98 (0.75-1.27) ^b^ |
|  | Minjae Kim | 2021 | 1.84 (1.08-3.14) ^b^ |
|  | Bo Rim Kim | 2021 | 2.24 (1.54-3.26) ^b^ |
|  | Tak Kyu oh | 2019 | 1.79 (1.24-2.58) ^b^ |
|  | Or Goren | 2017 | 0.80 (0.30-2.80) ^a^ |
|  | Catarina Teixeira | 2014 | 1.60 (0.90-3.00) ^b^ |
| Intraoperative RBC transfusion (yes/no) | Ji Hoon Sim | 2021 | 1.18 (0.45-3.08) ^b^ |
|  | Ji Hoon Sim | 2021 | 1.04 (0.51-2.11) ^b^ |
|  | Bo Rim Kim | 2021 | 1.58 (1.06-2.35) ^b^ |
|  | Zayan Mahmooth | 2020 | 2.20 (0.60-8.06) ^b^ |
|  | Chang Seong Kim | 2013 | 1.72 (1.38-2.15) |
|  | Minjae Kim | 2021 | 1.60 (0.73-3.50) ^b^ |
| Laparoscopic surgery | Ji Hoon Sim | 2021 | 1.05 (0.80-1.39) ^b^ |
|  | Andrés Zorrilla‑Vaca | 2021 | 0.39 (0.24-0.62) ^b^ |
|  | S. T. Collaborative | 2018 | 0.76 (1.80-4.40) ^b^ |
|  | Stefano Romagnoli | 2016 | 0.26 (0.09-0.75) ^b^ |
|  | Catarina Teixeira | 2014 | 0.60 (0.30-1.20) ^a^ |
|  | Ian M. Paquette | 2013 | 0.69 (0.15-3.17) ^a^ |
| Emergent surgery | Bo Rim Kim | 2021 | 1.74 (0.89-3.40) ^a^ |
|  | Alexandra Briggs | 2018 | 1.59 (1.34-1.88) ^b^ |
|  | Thorir E Long | 2016 | 1.46 (1.05-2.03) ^a^ |
|  | Ji-Yeon Bang | 2016 | 1.89 (1.03-3.49) ^b^ |
|  | AJ Vaught | 2015 | 4.10 (2.84-5.67) ^b^ |
|  | Catarina Teixeira | 2014 | 1.30 (0.80-2.10) ^a^ |
| Lowest MBP (mmHg) | Ji Hoon Sim | 2021 | 1.00 (0.99-1.02) ^b^ |
| SBP means (10 mmHg) | Minjae Kim | 2021 | 0.87 (0.75-1.01) ^b^ |
| DBP coefficient of variation | Minjae Kim | 2021 | 1.24 (1.01-1.50) ^b^ |
| Minutes with MAP > 90 mmHg (5 min) | Minjae Kim | 2021 | 1.02 (1.01-1.03) ^b^ |
| Intraoperative hypotension (MAP <60mmHg) | Zayan Mahmooth | 2020 | 0.76 (0.41-1.44) ^b^ |
| Intraoperative average MBP (mmHg) | Bo Rim Kim | 2021 | 0.98 (0.96-1.00) ^b^ |
| Hypotension (< 80% baseline value) frequency | Xiaowei Guo | 2020 | 1.28 (1.04-1.57) ^b^ |
| Duration of hypotension (< 80% baseline value) | Xiaowei Guo | 2020 | 1.03 (0.99-1.07) ^a^ |
| MBP < 60 mmHg during surgery over 1 min | Tak Kyu oh | 2019 | 1.04 (0.77-1.40) ^b^ |
| Lowest MAP during surgery | A Abrahamsson | 2017 | 0.97 (0.87-1.09) ^a^ |
| ΔMAP during surgery | A Abrahamsson | 2017 | 0.99 (0.93-1.05) ^a^ |
| MAP (mmHg) | A. Arjona-Sanchez | 2016 | 0.99 (0.99-1.05) ^a d^ |
| MAP (mmHg, at initial laparotomy) | Tyler J. Loftus | 2017 | 0.97 (0.95-0.98) ^b c^ |
| Intraoperative hypotension (MAP <65mmHg) (per record) | Catarina Teixeira | 2014 | 1.10 (0.90-1.20) ^b^ |
| Intraoperative hypotension (SBP<90mmHg) | Chang Seong Kim | 2013 | 1.02 (0.82-1.28) ^b^ |
| ACEi, angiotensin converting enzyme inhibitor; ARB angiotensin II receptor blocker; AKI, acute kidney injury; BMI, body mass index; BUN, Blood Urea Nitrogen; CKD, chronic kidney disease; COPD, chronic obstructive pulmonary disease; ICU, intensive care unit; MAP, mean arterial blood pressure; MBP, mean blood pressure; PNI, prognostic nutritional index, PNI=[10 × serum albumin (g/dL)] + [0.005 × total lymphocyte count (per mm^3^)]; POD, post-operative day; RBC, red blood cell.  ^a^ Univariate analysis to risk factors of postoperative acute kidney injury. ^b^ Multivariate analysis to risk factors of postoperative acute kidney injury. ^C^ Risk factors for AKI stage 2 and 3. ^d^ Risk factors for AKI RIFLE-I. | | | |

Table S10 Characteristics and outcomes of Randomized Controlled Trials Evaluating perioperative hemodynamic management strategies

| Study | Year | Country | Study design | N (Intervention/Control) | Age (years) | Male (%) | Surgery type | Intervention | Control | AKI definition | AKI incidence intervention/control n (%) |
| --- | --- | --- | --- | --- | --- | --- | --- | --- | --- | --- | --- |
| Javier Ripollés-Melchor | 2025 | Spain | Multicenter RCT | 917（459/458） | HPI: 71 (65-77)  Control: 70 (63-76) | 533(58） | Moderate- to high-risk elective abdominal surgery | HPI-guided hemodynamic management (intervention when HPI >80) | Standard care based on local clinical practice | KDIGO | 28/459 (6.1) vs 32/458 (7.0) |
| Marc-Olivier Fischer | 2020 | France | Single-center RCT | 159 (80/79) | PPG-guided: 58 (16)  Control: 59 (15) | 91(57) | Elective colorectal surgery (laparoscopic or open) | PPG-guided hemodynamic optimization using stroke volume maximization | Intermittent non-invasive blood pressure monitoring | KDIGO | 1/79 (1.3) vs  3/80 (3.8) |
| John Diaper | 2021 | Switzerland | Single-center RCT | 394 (196/198) | GDHT: 65 (14)  RNT: 64 (12) | 252 (64) | Major open abdominal, urological, or vascular surgery (≥2 hours) | Goal-directed hemodynamic therapy (GDHT) with cardiac output monitoring | Restrictive normovolemic therapy (RNT) | 25% eGFR decrease | 48/196 (24.5) vs  37/198 (18.9) |
| J.M. Calvo-Vecino | 2018 | Spain | Multicenter RCT | 420 (209/211) | GDHT: 66.3 (71)  Control: 64.2 (67) | 260 (49) | Major elective abdominal, urological, gynecological, or orthopedic surgery | Oesophageal Doppler monitor-guided GDHT (stroke volume maximization, MAP >70 mmHg, CI ≥2.5 L/min/m²) | Traditional fluid management | NA | 3/209 (1.4) vs  18/211 (8.5) |
| RupertM.Pearse | 2014 | United Kingdom | Multicenter RCT | 733 (368/365) | Protocol: 71.3 (8.4)  Control: 72.2 (8.6) | 466 (63.5) | Major gastrointestinal surgery (>90 minutes duration) | Cardiac output-guided hemodynamic therapy algorithm with IV fluid and dopexamine during and 6 hours post-surgery | Usual care | NA | 17/366 (4.6) vs  17/364 (4.7) |
| OPTIMISE II Trial Group | 2024 | International (11 countries, 55 hospitals) | Multicenter RCT | 2498 (1251/1247) | Protocol:74.0 (6.4)  Control: 73.9 (6.3) | 1432 (57.3) | Major elective gastrointestinal surgery (>90 minutes) | Minimally invasive cardiac output-guided IV fluid therapy with low dose inotrope during and 4 hours post-surgery | Usual care without cardiac output monitoring | KDIGO | 40/1250 (3.2) vs  32/1247 (2.6) |
| Sandra Funcke | 2024 | Germany & Spain | Multicenter RCT | 318 (152/166) | Protocol:68 (61-74)  Control: 64 (55-75) | 191(60.1) | Elective major open abdominal surgery (>2 hours, >2L IV fluids) | Cardiac index-guided therapy to maintain optimized postinduction cardiac index using IV fluids and dobutamine during and 8h post-surgery | Routine care | NA | 15/152 (9.9) vs  10/166 (6) |
| Julia Y. Nicklas | 2020 | Germany | Single-center RCT | 188 (94/94) | Protocol:63 (14)  Control: 63 (14) | 114 (60.6) | Major abdominal surgery (≥90 min or blood loss >1000ml) | Personalized hemodynamic management targeting individual baseline cardiac index using IV fluids and/or dobutamine | Routine management | KDIGO | 5/94 (5) vs  10/94 (11) |
| Pavel Szturz | 2019 | Czech Republic | Single-center RCT | 140 (71/69) | Protocol:66 (11)  Control: 65 (10) | 84 (60) | Open gastrointestinal surgery (>120 min or blood loss >15% blood volume) | Multi-parametric pGDT using esophageal Doppler (corrected flow time, peak velocity, SVRI) | Standard care | NA | 8/71 (11.3) vs  13/69 (18.8) |
| Eric E.C. de Waal | 2021 | Netherlands | Multicenter RCT | 482 (241/241) | Protocol:66.2 (11.7)  Control: 64.7 (11.1) | 321 (66.6) | High-risk elective abdominal surgery (esophagectomy, pancreaticoduodenectomy, AAA repair, etc.) | Age-specific PGDT algorithm using CI and SVV for 24h perioperative period | Standard care | NA | 12/248 (4.8) vs  10/234 (4.3) |
| Sebastian Schmid | 2016 | Germany | Single-center RCT | 180 (90/90) | GDT: 67(12)  Control: 65 (11) | 138 | Major abdominal surgery (≥3h, expected ICU >3 days) | Algorithm-guided GDT using transpulmonary thermodilution (PiCCO2) | Standard clinical care | KDIGO | 53/92 (58) vs  45/88 (51) |
| Iris M. Jongerius | 2021 | Netherlands | Single-center RCT | 40 (20/20) | GDT: 67 (58, 70)  Control: 66 (54, 74) | 23 (57.5) | Elective open left or right (extended) hem hepatectomy | Goal-directed ﬂuid therapy | Low central venous pressure | RIFLE | 8/20 (40) vs  5/20 (25) |
| P.S. Myles | 2018 | International (Australia, New Zealand) | Multicenter RCT | 2983 (1490/1493) | Restrictive: 66 (13)  Liberal:  66 (13) | 1554 (52) | Major abdominal surgery (≥2h, ≥3 days stay, increased risk) | Restrictive IV fluid regimen | Liberal IV fluid regimen (traditional care) | RIFLE | 124/1443 (8.6) vs  72/1439 (5.0) |
| Ana B. Serrano | 2016 | Spain | Single-center RCT | 328 (164/164) | Protocol:64.6 (14.9)  Control: 62.0 (15.6) | 179 (54.6) | Major elective open abdominal surgery (ASA II-IV) | Preoperative 0.9% normal saline 1.5 ml/kg/h for 12h before surgery | No fluid intervention | AKIN | 17/159 (11.4%) vs 11/140 (7.9%) |
| Toshinari Suzuki | 2020 | Japan | Single-center RCT | 50 (25/25) | HES: 70 (62–75)  Albumin: 65 (60–71) | 37 (74) | Hepatic or pancreatic elective surgery (ASA 1-2) | 6% HES 130/0.4/9 (2 mL/kg/h) + Ringer's acetate (3 mL/kg/h) | 5% albumin (2 mL/kg/h) + Ringer's acetate (3 mL/kg/h) | KDIGO | 3/25 (12) VS  3/25 (12) |
| Barbara Kabon | 2019 | International (Austria, USA) | Multicenter double-blind RCT | 1057 (523/534) | Colloids: 52 (16)  Crystalloid: 52 (16) | 547 (51.8) | Moderate to high-risk open and laparoscopic abdominal surgery | Doppler-guided 6% HES 130/0.4 | Doppler-guided lactated Ringer's solution | KDIGO | 21/523 (4) vs  16/534 (3) |
| Alexandre Joosten | 2017 | Belgium | Bi-center double-blind RCT | 160 (80/80) | Colloids: 65 (53-73)  Crystalloid: 62 (48-70) | 96 (60) | Major elective open abdominal surgery (≥3h) | Closed-loop goal-directed balanced colloid (Volulyte) + maintenance crystalloid (3 ml/kg/h) | Closed-loop goal-directed balanced crystalloid (Plasmalyte) + maintenance crystalloid (3 ml/kg/h) | KDIGO | 19/80 (23.8) vs  23/80 (28.8) |
| Emmanuel Futier | 2020 | France | Multicenter double-blind RCT | 775 (389/386) | Colloids:68 (7)  Crystalloid: 69 (7) | 684 (88.3) | Major abdominal surgery (high-risk patients, AKI risk index ≥3) | 6% HES 130/0.4 in 0.9% saline (250mL boluses) | 0.9% saline alone (250mL boluses) | KDIGO | 85/389 (21.9) vs  63/386 (16.3) |
| Zhaohua Pang | 2025 | China | Single-center pilot RCT | 179 (89/90) | Protocol:69 (6)  Control: 68 (5) | 115 (64.2) | Elective major abdominal surgery (laparoscopic gastric and intestinal tumor resection, ≥60 years) | Individualized BP management + goal-directed fluid therapy with HES | Routine BP management (SBP≥90 mmHg, MAP≥65 mmHg) + goal-directed fluid therapy with HES | KDIGO | 10/89 (11.2) vs  14/90 (15.6) |
| Emmanuel Futier | 2017 | France | Multicenter RCT | 292 (147/145) | Protocol: 69.7 (7.1)  Control: 70.0 (7.5) | 248 (84.9) | Major surgery (predominantly abdominal, ≥2h, high-risk patients with AKI risk index ≥III) | Individualized BP management (SBP within ±10% of reference) with norepinephrine | Standard BP management (SBP <80 mmHg or <40% from reference) with ephedrine | RIFLE | 48/147 (32.7) VS  71/145 (49) |
| POISE-3 Trial Investigators | 2025 | International (22 countries, 110 hospitals) | Multicenter RCT (pre-specified substudy) | 7307 (3654/3653) | Protocol: 69.8 (9.2)  Control: 69.7 (9.3) | 4085 (55.9) | Noncardiac surgery | Hypotension-avoidance strategy: Target intraoperative MAP ≥80 mmHg + withhold RASI perioperatively | Hypertension-avoidance strategy: Target intraoperative MAP ≥60 mmHg + continue all antihypertensives | KDIGO | 531/3502 (15.2) vs  505/3504 (14.4) |
| Carlos Ferrando | 2020 | Spain (21 university hospitals) | Multicenter RCT | 717 (362/355) | High FiO_2_: 64.2 (12.8)  Conventional: 63.9 (13.9) | 446 (62.2) | Abdominal surgery | High FiO2 (0.80) + individualized open-lung ventilation strategy | Conventional FiO2 (0.30) + individualized open-lung ventilation strategy | NA | 20/362 (5.7) vs  18/355 (5.0) |
| Xue-Fei Li | 2020 | China | Single-center RCT | 251 (126/125) | High FiO_2_: 54 (14)  Low FiO_2_:53 (13) | 146 (58.2) | Elective abdominal surgery (≥2h duration) | High FiO_2_ (80%) + lung-protective ventilation | Low FiO_2_ (30%) + lung-protective ventilation | NA | 0/126 vs  1/125 |
| Yoon Jung Kim | 2023 | Korea | Single-center RCT | 384 (178/185) | Protocol: 67 (39-83)  Control: 67 (38-87) | 297 (77.3) | Laparoscopic or robotic lower abdominal surgery (steep Trendelenburg position) | Driving pressure-guided individualized PEEP | Fixed PEEP 5 cmH2O | KDIGO | 9/178 (5.4) vs  20/185 (10.8)  P = 0.068 |
| Dharshi Karalapillai | 2020 | Australia | Single-center RCT | 1206 (614/592) | Protocol: 63.5 (11.8)  Control: 63.8 (12.1) | 712 (59) | Major noncardiothoracic, nonintracranial surgery (>2h) | Low tidal volume (6 mL/kg PBW) + PEEP 5 cmH2O | Conventional tidal volume (10 mL/kg PBW) + PEEP 5 cmH2O | RIFLE | 36/420 (8.6) vs  42/394 (10.7)  P = 0.31 |
| Toby Richards | 2020 | UK | Multicenter double-blind RCT | 487 (244/243) | Protocol: 66 (57–72)  Control: 65 (50–72) | 220 (45.2) | Major open elective abdominal surgery (>1h) | Preoperative intravenous iron (ferric carboxymaltose 1000mg) | Placebo (100mL normal saline) | KDIGO | 11/137 (8) vs  13/122 (11) |
| P.J. Devereaux | 2014 | International (23 countries, 135 hospitals) | Multicenter RCT (2×2 factorial design) | 10,010 (4,998/5,012) | Aspirin: 68.6 (10.3)  Placebo: 68.6 (10.3) | 5283 (52.8) | Noncardiac surgery (at risk for vascular complications) | Aspirin (200mg pre-op, then 100mg daily) | Placebo | Acute kidney injury with receipt of dialysis | 33/4998 (0.7) vs  19/5012 (0.4)  P = 0.05 |
| Jan Pallesen | 2022 | Denmark | Multicenter RCT | 327 (163/164) | FOCUS: 81 (8)  Control: 81 (8) | 135 (41.3) | Urgent orthopedic or abdominal surgery | Preoperative FOCUS (FATE protocol) | No preoperative FOCUS | KDIGO | 17/163 (10) vs  14/164 (9)  P = 0.69 |
| Xuecai Lv | 2024 | China | Multicenter RCT | 154 (78/76) | Intervention: 70.9 (5.3)  Control: 69.9 (4.8) | 107 (69.5) | Gastrointestinal surgery (gastrectomy, enterectomy) | Perioperative rehabilitation exercise program | Usual care | NA | 1/78 (1.3) vs  1/76 (1.3)  P = 0.985 |
| Rahul Kumar Chaudhary | 2025 | India | Single-center double-blind RCT | 90 (45/45) | BCS: 38.51 (12.76)  ISB: 38.44 (14.03) | 74 (82.2) | Emergency laparotomy for perforation peritonitis | Isotonic sodium bicarbonate (ISB) | Balanced crystalloid solution (Ringer's Lactate) | NA | 11/45 (24.4) VS  4/45 (8.9)  P = 0.048 |
