# Supplementary figures and images for: Incidence and risk factors of acute kidney injury after abdominal surgery: a systematic review and meta-analysis
Source: Ann Med. 2025 Aug 17;57(1):2547324. doi: 10.1080/07853890.2025.2547324 (PMC12360056; doi:10.1080/07853890.2025.2547324)

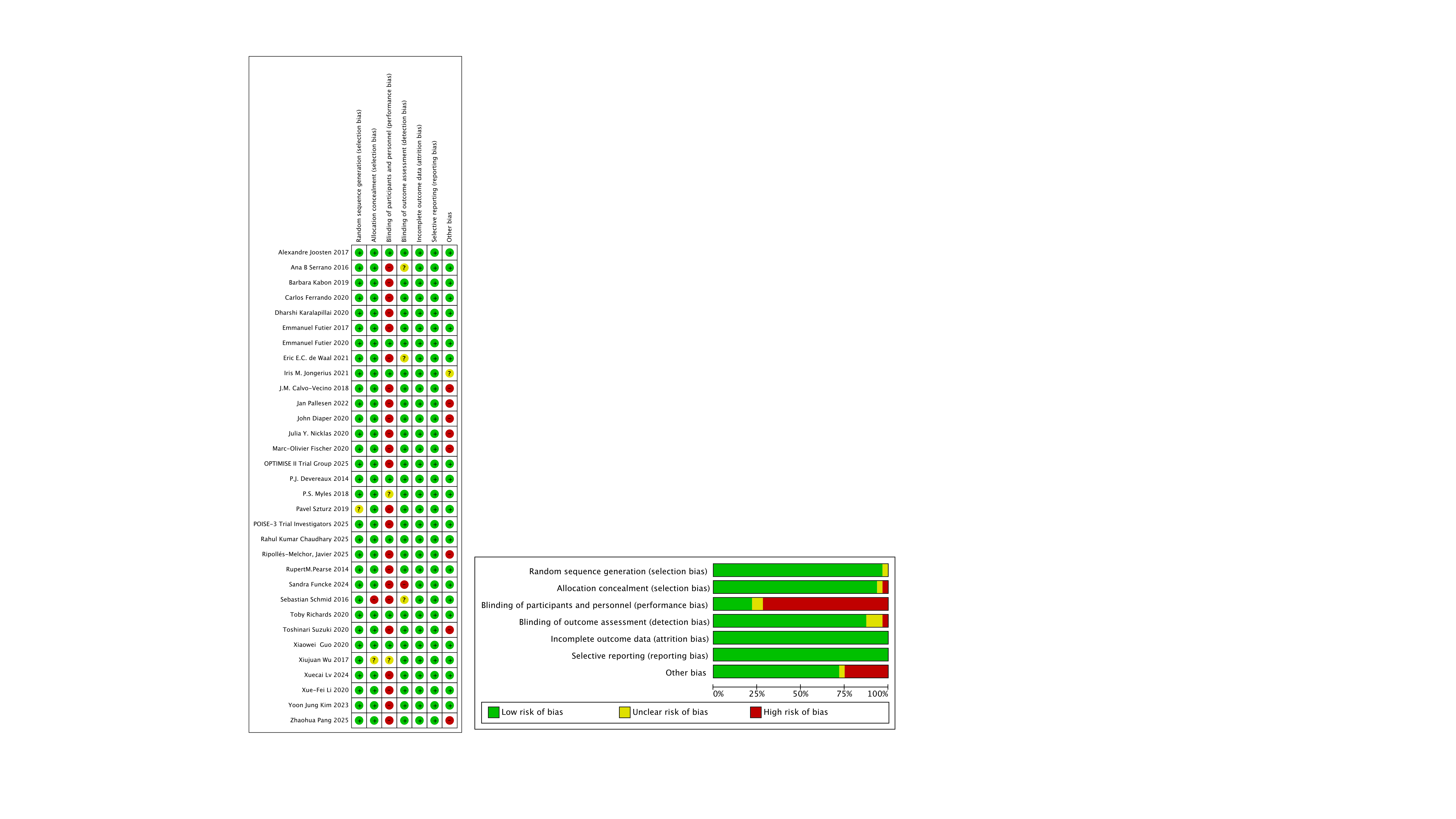

Supplement: Supplemental Material [file IANN_A_2547324_SM9117.zip › Suppl_data/Figure S1.jpg]

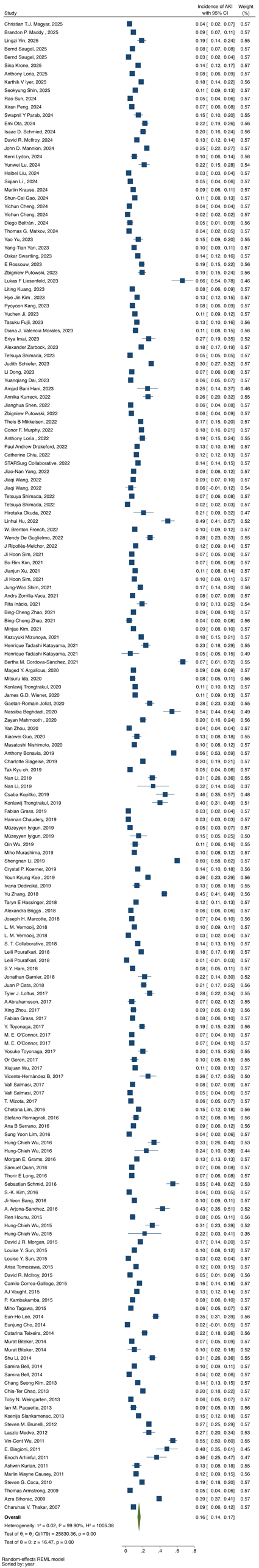

Supplement: Supplemental Material [file IANN_A_2547324_SM9117.zip › Suppl_data/Figure S2.jpg]

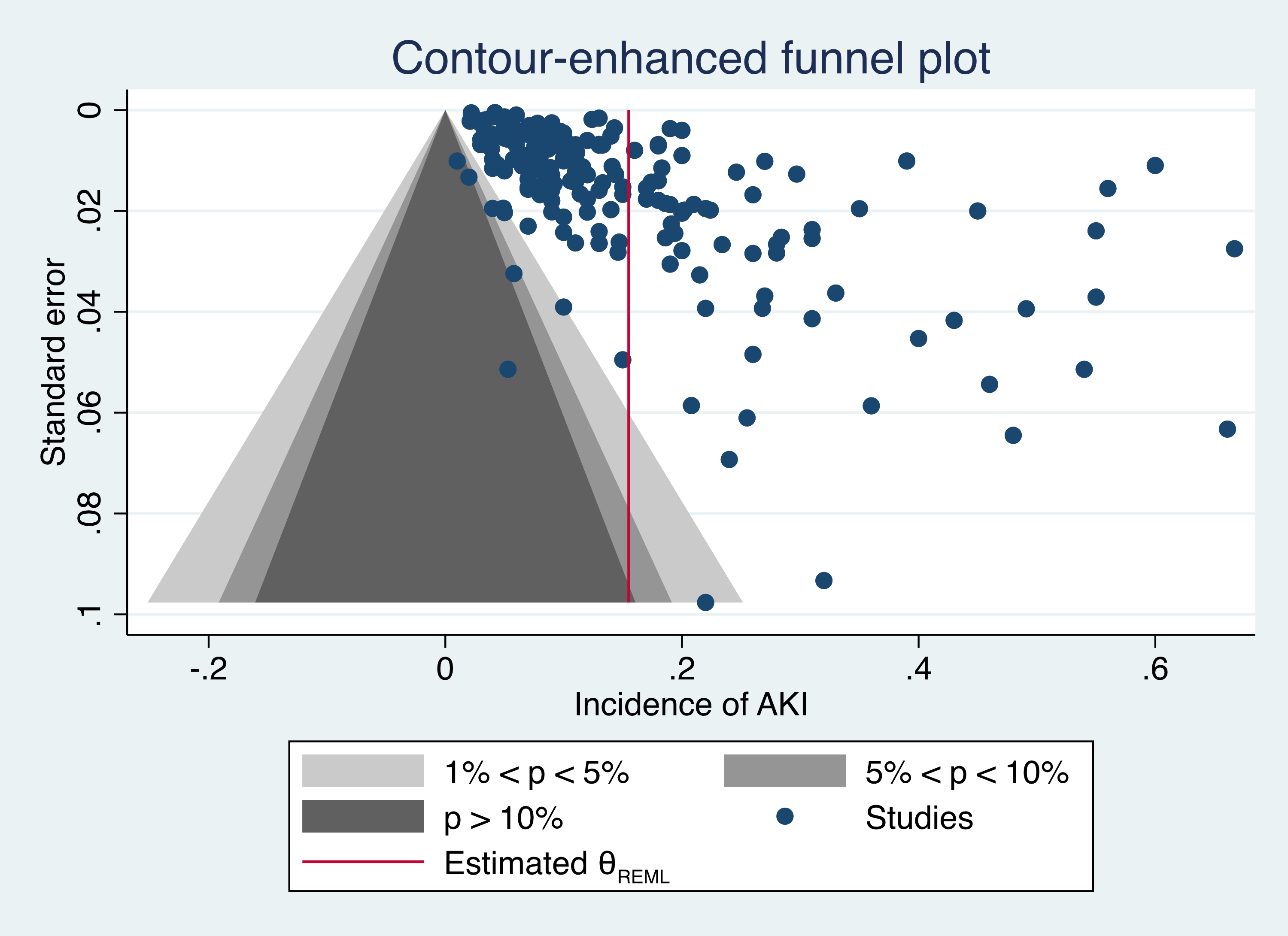

Supplement: Supplemental Material [file IANN_A_2547324_SM9117.zip › Suppl_data/Figure S3.jpg]

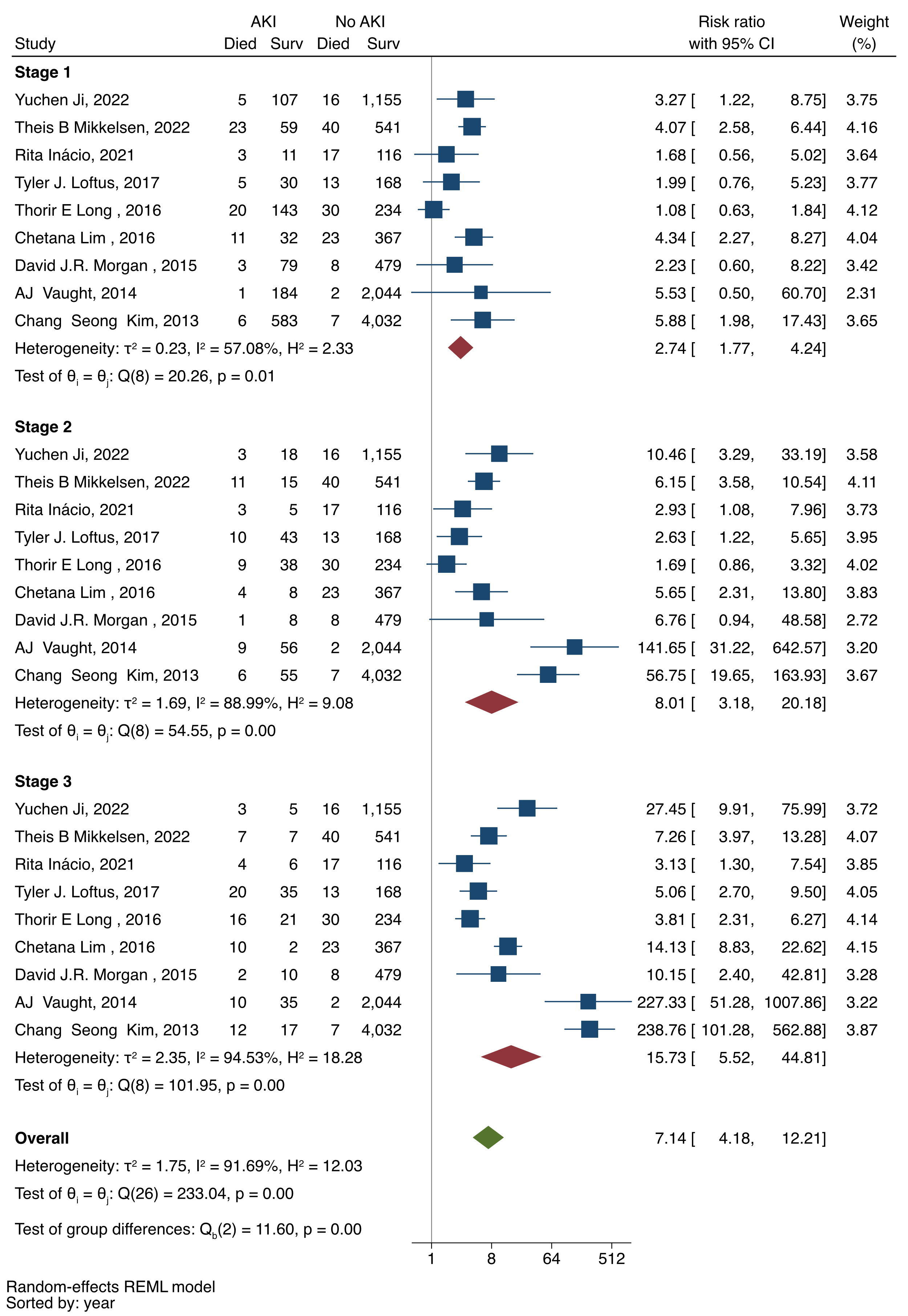

Supplement: Supplemental Material [file IANN_A_2547324_SM9117.zip › Suppl_data/Figure S4.jpg]

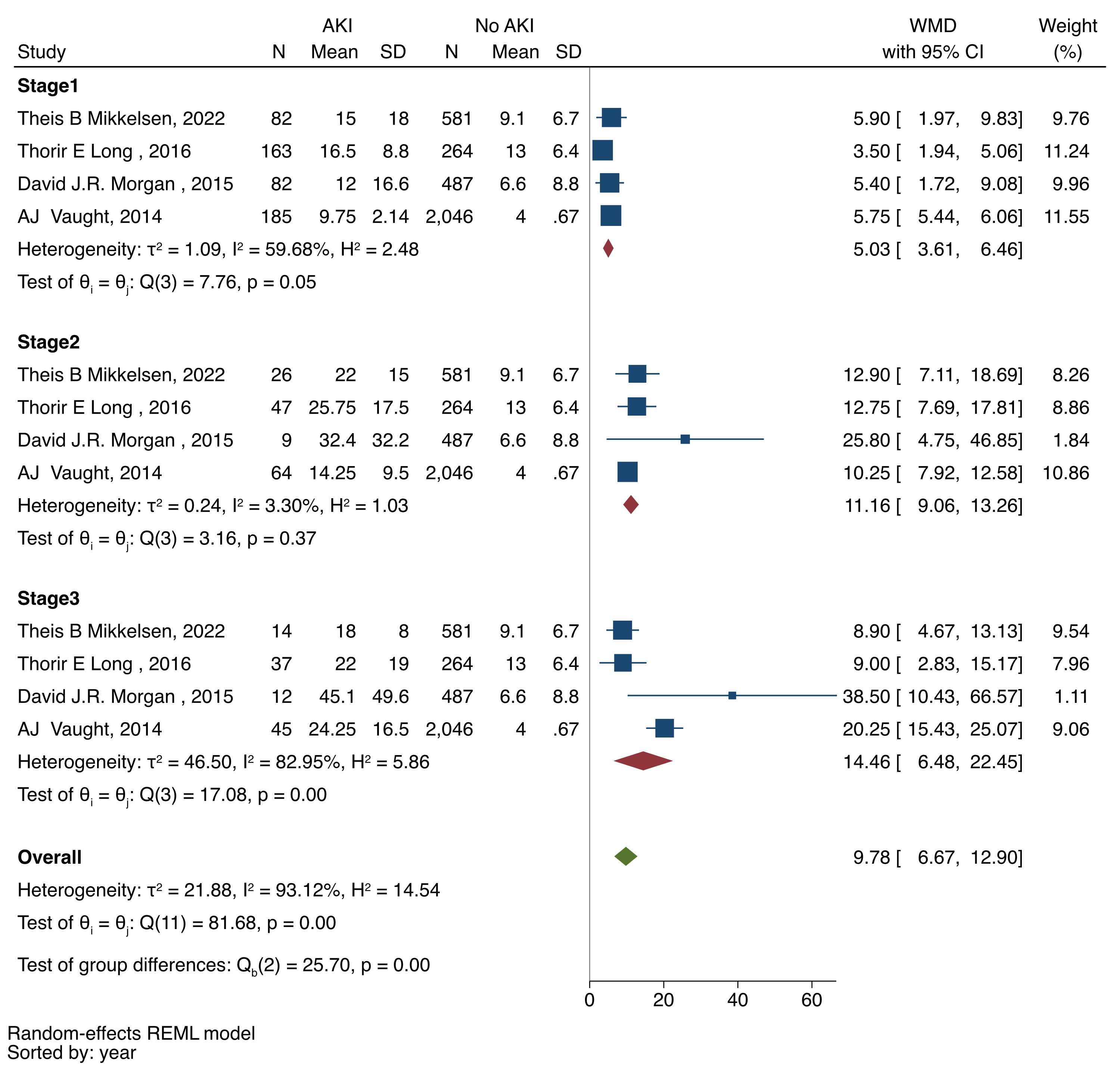

Supplement: Supplemental Material [file IANN_A_2547324_SM9117.zip › Suppl_data/Figure S5.jpg]
